# RNA sequencing-based identification of microRNAs in the antler cartilage of Gansu red deer (*Cervus elaphus kansuensis*)

**DOI:** 10.7717/peerj.13947

**Published:** 2022-09-21

**Authors:** Yanxia Chen, Zhenxiang Zhang, Jingjing Zhang, Xiaxia Chen, Yuqin Guo, Changzhong Li

**Affiliations:** 1 College of Eco-Environmental Engineering, Qinghai University, Xining, Qinghai, China; 2 Academy of Animal Science and Veterinary Medicine, Qinghai University, Xining, Qinghai, China; 3 School of Life Sciences and Engineering, Hexi University, Zhangye, Gansu, China; 4 Research Monitoring and Evaluation Center of Qinghai National Park, Xining, Qinghai, China

**Keywords:** *Cervus elaphus kansuensis*, Gansu red deer, Velvet antler, Rapid growth, microRNAs

## Abstract

**Background:**

The velvet antler is a complex mammalian bone organ with unique biological characteristics, such as regeneration. The rapid growth stage (RGS) is a special period in the regeneration process of velvet antler.

**Methods:**

To elucidate the functions of microRNAs (miRNAs) at the RGS of antler development in Gansu red deer (*Cervus elaphus kansuensis*), we used RNA sequencing (RNA-seq) to analyze miRNA expression profiles in cartilage tissues of deer antler tips at three different growth stages.

**Results:**

The RNA-seq results revealed 1,073 known and 204 novel miRNAs, including 1,207, 1,242, and 1,204 from 30-, 60-, and 90-d antler cartilage tissues, respectively. To identify key miRNAs controlling rapid antler growth, we predicted target genes of screened 25 differentially expressed miRNAs (DEMs) and specifically expressed miRNAs (SEMs) in 60 d and annotated their functions. The KEGG results revealed that target genes of 25 DEMs and 30 SEMs were highly classified in the “Metabolic pathways”, “Pathways in cancer”, “Proteoglycans in cancer” and “PI3K-Akt signaling pathway”. In addition, a novel miRNA (CM008039.1_315920), highly enriched in “NF-kappa B signaling pathway”, may need further study.

**Conclusions:**

The miRNAs identified in our study are potentially important in rapid antler growth. Our findings provide new insights to help elucidate the miRNA-mediated regulatory mechanisms involved during velvet antler development in *C. elaphus kansuensis*.

## Introduction

Velvet antlers, the bone-free and furry horns in male deer, develop through a highly complicated and genetically programmed process facilitating the growth of bone, skin, blood vessels and nerves. The proliferation and differentiation of cells in growing antler tips are regulated by several intracellular and extracellular factors and signaling pathways (Hu et al., 2019). The growth process of velvet antlers is very similar to endochondral ossification of limbs, which drives cartilage formation through mesenchymal cell differentiation. Therefore, velvet antlers are often used as a model to study cartilage and bone tissue regeneration and injury repair (Price & Allen, 2004).

During their rapid growth phase, antlers can exceed 2 cm/day. This is a hot topic in the field of biology (Price, Faucheux & Allen, 2005; Chu et al., 2017). Combining data on growth rate, shedding time and age, the fastest growing period of velvet antlers was ∼60 days after shedding, indicating that this is a critical stage during antler development. However, the underlying mechanisms involved in the rapid growth stage (RGS) of antler development remains unknown. Deer antler growth is mainly controlled by the growth center located at antler tips, comprising velvet skin, mesenchyme and cartilage tissue (Li et al., 2002). The cartilage zone dominates a large part of the antler growth center, and hence, plays a key role during the RGS of antler development (Ba et al., 2019).

MicroRNAs (miRNAs) are a class of small non-coding RNAs (ncRNAs), usually 17–22 nucleotides long, which are critical in post-transcriptional gene regulation via target messenger RNA (mRNA) degradation or via translation inhibition. MicroRNAs function through pairing with complementary sequences in the 3′-untranslated region (3′-UTR) of their target mRNAs (Mohr & Mott, 2015). These small regulatory molecules are ubiquitous in the genomes of animals, plants and even some viruses (Lu & Rothenberg, 2018). Previous studies have provided a considerable amount of information on the miRNA-mediated regulation of various biological processes, including organ development (Zhang et al., 2016a; Zhang et al., 2016b), phase transition (Wójcik & Gaj, 2016; Zhang et al., 2016a; Zhang et al., 2016b) and stress response (Candar-Cakir, Arican & Zhang, 2016; Li et al., 2016). Furthermore, recent studies have reported that specific miRNAs participate in the regulation of cartilage development, degradation and integrity (Zhang et al., 2019; Liu et al., 2020; Tu et al., 2020).

Deep sequencing methods now provide a fast and convenient way to identify and profile small RNA populations in various tissues and at different developmental stages (Fahlgren et al., 2007). Recent advances in RNA sequencing (RNA-seq) have also provided new opportunities to integrate changes in protein-coding mRNAs with regulatory signals, such as those driven by ncRNAs and alternative RNA splicing events (Ackerman 4th et al., 2018). RNA-seq has been widely used to study miRNA expression in humans, other animals and plants (Chiba et al., 2021; Hao et al., 2021; Wang et al., 2021; Yang et al., 2021).

To elucidate miRNA function during the RGS of antler development, we investigated miRNA expression profiles in the cartilage of antler growth centers at three developmental stages, specifically at 30, 60, and 90 days (d). During the proliferation stage, antlers grow at an extremely rapid rate without any carcinogenesis, which is unique among mammals. This growth characteristic makes deer antlers an ideal medical model for studying cancer treatment. The longitudinal growth of velvet antler is achieved through osteogenesis in the cartilage of each branch, very similar to the cartilage formation process in mammals. Thus, the velvet antler is a model of cartilage damage repair. Our findings may contribute new insights into the roles of miRNAs and their target mRNAs in the complex regulatory networks affecting antler development.

## Materials & Methods

### Ethics approval statement

All experimental protocols were approved by the Institutional Animal Care and Use Committee of Qinghai University (Xining, China), and all methods were carried out in accordance with approved guidelines and regulations (code: SL-2022024). All procedures involving animals were conducted in accordance with the US National Institutes of Health Guide for the Care and Use of Laboratory Animals (National Academies Council, 2011). This study was carried out in compliance with the ARRIVE guidelines for reporting animal research (Percie du Sert et al., 2020).

### Animals and samples collection

Samples were collected from a *C. elaphus kansuensis* population of 200 individuals, raised in a semi-wild setting and fed under the same conditions (Shandan County, Gansu Province, China). Cartilage tissues in the antler tips at different growth stages (30, 60, and 90 d) were collected from three healthy adult individuals (4–5 years old, male) for RNA extraction. Three antler samples from three different individuals were prepared for RNA-seq and qRT-PCR. Antlers were collected after anaesthetising deer with special Mian Naining (anaesthetic, No.9812, People’s Liberation Army (PLA) Military Supplies University Research Institute, China), and hemostasis measures were taken immediately after velvet cutting. After recovering from anaesthesia, deer were returned to the herd.

### RNA extraction, library preparation, and sequencing

Total RNA was extracted using TRIzol™ Reagent (Thermo Fisher Scientific, Waltham, MA, USA) following the manufacturer’s protocol. Extracted RNA was treated with DNase I (Promega, Madison, WI, USA) to remove contamination. To ensure that the RNA samples were qualified for sequencing, concentration and integrity were assessed using a NanoDrop 2000 Spectrophotometer (Thermo Fisher Scientific, Waltham, MA, USA) and RNA Nano 6000 Assay Kit with the Bioanalyzer 2100 System (Agilent Technologies, Santa Clara, CA, USA), respectively.

In this study, sample collection, high-throughput sequencing and data collection were conducted as previously described in Chen et al. (2022). Specifically, a total of 2.5 ng RNA per sample was used as input material for library preparation. Sequencing libraries were generated using NEBNext® Ultra™ RNA Library Prep Kit for Illumina® (New England Biolab, Ipswich, MA, USA) following manufacturer’s protocol, and index codes were added to attribute sequences. Library quality was assessed using Bioanalyzer 2100 (Agilent Technologies, Santa Clara, CA, USA).

Pooled miRNA libraries were sequenced for 50 cycles using HiSeq® Rapid SBS Kit v2 and HiSeq® 2500 platform (Illumina, San Diego, CA, USA). Sequencing data are available at the National Center for Biotechnology Information (NCBI) Sequence Read Archive (SRA) (BioProject: PRJNA731682).

### Data analysis

Raw reads in FASTQ format were preprocessed using in-house Perl scripts. In this step, clean reads were obtained via trimming adapters and removing reads containing poly(A) tails, low-quality bases, and sequences <18 or >30 nucleotides long. Additionally, Q20 and Q30 scores, GC content (%), and sequence duplication level of cleaned data were determined.

Second, cleaned reads were aligned against the Silva (Quast et al., 2013) (https://www.arb-silva.de/), Genomic tRNA (Chan & Lowe, 2016) (GtRNAdb; http://lowelab.ucsc.edu/GtRNAdb/), Rfam (Kalvari et al., 2018) (http://rfam.xfam.org/), and Repbase (Bao, Kojima & Kohany, 2015) (http://www.girinst.org/repbase) databases using Bowtie (Langmead et al., 2009) to remove sequences that matched to rRNAs, tRNAs, snRNAs, snoRNAs, other ncRNAs, and repeats. Then clean small RNAs with a length range from 18 to 35 nucleotides were further analyzed. Third, Randfold tools (Bonnet et al., 2004) (http://www.aquafold.com) was used for predicting novel miRNA secondary structure. Fourth, the miRBase v21.0 database (Kozomara & Griffiths-Jones, 2014) (http://www.mirbase.org/) with 0 or 1 mismatch and miRDeep2 (Friedländer et al., 2012) with default parameters were used to identify known miRNAs and predict novel miRNAs. The miRNA expression profiles were determined based on TPM values using the normalized formula (Zhao, Ye & Stanton, 2020): TPM = 10^6^ ∗ [Reads mapped to transcript/Transcript length]/[Sum (Reads mapped to transcript/Transcript length)]. (Note: “Reads mapped to the transcript” refers to read number of one miRNA, “Transcript length” refers to length of that one miRNA).

Based on the normalization counts, the significantly differentially expressed miRNAs in three stages were determined by their fold change. Based on a power analysis using the IDEG6 (Romualdi et al., 2003), we determined that our design had over 99% power to detect differentially expressed miRNAs (DEMs) at | log2(FC) | > = 1 and FDR < = 0.01 (Hou et al., 2019). We then screened for DEMs in the 30 d vs. 60 d and 60 d vs. 90 d pairwise comparisons.

### MiRNA target prediction and functional analysis of candidate target genes

We put more focus on the 25 selected DEMs and the 30 specifically expressed miRNAs in 60 d library. Potential target genes of novel miRNAs and specifically expressed miRNAs were predicted using Miranda v3.3a (https://anaconda.org/bioconda/miranda) and TargetScan 7.2 (http://www.targetscan.org/vert_72/) with default parameters. Genes simultaneously predicted by both methods were considered as candidate targets of selected miRNAs. Functional annotation of these candidates were performed using GO enrichment (Ashburner et al., 2000) and KEGG pathway (Kanehisa & Goto, 2000) analyses.

### Verification of miRNA expression using qRT-PCR

Stem-loop qRT-PCR was used to verify the accuracy of the sequencing data. Twelve of the selected 25 DEMs had similar names (except species), sequences and expression levels, and the remaining 13 DEMs were used for qRT-PCR. Total RNA was extracted as previously described. Approximately 1 µg of total RNA was reverse transcribed using Revert Aid First Strand cDNA Synthesis Kit (Fermentas, Thermo Fisher Scientific, Waltham, MA, USA). Reactions were incubated at 42 °C for 15 min and then terminated at 80 °C for 5 s. The qRT-PCR assay was performed using TB Green® Premix Ex Taq™ II (Tli RNaseH Plus) Kit (Takara Bio, Shiga, Japan) and CFX96 Real-time PCR Detection System (Bio-Rad Laboratories, Hercules, CA, USA). The miRNA expression level was detected using stem-loop qRT-PCR and miRNA-specific stem-loop primers. Gene-specific forward primers and universal reverse primers (Sangon Biotech, Shanghai, China) were used for qRT-PCR, with U6 RNA as internal control. Cycling profiles were as follows: 95 °C for 30 s, followed by 40 cycles at 95 °C for 5 s, and 60 °C for 30 s. Table 1 lists all primers used in this study. Melting curve analysis was also performed to determine product specificity. All statistical analyses were performed in triplicate. All data are presented as the mean ± standard deviation. Relative gene expression was subsequently analyzed using the comparative 2^−ΔΔCt^ method (Livak & Schmittgen, 2001; Rao et al., 2013).

**Table 1:** Primers uesd in qPCR. Primers used for reverse transcription and stem-loop real-time qPCR.

Gene ID	miRNA sequence	Primers	Sequence
U6 RNA		RTP	AACGCTTCACGAATTTGCGT
		Forward primer	CTCGCTTCGGCAGCACA
		Reverse primer	AACGCTTCACGAATTTGCG
aga-miR-184	TGGACGGAGAACTGATAAGGG	RTP	GGCCAAGCAGTGGTATCAACGCAGAGTGGCCCCCTTA
		Forward primer	CTTATCAGTTCTCCGTCCATGCGTG
		Reverse primer	AAGCAGTGGTATCAACGCAGAGT
bta-miR-146b	TGAGAACTGAATTCCATAGGCTGT	RTP	GGCCAAGCAGTGGTATCAACGCAGAGTGGCCACAGCC
		Forward primer	GCCTATGGAATTCAGTTCTCAGCGTG
		Reverse primer	AAGCAGTGGTATCAACGCAGAGT
bta-miR-187	TCGTGTCTTGTGTTGCAGCCGG	RTP	GGCCAAGCAGTGGTATCAACGCAGAGTGGCCCCGGCT
		Forward primer	TGCAACACAAGACACGAGCGTG
		Reverse primer	AAGCAGTGGTATCAACGCAGAGT
bta-miR-27a-5p	AGGGCTTAGCTGCTTGTGAGCA	RTP	GGCCAAGCAGTGGTATCAACGCAGAGTGGCCTGCTCA
		Forward primer	CAAGCAGCTAAGCCCTTGCGTG
		Reverse primer	AAGCAGTGGTATCAACGCAGAGT
bta-miR-504	AGACCCTGGTCTGCACTCTGTC	RTP	GGCCAAGCAGTGGTATCAACGCAGAGTGGCCGACAGA
		Forward primer	CAGAGTGCAGACCAGGGTCTTCATG
		Reverse primer	AAGCAGTGGTATCAACGCAGAGT
cfa-miR-10a	TACCCTGTAGATCCGAATTTGT	RTP	GGCCAAGCAGTGGTATCAACGCAGAGTGGCCACAAAT
		Forward primer	ACAAATTCGGATCTACAGGGTATGCGTG
		Reverse primer	AAGCAGTGGTATCAACGCAGAGT
chi-miR-411b-3p	TATGTCACATGGTCCACTAAT	RTP	GGCCAAGCAGTGGTATCAACGCAGAGTGGCCATTAGT
		Forward primer	ATTAGTGGACCATGTGACATAGGCGTG
		Reverse primer	AAGCAGTGGTATCAACGCAGAGT
efu-miR-181a	AACCATCGACCGTTGATTGTACC	RTP	GGCCAAGCAGTGGTATCAACGCAGAGTGGCCGGTACA
		Forward primer	ATCAACGGTCGATGGTTTGCGTG
		Reverse primer	AAGCAGTGGTATCAACGCAGAGT
oar-miR-10a	TACCCTGTAGATCCGAATTTG	RTP	GGCCAAGCAGTGGTATCAACGCAGAGTGGCCCAAATT
		Forward primer	CAAATTCGGATCTACAGGGTATGCGTG
		Reverse primer	AAGCAGTGGTATCAACGCAGAGT
ssa-miR-144-5p	GGATATCATCATATACTGTAAGTT	RTP	GGCCAAGCAGTGGTATCAACGCAGAGTGGCCAACTTA
		Forward primer	CAGTATATGATGATATCCGGCAACGCG
		Reverse primer	AAGCAGTGGTATCAACGCAGAGT
unconservative_CM008019.1_137729	CCCAGGGATGTAGCTCCTAGTGC	RTP	GGCCAAGCAGTGGTATCAACGCAGAGTGGCCGCACTA
		Forward primer	AGCTACATCCCTGGGTTATGCGTG
		Reverse primer	AAGCAGTGGTATCAACGCAGAGT
unconservative_CM008022.1_159134	CAAATTCGTGAAGCGTTCCATATTT	RTP	GGCCAAGCAGTGGTATCAACGCAGAGTGGCCAAATAT
		Forward primer	GAACGCTTCACGAATTTGTGCGTG
		Reverse primer	AAGCAGTGGTATCAACGCAGAGT
unconservative_CM008039.1_315920	CGGATCAGCTCAGTGCCGGGC	RTP	GGCCAAGCAGTGGTATCAACGCAGAGTGGCCGCCCGG
		Forward primer	CACTGAGCTGATCCGAATTGCGTG
		Reverse primer	AAGCAGTGGTATCAACGCAGAGT

**Notes.**

RTP Primer used in reverse transcription

## Results

### MiRNA sequencing

To determine miRNA function during velvet antler RGS, we constructed three small RNA libraries for antler growth centers at 30, 60, and 90 d, generating 14,971,388, 16,985,192, and 15,732,412 raw reads, respectively.

After data preprocessing, we obtained 9,836,383, 13,092,918, and 10,769,127 clean reads for 30-, 60-, and 90-d antler tips, respectively. We then aligned clean reads with Silva, GtRNAdb, Rfam, and Repbase databases using Bowtie (Langmead et al., 2009), a comparison software for short sequences, especially reads generated from highlight sequencing. After filtering out sequences that matched ribosomal RNAs (rRNAs), transfer RNAs (tRNAs), small nuclear RNAs (snRNAs), small nucleolar RNAs (snoRNAs), other ncRNAs, and repeats, we obtained 8,646,311 (30 d), 11,699,869 (60 d), and 9,461,208 (90 d) unannotated reads that only contained miRNAs.

For miRNA prediction, the remaining reads were aligned to the deer genome (https://www.ncbi.nlm.nih.gov/genome/?term=red+deer), with no mismatch allowed between unannotated reads and the reference sequence. Respectively, 5,430,521 (62.81%), 7,081,757 (60.52%), and 5,621,148 (59.41%) reads from 30-, 60-, and 90-d antler cartilage tissues were successfully mapped and annotated (Table 2).

**Table 2:** Data of miRNA-seq. Statistic for deep-sequencing results generated from antler cartilage tissues of *Cervus elaphus kansuensis*. miRNA: microRNA.

	30 d	60 d	90 d
Raw_reads	14971388	16985192	15732412
Clean_reads	9836383	13092918	10769127
rRNA	854250	996335	932966
scRNA	0	0	0
snRNA	3	4	4
snoRNA	21446	18939	15955
tRNA	188055	239173	237900
Repbase	126318	138598	121094
Unannotated Reads	8646311	11699869	9461208
Mapped_Reads	5430521	7081757	5621148
Known miRNAs	1003	1052	1021
Novel miRNAs	184	190	183

### Identification of known and novel miRNAs in velvet antler

**Figure 1:**
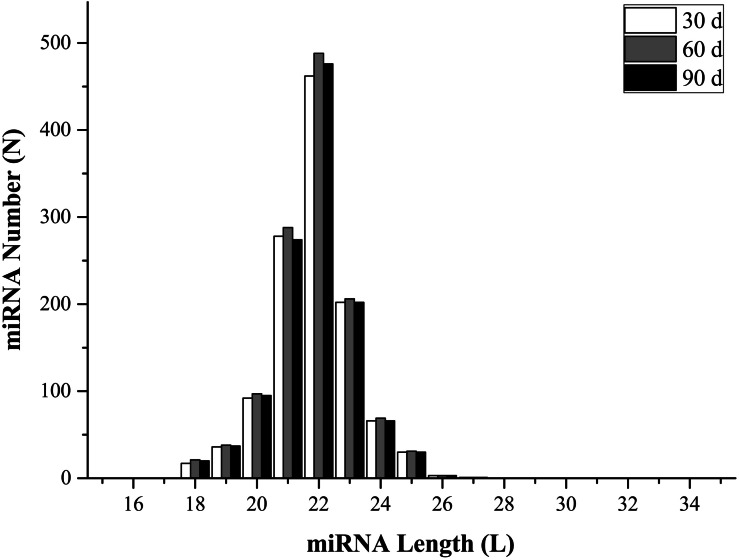
miRNAs length distribution.

To identify miRNAs in the three libraries, all mapped reads that were 18–35 nucleotides long were compared with mature animal miRNAs in the miRBase (v21.0) database. Perfectly matched sequences were considered as known miRNAs, while the remaining sequences were designated as novel miRNAs. The analysis identified 1,073 known miRNAs (1,003, 1,052, and 1,021 from 30-, 60-, and 90-d antler tips) and 204 novel miRNAs (184, 190, and 183 from 30-, 60-, and 90-d antler tips). The size distribution of identified miRNAs was similar in all samples, and most of them changed from 20 to 24 nt, which was consistent with the typical size range of miRNAs (Fig. 1; Table S1). The most abundant size class was 22 nt, which accounted for approximately 38.92%, 39.29% and 39.53% in the three libraries. All miRNAs were classified into 188 miRNA families, with the mir-2284 family being the most represented (Table S2).

The expression levels of each miRNA were normalized to the expression of tags per million (TPM) (Table S3). The top 20 known miRNAs with the highest number of reads (Table 3) accounted for 82.36%, 74.23%, and 76.69% of total reads from 30-, 60-, and 90-d antler cartilage tissues, respectively. The top 20 novel miRNAs also gathered the most reads (Table 4), accounting for 69.36%, 70.27%, and 79.65% of total reads from 30-, 60-, and 90-d antler cartilage tissues, respectively. Novel miRNAs had considerably lower sequencing frequencies than known miRNAs (Table S3). The same pattern has also been reported in other species, suggesting that novel miRNAs are typically weakly expressed and known miRNAs are highly expressed in different organisms.

**Table 3:** Top 20 known miRNAs. The 20 most abundant known miNRAs in antler cartilage of *Cervus elaphus kansuensis*.

30 d			60 d			90 d		
miRNA	Count	TPM	miRNA	Count	TPM	miRNA	Count	TPM
ggo-miR-148a	755406	220514.4	ggo-miR-148a	781385	181034.9	ggo-miR-148a	704227	206292
aca-miR-148a-3p	750540	219093.9	aca-miR-148a-3p	778239	180306	aca-miR-148a-3p	700427	205178.9
bta-miR-21-5p	215228	62828.29	bta-miR-21-5p	198752	46047.78	bta-miR-21-5p	164868	48295.45
chi-miR-21-5p	215227	62828	chi-miR-21-5p	198752	46047.78	chi-miR-21-5p	164866	48294.86
sha-miR-21	215110	62793.85	sha-miR-21	198623	46017.9	sha-miR-21	164771	48267.03
aca-miR-21-5p	215106	62792.68	aca-miR-21-5p	198623	46017.9	aca-miR-21-5p	164766	48265.57
ccr-let-7i	65670	19170.06	ccr-let-7i	114404	26505.65	ccr-let-7i	74011	21680.34
aca-let-7i-5p	65670	19170.06	aca-let-7i-5p	114404	26505.65	aca-let-7i-5p	74011	21680.34
ssc-let-7i	65670	19170.06	ssc-let-7i	114404	26505.65	ssc-let-7i	74010	21680.05
gga-let-7g-5p	38050	11107.37	gga-let-7g-5p	55889	12948.62	aca-miR-27b-3p	41660	12203.63
aca-let-7g	37827	11042.27	aca-let-7g	55593	12880.04	bta-miR-27b	41660	12203.63
aca-let-7f-5p	26320	7683.204	aca-miR-27b-3p	53525	12400.92	gga-let-7g-5p	39633	11609.85
ggo-let-7f	26320	7683.204	bta-miR-27b	53525	12400.92	aca-let-7g	39451	11556.54
aca-miR-27b-3p	25840	7543.085	aca-miR-99a-5p	49497	11467.69	aca-let-7f-5p	28763	8425.661
bta-miR-27b	25840	7543.085	bta-miR-99a-5p	49497	11467.69	ggo-let-7f	28762	8425.368
aca-let-7a-5p	16504	4817.766	oar-miR-99a	49497	11467.69	aca-miR-99a-5p	22479	6584.864
prd-let-7-5p	16495	4815.139	aca-let-7f-5p	39435	9136.484	bta-miR-99a-5p	22479	6584.864
bta-miR-199b	15746	4596.495	ggo-let-7f	39435	9136.484	oar-miR-99a	22479	6584.864
ssc-miR-199b-5p	15745	4596.203	aca-let-7a-5p	30258	7010.314	aca-let-7a-5p	22389	6558.5
aca-miR-99a-5p	13211	3856.49	prd-let-7-5p	30254	7009.387	prd-let-7-5p	22382	6556.449

**Table 4:** Top 20 novel miRNAs. The 20 most abundant novel miRNAs in antler cartilage of *Cervus elaphus kansuensis*.

30 d			60 d			90 d		
miRNA	Count	TPM	miRNA	Count	TPM	miRNA	Count	TPM
CM008037.1_299589	1225	357.596	CM008037.1_299589	1537	356.0993	CM008037.1_299589	1135	332.4801
CM008025.1_188962	504	147.1252	CM008025.1_188962	597	138.3157	CM008012.1_55433	632	185.1343
CM008041.1_334512	326	95.16431	CM008022.1_160011	417	96.61249	CM008041.1_329491	631	184.8414
CM008022.1_160011	220	64.22131	CM008027.1_215935	283	65.56675	CM008037.1_302513	631	184.8414
CM008008.1_9244	137	39.99236	CM008041.1_334512	275	63.71327	CM008025.1_188962	541	158.4773
CM008021.1_154122	121	35.32172	CM008021.1_154122	265	61.39643	CM008022.1_160011	316	92.56715
CM008029.1_241215	118	34.44598	CM008021.1_149700	182	42.1666	CM008034.1_284828	287	84.07206
CM008021.1_149700	113	32.9864	CM008029.1_241215	164	37.99628	CM008041.1_334512	264	77.33458
CM008027.1_215935	104	30.35917	CM008008.1_9244	120	27.80216	CM008027.1_215935	154	45.11184
CM008020.1_143075	93	27.1481	CM008026.1_198184	97	22.47341	CM008029.1_241215	139	40.71783
CM008027.1_214244	86	25.1047	CM008041.1_329491	85	19.69319	CM008008.1_9244	125	36.61675
CM008041.1_332283	82	23.93704	CM008037.1_302513	85	19.69319	CM008021.1_149700	119	34.85915
CM008022.1_159134	71	20.72597	CM008012.1_55433	85	19.69319	CM008021.1_154122	84	24.60646
CM008025.1_184228	71	20.72597	CM008041.1_332283	82	18.99814	CM008021.1_147678	66	19.33365
CM008012.1_63603	60	17.5149	CM008019.1_131447	77	17.83972	CM008020.1_143075	65	19.04071
CM008026.1_198184	53	15.4715	CM008027.1_214244	71	16.44961	CM008018.1_117444	63	18.45484
CM008020.1_147456	51	14.88767	CM008012.1_63603	66	15.29119	CM008012.1_63603	60	17.57604
CM008016.1_98716	50	14.59575	CM008016.1_98716	65	15.0595	CM008025.1_184228	58	16.99017
CM008021.1_151843	43	12.55235	CM008036.1_292916	60	13.90108	CM008022.1_159134	57	16.69724
CM008021.1_150571	43	12.55235	CM008020.1_147456	58	13.43771	CM008027.1_214244	51	14.93964

**Figure 2:**
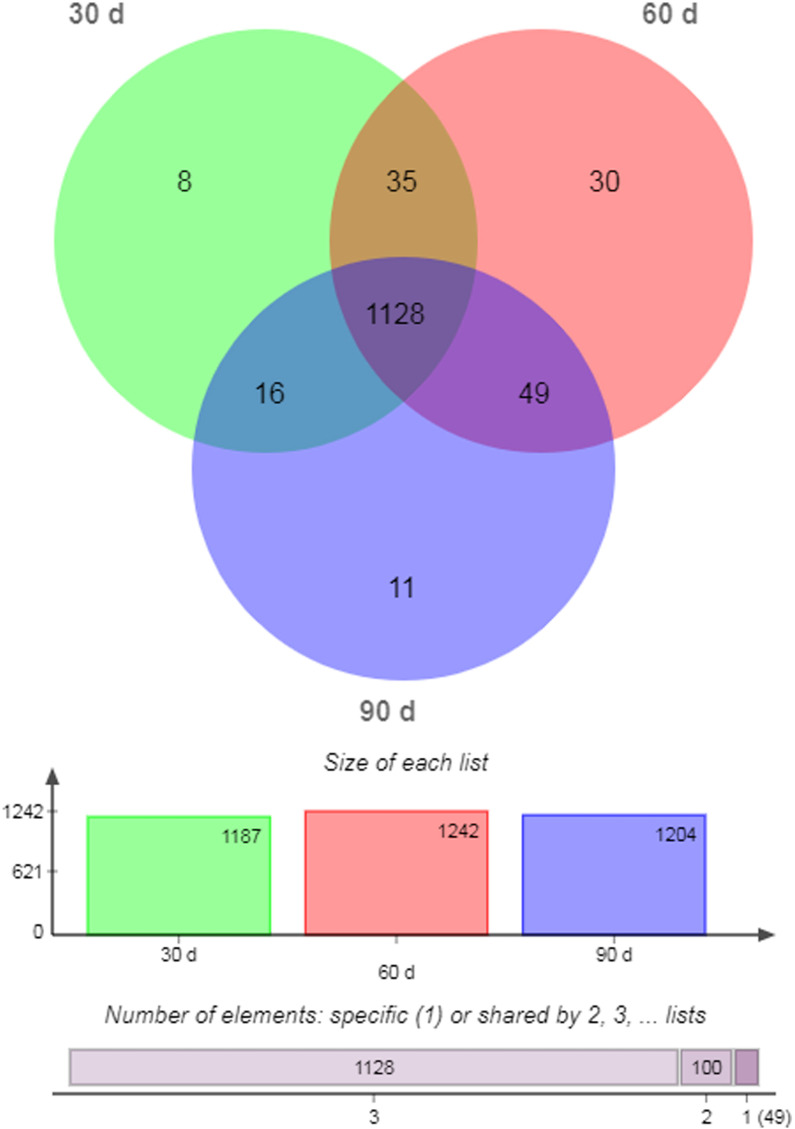
Venn diagram of all miRNAs.

There were 1,128 unique miRNAs co-expressed in 30- (95.03%), 60- (90.82%), and 90-d (93.69%) antler cartilage tissues (Fig. 2). The most abundant miRNA was ggo-miR-148a, then aca-miR-148a-3p, bta-miR-21-5p, chi-miR-21-5p, and sha-miR-21 (Table 3). In addition, eight (0.67%), 30 (2.42%), and 11 (0.91%) miRNAs were specifically expressed (SEMs) in each sample (Fig. 2; Table 5).

**Table 5:** Specifically expressed miRNAs. The specifically expressed miNRAs in antler cartilage of *Cervus elaphus kansuensis*.

30 d			60 d			90 d		
miRNA	Count	TPM	miRNA	Count	TPM	miRNA	Count	TPM
aja-miR-3120	2	0.6102	aga-miR-10	3	0.7211	bta-miR-105a	1	0.2779
bta-miR-3154	1	0.3051	bma-miR-92	1	0.2524	cgr-miR-128-5p	1	0.2779
cgr-miR-23a-5p	1	0.2912	bta-miR-154a	1	0.2294	dre-miR-181b-3p	1	0.2906
cgr-miR-29b-5p	2	0.5339	bta-miR-202	1	0.2404	eca-miR-302d	1	0.2779
eca-miR-421	1	0.3051	bta-miR-2366	1	0.2294	hsa-miR-181b-2-3p	1	0.3196
CM008010.1_21055	6	1.8306	bta-miR-490	3	0.6883	mdo-miR-218-2-3p	1	0.3044
CM008025.1_186565	2	0.5825	bta-miR-544a	1	0.2294	mml-miR-7178-5p	1	0.2906
CM008038.1_307839	8	2.2285	bta-miR-767	1	0.2195	mmu-miR-128-1-5p	1	0.3044
			bta-miR-885	5	1.1472	CM008015.1_78022	10	3.1962
			cfa-miR-377	1	0.2294	CM008020.1_145882	4	1.1117
			cgr-miR-103-5p	1	0.2103	CM008027.1_215433	5	1.4528
			cgr-miR-505-5p	2	0.4389			
			chi-miR-324-3p	1	0.2404			
			chi-miR-33b-3p	1	0.2404			
			chi-miR-411b-3p	9	2.1633			
			chi-miR-491-3p	1	0.2294			
			gga-miR-103-2-5p	1	0.2195			
			hsa-miR-324-3p	1	0.2524			
			hsa-miR-34a-3p	1	0.2294			
			mdo-miR-34a-3p	1	0.2294			
			mml-miR-3059-5p	1	0.2294			
			mmu-miR-324-3p	1	0.2524			
			oan-miR-145-5p	1	0.2657			
			oar-miR-544-3p	1	0.2404			
			ssa-miR-144-3p	2	0.5048			
			CM008012.1_49180	2	0.4807			
			CM008012.1_53957	2	0.4589			
			CM008022.1_167031	2	0.4807			
			CM008025.1_186278	2	0.4807			
			CM008039.1_315920	13	3.1248			

### Differential expression analysis of miRNAs

**Figure 3:**
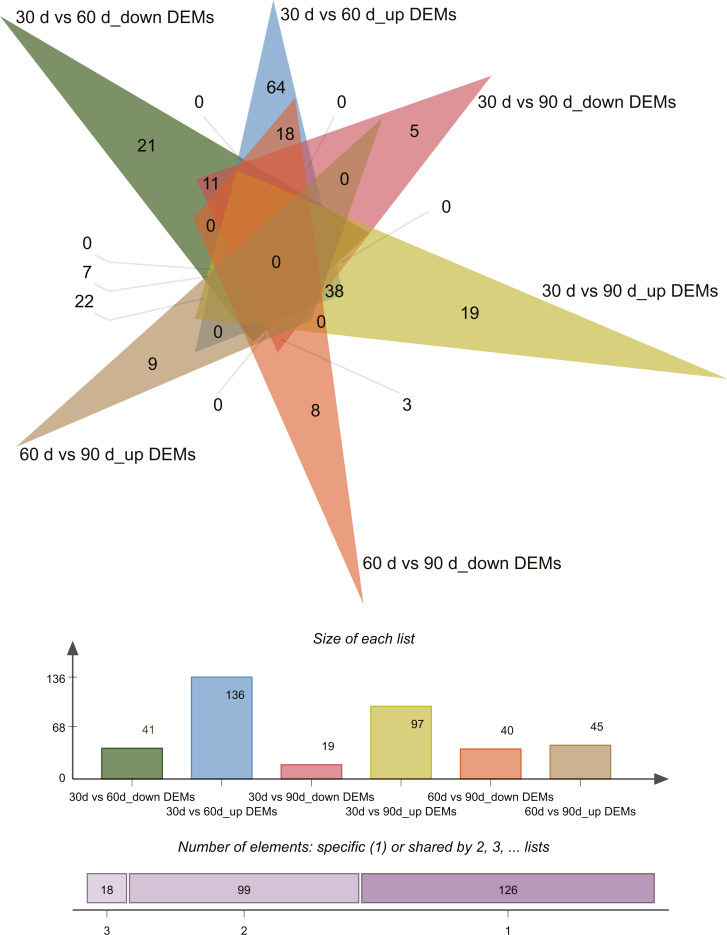
Venn diagram of differentially expressed miRNAs.

Differential expression analysis (| log2 fold change [FC] values | ≥ 1 and *P* ≤ 0.05) revealed 243 differentially expressed miRNAs (DEMs) across the three growth stages in this study (Tables S4–S5). Pairwise comparisons of 30 d vs. 60 d, 30 d vs. 90 d, and 60 d vs. 90 d revealed 177 (136 upregulated and 41 downregulated), 116 (97 upregulated and 19 downregulated), and 85 (45 upregulated and 40 downregulated) DEMs, respectively. Venn diagrams of DEMs in the three pairwise comparisons are presented in Fig. 3.

To identify key miRNAs controlling rapid antler growth, we selected 25 DEMs that had different expression patterns in the 30 d vs. 60 d and 60 d vs. 90 d comparisons only (Table 6). These are the miRNAs that likely play important roles in antler development during RGS at day 60. Among the 25 DEMs, cfa-miR-146b has the highest expression in 60 d, followed by cgr-miR-146b-5p, and bta-miR-146b, bta-miR-27a-5p, and cgr-miR-181a-3p. Two DEMs were specifically expressed in 60 d antler cartilage tissue, namely chi-miR-411b-3p and CM008039.1_315920. We were more interested in the potential biological function of novel miRNA CM008039.1_315920.

### MiRNA target prediction and functional annotation of candidate target genes

MiRNAs usually act *via* translational repression and/or mRNA cleavage, but some evidence shows that they can also upregulate translation through diverse mechanisms. The rapid growth stage is a special period in the growth process of antler velvet; thus, we pay more attention to miRNAs in 60d. In this study, the target genes of 25 DEMs, 30 miRNAs specifically expressed in 60 d and novel miRNA CM008039.1_315920 were predicted. In particular, 726, 1,759 and 139 targets were predicted for 25 selected DEMs, specifically expressed miRNAs in 60 d and CM008039.1_315920, respectively. We then functionally annotated candidate target genes by using Gene Ontology (GO) and Kyoto Encyclopedia of Genes and Genomes (KEGG) analyses.

**Figure 4:**
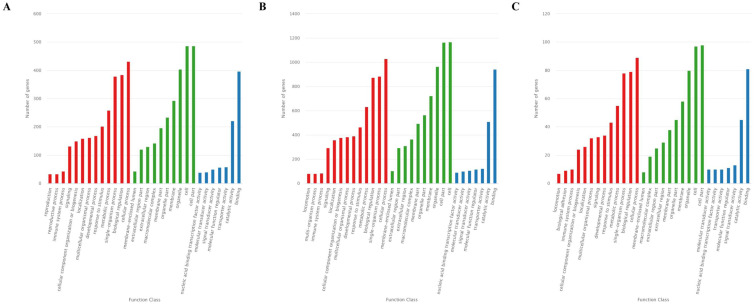
GO classification for target genes of 25 DEMs. For target genes of 25 DEMs, specifically expressed miRNAs in 60 d and CM008039.1_315920, the GO terms of “cellular process,” “cell part,” “cell,” and “binding” were clustered the most target genes. (A) GO classification of target genes for 25 DEMs; (B) GO classification of target genes for specifically expressed miRNAs in 60 d; (C) GO classification of target genes for novel miRNA CM008039.1_315920.

The GO analysis showed that 726 target genes of the 25 DEMs were classified into 52 functional subcategories. “cellular process” (431 genes), “cell part” (487 genes) and “cell” (486 genes), and “binding” (397 genes) were the top enriched GO terms under the biological process (BP), cellular component (CC), and molecular function (MF) categories, respectively (Fig. 4A). For the specifically expressed miRNAs in 60 d, 1,759 target genes were classified into 53 functional subcategories. Similarly, “cellular process” (1,028 genes) “cell” (1,169 genes) and “binding” (943 genes) were the top enriched GO terms under BP, CC, and MF (Fig. 4B). For novel miRNA CM008039.1_315920, 139 target genes were classified into 44 functional subcategories. “cellular process” (89 genes), “cell part” (98 genes) and “binding” (81 genes) were also the top enriched GO terms under BP, CC, and MF (Fig. 4C).

**Figure 5:**
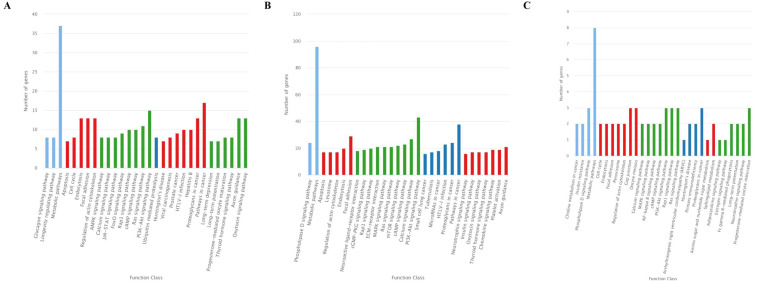
KEGG classification for target genes of 25 DEMs. The ordinate represents the KEGG pathways, and the abscissa represents the number and proportion of target genes annotated to the pathway. For target genes of 25 DEMs, specifically expressed miRNAs in 60 d and CM008039.1_315920, most target genes annotated to pathways of “Metabolic pathways”, “PI3K-Akt signaling pathway” and “Proteoglycans in cancer”. (A) KEGG classification for target genes of all 243 DEMs. (B) KEGG classification for target genes of specifically expressed miRNAs in 60 d; (C) KEGG classification for target genes of CM008039.1_315920.

The KEGG classification showed that target genes of the 25 DEMs were mainly associated with the “Metabolic pathways”, followed by pathways for “Pathways in cancer”, and “PI3K-Akt signaling pathway” (Fig. 5A). Similarly, most target genes of the specifically expressed miRNAs in 60 d were related to the “Metabolic pathways”, followed by pathways for “PI3K-Akt signaling pathway” and “Pathways in cancer” (Fig. 5B). For novel miRNA CM008039.1_315920, most targets were classified to “Metabolic pathways”, as well as pathways of “Gap junction”, “Oocyte meiosis”, “PI3K-Akt signaling pathway”, “Rap1 signaling pathway”, “Ras signaling pathway”, “Proteoglycans in cancer” and “Progesterone-mediated oocyte maturation” (Fig. 5C). KEGG enrichment of miRNA target genes was also analyzed (Fig. S1). Notably, among predicted targets of the 25 DEMs, we discovered that CM008039.1_315920 miRNA was highly enriched in “NF-kappa B signaling pathway” (Fig. S1C), which was mainly associated with apoptosis and cell proliferation.

**Table 6:** Selected 25 DEMs that may participate in antler rapid growth.

	#ID	miRNA_Seq	Length	30 d	60 d	90 d	30 d_vs_60 d	30 d_vs_90 d	60 d_vs_90 d
1	aca-miR-10a-5p	UACCCUGUAGAUCCGAAUUUGUG	23	6.12849554	17.99627	4.724809	Up	Normal	Down
2	aca-miR-144-5p	GGAUAUCAUCAUAUACUGUAA	21	1.83058958	12.97989	5.174791	Up	Normal	Down
3	aca-miR-146a-5p	UGAGAACUGAAUUCCAUAGGC	21	1.52549131	7.932154	1.521997	Up	Normal	Down
4	aga-miR-184	UGGACGGAGAACUGAUAAGGG	21	0.30509826	5.047734	0.608799	Up	Normal	Down
5	bta-miR-146b	UGAGAACUGAAUUCCAUAGGCUGU	24	17.8863856	41.85413	12.78478	Up	Normal	Down
6	bta-miR-187	UCGUGUCUUGUGUUGCAGCCGG	22	1.4561508	5.277177	1.452816	Up	Normal	Down
7	bta-miR-27a-5p	AGGGCUUAGCUGCUUGUGAGCA	22	54.75127	26.15644	68.5729	Down	Normal	Up
8	bta-miR-504	AGACCCUGGUCUGCACUCUGUC	22	0.29123016	3.67108	0.581126	Up	Normal	Down
9	cfa-miR-10a	UACCCUGUAGAUCCGAAUUUGU	22	6.40706351	18.81428	4.939573	Up	Normal	Down
10	cfa-miR-146b	UGAGAACUGAAUUCCAUAGGCU	22	19.5124207	45.65905	13.94703	Up	Normal	Down
11	cgr-miR-146b-5p	UGAGAACUGAAUUCCAUAGGCUG	23	18.6640546	43.67388	13.34064	Up	Normal	Down
12	cgr-miR-181a-3p	ACCAUCGACCGUUGAUUGUACC	22	41.0634525	20.19094	42.13165	Down	Normal	Up
13	cgr-miR-187	UCGUGUCUUGUGUUGCAGCCG	21	1.52549131	5.528471	1.521997	Up	Normal	Down
14	chi-miR-187	UCGUGUCUUGUGUUGCAGCC	20	1.60176588	5.804894	1.598097	Up	Normal	Down
15	chi-miR-411b-3p	UAUGUCACAUGGUCCACUAAU	21	0	2.163315	0	Up	–	Down
16	chi-miR-504	AGACCCUGGUCUGCACUCUGU	21	0.30509826	3.845893	0.608799	Up	Normal	Down
17	efu-miR-181a	AACCAUCGACCGUUGAUUGUACC	23	39.278085	19.31307	40.29984	Down	Normal	Up
18	mdo-miR-181a-1-3p	CCAUCGACCGUUGAUUGUACC	21	42.7137568	20.67167	43.22472	Down	Normal	Up
19	oar-miR-10a	UACCCUGUAGAUCCGAAUUUG	21	7.01726004	19.95057	5.174791	Up	Normal	Down
20	sha-miR-181a-3p	ACCAUCGACCGUUGAUUGU	19	47.5471556	23.37898	48.78402	Down	Normal	Up
21	ssa-miR-144-5p	GGAUAUCAUCAUAUACUGUAAGUU	24	1.60176588	11.3574	4.527942	Up	Normal	Down
22	CM008019.1_137729	CCCAGGGAUGUAGCUCCUAGUGC	23	1.39283989	4.389334	1.11172	Up	Normal	Down
23	CM008022.1_159134	CAAAUUCGUGAAGCGUUCCAUAUUU	25	18.1960604	6.259191	14.57465	Down	Normal	Up
24	CM008025.1_184228	CAAAUUCGUGAAGCGUUCCAUAUUU	25	18.1960604	6.259191	14.83034	Down	Normal	Up
25	CM008039.1_315920	CGGAUCAGCUCAGUGCCGGGC	21	0	3.124788	0	Up	–	Down

### Quantitative real-time PCR (qRT-PCR) verification

**Figure 6:**
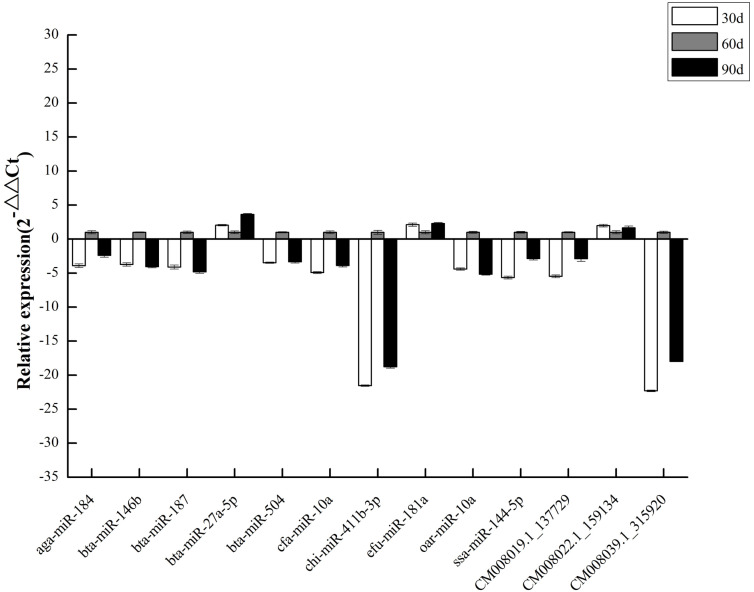
qPCR of DEMs.

To validate DEM expression profiles, we randomly selected 13 miRNAs, including 10 known and three novel miRNAs, for stem-loop qRT-PCR. We discovered that qRT-PCR results and high-throughput sequencing data were correlated (Fig. 6), validating DEM expression patterns and confirming the reliability and accuracy of our miRNA-seq data.

## Discussion

The velvet antler is capable of regeneration and rapid growth, processes that are inseparable from the self-renewal and differentiation ability of mesenchymal tissue in the antler growth center. These characteristics differentiate the antler from normal cartilage or bone tissue (Li et al., 2014). The growth center (mainly comprising velvet skin, mesenchyme, and cartilage) controls velvet antler regeneration and development (Li et al., 2002; Ba et al., 2019). Antler growth centers are rich in regulatory factors, including growth and transcription factors related to cartilage growth and ossification (Yao et al., 2018). Multiple studies have reported that let-7a and let-7f (Hu et al., 2014a), miR-18a (Hu et al., 2014b), miR-19a/b (Yan et al., 2020), and miR-15a/b (Liu et al., 2018) participate in the regulation of antler chondrocyte proliferation and regeneration. Therefore, discovering novel regulators and their associated pathways will help us understand the mechanisms involved in velvet antler RGS.

Here, we investigated and compared miRNA profiles of antler cartilage tissues at three growth stages (30, 60, and 90 d), revealing several DEMs and candidate target genes that are associated with the rapid growth of velvet antlers.

This study predicted 1,277 miRNAs, including 1,073 known and 204 novel miRNAs. Among these, 243 were differentially expressed across the three growth stages. We screened 25 DEMs from the 30 d vs. 60 d and 60 d vs 90 d comparisons, including 21 known and four novel miRNAs; 18 of these were upregulated and seven were downregulated in 60 d antler cartilage tissue. MicroRNA (miRNA) is evolutionarily conserved non-coding small RNA molecule between species. Since no miRNA sequence data of *C. elaphus kansuensis* was available in the miRBase v21.0 database, the miRNAs in this study were extrapolated from other species. This method was also used in other studies (Ba, Wang & Li, 2016).

Two DEMs (CM008039.1_315920 and chi-miR-411b-3p) with the lowest expression levels among 25 DEMs were specifically expressed in 60 d antler cartilage. Through suppressing target gene expression, miRNAs are known to alter expression patterns in specific tissues at specific times (Mohr & Mott, 2015). However, no studies have investigated the role of mir-411b in chondrogenesis. Thus, further research on these two miRNAs is required to determine their biological functions during the RGS of antler development.

Bioinformatics analysis revealed that the predicted target genes of 25 DEMs and specifically expressed miRNAs in 60d were widely classified into multiple GO terms, indicating that these genes may participate in many important biological processes. The GO enrichment results showed that most target genes were highly enriched in the “cellular process”, “cell”, “cell part”, and “binding” terms, implying involvement in antler development. The velvet antler may grow at a speed of two cm/d during RGS, a period of rapid cell proliferation, differentiation, and aggregation. The amount of ATP, nucleotides, proteins, and other factors involved in velvet antler growth may be able to support vigorous cell activity of antler, such as proliferation and differentiation.

Unlike the GO analysis, the KEGG pathway analysis yielded more direct and detailed results. The pathways represent protein-protein interactions and any change in each pathway reflects changes in a specific protein’s expression or activity. KEGG pathway analysis revealed that most target genes of 25 DEMs, specifically expressed miRNAs in 60d and CM008039.1_315920 were mainly associated with “Metabolic pathways”, “PI3K-Akt signaling pathway” and “Proteoglycans in cancer”. Metabolic pathways have extensive contents, such as glycolysis, fatty acid oxidation, and amino acid metabolism (Boroughs & DeBerardinis, 2015). These pathways play important roles in cells fate and functions (Boroughs & DeBerardinis, 2015; Kim & DeBerardinis, 2019) and determine vasculature formation (Li, Kumar & Carmeliet, 2019). Antler cartilage is rich in nerves and blood vessels, and there is a definite link between the abundant blood vessels and the rapid growth and regeneration of antlers (Clark et al., 2006; Hu et al., 2019). In summer, velvet antler grows rapidly, and a large number of blood vessels in antler can transport nutrients to and excrete metabolites from the antler. It has been reported that there may be a close relationship between metabolites and the size of velvet antlers (Su et al., 2020). Thus, metabolic pathways play important roles in rapid growth and regeneration of velvet antler.

PI3K-Akt pathway can regulate multiple cellular events including cell apoptosis, proliferation and differentiation. Moreover, PI3K-Akt pathway is frequently involved in cancers (Jiang et al., 2020). It can be activated by Wnt signaling pathway and there is complex crosstalk between PI3K-Akt and Wnt/*β*-catenin pathways (Dong et al., 2020). These two signaling pathways can function synergistically (Evangelisti et al., 2020; Prossomariti et al., 2020). PI3K-Akt pathway also participates in glucose and glutamine metabolism within the hierarchy of pathways altered in cancer (Boroughs & DeBerardinis, 2015).

Proteoglycans are a group of molecules, which play significant roles in cancer development, progression, invasion and metastasis (Wei et al., 2020). Whether the pathway of “Proteoglycans in cancer” participates in antler rapid growth and regeneration needs further investigation.

Target genes associated with the above signaling pathways may also be involved with the well-known Wnt signaling pathway, widely distributed in invertebrates and vertebrates. This pathway mediates important cellular responses, including cancer development, body axis development, and morphogenesis. Wnt signaling also plays a crucial role in the early development of animal embryos, organ formation, tissue regeneration, and other normal physiological processes (Leucht, Lee & Yim, 2019). Previous studies have reported that the Wnt signaling pathway participates in the regulation of antler growth and development (Mount et al., 2006; Li et al., 2012; Zhang et al., 2018). Some researchers believe that antler growth is a tumor-like development due to their similar growth rates (Goss, 1990; Kierdorf & Kierdorf, 2011; Wang et al., 2017), suggesting a possible mechanism connecting the miRNAs detected in this study and antler development. Indeed, numerous genes are involved in both cancer and antler development. However, further studies are required to identify the specific genes acting on antler growth and development during RGS.

KEGG pathway analysis also revealed that CM008039.1_315920 miRNA was abundant in the NF- *κ*B pathway. NF- *κ*B is a ubiquitous transcription factor that regulates the expression of genes involved in multiple cell functions; it is activated by various extracellular stimuli (Caviedes et al., 2017). NF- *κ*B plays an important role during immune response and inflammation. However, growing evidence suggests that NF- *κ*B also has a major role in oncogenesis through regulating the expression of genes involved in cancer development and progression, such as tumor cell proliferation, migration, and apoptosis (Dolcet et al., 2005). Therefore, further investigation is required to confirm the function of this novel miRNA in velvet antler development.

We screened 25 DEMs that are potentially important for antler development from the 30 d vs. 60 d and 60 d vs. 90 d comparisons. Through qRT-PCR, we confirmed that the expression levels of 13 randomly selected miRNAs were consistent with the bioinformatics analysis.

Taken together, our findings provide new insights into the possible mechanisms involved in antler development during the RGS. Further research on the 25 DEMs and specifically expressed miRNAs in 60 d identified here, especially the novel miRNA CM008039.1_315920, will be helpful in elucidating mechanisms underlying the rapid growth of deer antlers and may also contribute novel therapeutic targets for cancer treatment.

## Conclusions

In this study, the novel miRNA CM008039.1_315920 was differentially expressed in the 30 d vs. 60 d and 60 d vs. 90 d comparisons, but not in the 30 d vs. 90 d comparison. This miRNA was also a specifically expressed miRNA in deer antler cartilage that grew for about 60 d. Moreover, the target genes of this novel miRNA were annotated to NF- *κ*B pathway, indicating that CM008039.1_315920 may possess important biological functions in rapid antler growth.

##  Supplemental Information

10.7717/peerj.13947/supp-1 Supplemental Information 1 Author Checklist - Full

10.7717/peerj.13947/supp-2 Table S1 MiRNAs length distribution

10.7717/peerj.13947/supp-3 Table S2 MiRNA family clusters

10.7717/peerj.13947/supp-4 Table S3 All miRNAs

10.7717/peerj.13947/supp-5 Table S4 Log2fold changes of all miRNAs

10.7717/peerj.13947/supp-6 Table S5 All 243 DEMs

10.7717/peerj.13947/supp-7 Data S1 Raw data for qRT-PCR

10.7717/peerj.13947/supp-8 Figure S1A KEGG enrichment for target genes of 25 DEMs

10.7717/peerj.13947/supp-9 Figure S1B KEGG enrichment for target genes of SEMs in 60 d

10.7717/peerj.13947/supp-10 Figure S1C KEGG enrichment for target genes of selected novel miRNAs

## Additional Information and Declarations

### Data Availability

The following information was supplied regarding data availability: The datasets are available in NCBI SRA BioProject: PRJNA731682.
https://www.ncbi.nlm.nih.gov/bioproject/PRJNA731682.

## References

[ref-1] Ackerman 4th WE, Buhimschi IA, Brubaker D, Maxwell S, Rood KM, Chance MR, Jing H, Mesiano S, Buhimschi CS (2018). Integrated microRNA and mRNA network analysis of the human myometrial transcriptome in the transition from quiescence to labor. Biology of Reproduction.

[ref-2] Ashburner M, Ball CA, Blake JA, Botstein D, Butler H, Cherry JM, Davis AP, Dolinski K, Dwight SS, Eppig JT, Harris MA, Hill DP, Issel-Tarver L, Kasarskis A, Lewis S, Matese JC, Richardson JE, Ringwald M, Rubin GM, Sherlock G (2000). Gene ontology: tool for the unification of biology. Nature Genetics.

[ref-3] Ba H, Wang D, Li C (2016). MicroRNA profiling of antler stem cells in potentiated and dormant states and their potential roles in antler regeneration. Molecular Genetics and Genomics.

[ref-4] Ba H, Wang D, Yau TO, Shang Y, Li C (2019). Transcriptomic analysis of different tissue layers in antler growth center in cika deer (*Cervus nippon*). BMC Genomics.

[ref-5] Bao W, Kojima KK, Kohany O (2015). Repbase Update, a database of repetitive elements in eukaryotic genomes. Mobile DNA.

[ref-6] Bonnet E, Wuyts J, Rouzé P, Van de Peer Y (2004). Evidence that microRNA precursors, unlike other non-coding RNAs, have lower folding free energies than random sequences. Bioinformatics.

[ref-7] Boroughs LK, DeBerardinis RJ (2015). Metabolic pathways promoting cancer cell survival and growth. Nature Cell Biology.

[ref-8] Candar-Cakir B, Arican E, Zhang B (2016). Small RNA and degradome deep sequencing reveals drought-and tissue-specific micrornas and their important roles in drought-sensitive and drought-tolerant tomato genotypes. Plant Biotechnology Journal.

[ref-9] Caviedes A, Lafourcade C, Soto C, Wyneken U (2017). Bdnf/nf-kb signaling in the neurobiology of depression. Current Pharmaceutical Design.

[ref-10] Chan PP, Lowe TM (2016). GtRNAdb 2.0: an expanded database of transfer RNA genes identified in complete and draft genomes. Nucleic Acids Research.

[ref-11] Chen Y, Zhang Z, Jin W, Li Z, Bao C, He C, Guo Y, Li C (2022). Integrative analyses of antler cartilage transcriptome and proteome of Gansu red deer (*Cervus elaphus kansuensis*) at different growth stages. Animals.

[ref-12] Chiba T, Kurimoto R, Matsushima T, Ito Y, Nakamichi R, Lotz M, Asahara H (2021). MicroRNA expression profiling, target identification, and validation in chondrocytes. Methods in Molecular Biology.

[ref-13] Chu W, Zhao H, Li J, Li C (2017). Custom-built tools for the study of deer antler biology. Frontiers in Bioscience.

[ref-14] Clark DE, Li C, Wang W, Martin SK, Suttie JM (2006). Vascular localization and proliferation in the growing tip of the deer antler. The Anatomical Record. Part A.

[ref-15] Dolcet X, Llobet D, Pallares J, Matias-Guiu X (2005). NF-kB in development and progression of human cancer. Virchows Archiv.

[ref-16] Dong J, Xu X, Zhang Q, Yuan Z, Tan B (2020). The PI3K/AKT pathway promotes fracture healing through its crosstalk with Wnt/*β*-catenin. Experimental Cell Research.

[ref-17] Evangelisti C, Chiarini F, Cappellini A, Paganelli F, Fini M, Santi S, Martelli AM, Neri LM, Evangelisti C (2020). Targeting Wnt/*β*-catenin and PI3K/Akt/mTOR pathways in T-cell acute lymphoblastic leukemia. Journal of Cellular Physiology.

[ref-18] Fahlgren N, Howell MD, Kasschau KD, Chapman EJ, Sullivan CM, Cumbie JS, Givan SA, Law TF, Grant SR, Dangl JL, Carrington JC (2007). High-throughput sequencing of Arabidopsis microRNAs: evidence for frequent birth and death of MIRNA genes. PLOS ONE.

[ref-19] Friedländer MR, Mackowiak SD, Li N, Chen W, Rajewsky N (2012). miRDeep2 accurately identifies known and hundreds of novel microRNA genes in seven animal clades. Nucleic Acids Research.

[ref-20] Goss RJ (1990). Tumor-like growth of antlers in castrated fallow deer: an electron microscopic study. Scanning Microscopy.

[ref-21] Hao ZY, Wang JQ, Luo YL, Liu X, Li SB, Zhao ML, Jin XY, Shen JY, Ke N, Song YZ, Qiao LR (2021). Deep small RNA-Seq reveals microRNAs expression profiles in lactating mammary gland of 2 sheep breeds with different milk performance. Domestic Animal Endocrinology.

[ref-22] Hou J, Zhao L, Yan J, Ren X, Zhu K, Gao T, Du X, Luo H, Li Z, Xu M (2019). MicroRNA expression profile is altered in the upper airway skeletal muscle tissue of patients with obstructive sleep apnea-hypopnea syndrome. The Journal of International Medical Research.

[ref-23] Hu P, Wang T, Liu H, Xu J, Wang L, Zhao P, Xing X (2019). Full-length transcriptome and microRNA sequencing reveal the specific gene-regulation network of velvet antler in sika deer with extremely different velvet antler weight. Molecular Genetics and Genomics.

[ref-24] Hu W, Li T, Hu R, Wu L, Li M, Meng X (2014a). MicroRNA let-7a and let-7f as novel regulatory factors of the sika deer (*Cervus nippon*) IGF-1R gene. Growth Factors.

[ref-25] Hu W, Li T, Wu L, Li M, Meng X (2014b). Identification of microRNA-18a as a novel regulator of the insulin-like growth factor-1 in the proliferation and regeneration of deer antler. Biotechnology Letters.

[ref-26] Jiang N, Dai Q, Su X, Fu J, Feng X, Peng J (2020). Role of PI3K/AKT pathway in cancer: the framework of malignant behavior. Molecular Biology Reports.

[ref-27] Kalvari I, Argasinska J, Quinones-Olvera N, Nawrocki EP, Rivas E, Eddy SR, Bateman A, Finn RD, Petrov AI (2018). Rfam 13.0: shifting to a genome-centric resource for non-coding RNA families. Nucleic Acids Research.

[ref-28] Kanehisa M, Goto S (2000). KEGG: Kyoto Encyclopedia of Genes and Genomes. Nucleic Acids Research.

[ref-29] Kierdorf U, Kierdorf H (2011). Deer antlers—a model of mammalian appendage regeneration: an extensive review. Gerontology.

[ref-30] Kim J, DeBerardinis RJ (2019). Mechanisms and implications of metabolic heterogeneity in cancer. Cell Metabolism.

[ref-31] Kozomara A, Griffiths-Jones S (2014). miRBase: annotating high confidence microRNAs using deep sequencing data. Nucleic Acids Research.

[ref-32] Langmead B, Trapnell C, Pop M, Salzberg SL (2009). Ultrafast and memory-efficient alignment of short DNA sequences to the human genome. Genome Biology.

[ref-33] Leucht P, Lee S, Yim N (2019). Wnt signaling and bone regeneration: can’t have one without the other. Biomaterials.

[ref-34] Li Y, Alonso-Peral M, Wong G, Wang MB, Millar AA (2016). Ubiquitous miR159 repression of MYB33/65 in Arabidopsis rosettes is robust and is not perturbed by a wide range of stresses. BMC Plant Biology.

[ref-35] Li C, Clark DE, Lord EA, Stanton JA, Suttie JM (2002). Sampling technique to discriminate the different tissue layers of growing antler tips for gene discovery. The Anatomical Record.

[ref-36] Li C, Harper A, Puddick J, Wang W, McMahon C (2012). Proteomes and signalling pathways of antler stem cells. PLOS ONE.

[ref-37] Li X, Kumar A, Carmeliet P (2019). Metabolic pathways fueling the endothelial cell drive. Annual Review of Physiology.

[ref-38] Li C, Zhao H, Liu Z, McMahon C (2014). Deer antler—a novel model for studying organ regeneration in mammals. The International Journal of Biochemistry & Cell Biology.

[ref-39] Liu F, Liu X, Yang Y, Sun Z, Deng S, Jiang Z, Li W, Wu F (2020). NEAT1/miR-193a-3p/SOX5 axis regulates cartilage matrix degradation in human osteoarthritis. Cell Biology International.

[ref-40] Liu M, Han X, Cui D, Yan Y, Li L, Wei H (2018). Post-transcriptional regulation of mirna-15a and mirna-15b on vegfr gene and deer antler cell proliferation. Turkish Journal of Biochemistry.

[ref-41] Livak KJ, Schmittgen TD (2001). Analysis of relative gene expression data using real-time quantitative PCR and the 2(-Delta Delta C(T)) Method. Methods.

[ref-42] Lu TX, Rothenberg ME (2018). MicroRNA. The Journal of Allergy and Clinical Immunology.

[ref-43] Mohr AM, Mott JL (2015). Overview of microRNA biology. Seminars in Liver Disease.

[ref-44] Mount JG, Muzylak M, Allen S, Althnaian T, McGonnell IM, Price JS (2006). Evidence that the canonical Wnt signalling pathway regulates deer antler regeneration. Developmental Dynamics.

[ref-45] National Academies Council (2011). 2011 Guide for the care and use of laboratory animals.

[ref-46] Price J, Allen S (2004). Exploring the mechanisms regulating regeneration of deer antlers. Philosophical Transactions of the Royal Society of London. Series B.

[ref-47] Price J, Faucheux C, Allen S (2005). Deer antlers as a model of Mammalian regeneration. Current Topics in Developmental Biology.

[ref-48] Prossomariti A, Piazzi G, Alquati C, Ricciardiello L (2020). Are Wnt/*β*-Catenin and PI3K/AKT/mTORC1 distinct pathways in colorectal cancer?. Cellular and Molecular Gastroenterology and Hepatology.

[ref-49] Quast C, Pruesse E, Yilmaz P, Gerken J, Schweer T, Yarza P, Peplies J, Glöckner FO (2013). The SILVA ribosomal RNA gene database project: improved data processing and web-based tools. Nucleic Acids Research.

[ref-50] Rao X, Huang X, Zhou Z, Lin X (2013). An improvement of the 2}{}$\hat {}$ (−delta delta CT) method for quantitative real-time polymerase chain reaction data analysis. Biostatistics, Bioinformatics and Biomathematics.

[ref-51] Romualdi C, Bortoluzzi S, D’Alessi F, Danieli GA (2003). IDEG6: a web tool for detection of differentially expressed genes in multiple tag sampling experiments. Physiological Genomics.

[ref-52] Percie du Sert N, Ahluwalia A, Alam S, Avey MT, Baker M, Browne WJ, Clark A, Cuthill IC, Dirnagl U, Emerson M, Garner P, Holgate ST, Howells DW, Hurst V, Karp NA, Lazic SE, Lidster K, MacCallum CJ, Macleod M, Pearl EJ, Petersen OH, Rawle F, Reynolds P, Rooney K, Sena ES, Silberberg SD, Steckler T, Würbel H (2020). Reporting animal research: explanation and elaboration for the ARRIVE guidelines 2.0. PLOS Biology.

[ref-53] Su H, Yang C, Jin C, Zhang H, Yin C, Yang Y, Chen H, Jing L, Qi B, Zhao D, Bai X, Liu L (2020). Comparative metabolomics study revealed difference in central carbon metabolism between sika deer and red deer antler. International Journal of Genomics.

[ref-54] Tu Y, Ma T, Wen T, Yang T, Xue L, Cai M, Wang F, Guan M, Xue H (2020). MicroRNA-377-3p alleviates IL-1 *β*-caused chondrocyte apoptosis and cartilage degradation in osteoarthritis in part by downregulating ITGA6. Biochemical and Biophysical Research Communications.

[ref-55] Wang DT, Chu WH, Sun HM, Ba HX, Li CY (2017). Expression and functional analysis of tumor-related factor S100A4 in antler stem cells. The Journal of Histochemistry and Cytochemistry.

[ref-56] Wang C, Li F, Deng L, Li M, Wei M, Zeng B, Wu K, Xu Z, Wei R, Wei L, Liu W, Zhang S, Xu L, Huang Y, Li D, Li Y, Zhang H (2021). Identification and characterization of miRNA expression profiles across five tissues in giant panda. Gene.

[ref-57] Wei J, Hu M, Huang K, Lin S, Du H (2020). Roles of proteoglycans and glycosaminoglycans in cancer development and progression. International Journal of Molecular Sciences.

[ref-58] Wójcik AM, Gaj MD (2016). miR393 contributes to the embryogenic transition induced in vitro in Arabidopsis via the modification of the tissue sensitivity to auxin treatment. Planta.

[ref-59] Yan Y, Chen D, Han X, Liu M, Hu W (2020). MiRNA-19a and miRNA-19b regulate proliferation of antler cells by targeting TGFBR2. Mammal Research.

[ref-60] Yang YH, Li MJ, Yi YJ, Li RF, Li CX, Yang H, Wang J, Zhou JX, Shang S, Zhang ZY (2021). Integrated miRNA-mRNA analysis reveals the roles of miRNAs in the replanting benefit of Achyranthes bidentata roots. Scientific Reports.

[ref-61] Yao B, Zhang M, Liu M, Wang Q, Liu M, Zhao Y (2018). Sox9 Functions as a master regulator of antler growth by controlling multiple cell lineages. DNA and Cell Biology.

[ref-62] Zhang H, Hu J, Qian Q, Chen H, Jin J, Ding Y (2016a). Small RNA profiles of the rice PTGMS Line Wuxiang S reveal miRNAs involved in fertility transition. Frontiers in Plant Science.

[ref-63] Zhang H, Wang Y, Yang G, Yu H, Zhou Z, Tang M (2019). MicroRNA-30a regulates chondrogenic differentiation of human bone marrow-derived mesenchymal stem cells through targeting Sox9. Experimental and Therapeutic Medicine.

[ref-64] Zhang W, Xie Y, Xu L, Wang Y, Zhu X, Wang R, Zhang Y, Muleke EM, Liu L (2016b). Identification of microRNAs and their target genes explores miRNA-mediated regulatory network of cytoplasmic male sterility occurrence during anther development in radish (*Raphanus sativus L.*). Frontiers in Plant Science.

[ref-65] Zhang HL, Yang ZQ, Duan CC, Geng S, Wang K, Yu HF, Yue ZP, Guo B (2018). WNT4 acts downstream of BMP2 to mediate the regulation of ATRA signaling on RUNX1 expression: implications for terminal differentiation of antler chondrocytes. Journal of Cellular Physiology.

[ref-66] Zhao S, Ye Z, Stanton R (2020). Misuse of RPKM or TPM normalization when comparing across samples and sequencing protocols. RNA.

