# Supplementary material for: RNA sequencing-based identification of microRNAs in the antler cartilage of Gansu red deer (Cervus elaphus kansuensis)
Source: PeerJ. 2022 Sep 21;10:e13947. doi: 10.7717/peerj.13947 (PMC9508884; doi:10.7717/peerj.13947)
Supplement: Table S1 [file peerj-10-13947-s002.docx]

Additional file Table S1 miRNAs length distribution

| miRNA Length (nt) | miRNA number (N) | | |
| --- | --- | --- | --- |
|  | **30 d** | **60 d** | **90 d** |
| 15 | 0 | 0 | 0 |
| 16 | 0 | 0 | 0 |
| 17 | 0 | 0 | 0 |
| 18 | 17 | 21 | 20 |
| 19 | 36 | 38 | 37 |
| 20 | 92 | 97 | 95 |
| 21 | 278 | 288 | 274 |
| 22 | 462 | 488 | 476 |
| 23 | 202 | 206 | 202 |
| 24 | 66 | 69 | 66 |
| 25 | 30 | 31 | 30 |
| 26 | 3 | 3 | 3 |
| 27 | 1 | 1 | 1 |
| 28 | 0 | 0 | 0 |
| 29 | 0 | 0 | 0 |
| 30 | 0 | 0 | 0 |
| 31 | 0 | 0 | 0 |
| 32 | 0 | 0 | 0 |
| 33 | 0 | 0 | 0 |
| 34 | 0 | 0 | 0 |
| 35 | 0 | 0 | 0 |
