# Supplementary material for: RNA sequencing-based identification of microRNAs in the antler cartilage of Gansu red deer (Cervus elaphus kansuensis)
Source: PeerJ. 2022 Sep 21;10:e13947. doi: 10.7717/peerj.13947 (PMC9508884; doi:10.7717/peerj.13947)
Supplement: Table S2 [file peerj-10-13947-s003.docx]

**Supplementary Table S2 miRNA family clusters**

| Index | miRNA family | miRNA |
| --- | --- | --- |
| 1 | let-7 | cgr-let-7g-3p; efu-let-7d; gga-let-7g-5p; aca-miR-98-5p; aja-let-7i; cgr-let-7i; bta-let-7d; aca-let-7d-5p; aca-let-7f-1-3p; ggo-let-7c; aca-let-7c-5p; aca-let-7a-5p; rno-miR-98-3p; chi-miR-98-3p; bta-let-7a-3p; ssc-let-7i; mse-let-7a; prd-let-7-5p; chi-let-7f-3p; aca-let-7a-3p; efu-let-7e; ccr-let-7i; chi-let-7a-3p; age-miR-98; cfa-let-7e ;ame-let-7; ggo-let-7f; oar-let-7d; aca-let-7i-3p; bta-let-7e; aca-let-7f-5p; aca-let-7f-2-3p; aca-let-7c-1-3p; cgr-let-7d-3p; aca-let-7c-2-3p; chi-let-7e-3p; hsa-let-7f-2-3p; aca-let-7g; aca-let-7i-5p; chi-let-7d-3p |
| 2 | mir-1 | crm-miR-1-3p; aca-miR-1a-3p; bta-miR-206; asu-miR-1-3p |
| 3 | mir-10 | oar-miR-10b; eca-miR-125a-3p; cgr-miR-125b-3p; oar-miR-10a; gga-miR-125b-3p; oha-miR-10c-5p; aae-miR-125-5p; chi-miR-125b-5p; mmu-miR-100-3p; chi-miR-99b-3p; bta-miR-125a; dvi-miR-125-5p; cfa-miR-125a; ccr-miR-100; efu-miR-125a; aca-miR-10b-3p; hsa-miR-99b-3p; oar-miR-99a; mmu-miR-10b-3p; chi-miR-99a-3p; mdo-miR-125b-2-3p; bta-miR-10b; rno-miR-10b-3p; cgr-miR-10b-3p; cgr-miR-100-3p; chi-miR-125b-3p; gga-miR-10b-3p; ola-miR-100; ggo-miR-125a; chi-miR-100-3p; bta-miR-99a-5p; cgr-miR-99a-3p; aca-miR-10b-5p; aga-miR-10; aca-miR-99a-5p; cgr-miR-125a-3p; cgr-miR-125a-5p; aca-miR-10a-5p; bta-miR-99b; efu-miR-125b; mml-miR-99b-3p; sha-miR-10b; aae-miR-100; gga-miR-99a-3p; cfa-miR-10a; aca-miR-99a-3p |
| 4 | mir-101 | bta-miR-101; age-miR-101; aca-miR-101-3p; ola-miR-101a-3p; gga-miR-101-3p |
| 5 | mir-103 | efu-miR-107; cgr-miR-103-5p; ipu-miR-107b; dre-miR-107a-3p; aca-miR-107-3p; gga-miR-103-2-5p; efu-miR-103b; aca-miR-103-3p; age-miR-103; cfa-miR-107 |
| 6 | mir-105 | bta-miR-105a |
| 7 | mir-1193 | mmu-miR-1193-5p |
| 8 | mir-1246 | bta-miR-1246 |
| 9 | mir-1249 | CM008029.1_236567 |
| 10 | mir-126 | efu-miR-126; bta-miR-126-3p; aca-miR-126-5p; chi-miR-126-3p; sha-miR-126; gga-miR-126-3p; ccr-miR-126-3p; aca-miR-126-3p; tgu-miR-126-5p; ola-miR-126-3p; chi-miR-126-5p |
| 11 | mir-1260a | CM008018.1_117444; hsa-miR-1260a |
| 12 | mir-1260b | bta-miR-1260b |
| 13 | mir-127 | chi-miR-127-3p; oar-miR-127; mml-miR-127-5p; rno-miR-127-5p; age-miR-127; ggo-miR-127; hsa-miR-127-5p; chi-miR-127-5p |
| 14 | mir-1271 | chi-miR-1271-5p; bta-miR-1271 |
| 15 | mir-1277 | CM008024.1_180123 |
| 16 | mir-128 | efu-miR-128a; aca-miR-128-3p; efu-miR-128b; mmu-miR-128-1-5p; cgr-miR-128-5p; ggo-miR-128; age-miR-128 |
| 17 | mir-129 | aca-miR-129b-3p; bta-miR-129; bta-miR-129-3p; aca-miR-129a-5p |
| 18 | mir-1296 | mml-miR-1296-5p; bta-miR-1296; cfa-miR-1296 |
| 19 | mir-1298 | mmu-miR-1298-3p; CM008029.1_236542; bta-miR-1298; ggo-miR-1298 |
| 20 | mir-130 | bta-miR-301a; bta-miR-130b; hsa-miR-130b-5p; aca-miR-301a-3p; cgr-miR-301a-5p; cfa-miR-301b; dre-miR-301c-5p; hhi-miR-301; cgr-miR-130b-5p; chi-miR-301a-5p; ggo-miR-130b |
| 21 | mir-1304 | MKHE01008352.1_338794 |
| 22 | mir-1306 | bta-miR-1306; aca-miR-1306; cgr-miR-1306-3p; chi-miR-1306-3p; gga-miR-1306-3p; rno-miR-1306-3p; dre-miR-1306 |
| 23 | mir-1307 | bta-miR-1307; hsa-miR-1307-5p; eca-miR-1307; chi-miR-1307-3p |
| 24 | mir-132 | mmu-miR-132-5p; mmu-miR-212-3p; oha-miR-212-5p; cfa-miR-132; bta-miR-212; cgr-miR-132-5p; CM008012.1_46537; bta-miR-132; aca-miR-212-5p |
| 25 | mir-133 | chi-miR-133a-3p; aae-miR-133; efu-miR-133-3p; aca-miR-133a; cfa-miR-133c; ccr-miR-133a-5p; chi-miR-133a-5p; bfl-miR-133; efu-miR-133-5p |
| 26 | mir-134 | cfa-miR-134; CM008020.1_143049; ggo-miR-134 |
| 27 | mir-1343 | cfa-miR-1343; chi-miR-1343; bta-miR-1343-3p |
| 28 | mir-135 | mdo-miR-135b-5p; aca-miR-135-5p; bta-miR-135b |
| 29 | mir-136 | hsa-miR-136-3p; chi-miR-136-5p; chi-miR-136-3p; mmu-miR-136-3p |
| 30 | mir-138 | bta-miR-138; tgu-miR-138-1-3p; aca-miR-138-5p |
| 31 | mir-1388 | bta-miR-1388-5p; dre-miR-1388-5p |
| 32 | mir-139 | eca-miR-139-3p; cgr-miR-139-3p; mmu-miR-139-3p; lla-miR-139; cgr-miR-139-5p; bta-miR-139 |
| 33 | mir-140 | aca-miR-140-3p; bta-miR-140; gga-miR-140-5p; dre-miR-140-3p; ccr-miR-140-5p; cfa-miR-140; aca-miR-140-5p; cgr-miR-140-3p; gga-miR-140-3p; ola-miR-140-3p; tch-miR-140-5p |
| 34 | mir-143 | oan-miR-143-5p; aca-miR-143-5p; aja-miR-143; efu-miR-143; ccr-miR-143; aca-miR-143-3p; hsa-miR-143-5p |
| 35 | mir-1434 | bta-miR-1434-3p |
| 36 | mir-144 | chi-miR-144-3p; chi-miR-144-5p; aca-miR-144-5p; ccr-miR-144; aca-miR-144-3p; ssa-miR-144-5p; mmu-miR-144-5p; cgr-miR-144; ssa-miR-144-3p |
| 37 | mir-145 | bta-miR-145; efu-miR-145; mml-miR-145-3p; mmu-miR-145a-3p; mdo-miR-145-3p; rno-miR-145-3p; oan-miR-145-5p; aca-miR-145-5p; ggo-miR-145; chi-miR-145-3p; aca-miR-145-3p; aja-miR-145 |
| 38 | mir-146 | eca-miR-146b-3p; chi-miR-146b-3p; bta-miR-146b; cfa-miR-146a; cgr-miR-146b-3p; cfa-miR-146b; cgr-miR-146b-5p; aca-miR-146a-5p; ggo-miR-146a |
| 39 | mir-1468 | eca-miR-1468; bta-miR-1468; cfa-miR-1468 |
| 40 | mir-1473 | bta-miR-6123 |
| 41 | mir-148 | aca-miR-148a-3p; cgr-miR-152-5p; bta-miR-152; chi-miR-148a-5p; ggo-miR-148a; cfa-miR-152 |
| 42 | mir-15 | age-miR-16; age-miR-15a; cgr-miR-15b-3p; cgr-miR-16-3p; eca-miR-195; rno-miR-16-3p; mml-miR-195-3p; efu-miR-16; hsa-miR-195-3p; cfa-miR-15b; bta-miR-195; bta-miR-16b; cgr-miR-15a-5p; bta-miR-15a; hsa-miR-16-1-3p; aca-miR-16a-5p; chi-miR-195-3p; chi-miR-16b-3p; chi-miR-15a-5p; rno-miR-15a-5p; age-miR-15b; hsa-miR-15b-3p |
| 43 | mir-150 | aca-miR-150-5p; bta-miR-150 |
| 44 | mir-154 | CM008020.1_143075; bta-miR-1185; oar-miR-1185-5p; oar-miR-323b; bta-miR-655; bta-miR-154a; chi-miR-409-3p; bta-miR-154c; bta-miR-381; ggo-miR-409; hsa-miR-487b-5p; cfa-miR-377; bta-miR-369-3p; oar-miR-3958-5p; chi-miR-154b-5p; oar-miR-494-5p; chi-miR-494; cgr-miR-369-5p; rno-miR-409a-3p; bta-miR-323; bta-miR-377; chi-miR-1185-5p; bta-miR-154b; chi-miR-1185-3p; oar-miR-409-3p; cfa-miR-323; bta-miR-494; cgr-miR-409-5p; cfa-miR-382; cfa-miR-3958; ggo-miR-381; oar-miR-487b-5p; bta-miR-409a; bta-miR-382; oar-miR-539-3p; hsa-miR-539-3p; bta-miR-496; bta-miR-656; ssc-miR-382; efu-miR-409; cgr-miR-409-3p; oar-miR-154a-3p; chi-miR-496-3p; chi-miR-154b-3p; chi-miR-382-3p; ggo-miR-323a; efu-miR-487; chi-miR-369-5p; cgr-miR-377-3p; bta-miR-487b; efu-miR-381; ggo-miR-487b; oar-miR-381-3p; bta-miR-539; efu-miR-323; mml-miR-382-3p; ggo-miR-656; eca-miR-154b; ggo-miR-410; bta-miR-410 |
| 45 | mir-155 | ipu-miR-155; bta-miR-155; efu-miR-155; aca-miR-155-5p; tgu-miR-155-5p |
| 46 | mir-17 | age-miR-106b; oan-miR-106-5p; chi-miR-106b-3p; dre-miR-17a-5p; chi-miR-20b; hsa-miR-106a-5p; ola-miR-20a; bta-miR-20a; ipu-miR-18a; cgr-miR-18a-3p; age-miR-106a; aca-miR-20a-5p; age-miR-93; aca-miR-18a-3p; efu-miR-106; oan-miR-20a-5p; ola-miR-106a; aca-miR-17-5p; bta-miR-106a; rno-miR-18a-3p; chi-miR-93-3p; cgr-miR-106b-3p; age-miR-17-5p; pma-miR-20a-5p; aca-miR-17-3p; mml-miR-106b-3p; age-miR-17-3p; bta-miR-93; sha-miR-93; aca-miR-18a-5p; cfa-miR-93; mdo-miR-18a-3p; efu-miR-20; age-miR-18; mdo-miR-17-3p; bta-miR-20b; chi-miR-17-3p; cgr-miR-106b-5p |
| 47 | mir-181 | efu-miR-181a; aca-miR-181a; ccr-miR-181b; ccr-miR-181a; cfa-miR-181b; sha-miR-181a-3p; bta-miR-181b; cgr-miR-181a-3p; cgr-miR-181c-3p; aca-miR-181b; cfa-miR-181c; bta-miR-181c; bta-miR-181a; chi-miR-181c-5p; tgu-miR-181b-1-3p; dre-miR-181b-3p; mdo-miR-181a-1-3p; efu-miR-181f; chi-miR-181b-5p; efu-miR-181e; cgr-miR-181c-5p; hsa-miR-181b-3p; cgr-miR-181b-3p; hsa-miR-181b-2-3p; efu-miR-181d; ola-miR-181a-5p; hsa-miR-181c-3p; cfa-miR-181a; bta-miR-181d |
| 48 | mir-1814 | chi-miR-1814 |
| 49 | mir-182 | eca-miR-182; aca-miR-182-5p; ccr-miR-182-5p; bta-miR-182 |
| 50 | mir-183 | cgr-miR-183; hhi-miR-183; bta-miR-183; bfl-miR-183 |
| 51 | mir-1839 | cfa-miR-1839; bta-miR-1839; cgr-miR-1839-5p; mmu-miR-1839-3p; cgr-miR-1839-3p |
| 52 | mir-184 | aga-miR-184; aca-miR-184-3p |
| 53 | mir-1842 | cfa-miR-1842 |
| 54 | mir-185 | efu-miR-185; bta-miR-185 |
| 55 | mir-186 | bta-miR-186; cgr-miR-186-5p |
| 56 | mir-187 | cgr-miR-187; chi-miR-187; bta-miR-187 |
| 57 | mir-188 | bta-miR-660; bta-miR-188; chi-miR-660; efu-miR-532; bta-miR-532; cgr-miR-188; chi-miR-532-3p; ggo-miR-532; eca-miR-532-3p; chi-miR-188-5p |
| 58 | mir-19 | aca-miR-19b; age-miR-19b; cgr-miR-19b-5p; age-miR-19a; aca-miR-19a-3p; cfa-miR-19b |
| 59 | mir-190 | CM008027.1_210382; bta-miR-190a; aca-miR-190a-5p; cgr-miR-190a |
| 60 | mir-191 | aca-miR-191-5p; cgr-miR-191-3p; ggo-miR-191; ocu-miR-191-3p; cfa-miR-191 |
| 61 | mir-1911 | mml-miR-1911-5p; hsa-miR-1911-5p |
| 62 | mir-1912 | eca-miR-1912 |
| 63 | mir-192 | chi-miR-215-5p; bta-miR-215; bta-miR-192; CM008021.1_148608; cgr-miR-192; cfa-miR-192; ola-miR-192-5p |
| 64 | mir-193 | ola-miR-193a; cgr-miR-193b-3p; sha-miR-193; ggo-miR-193a; aca-miR-193-5p; bta-miR-193a-3p; chi-miR-193a; cfa-miR-193b; bta-miR-193a-5p; bta-miR-193b; CM008020.1_145771 |
| 65 | mir-194 | tch-miR-194-5p; hsa-miR-194-3p; aca-miR-194-5p; CM008021.1_148609; ola-miR-194-5p; dre-miR-194a |
| 66 | mir-197 | age-miR-197 |
| 67 | mir-199 | aca-miR-199a-5p; efu-miR-199; dre-miR-199-3p; ccr-miR-199-5p; bta-miR-199c; bta-miR-199b; pma-miR-199a-3p; mdo-miR-199b-2-5p; bta-miR-199a-3p; bta-miR-199a-5p; ssc-miR-199b-5p; ccr-miR-199-3p |
| 68 | mir-202 | bta-miR-202 |
| 69 | mir-204 | ola-miR-204; aca-miR-204a-3p; aca-miR-204a-5p; rno-miR-204-3p; chi-miR-211 |
| 70 | mir-205 | cgr-miR-205; aca-miR-205a; efu-miR-205; oan-miR-205-5p |
| 71 | mir-21 | sha-miR-21; chi-miR-21-3p; bta-miR-21-5p; aca-miR-21-5p; aja-miR-21; chi-miR-21-5p |
| 72 | mir-210 | bta-miR-210; bfl-miR-210-3p; cgr-miR-210-5p |
| 73 | mir-214 | aca-miR-214-3p; chi-miR-214-3p; oan-miR-214-5p; aja-miR-3120; aca-miR-214-5p; efu-miR-214; ccr-miR-214; age-miR-214 |
| 74 | mir-2154 | CM008016.1_90401 |
| 75 | mir-2160 | CM008019.1_130001 |
| 76 | mir-218 | ipu-miR-218a; aca-miR-218-5p; efu-miR-218a; mdo-miR-218-2-3p; age-miR-218 |
| 77 | mir-22 | age-miR-22; cgr-miR-22-5p; bta-miR-22-5p; bta-miR-22-3p; chi-miR-22-3p |
| 78 | mir-221 | cgr-miR-222-3p; ola-miR-222; hsa-miR-221-5p; age-miR-222; rno-miR-221-5p; ccr-miR-221; chi-miR-221-5p; ccr-miR-222; efu-miR-221; bta-miR-222; sha-miR-221; aca-miR-221-3p; aca-miR-222a-3p; cgr-miR-221-5p; cgr-miR-221-3p; gga-miR-221-5p |
| 79 | mir-223 | ola-miR-223; cfa-miR-223; bta-miR-223; mmu-miR-223-5p; hsa-miR-223-5p; efu-miR-223 |
| 80 | mir-224 | bta-miR-224; chi-miR-224-3p; eca-miR-224; cfa-miR-224 |
| 81 | mir-2284 | CM008031.1_262970; CM008012.1_49180; CM008028.1_233163; CM008013.1_67139; CM008030.1_244854; CM008024.1_183606; CM008036.1_295794; CM008008.1_5534; CM008029.1_242977; bta-miR-2285b; CM008026.1_208845; CM008014.1_69773; CM008008.1_6671; CM008030.1_248867; CM008025.1_192294; CM008031.1_263955; CM008025.1_186278; CM008038.1_307359; CM008034.1_285336; CM008018.1_110103; CM008016.1_97064; CM008021.1_147678; CM008028.1_229104; CM008012.1_53957; CM008018.1_119020; CM008009.1_16740; CM008040.1_317809; CM008028.1_228985; bta-miR-2284y; CM008040.1_323630; CM008041.1_325871; CM008008.1_10340; CM008031.1_259110; CM008018.1_120835; CM008012.1_42960; CM008014.1_76350; CM008019.1_131447; CM008032.1_269285; CM008041.1_329711; CM008027.1_215433; CM008012.1_47852; CM008012.1_42107; CM008015.1_81455; CM008023.1_171704; CM008037.1_301443; CM008025.1_186565; CM008037.1_300926; CM008021.1_154130; bta-miR-2285f; CM008010.1_26918; CM008019.1_130171; CM008021.1_154122; CM008028.1_234298; bta-miR-2285ab; CM008041.1_326111; CM008019.1_131018; CM008012.1_55392; CM008027.1_215941; CM008019.1_138018; CM008022.1_159625; CM008016.1_100327; CM008020.1_147241; CM008022.1_158829; bta-miR-2285k; CM008027.1_215935; CM008010.1_21891; CM008023.1_173900; CM008022.1_165248; CM008016.1_100323; CM008019.1_138754; CM008019.1_134217; CM008041.1_334666; CM008029.1_238965; CM008030.1_251217; bta-miR-2285c; CM008025.1_185853; CM008028.1_233164; CM008036.1_292916; CM008016.1_96188; CM008041.1_329257; CM008030.1_251835; CM008036.1_294342; bta-miR-2284x; CM008022.1_167031; CM008041.1_334231; CM008023.1_177547; CM008019.1_131998; bta-miR-2285l; CM008036.1_292007; CM008008.1_7149; CM008030.1_248613; CM008022.1_171200; CM008030.1_244939; bta-miR-2284ab; CM008013.1_68686; CM008012.1_43443; CM008037.1_302471; CM008025.1_188371; CM008030.1_245504; CM008032.1_270582; CM008041.1_334512 |
| 82 | mir-23 | cgr-miR-23b-5p; aca-miR-23b-3p; cfa-miR-23b; efu-miR-23b; aca-miR-23b-5p; chi-miR-23b-5p; cfa-miR-23a; oar-miR-23b; CM008012.1_62189; cgr-miR-23a-5p; dre-miR-23b; bta-miR-23a; aca-miR-23a-3p; tch-miR-23b-3p; bta-miR-23b-3p; mdo-miR-23a-3p |
| 83 | mir-2331 | chi-miR-2331; bta-miR-2331-3p |
| 84 | mir-2332 | bta-miR-2332 |
| 85 | mir-2355 | bta-miR-2355-3p |
| 86 | mir-2366 | bta-miR-2366 |
| 87 | mir-24 | chi-miR-2331; bta-miR-2331-3p; aca-miR-24-3p; hsa-miR-24-1-5p; hsa-miR-24-2-5p; sha-miR-24; oan-miR-24-1-5p; cfa-miR-24; bta-miR-24; rno-miR-24-1-5p; ola-miR-24b-3p; mdo-miR-24-5p; chi-miR-24-5p; chi-miR-24-3p |
| 88 | mir-2404 | MKHE01004964.1_338157 |
| 89 | mir-25 | mmu-miR-25-5p; hsa-miR-92a-1-5p; cqu-miR-92; cgr-miR-92b-5p; oar-miR-25; aga-miR-92a; bta-miR-92b; aca-miR-92a; aja-miR-25; efu-miR-25; hsa-miR-25-5p; bma-miR-92; sha-miR-25; gga-miR-92-3p; xtr-miR-92b; aae-miR-92a-3p; hsa-miR-92b-5p; cgr-miR-25-5p |
| 90 | mir-26 | ggo-miR-26b; mml-miR-26b-3p; gga-miR-26a-5p; efu-miR-26b; bta-miR-26a; chi-miR-26a-3p; bta-miR-26b; cgr-miR-26b-3p; aca-miR-26-5p |
| 91 | mir-27 | dre-miR-27b-5p; aca-miR-27b-5p; bta-miR-27b; age-miR-27a; ola-miR-27d-3p; oha-miR-27b-5p; bta-miR-27a-5p; aca-miR-27a-3p; bta-miR-27a-3p; aca-miR-27b-3p |
| 92 | mir-276 | CM008016.1_87034 |
| 93 | mir-2780 | bta-miR-2419-5p |
| 94 | mir-28 | ggo-miR-151a; age-miR-28; cgr-miR-28-5p; bta-miR-151-3p; efu-miR-28; bta-miR-151-5p; chi-miR-28-3p; hsa-miR-151b; ssc-miR-151-3p |
| 95 | mir-29 | mml-miR-29c-5p; ola-miR-29c; aja-miR-29c; bta-miR-29d-5p; cgr-miR-29c-5p; CM008041.1_329491; chi-miR-29c-5p; CM008037.1_302513; tgu-miR-29a-1-5p; chi-miR-29b-5p; aja-miR-29b; aca-miR-29b; gga-miR-29c-3p; chi-miR-29a-5p; aca-miR-29a-3p; CM008012.1_55433; mmu-miR-29b-2-5p; CM008033.1_276646; cgr-miR-29a-5p; hsa-miR-29a-5p; cfa-miR-29a; cgr-miR-29b-5p; efu-miR-29a; bta-miR-29a; age-miR-29b; tgu-miR-29b-2-5p; hsa-miR-29c-5p; age-miR-29a; cgr-miR-29a-3p; dre-miR-29b |
| 96 | mir-296 | ssc-miR-296-3p; bta-miR-296-3p; chi-miR-296-3p |
| 97 | mir-299 | mml-miR-299-3p; efu-miR-299; bta-miR-299; ssc-miR-299; rno-miR-299a-3p |
| 98 | mir-30 | chi-miR-30a-3p; chi-miR-30f-5p; ssc-miR-30c-1-3p; efu-miR-30b; aca-miR-30c-3p; aca-miR-30b-5p; bta-miR-30d; bta-miR-30f; gga-miR-30c-2-3p; oha-miR-30e-5p; aca-miR-30d-3p; efu-miR-30d; chi-miR-30c-3p; tch-miR-30a-5p; hsa-miR-30c-1-3p; bta-miR-30a-5p; aca-miR-30a-3p; cfa-miR-30c; mdo-miR-30a-3p; gga-miR-30e-3p; bta-miR-30e-5p; aca-miR-30a-5p; mml-miR-30c-1-3p; chi-miR-30e-3p; efu-miR-30c; age-miR-30b; ola-miR-30a-3p; aca-miR-30c-5p; aca-miR-30e-3p; aca-miR-30e-5p; efu-miR-30e; cfa-miR-30a; dre-miR-30e-5p; cgr-miR-30d; dre-miR-30c-5p; aca-miR-30d-5p; gga-miR-30e-5p; oar-miR-30d; ccr-miR-30d; bta-miR-30c |
| 99 | mir-302 | eca-miR-302d |
| 100 | mir-3059 | mml-miR-3059-5p |
| 101 | mir-3064 | hsa-miR-3064-5p |
| 102 | mir-31 | CM008018.1_113821 |
| 103 | mir-3154 | bta-miR-3154 |
| 104 | mir-32 | aca-miR-32-5p; oan-miR-32-5p; cgr-miR-32-5p; cgr-miR-32-3p |
| 105 | mir-320 | efu-miR-320; ssc-miR-320; bta-miR-320a; ptr-miR-320d |
| 106 | mir-322 | bta-miR-424-3p; eca-miR-424; mml-miR-424-3p; bta-miR-424-5p |
| 107 | mir-324 | chi-miR-324-3p; cgr-miR-324-5p; bta-miR-324; mmu-miR-324-3p; hsa-miR-324-3p |
| 108 | mir-326 | chi-miR-326-3p; bta-miR-326; chi-miR-326-5p |
| 109 | mir-328 | ggo-miR-328; bta-miR-328 |
| 110 | mir-329 | chi-miR-329b-3p; CM008041.1_326329; oar-miR-543-3p; bta-miR-495; ggo-miR-495; chi-miR-543-3p; CM008022.1_171045; cfa-miR-329b; efu-miR-495; bta-miR-543; mml-miR-543-5p; chi-miR-543-5p |
| 111 | mir-33 | aae-miR-33; chi-miR-33b-3p; chi-miR-33a-3p; cfa-miR-33a |
| 112 | mir-331 | hsa-miR-331-5p; efu-miR-331; chi-miR-331-5p; aja-miR-331; chi-miR-331-3p; cgr-miR-331-5p; cgr-miR-331-3p |
| 113 | mir-335 | chi-miR-335-3p; ggo-miR-335; chi-miR-335-5p; bta-miR-335 |
| 114 | mir-338 | aca-miR-338-3p; bta-miR-338; chi-miR-338-5p |
| 115 | mir-339 | cfa-miR-339; bta-miR-339a; cgr-miR-339; bta-miR-339b; ssc-miR-339 |
| 116 | mir-34 | aca-miR-34b-5p; mdo-miR-34a-3p; cfa-miR-34c; hsa-miR-34a-3p; efu-miR-34a; age-miR-34a; gga-miR-34a-5p; chi-miR-34b-3p |
| 117 | mir-342 | bta-miR-342; chi-miR-342-5p; chi-miR-342-3p |
| 118 | mir-3431 | bta-miR-3431; chi-miR-3431-3p |
| 119 | mir-3432 | chi-miR-3432-5p |
| 120 | mir-345 | bta-miR-345-5p; cfa-miR-345; bta-miR-345-3p; ssc-miR-345-3p; eca-miR-345-3p; chi-miR-345-3p |
| 121 | mir-359 | CM008020.1_147456 |
| 122 | mir-361 | ssc-miR-361-3p; tch-miR-361-5p; hsa-miR-361-3p; chi-miR-361-3p; bta-miR-361 |
| 123 | mir-362 | cgr-miR-362; ssc-miR-362; bta-miR-362-5p; eca-miR-362-3p; bta-miR-362-3p |
| 124 | mir-363 | bta-miR-363; sha-miR-92a; chi-miR-363-3p |
| 125 | mir-365 | tgu-miR-365-1-5p; aca-miR-365-5p; gga-miR-365-1-5p; aca-miR-365-3p; gga-miR-365-2-5p; bta-miR-365-5p; ggo-miR-365a; hsa-miR-365b-5p |
| 126 | mir-368 | rno-miR-376c-5p; chi-miR-376b-3p; bta-miR-376c; mmu-miR-376a-5p; eca-miR-376b; mml-miR-376a-1-5p; bta-miR-376a; hsa-miR-376c-5p; oar-miR-376a-5p; chi-miR-376b-5p; bta-miR-376d; bta-miR-376b; bta-miR-376e; chi-miR-376a; ggo-miR-376b; hsa-miR-376a-5p; chi-miR-376c-3p; ggo-miR-376c |
| 127 | mir-370 | mmu-miR-370-5p; bta-miR-370; hsa-miR-370-5p; rno-miR-370-5p; oar-miR-370-3p; ggo-miR-370 |
| 128 | mir-374 | bta-miR-374b; bta-miR-374a; chi-miR-374a-3p; oar-miR-374b; tch-miR-374b-5p; cgr-miR-374-3p; cfa-miR-374a |
| 129 | mir-375 | cfa-miR-375 |
| 130 | mir-378 | bta-miR-378; ggo-miR-378a; mmu-miR-378b; ppy-miR-378d; eca-miR-378; ppy-miR-378e; cgr-miR-378-5p; hsa-miR-378d; hsa-miR-378f; mmu-miR-378c |
| 131 | mir-379 | oar-miR-379-5p; chi-miR-411a-5p; mmu-miR-758-3p; bta-miR-411c-5p; chi-miR-379-3p; rno-miR-411-3p; bta-miR-1197; rno-miR-379-3p; oar-miR-758-3p; chi-miR-411b-3p; eca-miR-411; ssc-miR-758; mml-miR-380-5p; ssc-miR-411; bta-miR-379; bta-miR-758; chi-miR-411a-3p; bta-miR-411b; efu-miR-379; hsa-miR-379-3p; hsa-miR-411-3p; eca-miR-3959; bta-miR-380-3p; bta-miR-411c-3p; bta-miR-411a |
| 132 | mir-3955 | CM008019.1_128870; CM008016.1_89428 |
| 133 | mir-3956 | oar-miR-3956-5p |
| 134 | mir-3957 | CM008020.1_143069 |
| 135 | mir-412 | rno-miR-412-3p; cgr-miR-412-3p; chi-miR-412-5p; mml-miR-412-5p |
| 136 | mir-423 | bta-miR-423-3p; cgr-miR-423-3p; ggo-miR-423; efu-miR-423; bta-miR-423-5p |
| 137 | mir-425 | bta-miR-425-3p; cgr-miR-425-3p; chi-miR-425-3p; bta-miR-425-5p; cfa-miR-425; mmu-miR-425-3p |
| 138 | mir-431 | hsa-miR-431-5p; bta-miR-431; hsa-miR-431-3p; ggo-miR-431 |
| 139 | mir-432 | ssc-miR-432-5p; ggo-miR-432; bta-miR-432 |
| 140 | mir-433 | bta-miR-433; ggo-miR-433 |
| 141 | mir-449 | bta-miR-449b; bta-miR-449a; chi-miR-449a-5p; chi-miR-449a-3p |
| 142 | mir-451 | aca-miR-451-5p; bta-miR-451; chi-miR-451-5p |
| 143 | mir-452 | rno-miR-452-3p; efu-miR-452; cfa-miR-452; rno-miR-452-5p; bta-miR-452 |
| 144 | mir-454 | aca-miR-454-3p; ggo-miR-454; oan-miR-454-5p; cfa-miR-454; chi-miR-454-5p |
| 145 | mir-483 | bta-miR-483; chi-miR-483 |
| 146 | mir-485 | chi-miR-485-3p; ggo-miR-485; eca-miR-485-3p; bta-miR-485; mmu-miR-485-3p; chi-miR-485-5p |
| 147 | mir-486 | CM008039.1_315920; cfa-miR-486; bta-miR-486 |
| 148 | mir-490 | bta-miR-490 |
| 149 | mir-4900 | CM008025.1_184735 |
| 150 | mir-491 | chi-miR-491-3p; ggo-miR-491; bta-miR-491 |
| 151 | mir-493 | cfa-miR-493; bta-miR-493; chi-miR-493-5p; efu-miR-493; ggo-miR-493; mmu-miR-493-5p |
| 152 | mir-497 | efu-miR-497; cfa-miR-497; bta-miR-497; ggo-miR-497; chi-miR-497-5p |
| 153 | mir-499 | ggo-miR-499a; aca-miR-499-5p; bta-miR-499 |
| 154 | mir-500 | hsa-miR-500a-3p; efu-miR-500; cfa-miR-500; chi-miR-502b-5p; bta-miR-500; ggo-miR-502b; chi-miR-502b-3p |
| 155 | mir-503 | eca-miR-503; bta-miR-503-3p; efu-miR-503; bta-miR-503-5p |
| 156 | mir-504 | chi-miR-504; bta-miR-504 |
| 157 | mir-505 | chi-miR-505-3p; bta-miR-505; cgr-miR-505-3p; cgr-miR-505-5p |
| 158 | mir-506 | CM008041.1_326625 |
| 159 | mir-541 | CM008020.1_143067; hsa-miR-541-5p |
| 160 | mir-542 | cgr-miR-542-3p; chi-miR-542-3p; ssc-miR-542-5p; chi-miR-542-5p; cfa-miR-542; bta-miR-542-5p |
| 161 | mir-544 | chi-miR-544-5p; oar-miR-544-3p; bta-miR-544a |
| 162 | mir-574 | CM008012.1_46261; CM008011.1_37433; CM008037.1_299589; CM008020.1_143606 |
| 163 | mir-582 | cfa-miR-582; bta-miR-582; ggo-miR-582 |
| 164 | mir-590 | cfa-miR-590 |
| 165 | mir-592 | chi-miR-592 |
| 166 | mir-628 | efu-miR-628; bta-miR-628 |
| 167 | mir-630 | CM008020.1_145882 |
| 168 | mir-6516 | hsa-miR-6516-3p; hsa-miR-6516-5p |
| 169 | mir-652 | ssc-miR-652; bta-miR-652; cgr-miR-652-5p; cfa-miR-652 |
| 170 | mir-6529 | cfa-miR-6529; bta-miR-6529a |
| 171 | mir-654 | bta-miR-654; oar-miR-654-3p |
| 172 | mir-665 | oar-miR-665-3p; bta-miR-665 |
| 173 | mir-671 | bta-miR-671; aja-miR-671; cgr-miR-671-5p |
| 174 | mir-673 | CM008026.1_202979 |
| 175 | mir-7 | cin-miR-7-5p; api-miR-7; efu-miR-7c; fru-miR-7; aae-miR-7; efu-miR-7a |
| 176 | mir-708 | bta-miR-708; cgr-miR-708; chi-miR-708-3p |
| 177 | mir-7180 | mml-miR-7180-3p |
| 178 | mir-744 | cgr-miR-744-3p; cgr-miR-744-5p; bta-miR-744 |
| 179 | mir-760 | ggo-miR-760; bta-miR-760-3p |
| 180 | mir-767 | bta-miR-767 |
| 181 | mir-769 | bta-miR-769 |
| 182 | mir-7857 | ssc-miR-7857-3p; bta-miR-7857 |
| 183 | mir-8 | chi-miR-429; efu-miR-200a; efu-miR-200b; aca-miR-200a-3p; cgr-miR-200b; aca-miR-200b-3p; bta-miR-200a; aca-miR-429-3p; cfa-miR-200a; ccr-miR-200a; bta-miR-200b |
| 184 | mir-874 | efu-miR-874; bta-miR-874; cgr-miR-874 |
| 185 | mir-885 | bta-miR-885 |
| 186 | mir-9 | aca-miR-9-5p; sha-miR-9; bfl-miR-9-5p; aae-miR-9a |
| 187 | mir-95 | cfa-miR-421; bta-miR-421; bta-miR-95; eca-miR-421; chi-miR-545-3p; mmu-miR-1264-5p |
| 188 | mir-96 | chi-miR-96; bfl-miR-96-5p; ggo-miR-96 |
