# Supplementary material for: RNA sequencing-based identification of microRNAs in the antler cartilage of Gansu red deer (Cervus elaphus kansuensis)
Source: PeerJ. 2022 Sep 21;10:e13947. doi: 10.7717/peerj.13947 (PMC9508884; doi:10.7717/peerj.13947)
Supplement: Table S3 [file peerj-10-13947-s004.docx]

**SupplementaryTable S3 All miRNAs**

| Index | #miRNA ID | miRNA Sequence | Count |  |  | Length | TMP |  |  |
| --- | --- | --- | --- | --- | --- | --- | --- | --- | --- |
|  |  |  | 30 d | 60 d | 90 d |  | 30 d | 60 d | 90 d |
| 1 | aae-miR-100 | AACCCGUAGAUCCGAACUUGUG | 4031 | 10639 | 8550 | 22 | 1173.9488 | 2441.0384 | 2484.3147 |
| 2 | aae-miR-125-5p | UCCCUGAGACCCUAACUUGUGA | 976 | 3709 | 1862 | 22 | 284.2406 | 851.0021 | 541.0285 |
| 3 | aae-miR-133 | UUGGUCCCCUUCAACCAGCUGU | 14 | 18 | 19 | 22 | 4.0772 | 4.1300 | 5.5207 |
| 4 | aae-miR-33 | GUGCAUUGUAGUUGCAUUGCA | 1 | 5 | 4 | 21 | 0.3051 | 1.2018 | 1.2176 |
| 5 | aae-miR-7 | UGGAAGACUAGUGAUUUUGUUGU | 4307 | 2416 | 2479 | 23 | 1199.7923 | 530.2316 | 688.9883 |
| 6 | aae-miR-92a-3p | UAUUGCACUUGUCCCGGCCUA | 10 | 25 | 18 | 21 | 3.0510 | 6.0092 | 5.4792 |
| 7 | aae-miR-9a | UCUUUGGUUAUCUAGCUGUAUGA | 5 | 13 | 7 | 23 | 1.3928 | 2.8531 | 1.9455 |
| 8 | aca-let-7a-3p | CUAUACAGUCUACUGUCUUUC | 5 | 11 | 8 | 21 | 1.5255 | 2.6441 | 2.4352 |
| 9 | aca-let-7a-5p | UGAGGUAGUAGGUUGUAUAGUU | 16504 | 30258 | 22389 | 22 | 4806.4626 | 6942.4702 | 6505.4176 |
| 10 | aca-let-7c-1-3p | CUGUACAACCUUCUAGCUUUCC | 5 | 29 | 21 | 22 | 1.4562 | 6.6538 | 6.1018 |
| 11 | aca-let-7c-2-3p | CUGUACAGCCUCCUAGCUUUCC | 0 | 12 | 4 | 22 | 0.0000 | 2.7533 | 1.1623 |
| 12 | aca-let-7c-5p | UGAGGUAGUAGGUUGUAUGGUU | 4662 | 18099 | 8731 | 22 | 1357.7150 | 4152.6792 | 2536.9066 |
| 13 | aca-let-7d-5p | AGAGGUAGUAGGUUGCAUAGU | 336 | 548 | 355 | 21 | 102.5130 | 131.7218 | 108.0618 |
| 14 | aca-let-7f-1-3p | CUAUACAAUCUACUGUCUUU | 134 | 143 | 133 | 20 | 42.9273 | 36.0913 | 42.5094 |
| 15 | aca-let-7f-2-3p | CUAUACAAUCUAUUGCCUUCC | 13 | 13 | 12 | 21 | 3.9663 | 3.1248 | 3.6528 |
| 16 | aca-let-7f-5p | UGAGGUAGUAGAUUGUAUAGUU | 26320 | 39435 | 28763 | 22 | 7665.1778 | 9048.0638 | 8357.4669 |
| 17 | aca-let-7g | UGAGGUAGUAGUUUGUACAGUU | 37827 | 55593 | 39451 | 22 | 11016.3633 | 12755.3952 | 11463.0055 |
| 18 | aca-let-7i-3p | CUGCGCAAGCUACUGCCUUGCUA | 5 | 21 | 14 | 23 | 1.3928 | 4.6088 | 3.8910 |
| 19 | aca-let-7i-5p | UGAGGUAGUAGUUUGUGCUGUU | 65670 | 114404 | 74011 | 22 | 19125.0846 | 26249.1362 | 21504.8669 |
| 20 | aca-miR-101-3p | UACAGUACUGUGAUAACUGA | 381 | 803 | 523 | 20 | 122.0546 | 202.6665 | 167.1610 |
| 21 | aca-miR-103-3p | AGCAGCAUUGUACAGGGCUAUG | 1117 | 1706 | 1139 | 22 | 325.3041 | 391.4289 | 330.9514 |
| 22 | aca-miR-107-3p | AGCAGCAUUGUACAGGGCUAUC | 183 | 308 | 220 | 22 | 53.2951 | 70.6683 | 63.9239 |
| 23 | aca-miR-10a-5p | UACCCUGUAGAUCCGAAUUUGUG | 22 | 82 | 17 | 23 | 6.1285 | 17.9963 | 4.7248 |
| 24 | aca-miR-10b-3p | AGAUUCGAUUCUAGGGGAAU | 2 | 9 | 5 | 20 | 0.6407 | 2.2715 | 1.5981 |
| 25 | aca-miR-10b-5p | UACCCUGUAGAACCGAAUUUGU | 1773 | 2395 | 2131 | 22 | 516.3511 | 549.5147 | 619.1900 |
| 26 | aca-miR-126-3p | UCGUACCGUGAGUAAUAAUGCG | 7951 | 12854 | 10535 | 22 | 2315.5710 | 2949.2535 | 3061.0824 |
| 27 | aca-miR-126-5p | CAUUAUUACUUUUGGUACGCG | 64 | 131 | 101 | 21 | 19.5263 | 31.4882 | 30.7443 |
| 28 | aca-miR-128-3p | UCACAGUGAACCGGUCUCUUU | 165 | 183 | 106 | 21 | 50.3412 | 43.9874 | 32.2663 |
| 29 | aca-miR-129a-5p | CUUUUUGCGGUCUGGGCUUGC | 7 | 9 | 38 | 21 | 2.1357 | 2.1633 | 11.5672 |
| 30 | aca-miR-129b-3p | AAGCCCUUACCCCAAAAAGCA | 1 | 0 | 6 | 21 | 0.3051 | 0.0000 | 1.8264 |
| 31 | aca-miR-1306 | UGGACGUUGGCUCUGGUGGUGA | 6 | 10 | 5 | 22 | 1.7474 | 2.2944 | 1.4528 |
| 32 | aca-miR-133a | UUUGGUCCCCUUCAACCAGCUG | 14 | 18 | 19 | 22 | 4.0772 | 4.1300 | 5.5207 |
| 33 | aca-miR-135-5p | UAUGGCUUUUUAUUCCUAUGUGA | 1 | 2 | 1 | 23 | 0.2786 | 0.4389 | 0.2779 |
| 34 | aca-miR-138-5p | AGCUGGUGUUGUGAAUCAGGCC | 11 | 12 | 33 | 22 | 3.2035 | 2.7533 | 9.5886 |
| 35 | aca-miR-140-3p | UACCACAGGGUAGAACCACGG | 3361 | 3342 | 3749 | 21 | 1025.4353 | 803.3109 | 1141.1936 |
| 36 | aca-miR-140-5p | CAGUGGUUUUACCCUAUGGU | 1176 | 849 | 788 | 20 | 376.7353 | 214.2763 | 251.8601 |
| 37 | aca-miR-143-3p | UGAGAUGAAGCACUGUAGCUC | 13096 | 29498 | 21749 | 21 | 3995.5668 | 7090.3841 | 6620.3838 |
| 38 | aca-miR-143-5p | GGUGCAGUGCUGCAUCUCUGG | 89 | 178 | 132 | 21 | 27.1537 | 42.7856 | 40.1807 |
| 39 | aca-miR-144-3p | UACAGUAUAGAUGAUGUACU | 4 | 51 | 15 | 20 | 1.2814 | 12.8717 | 4.7943 |
| 40 | aca-miR-144-5p | GGAUAUCAUCAUAUACUGUAA | 6 | 54 | 17 | 21 | 1.8306 | 12.9799 | 5.1748 |
| 41 | aca-miR-145-3p | GAUUCCUGGAAAUACUGUUCU | 128 | 197 | 158 | 21 | 39.0526 | 47.3526 | 48.0951 |
| 42 | aca-miR-145-5p | GUCCAGUUUUCCCAGGAAUCCC | 1343 | 1428 | 1171 | 22 | 391.1221 | 327.6438 | 340.2494 |
| 43 | aca-miR-146a-5p | UGAGAACUGAAUUCCAUAGGC | 5 | 33 | 5 | 21 | 1.5255 | 7.9322 | 1.5220 |
| 44 | aca-miR-148a-3p | UCAGUGCACUACAGAACUUUGU | 750540 | 778239 | 700427 | 22 | 218579.8841 | 178561.0776 | 203518.2526 |
| 45 | aca-miR-150-5p | UCUCCCAACCCUUGUACCAGUG | 12 | 46 | 23 | 22 | 3.4948 | 10.5544 | 6.6830 |
| 46 | aca-miR-155-5p | UUAAUGCUAAUCGUGAUAGGGG | 813 | 962 | 939 | 22 | 236.7701 | 220.7237 | 272.8388 |
| 47 | aca-miR-16a-5p | UAGCAGCACGUAAAUAUUGG | 14 | 39 | 27 | 20 | 4.4849 | 9.8431 | 8.6297 |
| 48 | aca-miR-17-3p | ACUGCAGUGAAGGCACUUGUAG | 3 | 6 | 5 | 22 | 0.8737 | 1.3767 | 1.4528 |
| 49 | aca-miR-17-5p | CAAAGUGCUUACAGUGCAGGUAG | 646 | 600 | 424 | 23 | 179.9549 | 131.6800 | 117.8423 |
| 50 | aca-miR-181a | AACAUUCAACGCUGUCGGUGAGU | 536 | 1110 | 916 | 23 | 149.3124 | 243.6080 | 254.5838 |
| 51 | aca-miR-181b | AACAUUCAUUGCUGUCGGUGGG | 367 | 352 | 398 | 22 | 106.8815 | 80.7637 | 115.6441 |
| 52 | aca-miR-182-5p | UUUGGCAAUGGUAGAACUCACA | 14 | 6 | 18 | 22 | 4.0772 | 1.3767 | 5.2301 |
| 53 | aca-miR-184-3p | UGGACGGAGAACUGAUAAGGGU | 87 | 102 | 181 | 22 | 25.3370 | 23.4031 | 52.5919 |
| 54 | aca-miR-18a-3p | ACUGCCCUAAGUGCUCCUUCUG | 3 | 4 | 3 | 22 | 0.8737 | 0.9178 | 0.8717 |
| 55 | aca-miR-18a-5p | UAAGGUGCAUCUAGUGCAGAUAG | 47 | 30 | 21 | 23 | 13.0927 | 6.5840 | 5.8365 |
| 56 | aca-miR-190a-5p | UGAUAUGUUUGAUAUAUUAGGUU | 8 | 10 | 3 | 23 | 2.2285 | 2.1947 | 0.8338 |
| 57 | aca-miR-191-5p | CAACGGAAUCCCAAAAGCAGCUG | 915 | 1370 | 984 | 23 | 254.8897 | 300.6694 | 273.4831 |
| 58 | aca-miR-193-5p | UGGGUCUUUGCGGGCGAGAUG | 50 | 96 | 67 | 21 | 15.2549 | 23.0754 | 20.3948 |
| 59 | aca-miR-194-5p | UGUAACAGCAACUCCAUGUGGA | 37 | 71 | 57 | 22 | 10.7755 | 16.2904 | 16.5621 |
| 60 | aca-miR-199a-5p | CCCAGUGUUCAGACUACCUGU | 9831 | 17881 | 11866 | 21 | 2999.4210 | 4298.0256 | 3612.0039 |
| 61 | aca-miR-19a-3p | UGUGCAAAUCUAUGCAAAACUG | 110 | 47 | 48 | 22 | 32.0353 | 10.7838 | 13.9470 |
| 62 | aca-miR-19b | UGUGCAAAUCCAUGCAAAACU | 239 | 163 | 134 | 21 | 72.9185 | 39.1800 | 40.7895 |
| 63 | aca-miR-1a-3p | UGGAAUGUAAAGAAGUAUGUAU | 1710 | 1281 | 1898 | 22 | 498.0036 | 293.9158 | 551.4888 |
| 64 | aca-miR-200a-3p | UAACACUGUCUGGUAACGAUGU | 9 | 4 | 24 | 22 | 2.6211 | 0.9178 | 6.9735 |
| 65 | aca-miR-200b-3p | UAAUACUGCCUGGUAAUGAUGA | 8 | 3 | 20 | 22 | 2.3298 | 0.6883 | 5.8113 |
| 66 | aca-miR-204a-3p | GCUGGGAAGGCAAAGGGACGU | 4 | 29 | 14 | 21 | 1.2204 | 6.9707 | 4.2616 |
| 67 | aca-miR-204a-5p | UUCCCUUUGUCAUCCUAUGCCU | 120 | 506 | 248 | 22 | 34.9476 | 116.0979 | 72.0597 |
| 68 | aca-miR-205a | UCCUUCAUUCCACCGGAGUCUG | 16 | 12 | 44 | 22 | 4.6597 | 2.7533 | 12.7848 |
| 69 | aca-miR-20a-5p | UAAAGUGCUUAUAGUGCAGGUA | 2313 | 1762 | 1341 | 22 | 673.6154 | 404.2776 | 389.6451 |
| 70 | aca-miR-212-5p | ACCUUGGCUCUAGACUGCUUAC | 1 | 1 | 0 | 22 | 0.2912 | 0.2294 | 0.0000 |
| 71 | aca-miR-214-3p | ACAGCAGGCACAGACAGGCAGU | 970 | 1905 | 1389 | 22 | 282.4933 | 437.0879 | 403.5922 |
| 72 | aca-miR-214-5p | UGCCUGUCUACACUUGCUGUGC | 383 | 720 | 449 | 22 | 111.5412 | 165.1986 | 130.4628 |
| 73 | aca-miR-21-5p | UAGCUUAUCAGACUGAUGUUGA | 215106 | 198623 | 164766 | 22 | 62645.3548 | 45572.5515 | 47874.9226 |
| 74 | aca-miR-218-5p | UUGUGCUUGAUCUAACCAUGUG | 354 | 413 | 482 | 22 | 103.0955 | 94.7597 | 140.0514 |
| 75 | aca-miR-221-3p | AGCUACAUUGUCUGCUGGGUUU | 146 | 206 | 175 | 22 | 42.5196 | 47.2651 | 50.8485 |
| 76 | aca-miR-222a-3p | AGCUACAUCUGGCUACUGGGUC | 223 | 179 | 178 | 22 | 64.9443 | 41.0702 | 51.7202 |
| 77 | aca-miR-23a-3p | AUCACAUUGCCAGGGAUUUCC | 1829 | 2815 | 3054 | 21 | 558.0247 | 676.6368 | 929.6359 |
| 78 | aca-miR-23b-3p | AUCACAUUGCCAGGGAUUACC | 887 | 1685 | 1431 | 21 | 270.6222 | 405.0206 | 435.5956 |
| 79 | aca-miR-23b-5p | GGGUUCCUGGCAUGCUGAUUU | 6 | 3 | 8 | 21 | 1.8306 | 0.7211 | 2.4352 |
| 80 | aca-miR-24-3p | UGGCUCAGUUCAGCAGGAACAG | 753 | 1212 | 980 | 22 | 219.2963 | 278.0843 | 284.7519 |
| 81 | aca-miR-26-5p | UUCAAGUAAUCCAGGAUAGG | 11476 | 15770 | 14591 | 20 | 3676.3730 | 3980.1385 | 4663.5671 |
| 82 | aca-miR-27a-3p | UUCACAGUGGCUAAGUUCCGC | 876 | 2357 | 1889 | 21 | 267.2661 | 566.5481 | 575.0106 |
| 83 | aca-miR-27b-3p | UUCACAGUGGCUAAGUUCUGCA | 25840 | 53525 | 41660 | 22 | 7525.3873 | 12280.9081 | 12104.8595 |
| 84 | aca-miR-27b-5p | AGAGCUUAGCUGAUUGGUGAAC | 88 | 37 | 57 | 22 | 25.6283 | 8.4894 | 16.5621 |
| 85 | aca-miR-29a-3p | UAGCACCAUUUGAAAUCGGUU | 1 | 2 | 1 | 21 | 0.3051 | 0.4807 | 0.3044 |
| 86 | aca-miR-29b | UAGCACCAUUUGAAAUCAGUG | 0 | 1 | 1 | 21 | 0.0000 | 0.2404 | 0.3044 |
| 87 | aca-miR-301a-3p | CAGUGCAAUAGUAUUGUCAAAGC | 1 | 3 | 0 | 23 | 0.2786 | 0.6584 | 0.0000 |
| 88 | aca-miR-30a-3p | CUUUCAGUCGGAUGUUUGCAGC | 9 | 33 | 26 | 22 | 2.6211 | 7.5716 | 7.5546 |
| 89 | aca-miR-30a-5p | UGUAAACAUCCUCGACUGGAAG | 687 | 1994 | 1373 | 22 | 200.0751 | 457.5083 | 398.9432 |
| 90 | aca-miR-30b-5p | UGUAAACAUCCUACACUCAGCU | 321 | 811 | 661 | 22 | 93.4849 | 186.0778 | 192.0622 |
| 91 | aca-miR-30c-3p | CUGGGAGAAGGCUGUUUACUCU | 2 | 10 | 8 | 22 | 0.5825 | 2.2944 | 2.3245 |
| 92 | aca-miR-30c-5p | UGUAAACAUCCUACACUCUCA | 411 | 524 | 349 | 21 | 125.3954 | 125.9530 | 106.2354 |
| 93 | aca-miR-30d-3p | UUUCAGUCAGAUGUUUGCUGC | 0 | 1 | 1 | 21 | 0.0000 | 0.2404 | 0.3044 |
| 94 | aca-miR-30d-5p | UGUAAACAUCCCCGACUGGAAG | 2411 | 2848 | 2458 | 22 | 702.1559 | 653.4522 | 714.2041 |
| 95 | aca-miR-30e-3p | CUUUCAGUCGGAUGUUUACAGC | 101 | 136 | 115 | 22 | 29.4142 | 31.2042 | 33.4148 |
| 96 | aca-miR-30e-5p | UGUAAACAUCCUUGACUGGAAGC | 1019 | 1048 | 787 | 23 | 283.8608 | 230.0011 | 218.7309 |
| 97 | aca-miR-32-5p | UAUUGCACAUUACUAAGUUGCA | 85 | 155 | 81 | 22 | 24.7546 | 35.5636 | 23.5356 |
| 98 | aca-miR-338-3p | UCCAGCAUCAGUGAUUUUGUU | 1 | 5 | 2 | 21 | 0.3051 | 1.2018 | 0.6088 |
| 99 | aca-miR-34b-5p | UGGCAGUGUCUUAGCUGGUUG | 68 | 169 | 82 | 21 | 20.7467 | 40.6222 | 24.9608 |
| 100 | aca-miR-365-3p | UAAUGCCCCUAAAAAUCCUUAU | 232 | 177 | 161 | 22 | 67.5654 | 40.6113 | 46.7807 |
| 101 | aca-miR-365-5p | AGGGACUUUCAGGGGCAGCUGUG | 16 | 5 | 11 | 23 | 4.4571 | 1.0973 | 3.0572 |
| 102 | aca-miR-429-3p | UAAUACUGUCUGGUAAUGCCGU | 3 | 2 | 9 | 22 | 0.8737 | 0.4589 | 2.6151 |
| 103 | aca-miR-451-5p | AAACCGUUACCAUUACUGAGUU | 3357 | 22232 | 8535 | 22 | 977.6596 | 5100.9650 | 2479.9562 |
| 104 | aca-miR-454-3p | UAGUGCAAUAUUGCUUAUAGGGU | 15 | 27 | 22 | 23 | 4.1785 | 5.9256 | 6.1145 |
| 105 | aca-miR-499-5p | UUAAGACUUGCAGUGAUGUUUA | 7 | 9 | 4 | 22 | 2.0386 | 2.0650 | 1.1623 |
| 106 | aca-miR-92a | UAUUGCACUUGUCCCGGCCUGU | 1834 | 2576 | 1674 | 22 | 534.1161 | 591.0438 | 486.4027 |
| 107 | aca-miR-9-5p | UCUUUGGUUAUCUAGCUGUA | 5 | 13 | 7 | 20 | 1.6018 | 3.2810 | 2.2373 |
| 108 | aca-miR-98-5p | UGAGGUAGUAAGUUGUAUUGU | 972 | 1795 | 1006 | 21 | 296.5555 | 431.4611 | 306.2259 |
| 109 | aca-miR-99a-3p | CAAGCUCGCUUCUAUGGGUC | 7 | 33 | 19 | 20 | 2.2425 | 8.3288 | 6.0728 |
| 110 | aca-miR-99a-5p | AACCCGUAGAUCCGAUCUUGUG | 13211 | 49497 | 22479 | 22 | 3847.4416 | 11356.7139 | 6531.5683 |
| 111 | aga-miR-10 | ACCCUGUAGAUCCGAAUUUGU | 0 | 3 | 0 | 21 | 0.0000 | 0.7211 | 0.0000 |
| 112 | aga-miR-184 | UGGACGGAGAACUGAUAAGGG | 1 | 21 | 2 | 21 | 0.3051 | 5.0477 | 0.6088 |
| 113 | aga-miR-92a | UAUUGCACUUGUCCCGGCCUAU | 10 | 25 | 18 | 22 | 2.9123 | 5.7361 | 5.2301 |
| 114 | age-miR-101 | UACAGUACUGUGAUAACUGAAG | 384 | 809 | 527 | 22 | 111.8324 | 185.6190 | 153.1268 |
| 115 | age-miR-103 | AGCAGCAUUGUACAGGGCUAUGA | 1117 | 1706 | 1139 | 23 | 311.1604 | 374.4102 | 316.5622 |
| 116 | age-miR-106a | AAAAGUGCUUACAGUGCAGGUAGC | 2 | 1 | 2 | 24 | 0.5339 | 0.2103 | 0.5327 |
| 117 | age-miR-106b | UAAAGUGCUGACAGUGCAGAU | 117 | 140 | 108 | 21 | 35.6965 | 33.6516 | 32.8751 |
| 118 | age-miR-127 | UCGGAUCCGUCUGAGCUUGGCU | 2457 | 5402 | 3868 | 22 | 715.5525 | 1239.4482 | 1123.8981 |
| 119 | age-miR-128 | UCACAGUGAACCGGUCUCUUUU | 165 | 183 | 106 | 22 | 48.0530 | 41.9880 | 30.7997 |
| 120 | age-miR-15a | UAGCAGCACAUAAUGGUUUGUG | 21 | 34 | 29 | 22 | 6.1158 | 7.8010 | 8.4263 |
| 121 | age-miR-15b | UAGCAGCACAUCAUGGUUUACA | 113 | 169 | 113 | 22 | 32.9090 | 38.7758 | 32.8336 |
| 122 | age-miR-16 | UAGCAGCACGUAAAUAUUGGCG | 441 | 693 | 580 | 22 | 128.4325 | 159.0036 | 168.5266 |
| 123 | age-miR-17-3p | ACUGCAGUGAAGGCACUUGU | 3 | 6 | 5 | 20 | 0.9611 | 1.5143 | 1.5981 |
| 124 | age-miR-17-5p | CAAAGUGCUUACAGUGCAGGUAGU | 646 | 600 | 424 | 24 | 172.4568 | 126.1934 | 112.9322 |
| 125 | age-miR-18 | UAAGGUGCAUCUAGUGCAGAUA | 47 | 30 | 21 | 22 | 13.6878 | 6.8833 | 6.1018 |
| 126 | age-miR-197 | UUCACCACCUUCUCCACCCAGC | 182 | 235 | 194 | 22 | 53.0039 | 53.9190 | 56.3692 |
| 127 | age-miR-19a | UGUGCAAAUCUAUGCAAAACUGA | 110 | 47 | 48 | 23 | 30.6425 | 10.3149 | 13.3406 |
| 128 | age-miR-19b | UGUGCAAAUCCAUGCAAAACUGA | 264 | 178 | 154 | 23 | 73.5419 | 39.0651 | 42.8012 |
| 129 | age-miR-214 | ACAGCAGGCACAGACAGGCAG | 970 | 1905 | 1389 | 21 | 295.9453 | 457.9016 | 422.8108 |
| 130 | age-miR-218 | UUGUGCUUGAUCUAACCAUGU | 357 | 415 | 488 | 21 | 108.9201 | 99.7528 | 148.5469 |
| 131 | age-miR-22 | AAGCUGCCAGUUGAAGAACUGU | 441 | 1247 | 807 | 22 | 128.4325 | 286.1148 | 234.4844 |
| 132 | age-miR-222 | AGCUACAUCUGGCUACUGGGUCUC | 223 | 179 | 178 | 24 | 59.5323 | 37.6477 | 47.4102 |
| 133 | age-miR-27a | UUCACAGUGGCUAAGUUCCGCC | 943 | 2446 | 1983 | 22 | 274.6300 | 561.2163 | 576.1867 |
| 134 | age-miR-28 | AAGGAGCUCACAGUCUAUUGAG | 99 | 207 | 166 | 22 | 28.8318 | 47.4946 | 48.2335 |
| 135 | age-miR-29a | CUAGCACCAUCUGAAAUCGGUU | 175 | 253 | 170 | 22 | 50.9653 | 58.0489 | 49.3957 |
| 136 | age-miR-29b | UAGCACCAUUUGAAAUCAGU | 14 | 8 | 9 | 20 | 4.4849 | 2.0191 | 2.8766 |
| 137 | age-miR-30b | UGUAAACAUCCUACACUCAGC | 321 | 811 | 661 | 21 | 97.9365 | 194.9387 | 201.2080 |
| 138 | age-miR-34a | UGGCAGUGUCUUAGCUGGUUGU | 68 | 169 | 82 | 22 | 19.8037 | 38.7758 | 23.8262 |
| 139 | age-miR-93 | AAAGUGCUGUUCGUGCAGGUAG | 563 | 567 | 424 | 22 | 163.9626 | 130.0939 | 123.1988 |
| 140 | age-miR-98 | UGAGGUAGUAAGUUGUAUUGUU | 972 | 1795 | 1006 | 22 | 283.0757 | 411.8492 | 292.3065 |
| 141 | aja-let-7i | CUGCGCAAGCUACUGCCUUGCU | 5 | 21 | 14 | 22 | 1.4562 | 4.8183 | 4.0679 |
| 142 | aja-miR-143 | UGAGAUGAAGCACUGUAGCUCG | 13096 | 29498 | 21749 | 22 | 3813.9502 | 6768.0939 | 6319.4572 |
| 143 | aja-miR-145 | GGAUUCCUGGAAAUACUGUUCU | 128 | 197 | 159 | 22 | 37.2775 | 45.2002 | 46.1995 |
| 144 | aja-miR-21 | CAACAGCAGUCGAUGGGCUGUC | 9 | 7 | 7 | 22 | 2.6211 | 1.6061 | 2.0339 |
| 145 | aja-miR-25 | CAUUGCACUUGUCUCGGUCUGA | 577 | 923 | 598 | 22 | 168.0398 | 211.7754 | 173.7567 |
| 146 | aja-miR-29b | UAGCACCAUUUGAAAUCAGUGUU | 14 | 8 | 9 | 23 | 3.9000 | 1.7557 | 2.5014 |
| 147 | aja-miR-29c | UAGCACCAUUUGAAAUCGGUUA | 1 | 2 | 1 | 22 | 0.2912 | 0.4589 | 0.2906 |
| 148 | aja-miR-3120 | CACAGCAAGUGUAGACAGGCA | 2 | 0 | 0 | 21 | 0.6102 | 0.0000 | 0.0000 |
| 149 | aja-miR-331 | GCCCCUGGGCCUAUCCUAGAA | 10 | 17 | 14 | 21 | 3.0510 | 4.0863 | 4.2616 |
| 150 | aja-miR-671 | UCCGGUUCUCAGGGCUCCACC | 17 | 23 | 16 | 21 | 5.1867 | 5.5285 | 4.8704 |
| 151 | ame-let-7 | UGAGGUAGUAGGUUGUAUAGU | 859 | 3075 | 2074 | 21 | 262.0794 | 739.1325 | 631.3245 |
| 152 | api-miR-7 | UGGAAGACUAGUGAUUUUGUUGUU | 4307 | 2416 | 2479 | 24 | 1149.8009 | 508.1386 | 660.2805 |
| 153 | asu-miR-1-3p | UGGAAUGUAAAGAAGUAUGUA | 38 | 53 | 67 | 21 | 11.5937 | 12.7395 | 20.3948 |
| 154 | bfl-miR-133 | UGGUCCCCUUCAACCAGCUGUA | 14 | 18 | 19 | 22 | 4.0772 | 4.1300 | 5.5207 |
| 155 | bfl-miR-183 | UAUGGCACUGGUAGAAUUCACU | 3 | 2 | 10 | 22 | 0.8737 | 0.4589 | 2.9056 |
| 156 | bfl-miR-210-3p | CUGUGCGUGUGACAGCGGCUGA | 9 | 18 | 17 | 22 | 2.6211 | 4.1300 | 4.9396 |
| 157 | bfl-miR-9-5p | UCUUUGGUUAUCUAGCUGUAUG | 5 | 13 | 7 | 22 | 1.4562 | 2.9828 | 2.0339 |
| 158 | bfl-miR-96-5p | UUUGGCACUAGCACAUUUUUGCU | 3 | 4 | 4 | 23 | 0.8357 | 0.8779 | 1.1117 |
| 159 | bma-miR-92 | UAUUGCACUCGUCCCGGCCU | 0 | 1 | 0 | 20 | 0.0000 | 0.2524 | 0.0000 |
| 160 | bta-let-7a-3p | CUAUACAAUCUACUGUCUUUC | 131 | 142 | 131 | 21 | 39.9679 | 34.1323 | 39.8763 |
| 161 | bta-let-7d | AGAGGUAGUAGGUUGCAUAGUU | 336 | 548 | 355 | 22 | 97.8533 | 125.7345 | 103.1499 |
| 162 | bta-let-7e | UGAGGUAGGAGGUUGUAUAGU | 671 | 1440 | 1066 | 21 | 204.7209 | 346.1304 | 324.4898 |
| 163 | bta-miR-101 | UACAGUACUGUGAUAACUGAA | 381 | 803 | 523 | 21 | 116.2424 | 193.0157 | 159.2009 |
| 164 | bta-miR-105a | UCAAAUGCUCAGACUCCUGUGGU | 0 | 0 | 1 | 23 | 0.0000 | 0.0000 | 0.2779 |
| 165 | bta-miR-106a | AAAAGUGCUUACAGUGCAGGUA | 2 | 1 | 2 | 22 | 0.5825 | 0.2294 | 0.5811 |
| 166 | bta-miR-10b | UACCCUGUAGAACCGAAUUUGUG | 1773 | 2395 | 2131 | 23 | 493.9010 | 525.6228 | 592.2687 |
| 167 | bta-miR-1185 | AGAGGAUACCCUUUGUAUGUU | 0 | 2 | 1 | 21 | 0.0000 | 0.4807 | 0.3044 |
| 168 | bta-miR-1197 | UAGGACACAUGGUCUACUUCU | 42 | 97 | 60 | 21 | 12.8141 | 23.3157 | 18.2640 |
| 169 | bta-miR-1246 | AAUGGAUUUUUGGAGCAGG | 30 | 44 | 75 | 19 | 10.1164 | 11.6895 | 25.2331 |
| 170 | bta-miR-125a | UCCCUGAGACCCUUUAACCUGUG | 746 | 1401 | 854 | 23 | 207.8117 | 307.4729 | 237.3522 |
| 171 | bta-miR-1260b | AUCCCACCACUGCCACCA | 32 | 78 | 97 | 18 | 11.3903 | 21.8735 | 34.4479 |
| 172 | bta-miR-126-3p | CGUACCGUGAGUAAUAAUGCG | 7951 | 12854 | 10535 | 21 | 2425.8363 | 3089.6941 | 3206.8483 |
| 173 | bta-miR-1271 | CUUGGCACCUAGUAAGUACUCA | 313 | 387 | 396 | 22 | 91.1550 | 88.7942 | 115.0630 |
| 174 | bta-miR-129 | CUUUUUGCGGUCUGGGCUUGCU | 7 | 9 | 38 | 22 | 2.0386 | 2.0650 | 11.0414 |
| 175 | bta-miR-129-3p | AAGCCCUUACCCCAAAAAGCAU | 1 | 0 | 7 | 22 | 0.2912 | 0.0000 | 2.0339 |
| 176 | bta-miR-1296 | UUAGGGCCCUGGCUCCAUCUCC | 10 | 11 | 9 | 22 | 2.9123 | 2.5239 | 2.6151 |
| 177 | bta-miR-1298 | UUCAUUCGGCUGUCCAGAUGUA | 74 | 438 | 1015 | 22 | 21.5510 | 100.4958 | 294.9216 |
| 178 | bta-miR-1306 | CCACCUCCCCUGCAAACGUCC | 9 | 14 | 11 | 21 | 2.7459 | 3.3652 | 3.3484 |
| 179 | bta-miR-1307 | ACUCGGCGUGGCGUCGGUCGUG | 245 | 234 | 209 | 22 | 71.3514 | 53.6895 | 60.7277 |
| 180 | bta-miR-130b | CAGUGCAAUGAUGAAAGGGCAU | 6 | 14 | 12 | 22 | 1.7474 | 3.2122 | 3.4868 |
| 181 | bta-miR-132 | UAACAGUCUACAGCCAUGGUCG | 6 | 10 | 8 | 22 | 1.7474 | 2.2944 | 2.3245 |
| 182 | bta-miR-1343-3p | CUCCUGGGGCCCGCACUCUC | 7 | 11 | 6 | 20 | 2.2425 | 2.7763 | 1.9177 |
| 183 | bta-miR-135b | UAUGGCUUUUCAUUCCUAUGUGA | 5 | 5 | 2 | 23 | 1.3928 | 1.0973 | 0.5559 |
| 184 | bta-miR-138 | AGCUGGUGUUGUGAAUCAGGCCG | 1 | 3 | 7 | 23 | 0.2786 | 0.6584 | 1.9455 |
| 185 | bta-miR-1388-5p | AGGACUGUCCAACCUGAGAAU | 1 | 6 | 3 | 21 | 0.3051 | 1.4422 | 0.9132 |
| 186 | bta-miR-139 | UCUACAGUGCACGUGUCUCCAGU | 129 | 241 | 180 | 23 | 35.9353 | 52.8915 | 50.0274 |
| 187 | bta-miR-140 | UACCACAGGGUAGAACCACGGA | 10083 | 8861 | 8406 | 22 | 2936.4737 | 2033.0897 | 2442.4736 |
| 188 | bta-miR-1434-3p | GAAGAAAUCUAAGGUCUGAGG | 19 | 11 | 17 | 21 | 5.7969 | 2.6441 | 5.1748 |
| 189 | bta-miR-145 | GUCCAGUUUUCCCAGGAAUCCCU | 1343 | 1429 | 1171 | 23 | 374.1168 | 313.6179 | 325.4560 |
| 190 | bta-miR-1468 | CUCCGUUUGCCUGUUUUGCUGA | 198 | 181 | 208 | 22 | 57.6636 | 41.5291 | 60.4371 |
| 191 | bta-miR-146b | UGAGAACUGAAUUCCAUAGGCUGU | 67 | 199 | 48 | 24 | 17.8864 | 41.8541 | 12.7848 |
| 192 | bta-miR-150 | UCUCCCAACCCUUGUACCAGUGU | 14 | 52 | 33 | 23 | 3.9000 | 11.4123 | 9.1717 |
| 193 | bta-miR-151-3p | CUAGACUGAAGCUCCUUGAGG | 5134 | 7743 | 5496 | 21 | 1566.3745 | 1861.1718 | 1672.9794 |
| 194 | bta-miR-151-5p | UCGAGGAGCUCACAGUCUAGU | 104 | 247 | 162 | 21 | 31.7302 | 59.3710 | 49.3127 |
| 195 | bta-miR-152 | UCAGUGCAUGACAGAACUUGGG | 2219 | 5673 | 4782 | 22 | 646.2397 | 1301.6271 | 1389.4728 |
| 196 | bta-miR-154a | UAGGUUAUCCGUGUAGCCUUCG | 0 | 1 | 0 | 22 | 0.0000 | 0.2294 | 0.0000 |
| 197 | bta-miR-154b | AGAGGUCUUCCAUGGUGCAUUCG | 42 | 83 | 35 | 23 | 11.6999 | 18.2157 | 9.7275 |
| 198 | bta-miR-154c | AGAUAUUGCACGGUUGAUCUCU | 569 | 979 | 634 | 22 | 165.7100 | 224.6242 | 184.2170 |
| 199 | bta-miR-155 | UUAAUGCUAAUCGUGAUAGGGGU | 813 | 962 | 939 | 23 | 226.4758 | 211.1270 | 260.9762 |
| 200 | bta-miR-15a | UAGCAGCACAUAAUGGUUUGU | 21 | 34 | 29 | 21 | 6.4071 | 8.1725 | 8.8276 |
| 201 | bta-miR-16b | UAGCAGCACGUAAAUAUUGGC | 434 | 685 | 568 | 21 | 132.4126 | 164.6523 | 172.8989 |
| 202 | bta-miR-181a | AACAUUCAACGCUGUCGGUGAGUU | 539 | 1112 | 919 | 24 | 143.8920 | 233.8784 | 244.7752 |
| 203 | bta-miR-181b | AACAUUCAUUGCUGUCGGUGGGUU | 371 | 354 | 400 | 24 | 99.0425 | 74.4541 | 106.5398 |
| 204 | bta-miR-181c | AACAUUCAACCUGUCGGUGAGUUU | 0 | 2 | 1 | 24 | 0.0000 | 0.4206 | 0.2663 |
| 205 | bta-miR-181d | AACAUUCAUUGUUGUCGGUGGGU | 56 | 114 | 78 | 23 | 15.5998 | 25.0192 | 21.6785 |
| 206 | bta-miR-182 | UUUGGCAAUGGUAGAACUCACACU | 14 | 6 | 18 | 24 | 3.7375 | 1.2619 | 4.7943 |
| 207 | bta-miR-183 | UAUGGCACUGGUAGAAUUCACUG | 3 | 5 | 13 | 23 | 0.8357 | 1.0973 | 3.6131 |
| 208 | bta-miR-1839 | AAGGUAGAUAGAACAGGUCUUGUU | 81 | 179 | 127 | 24 | 21.6238 | 37.6477 | 33.8264 |
| 209 | bta-miR-185 | UGGAGAGAAAGGCAGUUCCUGA | 728 | 1610 | 758 | 22 | 212.0156 | 369.4024 | 220.2468 |
| 210 | bta-miR-186 | CAAAGAAUUCUCCUUUUGGGCU | 1702 | 2968 | 1903 | 22 | 495.6737 | 680.9852 | 552.9416 |
| 211 | bta-miR-187 | UCGUGUCUUGUGUUGCAGCCGG | 5 | 23 | 5 | 22 | 1.4562 | 5.2772 | 1.4528 |
| 212 | bta-miR-188 | CAUCCCUUGCAUGGUGGAGGGU | 1 | 8 | 5 | 22 | 0.2912 | 1.8355 | 1.4528 |
| 213 | bta-miR-190a | UGAUAUGUUUGAUAUAUUAGGU | 8 | 10 | 3 | 22 | 2.3298 | 2.2944 | 0.8717 |
| 214 | bta-miR-192 | CUGACCUAUGAAUUGACAGCCAG | 202 | 278 | 215 | 23 | 56.2707 | 61.0117 | 59.7549 |
| 215 | bta-miR-193a-3p | AACUGGCCUACAAAGUCCCAGU | 3 | 2 | 2 | 22 | 0.8737 | 0.4589 | 0.5811 |
| 216 | bta-miR-193a-5p | UGGGUCUUUGCGGGCGAGAUGA | 58 | 108 | 81 | 22 | 16.8913 | 24.7798 | 23.5356 |
| 217 | bta-miR-193b | AACUGGCCCACAAAGUCCCGCUUU | 115 | 187 | 123 | 24 | 30.7005 | 39.3303 | 32.7610 |
| 218 | bta-miR-195 | UAGCAGCACAGAAAUAUUGGCA | 764 | 1312 | 988 | 22 | 222.4998 | 301.0285 | 287.0764 |
| 219 | bta-miR-199a-3p | ACAGUAGUCUGCACAUUGGUUA | 9174 | 29197 | 17162 | 22 | 2671.7455 | 6699.0318 | 4986.6442 |
| 220 | bta-miR-199a-5p | CCCAGUGUUCAGACUACCUGUU | 9830 | 17876 | 11865 | 22 | 2862.7925 | 4101.5136 | 3447.5314 |
| 221 | bta-miR-199b | CCCAGUGUUUAGACUAUCUGUUC | 15746 | 24978 | 17201 | 23 | 4386.3314 | 5481.8395 | 4780.6729 |
| 222 | bta-miR-199c | UACAGUAGUCUGCACAUUGG | 24 | 130 | 66 | 20 | 7.6885 | 32.8103 | 21.0949 |
| 223 | bta-miR-200a | UAACACUGUCUGGUAACGAUGUU | 9 | 4 | 24 | 23 | 2.5071 | 0.8779 | 6.6703 |
| 224 | bta-miR-200b | UAAUACUGCCUGGUAAUGAUG | 8 | 3 | 20 | 21 | 2.4408 | 0.7211 | 6.0880 |
| 225 | bta-miR-202 | UUCCUAUGCAUAUACUUCUUU | 0 | 1 | 0 | 21 | 0.0000 | 0.2404 | 0.0000 |
| 226 | bta-miR-206 | UGGAAUGUAAGGAAGUGUGUGG | 0 | 1 | 1 | 22 | 0.0000 | 0.2294 | 0.2906 |
| 227 | bta-miR-20a | UAAAGUGCUUAUAGUGCAGGUAG | 2313 | 1762 | 1341 | 23 | 644.3277 | 386.7003 | 372.7040 |
| 228 | bta-miR-20b | CAAAGUGCUCACAGUGCAGGUA | 10 | 9 | 8 | 22 | 2.9123 | 2.0650 | 2.3245 |
| 229 | bta-miR-210 | ACUGUGCGUGUGACAGCGGCUGA | 9 | 20 | 21 | 23 | 2.5071 | 4.3893 | 5.8365 |
| 230 | bta-miR-212 | ACCUUGGCUCUAGACUGCUUACU | 1 | 1 | 0 | 23 | 0.2786 | 0.2195 | 0.0000 |
| 231 | bta-miR-215 | AUGACCUAUGAAUUGACAGACA | 4 | 18 | 22 | 22 | 1.1649 | 4.1300 | 6.3924 |
| 232 | bta-miR-21-5p | UAGCUUAUCAGACUGAUGUUGACU | 215228 | 198752 | 164868 | 24 | 57457.4778 | 41801.9704 | 43912.5134 |
| 233 | bta-miR-222 | AGCUACAUCUGGCUACUGGGU | 223 | 179 | 178 | 21 | 68.0369 | 43.0259 | 54.1831 |
| 234 | bta-miR-223 | UGUCAGUUUGUCAAAUACCCCA | 3 | 8 | 6 | 22 | 0.8737 | 1.8355 | 1.7434 |
| 235 | bta-miR-22-3p | AAGCUGCCAGUUGAAGAACUG | 441 | 1247 | 807 | 21 | 134.5483 | 299.7393 | 245.6504 |
| 236 | bta-miR-224 | CAAGUCACUAGUGGUUCCGUUUA | 1432 | 1335 | 944 | 23 | 398.9093 | 292.9881 | 262.3659 |
| 237 | bta-miR-22-5p | AGUUCUUCAGUGGCAAGCUUUA | 54 | 130 | 112 | 22 | 15.7264 | 29.8275 | 32.5431 |
| 238 | bta-miR-2284ab | UAAAAGUUUGGUUGGGUUUUU | 28 | 35 | 31 | 21 | 8.5428 | 8.4129 | 9.4364 |
| 239 | bta-miR-2284x | UGAAAAGUUCGUUCGGGUUUU | 2072 | 3985 | 3183 | 21 | 632.1636 | 957.8677 | 968.9035 |
| 240 | bta-miR-2284y | AAAAGUUCGUUCGGGUUUUUC | 8 | 17 | 12 | 21 | 2.4408 | 4.0863 | 3.6528 |
| 241 | bta-miR-2285ab | AAAACCUGAAUGAACUUCUUGG | 3 | 3 | 3 | 22 | 0.8737 | 0.6883 | 0.8717 |
| 242 | bta-miR-2285b | AAAAUCUGAGUGAACUUUUUGG | 7 | 3 | 9 | 22 | 2.0386 | 0.6883 | 2.6151 |
| 243 | bta-miR-2285c | AAACCUGAACAAACUUUUUGGC | 1 | 5 | 6 | 22 | 0.2912 | 1.1472 | 1.7434 |
| 244 | bta-miR-2285f | AAAACCUGAAUGAACUUUUUGG | 118 | 114 | 95 | 22 | 34.3652 | 26.1564 | 27.6035 |
| 245 | bta-miR-2285k | AAAACCGGAAUGAACUUUUUG | 1 | 1 | 0 | 21 | 0.3051 | 0.2404 | 0.0000 |
| 246 | bta-miR-2285l | AAAACCCGCAUGAACUUUUUGGC | 857 | 609 | 763 | 23 | 238.7328 | 133.6552 | 212.0605 |
| 247 | bta-miR-2314 | UGCCCAUGAUGACUGCUGACC | 3 | 2 | 0 | 21 | 0.9153 | 0.4807 | 0.0000 |
| 248 | bta-miR-2331-3p | ACCCUGCAGCCAAAGAAGCUA | 2 | 4 | 1 | 21 | 0.6102 | 0.9615 | 0.3044 |
| 249 | bta-miR-2332 | CGGUUUAAGGUCUUGGAGACAAAG | 14 | 12 | 38 | 24 | 3.7375 | 2.5239 | 10.1213 |
| 250 | bta-miR-2355-3p | AUUGUCCUUGCUGUUUGGAGAU | 12 | 24 | 25 | 22 | 3.4948 | 5.5066 | 7.2641 |
| 251 | bta-miR-2366 | UGGGUCACAGAAGAGGGUCUGG | 0 | 1 | 0 | 22 | 0.0000 | 0.2294 | 0.0000 |
| 252 | bta-miR-23a | AUCACAUUGCCAGGGAUUUCCA | 1829 | 2815 | 3054 | 22 | 532.6600 | 645.8805 | 887.3798 |
| 253 | bta-miR-23b-3p | AUCACAUUGCCAGGGAUUACCAC | 887 | 1685 | 1431 | 23 | 247.0898 | 369.8014 | 397.7177 |
| 254 | bta-miR-24 | GUGCCUACUGAGCUGAUAUCAGU | 15 | 25 | 25 | 23 | 4.1785 | 5.4867 | 6.9482 |
| 255 | bta-miR-2419-5p | AUCGCAUCAACACUCGUCUGUU | 114 | 402 | 227 | 22 | 33.2002 | 92.2359 | 65.9578 |
| 256 | bta-miR-2447 | UCUGGGAACCGGUUUGGCUGCU | 2 | 4 | 3 | 22 | 0.5825 | 0.9178 | 0.8717 |
| 257 | bta-miR-2448-3p | GUGGUUGAUUGGAUCCGUGGGU | 0 | 1 | 1 | 22 | 0.0000 | 0.2294 | 0.2906 |
| 258 | bta-miR-26a | UUCAAGUAAUCCAGGAUAGGCU | 11476 | 15770 | 14591 | 22 | 3342.1573 | 3618.3077 | 4239.6064 |
| 259 | bta-miR-26b | UUCAAGUAAUUCAGGAUAGGUU | 1511 | 2641 | 2964 | 22 | 440.0488 | 605.9576 | 861.2291 |
| 260 | bta-miR-27a-3p | UUCACAGUGGCUAAGUUCCG | 943 | 2446 | 1983 | 20 | 302.0930 | 617.3379 | 633.8053 |
| 261 | bta-miR-27a-5p | AGGGCUUAGCUGCUUGUGAGCA | 188 | 114 | 236 | 22 | 54.7513 | 26.1564 | 68.5729 |
| 262 | bta-miR-27b | UUCACAGUGGCUAAGUUCUGC | 25840 | 53525 | 41660 | 21 | 7883.7391 | 12865.7133 | 12681.2813 |
| 263 | bta-miR-296-3p | GAGGGUUGGGCGGAGGCUUUCC | 91 | 97 | 94 | 22 | 26.5019 | 22.2559 | 27.3129 |
| 264 | bta-miR-299 | UGGUUUACCGUCCCACAUACAU | 7 | 22 | 11 | 22 | 2.0386 | 5.0477 | 3.1962 |
| 265 | bta-miR-29a | CUAGCACCAUCUGAAAUCGGUUA | 175 | 253 | 170 | 23 | 48.7494 | 55.5251 | 47.2481 |
| 266 | bta-miR-29d-5p | UGACCGAUUUCUCCUGGUGUU | 9 | 12 | 9 | 21 | 2.7459 | 2.8844 | 2.7396 |
| 267 | bta-miR-301a | CAGUGCAAUAGUAUUGUCAAAGCAU | 2 | 3 | 0 | 25 | 0.5126 | 0.6057 | 0.0000 |
| 268 | bta-miR-30a-5p | UGUAAACAUCCUCGACUGGAAGCU | 687 | 1994 | 1373 | 24 | 183.4022 | 419.3826 | 365.6979 |
| 269 | bta-miR-30c | UGUAAACAUCCUACACUCUCAGC | 411 | 524 | 349 | 23 | 114.4914 | 115.0006 | 96.9975 |
| 270 | bta-miR-30d | UGUAAACAUCCCCGACUGGAAGCU | 2411 | 2848 | 2459 | 24 | 643.6429 | 598.9978 | 654.9535 |
| 271 | bta-miR-30e-5p | UGUAAACAUCCUUGACUGGAAGCU | 1021 | 1050 | 788 | 24 | 272.5672 | 220.8384 | 209.8834 |
| 272 | bta-miR-30f | UGUAAACACCCUACACUCUCAGCU | 70 | 155 | 110 | 24 | 18.6873 | 32.6000 | 29.2984 |
| 273 | bta-miR-3154 | CAGAAGGGGAGUCGGAGCAGA | 1 | 0 | 0 | 21 | 0.3051 | 0.0000 | 0.0000 |
| 274 | bta-miR-320a | AAAAGCUGGGUUGAGAGGGCGA | 524 | 573 | 343 | 22 | 152.6046 | 131.4705 | 99.6631 |
| 275 | bta-miR-323 | GCACAUUACACGGUCGACCUCU | 56 | 124 | 81 | 22 | 16.3089 | 28.4509 | 23.5356 |
| 276 | bta-miR-324 | CGCAUCCCCUAGGGCAUUGGUGU | 4 | 10 | 9 | 23 | 1.1143 | 2.1947 | 2.5014 |
| 277 | bta-miR-326 | CCUCUGGGCCCUUCCUCCAG | 0 | 9 | 5 | 20 | 0.0000 | 2.2715 | 1.5981 |
| 278 | bta-miR-328 | CUGGCCCUCUCUGCCCUUCCGU | 92 | 217 | 119 | 22 | 26.7932 | 49.7890 | 34.5770 |
| 279 | bta-miR-335 | UCAAGAGCAAUAACGAAAAAUGU | 123 | 188 | 146 | 23 | 34.2639 | 41.2597 | 40.5778 |
| 280 | bta-miR-338 | UCCAGCAUCAGUGAUUUUGUUGA | 1 | 5 | 2 | 23 | 0.2786 | 1.0973 | 0.5559 |
| 281 | bta-miR-339a | UCCCUGUCCUCCAGGAGCUCAC | 324 | 732 | 500 | 22 | 94.3586 | 167.9519 | 145.2816 |
| 282 | bta-miR-339b | UCCCUGUCCUCCAGGAGCUC | 14 | 72 | 46 | 20 | 4.4849 | 18.1718 | 14.7025 |
| 283 | bta-miR-342 | UCUCACACAGAAAUCGCACCCAUCU | 1061 | 839 | 556 | 25 | 271.9158 | 169.4020 | 142.1667 |
| 284 | bta-miR-3431 | CCUCAGUCAGCCUUGUGGAUGU | 1076 | 1200 | 998 | 22 | 313.3637 | 275.3310 | 289.9820 |
| 285 | bta-miR-345-3p | CCUGAACUAGGGGUCUGGAG | 6 | 13 | 6 | 20 | 1.9221 | 3.2810 | 1.9177 |
| 286 | bta-miR-345-5p | GCUGACUCCUAGUCCAGUGCU | 5 | 8 | 4 | 21 | 1.5255 | 1.9229 | 1.2176 |
| 287 | bta-miR-361 | UUAUCAGAAUCUCCAGGGGUAC | 47 | 99 | 90 | 22 | 13.6878 | 22.7148 | 26.1507 |
| 288 | bta-miR-362-3p | AACACACCUAUUCAAGGAUUC | 2 | 12 | 6 | 21 | 0.6102 | 2.8844 | 1.8264 |
| 289 | bta-miR-362-5p | AAUCCUUGGAACCUAGGUGUGAGU | 110 | 132 | 113 | 24 | 29.3657 | 27.7625 | 30.0975 |
| 290 | bta-miR-363 | AUUGCACGGUAUCCAUCUGCG | 0 | 3 | 6 | 21 | 0.0000 | 0.7211 | 1.8264 |
| 291 | bta-miR-365-5p | AGGGACUUUUGGGGGCAGAUGUG | 89 | 41 | 55 | 23 | 24.7926 | 8.9981 | 15.2861 |
| 292 | bta-miR-369-3p | AAUAAUACAUGGUUGAUCUUU | 413 | 946 | 557 | 21 | 126.0056 | 227.3884 | 169.5505 |
| 293 | bta-miR-370 | GCCUGCUGGGGUGGAACCUGGU | 144 | 217 | 123 | 22 | 41.9371 | 49.7890 | 35.7393 |
| 294 | bta-miR-374a | UUAUAAUACAACCUGAUAAGUG | 386 | 686 | 535 | 22 | 112.4148 | 157.3975 | 155.4513 |
| 295 | bta-miR-374b | AUAUAAUACAACCUGCUAAGUG | 236 | 539 | 359 | 22 | 68.7303 | 123.6695 | 104.3122 |
| 296 | bta-miR-376a | AUCAUAGAGGAAAAUCCACGU | 3 | 9 | 8 | 21 | 0.9153 | 2.1633 | 2.4352 |
| 297 | bta-miR-376b | AUCAUAGAGGAAAAUCCAUGUU | 23 | 28 | 16 | 22 | 6.6983 | 6.4244 | 4.6490 |
| 298 | bta-miR-376c | GUGGAUAUUCCUUCUAUGUUUA | 16 | 16 | 16 | 22 | 4.6597 | 3.6711 | 4.6490 |
| 299 | bta-miR-376d | AUCAUAGAGGAAAAUCCACAU | 102 | 101 | 56 | 21 | 31.1200 | 24.2772 | 17.0464 |
| 300 | bta-miR-376e | AACAUAGAGGAAAAUCCACAUU | 479 | 496 | 359 | 22 | 139.4992 | 113.8035 | 104.3122 |
| 301 | bta-miR-377 | AUCACACAAAGGCAACUUUUGU | 3 | 1 | 1 | 22 | 0.8737 | 0.2294 | 0.2906 |
| 302 | bta-miR-378 | ACUGGACUUGGAGUCAGAAGGC | 2528 | 2208 | 1869 | 22 | 736.2298 | 506.6090 | 543.0625 |
| 303 | bta-miR-379 | UGGUAGACUAUGGAACGUAGG | 1502 | 3615 | 2232 | 21 | 458.2576 | 868.9314 | 679.4196 |
| 304 | bta-miR-380-3p | UAUGUAAUGUGGUCCACGUCU | 207 | 610 | 310 | 21 | 63.1553 | 146.6247 | 94.3638 |
| 305 | bta-miR-381 | UAUACAAGGGCAAGCUCUCUGU | 422 | 494 | 386 | 22 | 122.8991 | 113.3446 | 112.1574 |
| 306 | bta-miR-382 | GAAGUUGUUCGUGGUGGAUUCG | 242 | 622 | 284 | 22 | 70.4777 | 142.7132 | 82.5199 |
| 307 | bta-miR-409a | AGGUUACCCGAGCAACUUUGCAU | 213 | 350 | 204 | 23 | 59.3350 | 76.8133 | 56.6977 |
| 308 | bta-miR-410 | AAUAUAACACAGAUGGCCUGU | 44 | 69 | 41 | 21 | 13.4243 | 16.5854 | 12.4804 |
| 309 | bta-miR-411a | AUAGUAGACCGUAUAGCGUACG | 1426 | 3581 | 1992 | 22 | 415.2942 | 821.6335 | 578.8017 |
| 310 | bta-miR-411b | UGGUCGACCAUAAAACGUACGU | 137 | 216 | 121 | 22 | 39.8985 | 49.5596 | 35.1581 |
| 311 | bta-miR-411c-3p | UGUAUGUCAACUGAUCCACAGU | 134 | 397 | 203 | 22 | 39.0248 | 91.0887 | 58.9843 |
| 312 | bta-miR-411c-5p | GGUUGAUCAGAGAACAUACAUU | 1269 | 1929 | 1434 | 22 | 369.5711 | 442.5945 | 416.6675 |
| 313 | bta-miR-421 | AUCAACAGACAUUAAUUGGGCGC | 16 | 24 | 16 | 23 | 4.4571 | 5.2672 | 4.4469 |
| 314 | bta-miR-423-3p | AAGCUCGGUCUGAGGCCCCUCAGU | 1401 | 1537 | 1059 | 24 | 374.0123 | 323.2653 | 282.0641 |
| 315 | bta-miR-423-5p | UGAGGGGCAGAGAGCGAGACUUU | 866 | 1016 | 719 | 23 | 241.2399 | 222.9782 | 199.8316 |
| 316 | bta-miR-424-3p | CAAAACGUGAGGCGCUGCUAU | 202 | 285 | 153 | 21 | 61.6298 | 68.5050 | 46.5731 |
| 317 | bta-miR-424-5p | CAGCAGCAAUUCAUGUUUUGA | 425 | 1156 | 478 | 21 | 129.6668 | 277.8658 | 145.5029 |
| 318 | bta-miR-425-3p | AUCGGGAAUGUCGUGUCCGCCC | 5 | 8 | 4 | 22 | 1.4562 | 1.8355 | 1.1623 |
| 319 | bta-miR-425-5p | AUGACACGAUCACUCCCGUUGA | 200 | 177 | 146 | 22 | 58.2460 | 40.6113 | 42.4222 |
| 320 | bta-miR-431 | UGUCUUGCAGGCCGUCAUGCAGG | 70 | 231 | 123 | 23 | 19.4998 | 50.6968 | 34.1854 |
| 321 | bta-miR-432 | UCUUGGAGUAGGUCAUUGGGUGG | 54 | 119 | 70 | 23 | 15.0427 | 26.1165 | 19.4551 |
| 322 | bta-miR-433 | AUCAUGAUGGGCUCCUCGGUGU | 79 | 176 | 92 | 22 | 23.0072 | 40.3819 | 26.7318 |
| 323 | bta-miR-449a | UGGCAGUGUAUUGUUAGCUGGU | 54 | 74 | 55 | 22 | 15.7264 | 16.9787 | 15.9810 |
| 324 | bta-miR-449b | AGGCAGUGUAUUGUUAGCUGGC | 1 | 1 | 2 | 22 | 0.2912 | 0.2294 | 0.5811 |
| 325 | bta-miR-451 | AAACCGUUACCAUUACUGAGUUU | 3357 | 22232 | 8535 | 23 | 935.1527 | 4879.1839 | 2372.1320 |
| 326 | bta-miR-452 | UGUUUGCAGAGGAAACUGAGAC | 862 | 781 | 569 | 22 | 251.0404 | 179.1946 | 165.3304 |
| 327 | bta-miR-483 | UCACUCCUCUCCUCCCGUCUU | 3 | 15 | 5 | 21 | 0.9153 | 3.6055 | 1.5220 |
| 328 | bta-miR-485 | AGAGGCUGGCCGUGAUGAAUUCG | 8 | 14 | 7 | 23 | 2.2285 | 3.0725 | 1.9455 |
| 329 | bta-miR-486 | UCCUGUACUGAGCUGCCCCGAG | 95 | 814 | 256 | 22 | 27.6669 | 186.7662 | 74.3842 |
| 330 | bta-miR-487b | AAUCGUACAGGGUCAUCCACUU | 28 | 68 | 38 | 22 | 8.1544 | 15.6021 | 11.0414 |
| 331 | bta-miR-490 | CAACCUGGAGGACUCCAUGCUG | 0 | 3 | 0 | 22 | 0.0000 | 0.6883 | 0.0000 |
| 332 | bta-miR-491 | AGUGGGGAACCCUUCCAUGAGG | 4 | 15 | 6 | 22 | 1.1649 | 3.4416 | 1.7434 |
| 333 | bta-miR-493 | UGAAGGUCUACUGUGUGCCAGG | 1089 | 1658 | 853 | 22 | 317.1496 | 380.4156 | 247.8503 |
| 334 | bta-miR-494 | UGAAACAUACACGGGAAACCUC | 1850 | 1939 | 1559 | 22 | 538.7758 | 444.8889 | 452.9879 |
| 335 | bta-miR-495 | AAACAAACAUGGUGCACUUCUU | 353 | 770 | 441 | 22 | 102.8042 | 176.6707 | 128.1383 |
| 336 | bta-miR-496 | UGAGUAUUACAUGGCCAAUCUC | 1 | 2 | 1 | 22 | 0.2912 | 0.4589 | 0.2906 |
| 337 | bta-miR-497 | CAGCAGCACACUGUGGUUUGUA | 160 | 317 | 197 | 22 | 46.5968 | 72.7333 | 57.2409 |
| 338 | bta-miR-499 | UUAAGACUUGCAGUGAUGUUU | 8 | 12 | 6 | 21 | 2.4408 | 2.8844 | 1.8264 |
| 339 | bta-miR-500 | UAAUCCUUGCUACCUGGGUGAGA | 98 | 80 | 64 | 23 | 27.2997 | 17.5573 | 17.7875 |
| 340 | bta-miR-503-3p | GGAGUAUUGUUUCUGCUGCCCGG | 23 | 44 | 16 | 23 | 6.4071 | 9.6565 | 4.4469 |
| 341 | bta-miR-503-5p | UAGCAGCGGGAACAGUACUG | 328 | 504 | 237 | 20 | 105.0758 | 127.2029 | 75.7498 |
| 342 | bta-miR-504 | AGACCCUGGUCUGCACUCUGUC | 1 | 16 | 2 | 22 | 0.2912 | 3.6711 | 0.5811 |
| 343 | bta-miR-505 | CGUCAACACUUGCUGGUUUCCU | 39 | 36 | 45 | 22 | 11.3580 | 8.2599 | 13.0753 |
| 344 | bta-miR-532 | CAUGCCUUGAGUGUAGGACCGU | 861 | 1416 | 1235 | 22 | 250.7492 | 324.8905 | 358.8454 |
| 345 | bta-miR-539 | GGAGAAAUUAUCCUUGGUGUGU | 6 | 2 | 6 | 22 | 1.7474 | 0.4589 | 1.7434 |
| 346 | bta-miR-542-5p | UCGGGGAUCAUCAUGUCACGAG | 5 | 13 | 9 | 22 | 1.4562 | 2.9828 | 2.6151 |
| 347 | bta-miR-543 | AAACAUUCGCGGUGCACUUCUU | 209 | 388 | 207 | 22 | 60.8671 | 89.0237 | 60.1466 |
| 348 | bta-miR-544a | AUUCUGCAUUUUUAGCAAGUUC | 0 | 1 | 0 | 22 | 0.0000 | 0.2294 | 0.0000 |
| 349 | bta-miR-582 | UUACAGUUGUUCAACCAGUUACU | 2 | 7 | 1 | 23 | 0.5571 | 1.5363 | 0.2779 |
| 350 | bta-miR-6123 | UGCCAAGCCCACGUUCAAAGG | 17 | 18 | 20 | 21 | 5.1867 | 4.3266 | 6.0880 |
| 351 | bta-miR-628 | AUGCUGACAUAUUUACUAGAGG | 1 | 6 | 3 | 22 | 0.2912 | 1.3767 | 0.8717 |
| 352 | bta-miR-652 | AAUGGCGCCACUAGGGUUGUG | 18 | 33 | 45 | 21 | 5.4918 | 7.9322 | 13.6980 |
| 353 | bta-miR-6529a | GAGAGAUCAGAGGCGCAGAGU | 57 | 115 | 79 | 21 | 17.3906 | 27.6424 | 24.0476 |
| 354 | bta-miR-654 | UAUGUCUGCUGACCAUCACCUU | 174 | 332 | 260 | 22 | 50.6740 | 76.1749 | 75.5464 |
| 355 | bta-miR-655 | AUAAUACAUGGUUAACCUCUCU | 183 | 280 | 202 | 22 | 53.2951 | 64.2439 | 58.6937 |
| 356 | bta-miR-656 | AAUAUUAUACAGUCAACCUCU | 84 | 111 | 87 | 21 | 25.6283 | 26.6809 | 26.4828 |
| 357 | bta-miR-660 | UACCCAUUGCAUAUCGGAGCUG | 1134 | 1703 | 1339 | 22 | 330.2550 | 390.7405 | 389.0640 |
| 358 | bta-miR-665 | ACCAGUAGGCCGAGGCCCCU | 35 | 78 | 72 | 20 | 11.2124 | 19.6862 | 23.0126 |
| 359 | bta-miR-671 | AGGAAGCCCUGGAGGGGCUGGAG | 63 | 80 | 36 | 23 | 17.5498 | 17.5573 | 10.0055 |
| 360 | bta-miR-708 | AAGGAGCUUACAAUCUAGCUGGG | 169 | 208 | 199 | 23 | 47.0780 | 45.6491 | 55.3081 |
| 361 | bta-miR-744 | UGCGGGGCUAGGGCUAACAGCA | 55 | 72 | 45 | 22 | 16.0177 | 16.5199 | 13.0753 |
| 362 | bta-miR-758 | UUUGUGACCUGGUCCACUAACC | 38 | 85 | 59 | 22 | 11.0667 | 19.5026 | 17.1432 |
| 363 | bta-miR-760-3p | CGGCUCUGGGUCUGUGGGGA | 5 | 6 | 2 | 20 | 1.6018 | 1.5143 | 0.6392 |
| 364 | bta-miR-767 | UGCACCAUGGUUGUCUGAGCAUG | 0 | 1 | 0 | 23 | 0.0000 | 0.2195 | 0.0000 |
| 365 | bta-miR-769 | UGAGACCUCCGGGUUCUGAGCU | 71 | 82 | 86 | 22 | 20.6773 | 18.8143 | 24.9884 |
| 366 | bta-miR-7857 | AUAGCCAGUUGGGGAAGAAUGC | 34 | 40 | 41 | 22 | 9.9018 | 9.1777 | 11.9131 |
| 367 | bta-miR-7859 | AAAAACUGGCAGCUUCAUGUAA | 23 | 32 | 31 | 22 | 6.6983 | 7.3422 | 9.0075 |
| 368 | bta-miR-874 | CUGCCCUGGCCCGAGGGACCGA | 14 | 27 | 19 | 22 | 4.0772 | 6.1949 | 5.5207 |
| 369 | bta-miR-885 | UCCAUUACACUACCCUGCCUCU | 0 | 5 | 0 | 22 | 0.0000 | 1.1472 | 0.0000 |
| 370 | bta-miR-92b | UAUUGCACUCGUCCCGGCCUCC | 31 | 30 | 17 | 22 | 9.0281 | 6.8833 | 4.9396 |
| 371 | bta-miR-93 | CAAAGUGCUGUUCGUGCAGGUA | 563 | 567 | 424 | 22 | 163.9626 | 130.0939 | 123.1988 |
| 372 | bta-miR-95 | UUCAACGGGUAUUUAUUGAGCA | 3 | 28 | 27 | 22 | 0.8737 | 6.4244 | 7.8452 |
| 373 | bta-miR-99a-5p | AACCCGUAGAUCCGAUCUUGU | 13211 | 49497 | 22479 | 21 | 4030.6531 | 11897.5098 | 6842.5954 |
| 374 | bta-miR-99b | CACCCGUAGAACCGACCUUGCG | 969 | 1456 | 1148 | 22 | 282.2020 | 334.0682 | 333.5665 |
| 375 | ccr-let-7i | UGAGGUAGUAGUUUGUGCUGU | 65670 | 114404 | 74011 | 21 | 20035.8029 | 27499.0951 | 22528.9082 |
| 376 | ccr-miR-100 | AACCCGUAGAUCCGAACUUGU | 4072 | 10780 | 8648 | 21 | 1242.3601 | 2591.1703 | 2632.4465 |
| 377 | ccr-miR-126-3p | CUCGUACCGUGAGUAAUAAUGC | 3151 | 5557 | 4699 | 22 | 917.6662 | 1275.0118 | 1365.3561 |
| 378 | ccr-miR-133a-5p | AGCUGGUAAAAUGGAACCAAA | 0 | 1 | 3 | 21 | 0.0000 | 0.2404 | 0.9132 |
| 379 | ccr-miR-140-5p | CAGUGGUUUUACCCUAUGGUAG | 1176 | 849 | 788 | 22 | 342.4867 | 194.7967 | 228.9637 |
| 380 | ccr-miR-143 | UGAGAUGAAGCACUGUAGCU | 13094 | 29497 | 21748 | 20 | 4194.7045 | 7444.6510 | 6951.0833 |
| 381 | ccr-miR-144 | CUACAGUAUAGAUGAUGUACU | 4 | 51 | 15 | 21 | 1.2204 | 12.2588 | 4.5660 |
| 382 | ccr-miR-181a | AACAUUCAACGCUGUCGGUGA | 538 | 1112 | 918 | 21 | 164.1429 | 267.2896 | 279.4387 |
| 383 | ccr-miR-181b | AACAUUCAUUGCUGUCGGUGG | 364 | 350 | 395 | 21 | 111.0558 | 84.1289 | 120.2378 |
| 384 | ccr-miR-182-5p | UUUGGCAAUGGUAGAACUCACAC | 14 | 6 | 18 | 23 | 3.9000 | 1.3168 | 5.0027 |
| 385 | ccr-miR-199-3p | ACAGUAGUCUGCACAUUGGUU | 9197 | 29227 | 17182 | 21 | 2805.9887 | 7025.2443 | 5230.1915 |
| 386 | ccr-miR-199-5p | CCCAGUGUUCAGACUACCUGUUC | 9829 | 17874 | 11862 | 23 | 2738.0447 | 3922.7480 | 3296.8049 |
| 387 | ccr-miR-200a | UAACACUGUCUGGUAACGAUG | 9 | 4 | 24 | 21 | 2.7459 | 0.9615 | 7.3056 |
| 388 | ccr-miR-214 | UACAGCAGGCACAGACAGG | 148 | 394 | 259 | 19 | 49.9077 | 104.6741 | 87.1383 |
| 389 | ccr-miR-221 | AGCUACAUUGUCUGCUGGG | 146 | 206 | 175 | 19 | 49.2332 | 54.7281 | 58.8773 |
| 390 | ccr-miR-222 | AGCUACAUCUGGCUACUGGG | 222 | 179 | 177 | 20 | 71.1184 | 45.1772 | 56.5726 |
| 391 | ccr-miR-30d | UGUAAACAUCCCCGACUGGAAGC | 2411 | 2848 | 2458 | 23 | 671.6274 | 625.0412 | 683.1518 |
| 392 | cfa-let-7e | UGAGGUAGGAGGUUGUAUAGUU | 670 | 1439 | 1065 | 22 | 195.1242 | 330.1677 | 309.4497 |
| 393 | cfa-miR-107 | AGCAGCAUUGUACAGGGCUAU | 40 | 81 | 46 | 21 | 12.2039 | 19.4698 | 14.0024 |
| 394 | cfa-miR-10a | UACCCUGUAGAUCCGAAUUUGU | 22 | 82 | 17 | 22 | 6.4071 | 18.8143 | 4.9396 |
| 395 | cfa-miR-125a | UCCCUGAGACCCUUUAACCUGU | 746 | 1401 | 854 | 22 | 217.2577 | 321.4489 | 248.1409 |
| 396 | cfa-miR-1296 | UUAGGGCCCUGGCUCCAUCUCCUUU | 10 | 11 | 9 | 25 | 2.5628 | 2.2210 | 2.3013 |
| 397 | cfa-miR-132 | UAACAGUCUACAGCCAUGGUCGC | 6 | 10 | 8 | 23 | 1.6714 | 2.1947 | 2.2234 |
| 398 | cfa-miR-133c | UUGGUCCCCUUCAACCAGCUG | 14 | 18 | 19 | 21 | 4.2714 | 4.3266 | 5.7836 |
| 399 | cfa-miR-134 | UGUGACUGGUUGACCAGAGGGG | 77 | 145 | 84 | 22 | 22.4247 | 33.2692 | 24.4073 |
| 400 | cfa-miR-1343 | CUCCUGGGGCCCGCACUCUCGCU | 7 | 11 | 6 | 23 | 1.9500 | 2.4141 | 1.6676 |
| 401 | cfa-miR-140 | ACCACAGGGUAGAACCACGGA | 1434 | 1608 | 1920 | 21 | 437.5109 | 386.5122 | 584.4470 |
| 402 | cfa-miR-1468 | CUCCGUUUGCCUGUUUUGCUGAU | 198 | 181 | 208 | 23 | 55.1565 | 39.7235 | 57.8094 |
| 403 | cfa-miR-146a | UGAGAACUGAAUUCCAUGGGUU | 186 | 363 | 261 | 22 | 54.1688 | 83.2876 | 75.8370 |
| 404 | cfa-miR-146b | UGAGAACUGAAUUCCAUAGGCU | 67 | 199 | 48 | 22 | 19.5124 | 45.6591 | 13.9470 |
| 405 | cfa-miR-152 | UCAGUGCAUGACAGAACUUGG | 2150 | 5391 | 4563 | 21 | 655.9613 | 1295.8255 | 1388.9747 |
| 406 | cfa-miR-15b | UAGCAGCACAUCAUGGUUUA | 113 | 169 | 113 | 20 | 36.1999 | 42.6534 | 36.1170 |
| 407 | cfa-miR-181a | AACAUUCAACGCUGUCGGUGAG | 539 | 1112 | 918 | 22 | 156.9731 | 255.1400 | 266.7369 |
| 408 | cfa-miR-181b | AACAUUCAUUGCUGUCGGUG | 364 | 350 | 395 | 20 | 116.6086 | 88.3354 | 126.2497 |
| 409 | cfa-miR-181c | AACAUUCAACCUGUCGGUGAGUU | 0 | 2 | 1 | 23 | 0.0000 | 0.4389 | 0.2779 |
| 410 | cfa-miR-1839 | AAGGUAGAUAGAACAGGUCUUG | 76 | 173 | 123 | 22 | 22.1335 | 39.6935 | 35.7393 |
| 411 | cfa-miR-1842 | UGGCUCUGCGAGGUCAGCUCA | 5 | 5 | 6 | 21 | 1.5255 | 1.2018 | 1.8264 |
| 412 | cfa-miR-191 | CAACGGAAUCCCAAAAGCAGCU | 915 | 1370 | 984 | 22 | 266.4756 | 314.3362 | 285.9141 |
| 413 | cfa-miR-192 | CUGACCUAUGAAUUGACAGCC | 199 | 276 | 211 | 21 | 60.7146 | 66.3417 | 64.2283 |
| 414 | cfa-miR-193b | CGGGGUUUUGAGGGCGAGAUGA | 6 | 8 | 5 | 22 | 1.7474 | 1.8355 | 1.4528 |
| 415 | cfa-miR-19b | UGUGCAAAUCCAUGCAAAACUG | 78 | 61 | 51 | 22 | 22.7160 | 13.9960 | 14.8187 |
| 416 | cfa-miR-200a | CAUCUUACCGGACAGUGCUGGA | 1 | 2 | 8 | 22 | 0.2912 | 0.4589 | 2.3245 |
| 417 | cfa-miR-223 | UGUCAGUUUGUCAAAUACCCC | 3 | 8 | 6 | 21 | 0.9153 | 1.9229 | 1.8264 |
| 418 | cfa-miR-224 | CAAGUCACUAGUGGUUCCGUUU | 1432 | 1335 | 944 | 22 | 417.0416 | 306.3057 | 274.2916 |
| 419 | cfa-miR-23a | AUCACAUUGCCAGGGAUUU | 7 | 53 | 43 | 19 | 2.3605 | 14.0805 | 14.4670 |
| 420 | cfa-miR-23b | AUCACAUUGCCAGGGAUUA | 4 | 25 | 24 | 19 | 1.3489 | 6.6418 | 8.0746 |
| 421 | cfa-miR-24 | UGGCUCAGUUCAGCAGGAACAGG | 753 | 1211 | 980 | 23 | 209.7617 | 265.7742 | 272.3713 |
| 422 | cfa-miR-29a | UAGCACCAUCUGAAAUCGGUUA | 169 | 250 | 169 | 22 | 49.2179 | 57.3606 | 49.1052 |
| 423 | cfa-miR-301b | CAGUGCAAUGAUAUUGUCAAAGC | 5 | 17 | 18 | 23 | 1.3928 | 3.7309 | 5.0027 |
| 424 | cfa-miR-30a | UGUAAACAUCCUCGACUGGAAGC | 687 | 1992 | 1372 | 23 | 191.3762 | 437.1777 | 381.3199 |
| 425 | cfa-miR-30c | UGUAAACAUCCUACACUCUCAGCU | 411 | 524 | 349 | 24 | 109.7210 | 110.2089 | 92.9560 |
| 426 | cfa-miR-323 | CACAUUACACGGUCGACCUCU | 54 | 122 | 80 | 21 | 16.4753 | 29.3249 | 24.3520 |
| 427 | cfa-miR-329b | AACACACCUGGUUAACCUCUUU | 10 | 32 | 13 | 22 | 2.9123 | 7.3422 | 3.7773 |
| 428 | cfa-miR-339 | UCCCUGUCCUCCAGGAGCU | 324 | 732 | 500 | 19 | 109.2573 | 194.4706 | 168.2208 |
| 429 | cfa-miR-33a | GUGCAUUGUAGUUGCAUUGC | 1 | 5 | 4 | 20 | 0.3204 | 1.2619 | 1.2785 |
| 430 | cfa-miR-345 | CCUGAACUAGGGGUCUGGAGG | 6 | 12 | 6 | 21 | 1.8306 | 2.8844 | 1.8264 |
| 431 | cfa-miR-34c | AGGCAGUGUAGUUAGCUGAUUGC | 3 | 3 | 1 | 23 | 0.8357 | 0.6584 | 0.2779 |
| 432 | cfa-miR-374a | UUAUAAUACAACCUGAUAAGU | 386 | 686 | 535 | 21 | 117.7679 | 164.8927 | 162.8537 |
| 433 | cfa-miR-375 | UUUGUUCGUUCGGCUCGCGUGA | 0 | 2 | 1 | 22 | 0.0000 | 0.4589 | 0.2906 |
| 434 | cfa-miR-377 | AGAGGUUGCCCUUGGUGAAUUC | 0 | 1 | 0 | 22 | 0.0000 | 0.2294 | 0.0000 |
| 435 | cfa-miR-382 | AAUCAUUCACGGACAACACUUU | 246 | 553 | 373 | 22 | 71.6426 | 126.8817 | 108.3800 |
| 436 | cfa-miR-3958 | CAGAUAUUGCACGGUUGAUCUCU | 570 | 979 | 634 | 23 | 158.7837 | 214.8579 | 176.2076 |
| 437 | cfa-miR-421 | AUCAACAGACAUUAAUUGGGCG | 2 | 6 | 7 | 22 | 0.5825 | 1.3767 | 2.0339 |
| 438 | cfa-miR-425 | AAUGACACGAUCACUCCCGUUGA | 200 | 177 | 146 | 23 | 55.7136 | 38.8456 | 40.5778 |
| 439 | cfa-miR-452 | AACUGUUUGCAGAGGAAACUGA | 1162 | 964 | 718 | 22 | 338.4094 | 221.1825 | 208.6243 |
| 440 | cfa-miR-454 | UAGUGCAAUAUUGCUUAUAGGG | 96 | 77 | 73 | 22 | 27.9581 | 17.6671 | 21.2111 |
| 441 | cfa-miR-486 | UCCUGUACUGAGCUGCCCCGA | 93 | 749 | 244 | 21 | 28.3741 | 180.0359 | 74.2735 |
| 442 | cfa-miR-493 | UGAAGGUCUACUGUGUGCCAG | 42 | 132 | 53 | 21 | 12.8141 | 31.7286 | 16.1332 |
| 443 | cfa-miR-497 | CAGCAGCACACUGUGGUUUGU | 160 | 317 | 197 | 21 | 48.8157 | 76.1968 | 59.9667 |
| 444 | cfa-miR-500 | AUGCACCUGGGCAAGGAUUCU | 21 | 47 | 41 | 21 | 6.4071 | 11.2973 | 12.4804 |
| 445 | cfa-miR-542 | UGUGACAGAUUGAUAACUGAAA | 976 | 2302 | 1148 | 22 | 284.2406 | 528.1766 | 333.5665 |
| 446 | cfa-miR-582 | UACAGUUGUUCAACCAGUUACU | 2 | 7 | 1 | 22 | 0.5825 | 1.6061 | 0.2906 |
| 447 | cfa-miR-590 | UAAUUUUAUGUAUAAGCUAGU | 31 | 38 | 50 | 21 | 9.4580 | 9.1340 | 15.2200 |
| 448 | cfa-miR-652 | AAUGGCGCCACUAGGGUUGUGC | 18 | 33 | 45 | 22 | 5.2421 | 7.5716 | 13.0753 |
| 449 | cfa-miR-6529 | CCUGUGCCUUUUACCUCUUUAA | 3 | 1 | 0 | 22 | 0.8737 | 0.2294 | 0.0000 |
| 450 | cfa-miR-93 | CAAAGUGCUGUUCGUGCAGGUAG | 563 | 567 | 424 | 23 | 156.8338 | 124.4376 | 117.8423 |
| 451 | cgr-let-7d-3p | CUAUACGACCUGCUGCCUUUCU | 45 | 84 | 56 | 22 | 13.1054 | 19.2732 | 16.2715 |
| 452 | cgr-let-7g-3p | CUGUACAGGCCACUGCCUUGC | 1 | 1 | 0 | 21 | 0.3051 | 0.2404 | 0.0000 |
| 453 | cgr-let-7i | CUGCGCAAGCUACUGCCUUGC | 5 | 21 | 14 | 21 | 1.5255 | 5.0477 | 4.2616 |
| 454 | cgr-miR-100-3p | CAAGCUUGUGUCUAUAGGUAUG | 1 | 3 | 6 | 22 | 0.2912 | 0.6883 | 1.7434 |
| 455 | cgr-miR-103-5p | AGCUUCUUUACAGUGCUGCCUUGU | 0 | 1 | 0 | 24 | 0.0000 | 0.2103 | 0.0000 |
| 456 | cgr-miR-106b-3p | CCGCACUGUGGGUACUUGCUGC | 224 | 364 | 261 | 22 | 65.2356 | 83.5171 | 75.8370 |
| 457 | cgr-miR-106b-5p | UAAAGUGCUGACAGUGCAGAUA | 117 | 140 | 108 | 22 | 34.0739 | 32.1219 | 31.3808 |
| 458 | cgr-miR-10b-3p | ACAGAUUCGAUUCUAGGGGAAU | 2 | 9 | 5 | 22 | 0.5825 | 2.0650 | 1.4528 |
| 459 | cgr-miR-125a-3p | ACAGGUGAGGUUCUUGGGAGC | 3 | 8 | 3 | 21 | 0.9153 | 1.9229 | 0.9132 |
| 460 | cgr-miR-125a-5p | UCCCUGAGACCCUUUAACCUGUGA | 746 | 1401 | 854 | 24 | 199.1529 | 294.6615 | 227.4625 |
| 461 | cgr-miR-125b-3p | UCACAAGUCAGGCUCUUGGGAC | 59 | 212 | 123 | 22 | 17.1826 | 48.6418 | 35.7393 |
| 462 | cgr-miR-128-5p | CGGGGCCGUAGCACUGUCUGAGA | 0 | 0 | 1 | 23 | 0.0000 | 0.0000 | 0.2779 |
| 463 | cgr-miR-1306-3p | ACGUUGGCUCUGGUGGUGAUG | 1 | 4 | 3 | 21 | 0.3051 | 0.9615 | 0.9132 |
| 464 | cgr-miR-130b-5p | ACUCUUUCCCUGUUGCACUACU | 90 | 79 | 67 | 22 | 26.2107 | 18.1260 | 19.4677 |
| 465 | cgr-miR-132-5p | ACCGUGGCUUUCGAUUGUUACU | 4 | 7 | 5 | 22 | 1.1649 | 1.6061 | 1.4528 |
| 466 | cgr-miR-139-3p | UGGAGACGCGGCCCUGUUGGAGU | 3 | 5 | 7 | 23 | 0.8357 | 1.0973 | 1.9455 |
| 467 | cgr-miR-139-5p | UCUACAGUGCACGUGUCUCCAG | 129 | 241 | 180 | 22 | 37.5687 | 55.2956 | 52.3014 |
| 468 | cgr-miR-140-3p | ACCACAGGGUAGAACCACGGAC | 10086 | 8862 | 8410 | 22 | 2937.3474 | 2033.3192 | 2443.6358 |
| 469 | cgr-miR-144 | GGAUAUCAUCAUAUACUGUAAG | 16 | 121 | 37 | 22 | 4.6597 | 27.7625 | 10.7508 |
| 470 | cgr-miR-146b-3p | GCCCUAGGGACUCAGUUCUGGU | 1 | 2 | 0 | 22 | 0.2912 | 0.4589 | 0.0000 |
| 471 | cgr-miR-146b-5p | UGAGAACUGAAUUCCAUAGGCUG | 67 | 199 | 48 | 23 | 18.6641 | 43.6739 | 13.3406 |
| 472 | cgr-miR-152-5p | AGGUUCUGUGAUACACUCCGACU | 59 | 49 | 68 | 23 | 16.4355 | 10.7539 | 18.8992 |
| 473 | cgr-miR-15a-5p | UAGCAGCACAUAAUGGUUUGUGGA | 21 | 34 | 29 | 24 | 5.6062 | 7.1510 | 7.7241 |
| 474 | cgr-miR-15b-3p | CGAAUCAUUAUUUGCUGCUCU | 19 | 21 | 18 | 21 | 5.7969 | 5.0477 | 5.4792 |
| 475 | cgr-miR-16-3p | CCAGUAUUAACUGUGCUGCUGAA | 4 | 3 | 4 | 23 | 1.1143 | 0.6584 | 1.1117 |
| 476 | cgr-miR-181a-3p | ACCAUCGACCGUUGAUUGUACC | 141 | 88 | 145 | 22 | 41.0635 | 20.1909 | 42.1317 |
| 477 | cgr-miR-181b-3p | CUCACUGAACAAUGAAUGC | 3 | 7 | 7 | 19 | 1.0116 | 1.8597 | 2.3551 |
| 478 | cgr-miR-181c-3p | ACCAUCGACCGUUGAGUGGACC | 1 | 4 | 5 | 22 | 0.2912 | 0.9178 | 1.4528 |
| 479 | cgr-miR-181c-5p | AACAUUCAACCUGUCGGUGAGU | 0 | 2 | 1 | 22 | 0.0000 | 0.4589 | 0.2906 |
| 480 | cgr-miR-183 | AUGGCACUGGUAGAAUUCACUG | 3 | 5 | 13 | 22 | 0.8737 | 1.1472 | 3.7773 |
| 481 | cgr-miR-1839-3p | AGACCUACUUAUCUACCAACAG | 10 | 11 | 10 | 22 | 2.9123 | 2.5239 | 2.9056 |
| 482 | cgr-miR-1839-5p | AAGGUAGAUAGAACAGGUCUUGU | 81 | 179 | 127 | 23 | 22.5640 | 39.2845 | 35.2971 |
| 483 | cgr-miR-186-5p | CAAAGAAUUCUCCUUUUGGGCUU | 1702 | 2968 | 1903 | 23 | 474.1227 | 651.3772 | 528.9007 |
| 484 | cgr-miR-187 | UCGUGUCUUGUGUUGCAGCCG | 5 | 23 | 5 | 21 | 1.5255 | 5.5285 | 1.5220 |
| 485 | cgr-miR-188 | CAUCCCUUGCAUGGUGGAGG | 1 | 8 | 5 | 20 | 0.3204 | 2.0191 | 1.5981 |
| 486 | cgr-miR-18a-3p | ACUGCCCUAAGUGCUCCUUCUGG | 3 | 4 | 3 | 23 | 0.8357 | 0.8779 | 0.8338 |
| 487 | cgr-miR-190a | UGAUAUGUUUGAUAUAUUAGGUUG | 8 | 10 | 3 | 24 | 2.1357 | 2.1032 | 0.7990 |
| 488 | cgr-miR-191-3p | CUGCGCUUGGAUUUCGUUCCC | 10 | 7 | 3 | 21 | 3.0510 | 1.6826 | 0.9132 |
| 489 | cgr-miR-192 | CUGACCUAUGAAUUGACAGCCA | 202 | 278 | 215 | 22 | 58.8285 | 63.7850 | 62.4711 |
| 490 | cgr-miR-193b-3p | AACUGGCCCACAAAGUCCCGCU | 115 | 187 | 123 | 22 | 33.4915 | 42.9057 | 35.7393 |
| 491 | cgr-miR-19b-5p | AGUUUUGCAGGUUUGCAUCCAGC | 1 | 1 | 2 | 23 | 0.2786 | 0.2195 | 0.5559 |
| 492 | cgr-miR-200b | UAAUACUGCCUGGUAAUGAUGAC | 36 | 13 | 92 | 23 | 10.0284 | 2.8531 | 25.5696 |
| 493 | cgr-miR-205 | UCCUUCAUUCCACCGGAGU | 16 | 12 | 44 | 19 | 5.3954 | 3.1880 | 14.8034 |
| 494 | cgr-miR-210-5p | AGCCACUGCCCACCGCACACUG | 1 | 1 | 0 | 22 | 0.2912 | 0.2294 | 0.0000 |
| 495 | cgr-miR-221-3p | AGCUACAUUGUCUGCUGGGUUUC | 146 | 206 | 175 | 23 | 40.6709 | 45.2101 | 48.6377 |
| 496 | cgr-miR-221-5p | ACCUGGCAUACAAUGUAGAUUUCUGU | 46 | 39 | 48 | 26 | 11.3356 | 7.5716 | 11.8013 |
| 497 | cgr-miR-222-3p | AGCUACAUCUGGCUACUGGGUCUCU | 223 | 179 | 178 | 25 | 57.1510 | 36.1418 | 45.5138 |
| 498 | cgr-miR-22-5p | AGUUCUUCAGUGGCAAGCUUU | 54 | 130 | 112 | 21 | 16.4753 | 31.2479 | 34.0927 |
| 499 | cgr-miR-23a-5p | GGGGUUCCUGGGGAUGGGAUUU | 1 | 0 | 0 | 22 | 0.2912 | 0.0000 | 0.0000 |
| 500 | cgr-miR-23b-5p | GGGUUCCUGGCAUGCUGAUU | 6 | 3 | 8 | 20 | 1.9221 | 0.7572 | 2.5570 |
| 501 | cgr-miR-25-5p | AGGCGGAGACUUGGGCAAUUGCU | 2 | 2 | 2 | 23 | 0.5571 | 0.4389 | 0.5559 |
| 502 | cgr-miR-26b-3p | CCUGUUCUCCAUUACUUGGCUC | 0 | 1 | 1 | 22 | 0.0000 | 0.2294 | 0.2906 |
| 503 | cgr-miR-28-5p | AAGGAGCUCACAGUCUAUUGA | 99 | 207 | 166 | 21 | 30.2047 | 49.7562 | 50.5303 |
| 504 | cgr-miR-29a-3p | UAGCACCAUCUGAAAUCGGUU | 175 | 253 | 170 | 21 | 53.3922 | 60.8132 | 51.7479 |
| 505 | cgr-miR-29a-5p | ACUGAUUUCUUUUGGUGUUCAGAG | 1 | 1 | 1 | 24 | 0.2670 | 0.2103 | 0.2663 |
| 506 | cgr-miR-29b-5p | GCUGGUUUCAUAUGGUGGUUUAGA | 2 | 0 | 0 | 24 | 0.5339 | 0.0000 | 0.0000 |
| 507 | cgr-miR-29c-5p | ACCGAUUUCUCCUGGUGUUCAGA | 9 | 12 | 9 | 23 | 2.5071 | 2.6336 | 2.5014 |
| 508 | cgr-miR-301a-5p | GCUCUGACUUUAUUGCACUACU | 13 | 9 | 8 | 22 | 3.7860 | 2.0650 | 2.3245 |
| 509 | cgr-miR-30d | CUUUCAGUCAGAUGUUUGCUGC | 0 | 1 | 1 | 22 | 0.0000 | 0.2294 | 0.2906 |
| 510 | cgr-miR-32-3p | CAAUUUAGUGUGUGUGAUAUU | 14 | 12 | 11 | 21 | 4.2714 | 2.8844 | 3.3484 |
| 511 | cgr-miR-324-5p | CGCAUCCCCUAGGGCAUUGGUG | 4 | 10 | 9 | 22 | 1.1649 | 2.2944 | 2.6151 |
| 512 | cgr-miR-32-5p | UAUUGCACAUUACUAAGUUGC | 85 | 155 | 81 | 21 | 25.9334 | 37.2571 | 24.6564 |
| 513 | cgr-miR-331-3p | GCCCCUGGGCCUAUCCUAGA | 10 | 17 | 14 | 20 | 3.2035 | 4.2906 | 4.4747 |
| 514 | cgr-miR-331-5p | CUAGGUAUGGUCCCAGGGAUC | 26 | 35 | 29 | 21 | 7.9326 | 8.4129 | 8.8276 |
| 515 | cgr-miR-339 | UCCCUGUCCUCCAGGAGCUCACG | 385 | 851 | 566 | 23 | 107.2487 | 186.7662 | 157.3083 |
| 516 | cgr-miR-362 | AAUCCUUGGAACCUAGGUGUGAGUGC | 110 | 132 | 113 | 26 | 27.1068 | 25.6270 | 27.7823 |
| 517 | cgr-miR-369-5p | AGAUCGACCGUGUUAUAUUCGC | 51 | 144 | 74 | 22 | 14.8527 | 33.0397 | 21.5017 |
| 518 | cgr-miR-374-3p | CUUAUCAGGUUGUAUUAUCAUU | 22 | 28 | 28 | 22 | 6.4071 | 6.4244 | 8.1358 |
| 519 | cgr-miR-377-3p | UGAAUCACACAAAGGCAACUUUU | 3 | 1 | 1 | 23 | 0.8357 | 0.2195 | 0.2779 |
| 520 | cgr-miR-378-5p | CUCCUGACUCCAGGUCCUGUGU | 23 | 18 | 21 | 22 | 6.6983 | 4.1300 | 6.1018 |
| 521 | cgr-miR-409-3p | GAAUGUUGCUCGGUGAACCCCU | 577 | 844 | 500 | 22 | 168.0398 | 193.6494 | 145.2816 |
| 522 | cgr-miR-409-5p | AGGUUACCCGAGCAACUUUGCAUC | 213 | 350 | 204 | 24 | 56.8627 | 73.6128 | 54.3353 |
| 523 | cgr-miR-412-3p | GUACUUCACCUGGUCCACUAGC | 5 | 4 | 2 | 22 | 1.4562 | 0.9178 | 0.5811 |
| 524 | cgr-miR-423-3p | AGCUCGGUCUGAGGCCCCUCAGU | 1401 | 1537 | 1058 | 23 | 390.2737 | 337.3203 | 294.0499 |
| 525 | cgr-miR-425-3p | CAUCGGGAAUGUCGUGUCCGC | 5 | 8 | 4 | 21 | 1.5255 | 1.9229 | 1.2176 |
| 526 | cgr-miR-505-3p | GUCAACACUUGCUGGUUUCCUCU | 39 | 36 | 45 | 23 | 10.8642 | 7.9008 | 12.5068 |
| 527 | cgr-miR-505-5p | GGGAGCCAGGAAGUAUUGAUGUU | 0 | 2 | 0 | 23 | 0.0000 | 0.4389 | 0.0000 |
| 528 | cgr-miR-542-3p | UGUGACAGAUUGAUAACUGAAAG | 993 | 2325 | 1160 | 23 | 276.6180 | 510.2601 | 322.3987 |
| 529 | cgr-miR-652-5p | CAACCCUAGGAGAGGGUGCCAUUCA | 11 | 11 | 18 | 25 | 2.8191 | 2.2210 | 4.6025 |
| 530 | cgr-miR-671-5p | AGGAAGCCCUGGAGGGGCUGGAGG | 63 | 80 | 36 | 24 | 16.8185 | 16.8258 | 9.5886 |
| 531 | cgr-miR-708 | AAGGAGCUUACAAUCUAGCUGG | 169 | 208 | 199 | 22 | 49.2179 | 47.7240 | 57.8221 |
| 532 | cgr-miR-744-3p | CUGUUGCCACUAACCUCAACC | 2 | 2 | 0 | 21 | 0.6102 | 0.4807 | 0.0000 |
| 533 | cgr-miR-744-5p | UGCGGGGCUAGGGCUAACAGC | 55 | 72 | 45 | 21 | 16.7804 | 17.3065 | 13.6980 |
| 534 | cgr-miR-874 | CUGCCCUGGCCCGAGGGACCGAC | 14 | 27 | 19 | 23 | 3.9000 | 5.9256 | 5.2807 |
| 535 | cgr-miR-92b-5p | AGGGACGGGACGCGGUGCAGUGUU | 5 | 4 | 2 | 24 | 1.3348 | 0.8413 | 0.5327 |
| 536 | cgr-miR-99a-3p | CAAGCUCGCUUCUAUGGGUCUG | 7 | 33 | 19 | 22 | 2.0386 | 7.5716 | 5.5207 |
| 537 | chi-let-7a-3p | CUAUACAAUCUACUGUCUUUCC | 131 | 142 | 131 | 22 | 38.1512 | 32.5808 | 38.0638 |
| 538 | chi-let-7d-3p | CUAUACGACCUGCUGCCUUUC | 45 | 84 | 56 | 21 | 13.7294 | 20.1909 | 17.0464 |
| 539 | chi-let-7e-3p | CUAUACGGCCUCCUAGCUUUCC | 6 | 8 | 5 | 22 | 1.7474 | 1.8355 | 1.4528 |
| 540 | chi-let-7f-3p | CUAUACAAUCUAUUGCCUUCCC | 13 | 13 | 12 | 22 | 3.7860 | 2.9828 | 3.4868 |
| 541 | chi-miR-100-3p | CAAGCUUGUGUCUAUAGGUAU | 1 | 3 | 6 | 21 | 0.3051 | 0.7211 | 1.8264 |
| 542 | chi-miR-106b-3p | CCGCACUGUGGGUACUUGCU | 224 | 364 | 261 | 20 | 71.7591 | 91.8688 | 83.4207 |
| 543 | chi-miR-1185-3p | AUAUACAGAGGGAGACUCUUAU | 34 | 41 | 24 | 22 | 9.9018 | 9.4071 | 6.9735 |
| 544 | chi-miR-1185-5p | AGAGGAUACCCUUUGUAUGU | 0 | 2 | 1 | 20 | 0.0000 | 0.5048 | 0.3196 |
| 545 | chi-miR-125b-3p | ACAAGUCAGGCUCUUGGGACC | 58 | 212 | 122 | 21 | 17.6957 | 50.9581 | 37.1367 |
| 546 | chi-miR-125b-5p | UCCCUGAGACCCUAACUUGU | 1006 | 3827 | 1919 | 20 | 322.2753 | 965.8840 | 613.3497 |
| 547 | chi-miR-126-3p | UCGUACCGUGAGUAAUAAUGC | 7951 | 12854 | 10535 | 21 | 2425.8363 | 3089.6941 | 3206.8483 |
| 548 | chi-miR-126-5p | CAUUAUUACUUUUGGUACGCGC | 65 | 131 | 102 | 22 | 18.9300 | 30.0570 | 29.6374 |
| 549 | chi-miR-1271-5p | CUUGGCACCUAGUAAGUACU | 313 | 387 | 396 | 20 | 100.2705 | 97.6737 | 126.5693 |
| 550 | chi-miR-127-3p | UCGGAUCCGUCUGAGCUUGG | 2457 | 5402 | 3868 | 20 | 787.1078 | 1363.3930 | 1236.2880 |
| 551 | chi-miR-127-5p | GAAGCUCAGAGGGCUCUGAUUC | 81 | 208 | 126 | 22 | 23.5896 | 47.7240 | 36.6110 |
| 552 | chi-miR-1306-3p | ACGUUGGCUCUGGUGGUGAUGG | 1 | 4 | 3 | 22 | 0.2912 | 0.9178 | 0.8717 |
| 553 | chi-miR-1307-3p | ACUCGGCGUGGCGUCGGUCGUGG | 245 | 234 | 209 | 23 | 68.2492 | 51.3552 | 58.0874 |
| 554 | chi-miR-133a-3p | UUUGGUCCCCUUCAACCAGCUGU | 14 | 18 | 19 | 23 | 3.9000 | 3.9504 | 5.2807 |
| 555 | chi-miR-133a-5p | AGCUGGUAAAAUGGAACCAAAU | 0 | 1 | 3 | 22 | 0.0000 | 0.2294 | 0.8717 |
| 556 | chi-miR-1343 | CUCCUGGGGCCCGCACUCUCGC | 7 | 11 | 6 | 22 | 2.0386 | 2.5239 | 1.7434 |
| 557 | chi-miR-136-3p | AUCAUCGUCUCAAAUGAGUCU | 263 | 793 | 409 | 21 | 80.2408 | 190.6121 | 124.4994 |
| 558 | chi-miR-136-5p | ACUCCAUUUGUUUUGAUGAUGG | 92 | 273 | 109 | 22 | 26.7932 | 62.6378 | 31.6714 |
| 559 | chi-miR-144-3p | UACAGUAUAGAUGAUGUAC | 4 | 51 | 15 | 19 | 1.3489 | 13.5492 | 5.0466 |
| 560 | chi-miR-144-5p | UGGGAUAUCAUCAUAUACUGU | 16 | 121 | 36 | 21 | 4.8816 | 29.0846 | 10.9584 |
| 561 | chi-miR-145-3p | AUUCCUGGAAAUACUGUUCUU | 128 | 197 | 159 | 21 | 39.0526 | 47.3526 | 48.3995 |
| 562 | chi-miR-146b-3p | UGCCCUAGGGACUCAGUUCUGGU | 1 | 2 | 0 | 23 | 0.2786 | 0.4389 | 0.0000 |
| 563 | chi-miR-148a-5p | AAAGUUCUGAGACACUCCGACU | 1822 | 1068 | 1458 | 22 | 530.6214 | 245.0446 | 423.6410 |
| 564 | chi-miR-154b-3p | AUCAUACAUGGUUGACCUUUUUU | 4 | 8 | 10 | 23 | 1.1143 | 1.7557 | 2.7793 |
| 565 | chi-miR-154b-5p | AGAGGUCUUCCAUGGUGCAUUC | 42 | 83 | 35 | 22 | 12.2317 | 19.0437 | 10.1697 |
| 566 | chi-miR-15a-5p | UAGCAGCACAUAAUGGUUUGUGG | 21 | 34 | 29 | 23 | 5.8499 | 7.4619 | 8.0600 |
| 567 | chi-miR-16b-3p | ACCAAUAUUAUUGUGCUGCUUU | 5 | 12 | 6 | 22 | 1.4562 | 2.7533 | 1.7434 |
| 568 | chi-miR-17-3p | ACUGCAGUGAAGGCACUUGUAGCA | 3 | 6 | 5 | 24 | 0.8009 | 1.2619 | 1.3317 |
| 569 | chi-miR-1814 | GUUUUGUUUGGGUUUGUU | 1 | 2 | 2 | 18 | 0.3559 | 0.5609 | 0.7103 |
| 570 | chi-miR-181b-5p | AACAUUCAUUGCUGUCGGUGGGU | 371 | 354 | 400 | 23 | 103.3487 | 77.6912 | 111.1720 |
| 571 | chi-miR-181c-5p | AACAUUCAACCUGUCGGUGAG | 0 | 2 | 1 | 21 | 0.0000 | 0.4807 | 0.3044 |
| 572 | chi-miR-187 | UCGUGUCUUGUGUUGCAGCC | 5 | 23 | 5 | 20 | 1.6018 | 5.8049 | 1.5981 |
| 573 | chi-miR-188-5p | CAUCCCUUGCAUGGUGGAGGG | 1 | 8 | 5 | 21 | 0.3051 | 1.9229 | 1.5220 |
| 574 | chi-miR-193a | AACUGGCCCACAAAGUCCC | 115 | 186 | 122 | 19 | 38.7796 | 49.4147 | 41.0459 |
| 575 | chi-miR-195-3p | CCAAUAUUGGCUGUGCUGCUC | 177 | 156 | 142 | 21 | 54.0024 | 37.4975 | 43.2247 |
| 576 | chi-miR-20b | CAAAGUGCUCACAGUGCAGGUAG | 10 | 9 | 8 | 23 | 2.7857 | 1.9752 | 2.2234 |
| 577 | chi-miR-211 | UUCCCUUUGUCAUCCUUUGCCC | 0 | 1 | 6 | 22 | 0.0000 | 0.2294 | 1.7434 |
| 578 | chi-miR-21-3p | CAACAGCAGUCGAUGGGCUGU | 9 | 7 | 7 | 21 | 2.7459 | 1.6826 | 2.1308 |
| 579 | chi-miR-214-3p | UACAGCAGGCACAGACAGGC | 970 | 1906 | 1390 | 20 | 310.7426 | 481.0491 | 444.2710 |
| 580 | chi-miR-215-5p | AUGACCUAUGAAUUGACAGAC | 5 | 18 | 22 | 21 | 1.5255 | 4.3266 | 6.6968 |
| 581 | chi-miR-21-5p | UAGCUUAUCAGACUGAUGUUGAC | 215227 | 198752 | 164866 | 23 | 59955.3504 | 43619.4474 | 45821.1972 |
| 582 | chi-miR-221-5p | ACCUGGCAUACAAUGUAGAUU | 46 | 39 | 48 | 21 | 14.0345 | 9.3744 | 14.6112 |
| 583 | chi-miR-22-3p | AAGCUGCCAGUUGAAGAAC | 441 | 1247 | 807 | 19 | 148.7113 | 331.2908 | 271.5083 |
| 584 | chi-miR-224-3p | AAAUGGUACCCUAGUGACUACA | 1 | 2 | 1 | 22 | 0.2912 | 0.4589 | 0.2906 |
| 585 | chi-miR-2331 | ACCCUGCAGCCAAAGAAGCU | 2 | 4 | 1 | 20 | 0.6407 | 1.0095 | 0.3196 |
| 586 | chi-miR-23b-5p | UGGGUUCCUGGCAUGCUGAUUU | 6 | 3 | 8 | 22 | 1.7474 | 0.6883 | 2.3245 |
| 587 | chi-miR-24-3p | UGGCUCAGUUCAGCAGGAAC | 753 | 1212 | 980 | 20 | 241.2259 | 305.8927 | 313.2270 |
| 588 | chi-miR-24-5p | GUGCCUACUGAGCUGAUAUC | 15 | 25 | 25 | 20 | 4.8053 | 6.3097 | 7.9905 |
| 589 | chi-miR-26a-3p | CCUAUUCUCGGUUACUUGCACG | 3 | 4 | 5 | 22 | 0.8737 | 0.9178 | 1.4528 |
| 590 | chi-miR-28-3p | CACUAGAUUGAGAGCUCCUGGA | 2055 | 2326 | 2208 | 22 | 598.4780 | 533.6832 | 641.5634 |
| 591 | chi-miR-296-3p | AGGGUUGGGCGGAGGCUUUCCU | 91 | 97 | 94 | 22 | 26.5019 | 22.2559 | 27.3129 |
| 592 | chi-miR-29a-5p | ACUGAUUUCUUUUGGUGUUCA | 1 | 1 | 1 | 21 | 0.3051 | 0.2404 | 0.3044 |
| 593 | chi-miR-29b-5p | CUGGUUUCACAUGGUGGCUUAGA | 0 | 1 | 1 | 23 | 0.0000 | 0.2195 | 0.2779 |
| 594 | chi-miR-29c-5p | UGACCGAUUUCUCCUGGUGUUCA | 9 | 12 | 9 | 23 | 2.5071 | 2.6336 | 2.5014 |
| 595 | chi-miR-301a-5p | GCUCUGACUUUAUUGCACUAC | 13 | 9 | 8 | 21 | 3.9663 | 2.1633 | 2.4352 |
| 596 | chi-miR-30a-3p | CUUUCAGUCGGAUGUUUGCAG | 9 | 33 | 26 | 21 | 2.7459 | 7.9322 | 7.9144 |
| 597 | chi-miR-30c-3p | CUGGGAGAGGGUUGUUUACUC | 3 | 6 | 4 | 21 | 0.9153 | 1.4422 | 1.2176 |
| 598 | chi-miR-30e-3p | CUUUCAGUCGGAUGUUUACAG | 101 | 136 | 115 | 21 | 30.8149 | 32.6901 | 35.0059 |
| 599 | chi-miR-30f-5p | UGUAAACACCCUACACUCUCAGC | 70 | 155 | 110 | 23 | 19.4998 | 34.0173 | 30.5723 |
| 600 | chi-miR-324-3p | ACUGCCCCAGGUGCUGCUGGG | 0 | 1 | 0 | 21 | 0.0000 | 0.2404 | 0.0000 |
| 601 | chi-miR-326-3p | CCUCUGGGCCCUUCCUCCAGC | 0 | 9 | 5 | 21 | 0.0000 | 2.1633 | 1.5220 |
| 602 | chi-miR-326-5p | CCUCGUCUGUCUGUUGGGCU | 0 | 1 | 1 | 20 | 0.0000 | 0.2524 | 0.3196 |
| 603 | chi-miR-329b-3p | AACACACCUGGUUAACCUCU | 10 | 32 | 13 | 20 | 3.2035 | 8.0764 | 4.1551 |
| 604 | chi-miR-331-3p | CCCCUGGGCCUAUCCUAGAAC | 10 | 17 | 14 | 21 | 3.0510 | 4.0863 | 4.2616 |
| 605 | chi-miR-331-5p | UCUAGGUAUGGUCCCAGGGAU | 26 | 35 | 29 | 21 | 7.9326 | 8.4129 | 8.8276 |
| 606 | chi-miR-335-3p | UUUUUCAUUAUUGCUCCUGACC | 203 | 243 | 205 | 22 | 59.1197 | 55.7545 | 59.5654 |
| 607 | chi-miR-335-5p | UCAAGAGCAAUAACGAAAAAU | 123 | 188 | 146 | 21 | 37.5271 | 45.1892 | 44.4423 |
| 608 | chi-miR-338-5p | AACAAUAUCCUGGUGCUGAGU | 2 | 8 | 7 | 21 | 0.6102 | 1.9229 | 2.1308 |
| 609 | chi-miR-33a-3p | CAAUGUUUCCACAGUGCAUCA | 5 | 10 | 6 | 21 | 1.5255 | 2.4037 | 1.8264 |
| 610 | chi-miR-33b-3p | CAGUGCCUCGGCAGUGCAGCC | 0 | 1 | 0 | 21 | 0.0000 | 0.2404 | 0.0000 |
| 611 | chi-miR-342-3p | UCUCACACAGAAAUCGCACCCA | 1061 | 839 | 556 | 22 | 308.9952 | 192.5022 | 161.5531 |
| 612 | chi-miR-342-5p | AGGGGUGCUAUCUGUGGUUGAGG | 11 | 4 | 9 | 23 | 3.0642 | 0.8779 | 2.5014 |
| 613 | chi-miR-3431-3p | AUCUAGAGGACUGACUGAAAUU | 247 | 192 | 198 | 22 | 71.9338 | 44.0530 | 57.5315 |
| 614 | chi-miR-3432-5p | UGCGGGAUCUUUAGUUGUGGCG | 214 | 321 | 304 | 22 | 62.3233 | 73.6510 | 88.3312 |
| 615 | chi-miR-345-3p | CCCUGAACUAGGGGUCUGGAGG | 6 | 13 | 6 | 22 | 1.7474 | 2.9828 | 1.7434 |
| 616 | chi-miR-34b-3p | AAUCACUAGUUCCACUGCCAUC | 1 | 0 | 1 | 22 | 0.2912 | 0.0000 | 0.2906 |
| 617 | chi-miR-361-3p | UCCCCCAGGUGUGAUUCUGAUU | 319 | 430 | 289 | 22 | 92.9024 | 98.6603 | 83.9727 |
| 618 | chi-miR-363-3p | AAUUGCACGGUAUCCAUCUGCG | 0 | 3 | 6 | 22 | 0.0000 | 0.6883 | 1.7434 |
| 619 | chi-miR-369-5p | AGAUCGACCGUGUUAUAUUCG | 51 | 144 | 74 | 21 | 15.5600 | 34.6130 | 22.5256 |
| 620 | chi-miR-374a-3p | CUUAUCAGGUUGUAUUGUAAUU | 669 | 664 | 696 | 22 | 194.8330 | 152.3498 | 202.2319 |
| 621 | chi-miR-376a | GUAGAUUCUCCUUCUAUGAGU | 1 | 5 | 2 | 21 | 0.3051 | 1.2018 | 0.6088 |
| 622 | chi-miR-376b-3p | AUCAUAGAGGAAAAUCCAU | 23 | 28 | 16 | 19 | 7.7559 | 7.4388 | 5.3831 |
| 623 | chi-miR-376b-5p | GGUGGAUAUUCCUUCUAUGUUU | 12 | 14 | 11 | 22 | 3.4948 | 3.2122 | 3.1962 |
| 624 | chi-miR-376c-3p | AACAUAGAGGAAAUUCCACGU | 38 | 70 | 44 | 21 | 11.5937 | 16.8258 | 13.3936 |
| 625 | chi-miR-379-3p | UAUGUAACAUGGUCCACUAAC | 39 | 101 | 57 | 21 | 11.8988 | 24.2772 | 17.3508 |
| 626 | chi-miR-382-3p | AAUCAUUCACGGACAACACUU | 254 | 570 | 382 | 21 | 77.4950 | 137.0099 | 116.2806 |
| 627 | chi-miR-409-3p | GAAUGUUGCUCGGUGAACCCC | 577 | 844 | 500 | 21 | 176.0417 | 202.8708 | 152.1997 |
| 628 | chi-miR-411a-3p | UAUGUAACACGGUCCACUAAC | 57 | 139 | 69 | 21 | 17.3906 | 33.4112 | 21.0036 |
| 629 | chi-miR-411a-5p | AUAGUAGACCGUAUAGCGUAC | 1426 | 3581 | 1992 | 21 | 435.0701 | 860.7589 | 606.3637 |
| 630 | chi-miR-411b-3p | UAUGUCACAUGGUCCACUAAU | 0 | 9 | 0 | 21 | 0.0000 | 2.1633 | 0.0000 |
| 631 | chi-miR-412-5p | UGGUCGACCAGUUGGAAAGUAAU | 106 | 258 | 129 | 23 | 29.5282 | 56.6224 | 35.8530 |
| 632 | chi-miR-425-3p | CAUCGGGAAUGUCGUGUCCGCC | 5 | 8 | 4 | 22 | 1.4562 | 1.8355 | 1.1623 |
| 633 | chi-miR-429 | UAAUACUGUCUGGUAAUGCCG | 3 | 2 | 9 | 21 | 0.9153 | 0.4807 | 2.7396 |
| 634 | chi-miR-449a-3p | UCAGCUAACAUGCAACUGCUAUC | 1 | 1 | 1 | 23 | 0.2786 | 0.2195 | 0.2779 |
| 635 | chi-miR-449a-5p | UGGCAGUGUAUUGUUAGCUGG | 54 | 74 | 55 | 21 | 16.4753 | 17.7873 | 16.7420 |
| 636 | chi-miR-451-5p | AAACCGUUACCAUUACUGA | 3325 | 22076 | 8478 | 19 | 1121.2361 | 5864.9359 | 2852.3511 |
| 637 | chi-miR-454-5p | ACCCUAUCGAUAUUGUCUCUG | 5 | 2 | 0 | 21 | 1.5255 | 0.4807 | 0.0000 |
| 638 | chi-miR-483 | CACUCCUCUCCUCCCGUCUUCU | 3 | 15 | 5 | 22 | 0.8737 | 3.4416 | 1.4528 |
| 639 | chi-miR-485-3p | AGUCAUACACGGCUCUCCUCUCU | 7 | 15 | 16 | 23 | 1.9500 | 3.2920 | 4.4469 |
| 640 | chi-miR-485-5p | AGAGGCUGGCCGUGAUGAAUUC | 8 | 14 | 7 | 22 | 2.3298 | 3.2122 | 2.0339 |
| 641 | chi-miR-491-3p | CUUAUGCAAGAUUCCCUUCUAC | 0 | 1 | 0 | 22 | 0.0000 | 0.2294 | 0.0000 |
| 642 | chi-miR-493-5p | UUGUACAUGGUAGGCUUUCAUU | 1244 | 2444 | 1678 | 22 | 362.2903 | 560.7574 | 487.5649 |
| 643 | chi-miR-494 | UGAAACAUACACGGGAAACCUCU | 1850 | 1939 | 1559 | 23 | 515.3508 | 425.5459 | 433.2928 |
| 644 | chi-miR-496-3p | UGAGUAUUACAUGGCCAAUCU | 1 | 2 | 1 | 21 | 0.3051 | 0.4807 | 0.3044 |
| 645 | chi-miR-497-5p | AGCAGCACACUGUGGUUUGUAC | 160 | 317 | 197 | 22 | 46.5968 | 72.7333 | 57.2409 |
| 646 | chi-miR-502b-3p | AUCCACCUGGGCAAGGAUUCUGAA | 19 | 14 | 5 | 24 | 5.0723 | 2.9445 | 1.3317 |
| 647 | chi-miR-502b-5p | UAAUUCUUGCUCCCCAGGUGAG | 15 | 16 | 37 | 22 | 4.3685 | 3.6711 | 10.7508 |
| 648 | chi-miR-504 | AGACCCUGGUCUGCACUCUGU | 1 | 16 | 2 | 21 | 0.3051 | 3.8459 | 0.6088 |
| 649 | chi-miR-505-3p | UCAACACUUGCUGGUUUCCUCU | 39 | 36 | 45 | 22 | 11.3580 | 8.2599 | 13.0753 |
| 650 | chi-miR-532-3p | CCUCCCACACCCAAGGCUUGC | 14 | 46 | 33 | 21 | 4.2714 | 11.0569 | 10.0452 |
| 651 | chi-miR-542-3p | UGUGACAGAUUGAUAACUGA | 993 | 2325 | 1160 | 20 | 318.1107 | 586.7991 | 370.7585 |
| 652 | chi-miR-542-5p | UCGGGGAUCAUCAUGUCACGAGA | 5 | 13 | 9 | 23 | 1.3928 | 2.8531 | 2.5014 |
| 653 | chi-miR-543-3p | AAACAUUCGCGGUGCACUUCU | 209 | 388 | 207 | 21 | 63.7655 | 93.2629 | 63.0107 |
| 654 | chi-miR-543-5p | ACCUGUGGUGCUUAAGGAG | 1 | 12 | 5 | 19 | 0.3372 | 3.1880 | 1.6822 |
| 655 | chi-miR-544-5p | UCUUGUUAAAAGGCAGAUUCU | 5 | 2 | 5 | 21 | 1.5255 | 0.4807 | 1.5220 |
| 656 | chi-miR-545-3p | UCAACAAACAUUUAUUGUGUGC | 9 | 2 | 3 | 22 | 2.6211 | 0.4589 | 0.8717 |
| 657 | chi-miR-592 | UUGUGUCAAUAUGCGAUGAUGU | 1 | 8 | 7 | 22 | 0.2912 | 1.8355 | 2.0339 |
| 658 | chi-miR-660 | UACCCAUUGCAUAUCGGAGCUGU | 1134 | 1703 | 1339 | 23 | 315.8961 | 373.7518 | 372.1482 |
| 659 | chi-miR-708-3p | CAUCUAGACUGUGAGCUUCUAGA | 261 | 467 | 367 | 23 | 72.7062 | 102.4910 | 102.0003 |
| 660 | chi-miR-93-3p | ACUGCUGAGCCAGCACUUCCCGA | 5 | 2 | 8 | 23 | 1.3928 | 0.4389 | 2.2234 |
| 661 | chi-miR-96 | UUUGGCACUAGCACAUUUUUG | 3 | 4 | 5 | 21 | 0.9153 | 0.9615 | 1.5220 |
| 662 | chi-miR-98-3p | CUAUACAACUUACUACUUUCCC | 2 | 1 | 1 | 22 | 0.5825 | 0.2294 | 0.2906 |
| 663 | chi-miR-99a-3p | CAAGCUCGCUUCUAUGGGUCUGU | 7 | 33 | 19 | 23 | 1.9500 | 7.2424 | 5.2807 |
| 664 | chi-miR-99b-3p | CAAGCUCGUGUCUGUGGGUC | 39 | 55 | 54 | 20 | 12.4938 | 13.8813 | 17.2594 |
| 665 | cin-miR-7-5p | UGGAAGACUAGUGAUUUUGUUG | 4307 | 2416 | 2479 | 22 | 1254.3283 | 554.3330 | 720.3060 |
| 666 | cqu-miR-92 | AUUGCACUUGUCCCGGCCU | 10 | 25 | 18 | 19 | 3.3721 | 6.6418 | 6.0559 |
| 667 | crm-miR-1-3p | UGGAAUGUAAAGAAGUAUGU | 38 | 53 | 67 | 20 | 12.1734 | 13.3765 | 21.4145 |
| 668 | dre-miR-107a-3p | AGCAGCAUUGUACAGGGCUAUCA | 181 | 307 | 219 | 23 | 50.4208 | 67.3763 | 60.8667 |
| 669 | dre-miR-1306 | CCACCUCCCCUGCAAACGUCCA | 9 | 14 | 11 | 22 | 2.6211 | 3.2122 | 3.1962 |
| 670 | dre-miR-1388-5p | AGGACUGUCCAACCUGAGAAUG | 1 | 6 | 2 | 22 | 0.2912 | 1.3767 | 0.5811 |
| 671 | dre-miR-140-3p | UACCACAGGGUAGAACCACGGAC | 3361 | 3342 | 3749 | 23 | 936.2670 | 733.4577 | 1041.9593 |
| 672 | dre-miR-17a-5p | CAAAGUGCUUACAGUGCAGGUA | 646 | 600 | 423 | 22 | 188.1347 | 137.6655 | 122.9082 |
| 673 | dre-miR-181b-3p | CUCACUGAUCAAUGAAUGCAAA | 0 | 0 | 1 | 22 | 0.0000 | 0.0000 | 0.2906 |
| 674 | dre-miR-194a | UGUAACAGCAACUCCAUGUGG | 37 | 71 | 57 | 21 | 11.2886 | 17.0661 | 17.3508 |
| 675 | dre-miR-199-3p | UACAGUAGUCUGCACAUUGGUU | 9174 | 29197 | 17162 | 22 | 2671.7455 | 6699.0318 | 4986.6442 |
| 676 | dre-miR-23b | AUCACAUUGCCAGGGAUUACCA | 887 | 1685 | 1431 | 22 | 258.3212 | 386.6106 | 415.7958 |
| 677 | dre-miR-27b-5p | AGAGCUUAGCUGAUUGGUGAACA | 88 | 37 | 57 | 23 | 24.5140 | 8.1203 | 15.8420 |
| 678 | dre-miR-29b | UAGCACCAUUUGAAAUCAGUGU | 14 | 8 | 9 | 22 | 4.0772 | 1.8355 | 2.6151 |
| 679 | dre-miR-301c-5p | GCUCUGACGAUGUUGCACUAC | 34 | 8 | 16 | 21 | 10.3733 | 1.9229 | 4.8704 |
| 680 | dre-miR-30c-5p | UGUAAACAUCCUACACUCUCAG | 404 | 518 | 344 | 22 | 117.6570 | 118.8512 | 99.9537 |
| 681 | dre-miR-30e-5p | UGUAAACAUCCUUGACUGGAAG | 1010 | 1038 | 781 | 22 | 294.1425 | 238.1613 | 226.9298 |
| 682 | dvi-miR-125-5p | UCCCUGAGACCCUAACUUGUG | 976 | 3709 | 1862 | 21 | 297.7759 | 891.5260 | 566.7918 |
| 683 | eca-miR-125a-3p | ACAGGUGAGGUUCUUGGGAGCC | 3 | 8 | 3 | 22 | 0.8737 | 1.8355 | 0.8717 |
| 684 | eca-miR-1307 | ACUCGGCGUGGCGUCGGUCGUGGUA | 245 | 234 | 209 | 25 | 62.7892 | 47.2468 | 53.4404 |
| 685 | eca-miR-139-3p | GGAGACGCGGCCCUGUUGGAGU | 3 | 5 | 7 | 22 | 0.8737 | 1.1472 | 2.0339 |
| 686 | eca-miR-1468 | CUCCGUUUGCCUGUUUUGCUG | 198 | 181 | 208 | 21 | 60.4095 | 43.5067 | 63.3151 |
| 687 | eca-miR-146b-3p | UGCCCUAGGGACUCAGUUCUGG | 1 | 2 | 0 | 22 | 0.2912 | 0.4589 | 0.0000 |
| 688 | eca-miR-154b | AAUCAUACAUGGUUGACCUUUUU | 4 | 8 | 10 | 23 | 1.1143 | 1.7557 | 2.7793 |
| 689 | eca-miR-182 | UUUGGCAAUGGUAGAACUCACACUG | 14 | 6 | 18 | 25 | 3.5880 | 1.2115 | 4.6025 |
| 690 | eca-miR-1912 | UACCCAGAGCGUGCAGUGUGAA | 1 | 5 | 13 | 22 | 0.2912 | 1.1472 | 3.7773 |
| 691 | eca-miR-195 | UAGCAGCACAGAAAUAUUGGC | 764 | 1312 | 988 | 21 | 233.0951 | 315.3632 | 300.7467 |
| 692 | eca-miR-224 | CAAGUCACUAGUGGUUCCGUU | 1432 | 1335 | 944 | 21 | 436.9007 | 320.8917 | 287.3531 |
| 693 | eca-miR-302d | UAAGUGCUUCCAUGUUUUAGUGU | 0 | 0 | 1 | 23 | 0.0000 | 0.0000 | 0.2779 |
| 694 | eca-miR-345-3p | CCUGAACUAGGGGUCUGGAGGC | 6 | 13 | 6 | 22 | 1.7474 | 2.9828 | 1.7434 |
| 695 | eca-miR-362-3p | AACACACCUAUUCAAGGAUUCA | 2 | 12 | 6 | 22 | 0.5825 | 2.7533 | 1.7434 |
| 696 | eca-miR-376b | AUCAUAGAGGAAAAUCCAUGU | 23 | 28 | 16 | 21 | 7.0173 | 6.7303 | 4.8704 |
| 697 | eca-miR-378 | ACUGGACUUGGAGUCAGAAGG | 1495 | 1476 | 1165 | 21 | 456.1219 | 354.7836 | 354.6254 |
| 698 | eca-miR-3959 | UGUAUGUCAACUGAUCCACAGUC | 134 | 397 | 203 | 23 | 37.3281 | 87.1283 | 56.4198 |
| 699 | eca-miR-411 | UAGUAGACCGUAUAGCGUACG | 1426 | 3581 | 1992 | 21 | 435.0701 | 860.7589 | 606.3637 |
| 700 | eca-miR-421 | GGCCUCAUUAAAUGUUUGUUG | 1 | 0 | 0 | 21 | 0.3051 | 0.0000 | 0.0000 |
| 701 | eca-miR-424 | CAGCAGCAAUUCAUGUUUUGAA | 425 | 1156 | 478 | 22 | 123.7728 | 265.2355 | 138.8892 |
| 702 | eca-miR-485-3p | GUCAUACACGGCUCUCCUCUCU | 7 | 15 | 16 | 22 | 2.0386 | 3.4416 | 4.6490 |
| 703 | eca-miR-503 | UAGCAGCGGGAACAGUACUGCAG | 328 | 504 | 237 | 23 | 91.3703 | 110.6112 | 65.8694 |
| 704 | eca-miR-532-3p | CCUCCCACACCCAAGGCUUGCA | 14 | 46 | 33 | 22 | 4.0772 | 10.5544 | 9.5886 |
| 705 | efu-let-7d | AGAGGUAGUAGGUUGCAUAGUUUU | 336 | 548 | 355 | 24 | 89.6989 | 115.2566 | 94.5541 |
| 706 | efu-let-7e | UGAGGUAGGAGGUUGUAUAGUUGA | 671 | 1440 | 1066 | 24 | 179.1308 | 302.8641 | 283.9286 |
| 707 | efu-miR-103b | AGCAGCAUUGUACAGGGCUAUGAAA | 1136 | 1717 | 1148 | 25 | 291.1370 | 346.6784 | 293.5385 |
| 708 | efu-miR-106 | UACCGCACUGUGGGUACUUGCUGCU | 224 | 364 | 261 | 25 | 57.4073 | 73.4950 | 66.7365 |
| 709 | efu-miR-107 | AGCAGCAUUGUACAGGGCUAUCAAA | 183 | 308 | 220 | 25 | 46.8997 | 62.1881 | 56.2530 |
| 710 | efu-miR-125a | ACGGGUUAGGCUCUUGGGAGCU | 259 | 555 | 435 | 22 | 75.4286 | 127.3406 | 126.3950 |
| 711 | efu-miR-125b | UCCCUGAGACCCUAACUUGUGAGG | 1006 | 3827 | 1919 | 24 | 268.5627 | 804.9033 | 511.1247 |
| 712 | efu-miR-126 | CUCGUACCGUGAGUAAUAAUGCG | 7951 | 12855 | 10535 | 23 | 2214.8940 | 2821.2445 | 2927.9919 |
| 713 | efu-miR-128a | UCACAGUGAACCGGUCUCUUUC | 145 | 325 | 119 | 22 | 42.2284 | 74.5688 | 34.5770 |
| 714 | efu-miR-128b | UCACAGUGAACCGGUCUCUUUUU | 165 | 183 | 106 | 23 | 45.9637 | 40.1624 | 29.4606 |
| 715 | efu-miR-133-3p | UUUGGUCCCCUUCAACCAGCUGUA | 14 | 18 | 19 | 24 | 3.7375 | 3.7858 | 5.0606 |
| 716 | efu-miR-133-5p | AGCUGGUAAAAUGGAACCAAAUC | 0 | 1 | 3 | 23 | 0.0000 | 0.2195 | 0.8338 |
| 717 | efu-miR-143 | GUCUGAGAUGAAGCACUGUAGCUC | 11787 | 25658 | 19218 | 24 | 3146.6691 | 5396.4486 | 5118.7052 |
| 718 | efu-miR-145 | GUCCAGUUUUCCCAGGAAUCCCUUA | 1344 | 1429 | 1171 | 25 | 344.4437 | 288.5285 | 299.4195 |
| 719 | efu-miR-155 | UUAAUGCUAAUCGUGAUAGGGGUUU | 813 | 962 | 939 | 25 | 208.3577 | 194.2368 | 240.0981 |
| 720 | efu-miR-16 | UAGCAGCACGUAAAUAUUGGCGU | 434 | 686 | 572 | 23 | 120.8985 | 150.5542 | 158.9759 |
| 721 | efu-miR-181a | AACCAUCGACCGUUGAUUGUACC | 141 | 88 | 145 | 23 | 39.2781 | 19.3131 | 40.2998 |
| 722 | efu-miR-181d | AACAUUCAUUGUUGUCGGUGGGUU | 56 | 114 | 78 | 24 | 14.9498 | 23.9767 | 20.7753 |
| 723 | efu-miR-181e | AACAUUCAUUGCUGUCGGUGGGUUU | 371 | 353 | 400 | 25 | 95.0808 | 71.2740 | 102.2782 |
| 724 | efu-miR-181f | ACCACCGACCGUUGACUGUACC | 68 | 74 | 74 | 22 | 19.8037 | 16.9787 | 21.5017 |
| 725 | efu-miR-185 | UGGAGAGAAAGGCAGUUCCUGAU | 728 | 1610 | 758 | 23 | 202.7975 | 353.3414 | 210.6709 |
| 726 | efu-miR-199 | UACAGUAGUCUGCACAUUGGUUA | 9174 | 29197 | 17162 | 23 | 2555.5826 | 6407.7695 | 4769.8336 |
| 727 | efu-miR-20 | UAAAGUGCUUAUAGUGCAGGUAGUG | 2313 | 1762 | 1341 | 25 | 592.7815 | 355.7643 | 342.8877 |
| 728 | efu-miR-200a | UAACACUGUCUGGUAACGAUGUUC | 9 | 4 | 24 | 24 | 2.4026 | 0.8413 | 6.3924 |
| 729 | efu-miR-200b | UAAUACUGCCUGGUAAUGAUGACG | 36 | 13 | 92 | 24 | 9.6106 | 2.7342 | 24.5042 |
| 730 | efu-miR-205 | UCCUUCAUUCCACCGGAGUCUGU | 16 | 12 | 44 | 23 | 4.4571 | 2.6336 | 12.2289 |
| 731 | efu-miR-214 | UACAGCAGGCACAGACAGGCAGU | 970 | 1906 | 1390 | 23 | 270.2109 | 418.3035 | 386.3226 |
| 732 | efu-miR-218a | UUGUGCUUGAUCUAACCAUGUGGUU | 358 | 416 | 488 | 25 | 91.7491 | 83.9943 | 124.7794 |
| 733 | efu-miR-221 | ACCUGGCAUACAAUGUAGAUUUCUG | 46 | 39 | 48 | 25 | 11.7890 | 7.8745 | 12.2734 |
| 734 | efu-miR-223 | UGUCAGUUUGUCAAAUACCCCAA | 3 | 8 | 6 | 23 | 0.8357 | 1.7557 | 1.6676 |
| 735 | efu-miR-23b | AAUCACAUUGCCAGGGAUUACCACG | 1037 | 1881 | 1601 | 25 | 265.7650 | 379.7915 | 409.3686 |
| 736 | efu-miR-25 | CAUUGCACUUGUCUCGGUCUGACA | 577 | 923 | 598 | 24 | 154.0365 | 194.1274 | 159.2770 |
| 737 | efu-miR-26b | UUCAAGUAAUUCAGGAUAGGUUGU | 1511 | 2641 | 2964 | 24 | 403.3780 | 555.4611 | 789.4600 |
| 738 | efu-miR-28 | AAGGAGCUCACAGUCUAUUGAGU | 99 | 207 | 166 | 23 | 27.5782 | 45.4296 | 46.1364 |
| 739 | efu-miR-299 | UGGUUUACCGUCCCACAUACA | 7 | 18 | 11 | 21 | 2.1357 | 4.3266 | 3.3484 |
| 740 | efu-miR-29a | CUAGCACCAUCUGAAAUCGGUUAU | 175 | 253 | 170 | 24 | 46.7182 | 53.2115 | 45.2794 |
| 741 | efu-miR-30b | UGUAAACAUCCUACACUCUCAGCUG | 411 | 524 | 349 | 25 | 105.3321 | 105.8005 | 89.2377 |
| 742 | efu-miR-30c | CUGGGAGAGGGUUGUUUACUCCU | 3 | 6 | 4 | 23 | 0.8357 | 1.3168 | 1.1117 |
| 743 | efu-miR-30d | CUUUCAGUCAGAUGUUUGCUGCU | 2 | 4 | 3 | 23 | 0.5571 | 0.8779 | 0.8338 |
| 744 | efu-miR-30e | GCUUUCAGUCGGAUGUUUACAGC | 101 | 136 | 115 | 23 | 28.1354 | 29.8475 | 31.9619 |
| 745 | efu-miR-320 | AAAAGCUGGGUUGAGAGGGCGAAAA | 524 | 573 | 343 | 25 | 134.2921 | 115.6941 | 87.7036 |
| 746 | efu-miR-323 | GCACAUUACACGGUCGACCUCUU | 56 | 124 | 81 | 23 | 15.5998 | 27.2139 | 22.5123 |
| 747 | efu-miR-331 | GCCCCUGGGCCUAUCCUAGAAC | 10 | 17 | 14 | 22 | 2.9123 | 3.9005 | 4.0679 |
| 748 | efu-miR-34a | UGGCAGUGUCUUAGCUGGUUGUUG | 68 | 169 | 82 | 24 | 18.1533 | 35.5445 | 21.8407 |
| 749 | efu-miR-379 | UGGUAGACUAUGGAACGUAGGCUUU | 1502 | 3616 | 2232 | 25 | 384.9364 | 730.1043 | 570.7125 |
| 750 | efu-miR-381 | AUAUACAAGGGCAAGCUCUCUGU | 422 | 494 | 386 | 23 | 117.5557 | 108.4166 | 107.2810 |
| 751 | efu-miR-409 | CGAAUGUUGCUCGGUGAACCCCUU | 577 | 844 | 500 | 24 | 154.0365 | 177.5120 | 133.1748 |
| 752 | efu-miR-423 | UGAGGGGCAGAGAGCGAGACUUUU | 866 | 1016 | 719 | 24 | 231.1882 | 213.6874 | 191.5053 |
| 753 | efu-miR-452 | UCAGUCUCAUCUGCAAAGAAGU | 60 | 76 | 48 | 22 | 17.4738 | 17.4376 | 13.9470 |
| 754 | efu-miR-487 | GUGGUUAUCCCUGCUGUGUUCG | 4 | 6 | 0 | 22 | 1.1649 | 1.3767 | 0.0000 |
| 755 | efu-miR-493 | UUGUACAUGGUAGGCUUUCAUUCAU | 1244 | 2444 | 1678 | 25 | 318.8155 | 493.4665 | 429.0571 |
| 756 | efu-miR-495 | AAACAAACAUGGUGCACUUCUUU | 345 | 744 | 421 | 23 | 96.1060 | 163.2832 | 117.0085 |
| 757 | efu-miR-497 | CAGCAGCACACUGUGGUUUGUAC | 160 | 317 | 197 | 23 | 44.5709 | 69.5709 | 54.7522 |
| 758 | efu-miR-500 | AAUGCACCUGGGCAAGGAUUCUGA | 101 | 169 | 132 | 24 | 26.9631 | 35.5445 | 35.1581 |
| 759 | efu-miR-503 | UGGAGUAUUGUUUCUGCUGCCCGG | 23 | 45 | 17 | 24 | 6.1401 | 9.4645 | 4.5279 |
| 760 | efu-miR-532 | CAUGCCUUGAGUGUAGGACCGUU | 861 | 1416 | 1235 | 23 | 239.8470 | 310.7649 | 343.2435 |
| 761 | efu-miR-628 | AUGCUGACAUAUUUACUAGAGGGU | 1 | 6 | 3 | 24 | 0.2670 | 1.2619 | 0.7990 |
| 762 | efu-miR-7a | CUGGAAGACUAGUGAUUUUGUUGUU | 4332 | 2439 | 2500 | 25 | 1110.2160 | 492.4570 | 639.2389 |
| 763 | efu-miR-7c | UGGAAGACUAGUGAUUUUGUUGUUC | 4364 | 2433 | 2490 | 25 | 1118.4170 | 491.2455 | 636.6819 |
| 764 | efu-miR-874 | CUGCCCUGGCCCGAGGGACCGACU | 14 | 27 | 19 | 24 | 3.7375 | 5.6787 | 5.0606 |
| 765 | fru-miR-7 | UGGAAGACUAGUGAUUUUGUU | 4307 | 2416 | 2479 | 21 | 1314.0582 | 580.7298 | 754.6063 |
| 766 | gga-let-7g-5p | UGAGGUAGUAGUUUGUACAGU | 38050 | 55889 | 39633 | 21 | 11608.9889 | 13433.9440 | 12064.2637 |
| 767 | gga-miR-101-3p | GUACAGUACUGUGAUAACUGAA | 384 | 809 | 527 | 22 | 111.8324 | 185.6190 | 153.1268 |
| 768 | gga-miR-103-2-5p | AGCUUCUUUACAGUGCUGCCUUG | 0 | 1 | 0 | 23 | 0.0000 | 0.2195 | 0.0000 |
| 769 | gga-miR-10b-3p | AGAUUCGAUUCUAGGGGAAUA | 2 | 9 | 5 | 21 | 0.6102 | 2.1633 | 1.5220 |
| 770 | gga-miR-125b-3p | ACAAGUCAGGCUCUUGGGACCU | 58 | 212 | 122 | 22 | 16.8913 | 48.6418 | 35.4487 |
| 771 | gga-miR-126-3p | UCGUACCGUGAGUAAUAAUGCGC | 7951 | 12854 | 10535 | 23 | 2214.8940 | 2821.0251 | 2927.9919 |
| 772 | gga-miR-1306-3p | UGGACGUUGGCUCUGGUGGUGAU | 6 | 10 | 5 | 23 | 1.6714 | 2.1947 | 1.3896 |
| 773 | gga-miR-140-3p | CCACAGGGUAGAACCACGGAC | 3359 | 3340 | 3747 | 21 | 1024.8251 | 802.8301 | 1140.5848 |
| 774 | gga-miR-140-5p | AGUGGUUUUACCCUAUGGUAG | 1176 | 849 | 788 | 21 | 358.7956 | 204.0727 | 239.8668 |
| 775 | gga-miR-221-5p | AACCUGGCAUACAAUGUAGAUUUCUGU | 46 | 39 | 48 | 27 | 10.9157 | 7.2912 | 11.3642 |
| 776 | gga-miR-26a-5p | UUCAAGUAAUCCAGGAUAGGC | 11476 | 15770 | 14591 | 21 | 3501.3077 | 3790.6081 | 4441.4925 |
| 777 | gga-miR-29c-3p | UAGCACCAUUUGAAAUCGGU | 1 | 2 | 1 | 20 | 0.3204 | 0.5048 | 0.3196 |
| 778 | gga-miR-30c-2-3p | UGGGAGAAGGCUGUUUACUCU | 2 | 10 | 8 | 21 | 0.6102 | 2.4037 | 2.4352 |
| 779 | gga-miR-30e-3p | UUUCAGUCGGAUGUUUACAGC | 100 | 135 | 115 | 21 | 30.5098 | 32.4497 | 35.0059 |
| 780 | gga-miR-30e-5p | UGUAAACAUCCUUGACUGG | 996 | 1029 | 774 | 19 | 335.8650 | 273.3747 | 260.4057 |
| 781 | gga-miR-34a-5p | UGGCAGUGUCUUAGCUGGUUGUU | 68 | 169 | 82 | 23 | 18.9426 | 37.0899 | 22.7903 |
| 782 | gga-miR-365-1-5p | GAGGGACUUUUGGGGGCAGAUGU | 89 | 41 | 55 | 23 | 24.7926 | 8.9981 | 15.2861 |
| 783 | gga-miR-365-2-5p | GAGGGACUUUCAGGGGCAGCUGU | 16 | 5 | 11 | 23 | 4.4571 | 1.0973 | 3.0572 |
| 784 | gga-miR-92-3p | UAUUGCACUUGUCCCGGCCUG | 2383 | 3333 | 2188 | 21 | 727.0492 | 801.1475 | 666.0260 |
| 785 | gga-miR-99a-3p | CAAGCUCGCUUCUAUGGGUCU | 7 | 33 | 19 | 21 | 2.1357 | 7.9322 | 5.7836 |
| 786 | ggo-let-7c | UGAGGUAGUAGGUUGUAUGGU | 4662 | 18099 | 8731 | 21 | 1422.3681 | 4350.4259 | 2657.7117 |
| 787 | ggo-let-7f | UGAGGUAGUAGAUUGUAUAGU | 26320 | 39435 | 28762 | 21 | 8030.1863 | 9478.9239 | 8755.1372 |
| 788 | ggo-miR-125a | UCCCUGAGACCCUUUAACCUG | 746 | 1401 | 854 | 21 | 227.6033 | 336.7560 | 259.9571 |
| 789 | ggo-miR-127 | UCGGAUCCGUCUGAGCUUGGC | 2457 | 5402 | 3868 | 21 | 749.6264 | 1298.4696 | 1177.4171 |
| 790 | ggo-miR-128 | UCACAGUGAACCGGUCUCUU | 165 | 183 | 106 | 20 | 52.8583 | 46.1868 | 33.8797 |
| 791 | ggo-miR-1298 | UUCAUUCGGCUGUCCAGAUG | 74 | 438 | 1015 | 20 | 23.7061 | 110.5454 | 324.4137 |
| 792 | ggo-miR-130b | CAGUGCAAUGAUGAAAGGGCA | 6 | 14 | 12 | 21 | 1.8306 | 3.3652 | 3.6528 |
| 793 | ggo-miR-134 | UGUGACUGGUUGACCAGAGGG | 77 | 145 | 83 | 21 | 23.4926 | 34.8534 | 25.2652 |
| 794 | ggo-miR-145 | GUCCAGUUUUCCCAGGAAUCCCUU | 1344 | 1429 | 1171 | 24 | 358.7956 | 300.5505 | 311.8953 |
| 795 | ggo-miR-146a | UGAGAACUGAAUUCCAUGGGU | 235 | 422 | 305 | 21 | 71.6981 | 101.4354 | 92.8418 |
| 796 | ggo-miR-148a | UCAGUGCACUACAGAACUUUG | 755406 | 781385 | 704227 | 21 | 230473.0582 | 187820.1847 | 214366.3159 |
| 797 | ggo-miR-151a | UCGAGGAGCUCACAGUCUAG | 103 | 245 | 159 | 20 | 32.9964 | 61.8347 | 50.8195 |
| 798 | ggo-miR-191 | CAACGGAAUCCCAAAAGCAGC | 915 | 1370 | 984 | 21 | 279.1649 | 329.3046 | 299.5291 |
| 799 | ggo-miR-193a | AACUGGCCUACAAAGUCCCAG | 3 | 2 | 2 | 21 | 0.9153 | 0.4807 | 0.6088 |
| 800 | ggo-miR-26b | UUCAAGUAAUUCAGGAUAGGU | 1511 | 2641 | 2964 | 21 | 461.0035 | 634.8127 | 902.2400 |
| 801 | ggo-miR-323a | CACAUUACACGGUCGACCUC | 56 | 124 | 81 | 20 | 17.9398 | 31.2960 | 25.8892 |
| 802 | ggo-miR-328 | CUGGCCCUCUCUGCCCUUCCG | 92 | 217 | 119 | 21 | 28.0690 | 52.1599 | 36.2235 |
| 803 | ggo-miR-335 | UCAAGAGCAAUAACGAAAAAUG | 123 | 188 | 146 | 22 | 35.8213 | 43.1352 | 42.4222 |
| 804 | ggo-miR-365a | UAAUGCCCCUAAAAAUCCUUA | 232 | 177 | 161 | 21 | 70.7828 | 42.5452 | 49.0083 |
| 805 | ggo-miR-370 | GCCUGCUGGGGUGGAACCUGGUC | 144 | 217 | 123 | 23 | 40.1138 | 47.6243 | 34.1854 |
| 806 | ggo-miR-376b | AUCAUAGAGGAAAAUCCAUG | 23 | 28 | 16 | 20 | 7.3681 | 7.0668 | 5.1139 |
| 807 | ggo-miR-376c | AACAUAGAGGAAAUUCCACG | 38 | 70 | 44 | 20 | 12.1734 | 17.6671 | 14.0633 |
| 808 | ggo-miR-378a | ACUGGACUUGGAGUCAGAAGGCC | 1495 | 1476 | 1165 | 23 | 416.4591 | 323.9329 | 323.7884 |
| 809 | ggo-miR-381 | UAUACAAGGGCAAGCUCUCUG | 422 | 494 | 386 | 21 | 128.7515 | 118.7419 | 117.4982 |
| 810 | ggo-miR-409 | AGGUUACCCGAGCAACUUUGCA | 213 | 350 | 204 | 22 | 62.0320 | 80.3049 | 59.2749 |
| 811 | ggo-miR-410 | AAUAUAACACAGAUGGCCUG | 44 | 69 | 41 | 20 | 14.0955 | 17.4147 | 13.1044 |
| 812 | ggo-miR-423 | UGAGGGGCAGAGAGCGAGACUU | 866 | 1016 | 719 | 22 | 252.2053 | 233.1135 | 208.9149 |
| 813 | ggo-miR-431 | UGCAGGUCGUCUUGCAGGGCU | 22 | 27 | 18 | 21 | 6.7122 | 6.4899 | 5.4792 |
| 814 | ggo-miR-432 | UCUUGGAGUAGGUCAUUGGGUG | 54 | 119 | 70 | 22 | 15.7264 | 27.3037 | 20.3394 |
| 815 | ggo-miR-433 | AUCAUGAUGGGCUCCUCGGUG | 79 | 176 | 92 | 21 | 24.1028 | 42.3048 | 28.0047 |
| 816 | ggo-miR-454 | UAGUGCAAUAUUGCUUAUAGGGUU | 96 | 77 | 73 | 24 | 25.6283 | 16.1948 | 19.4435 |
| 817 | ggo-miR-485 | AGAGGCUGGCCGUGAUGAAU | 8 | 14 | 7 | 20 | 2.5628 | 3.5334 | 2.2373 |
| 818 | ggo-miR-487b | AAUCGUACAGGGUCAUCCACU | 28 | 68 | 38 | 21 | 8.5428 | 16.3450 | 11.5672 |
| 819 | ggo-miR-491 | AGUGGGGAACCCUUCCAUGAGGA | 4 | 15 | 6 | 23 | 1.1143 | 3.2920 | 1.6676 |
| 820 | ggo-miR-493 | UUGUACAUGGUAGGCUUUCAU | 1242 | 2441 | 1678 | 21 | 378.9320 | 586.7390 | 510.7823 |
| 821 | ggo-miR-495 | AAACAAACAUGGUGCACUUCU | 353 | 770 | 449 | 21 | 107.6997 | 185.0836 | 136.6754 |
| 822 | ggo-miR-497 | CAGCAGCACACUGUGGUUUG | 160 | 317 | 197 | 20 | 51.2565 | 80.0066 | 62.9650 |
| 823 | ggo-miR-499a | UUAAGACUUGCAGUGAUGUU | 8 | 12 | 6 | 20 | 2.5628 | 3.0286 | 1.9177 |
| 824 | ggo-miR-502b | AUGCACCUGGGCAAGGAUUCUGA | 101 | 169 | 132 | 23 | 28.1354 | 37.0899 | 36.6868 |
| 825 | ggo-miR-532 | CAUGCCUUGAGUGUAGGACCG | 858 | 1413 | 1232 | 21 | 261.7743 | 339.6404 | 375.0201 |
| 826 | ggo-miR-582 | UUACAGUUGUUCAACCAGUUAC | 2 | 7 | 1 | 22 | 0.5825 | 1.6061 | 0.2906 |
| 827 | ggo-miR-656 | AAUAUUAUACAGUCAACCUC | 84 | 111 | 87 | 20 | 26.9097 | 28.0149 | 27.8069 |
| 828 | ggo-miR-760 | CGGCUCUGGGUCUGUGGGGAG | 5 | 6 | 2 | 21 | 1.5255 | 1.4422 | 0.6088 |
| 829 | ggo-miR-96 | UUUGGCACUAGCACAUUUUUGC | 3 | 4 | 5 | 22 | 0.8737 | 0.9178 | 1.4528 |
| 830 | hhi-miR-183 | UAUGGCACUGGUAGAAUUCACUGU | 3 | 5 | 13 | 24 | 0.8009 | 1.0516 | 3.4625 |
| 831 | hhi-miR-301 | CAGUGCAAUAGUAUUGUCAAAGCA | 2 | 3 | 0 | 24 | 0.5339 | 0.6310 | 0.0000 |
| 832 | hsa-let-7f-2-3p | CUAUACAGUCUACUGUCUUUCC | 5 | 11 | 8 | 22 | 1.4562 | 2.5239 | 2.3245 |
| 833 | hsa-miR-106a-5p | AAAAGUGCUUACAGUGCAGGUAG | 2 | 1 | 2 | 23 | 0.5571 | 0.2195 | 0.5559 |
| 834 | hsa-miR-1260a | AUCCCACCUCUGCCACCA | 2 | 12 | 6 | 18 | 0.7119 | 3.3652 | 2.1308 |
| 835 | hsa-miR-127-5p | CUGAAGCUCAGAGGGCUCUGAU | 82 | 209 | 128 | 22 | 23.8809 | 47.9535 | 37.1921 |
| 836 | hsa-miR-1307-5p | UCGACCGGACCUCGACCGGCU | 3 | 4 | 3 | 21 | 0.9153 | 0.9615 | 0.9132 |
| 837 | hsa-miR-130b-5p | ACUCUUUCCCUGUUGCACUAC | 89 | 77 | 65 | 21 | 27.1537 | 18.5084 | 19.7860 |
| 838 | hsa-miR-136-3p | CAUCAUCGUCUCAAAUGAGUCU | 263 | 793 | 409 | 22 | 76.5935 | 181.9479 | 118.8403 |
| 839 | hsa-miR-143-5p | GGUGCAGUGCUGCAUCUCUGGU | 89 | 178 | 132 | 22 | 25.9195 | 40.8408 | 38.3543 |
| 840 | hsa-miR-151b | UCGAGGAGCUCACAGUCU | 6 | 18 | 14 | 18 | 2.1357 | 5.0477 | 4.9719 |
| 841 | hsa-miR-15b-3p | CGAAUCAUUAUUUGCUGCUCUA | 19 | 21 | 18 | 22 | 5.5334 | 4.8183 | 5.2301 |
| 842 | hsa-miR-16-1-3p | CCAGUAUUAACUGUGCUGCUGA | 4 | 3 | 4 | 22 | 1.1649 | 0.6883 | 1.1623 |
| 843 | hsa-miR-181b-2-3p | CUCACUGAUCAAUGAAUGCA | 0 | 0 | 1 | 20 | 0.0000 | 0.0000 | 0.3196 |
| 844 | hsa-miR-181b-3p | CUCACUGAACAAUGAAUGCAA | 3 | 7 | 7 | 21 | 0.9153 | 1.6826 | 2.1308 |
| 845 | hsa-miR-181c-3p | AACCAUCGACCGUUGAGUGGAC | 1 | 4 | 5 | 22 | 0.2912 | 0.9178 | 1.4528 |
| 846 | hsa-miR-1911-5p | UGAGUACCGCCAUGUCUGUUGGG | 1 | 6 | 7 | 23 | 0.2786 | 1.3168 | 1.9455 |
| 847 | hsa-miR-194-3p | CCAGUGGGGCUGCUGUUAUCUG | 3 | 2 | 2 | 22 | 0.8737 | 0.4589 | 0.5811 |
| 848 | hsa-miR-195-3p | CCAAUAUUGGCUGUGCUGCUCC | 177 | 156 | 142 | 22 | 51.5477 | 35.7930 | 41.2600 |
| 849 | hsa-miR-221-5p | ACCUGGCAUACAAUGUAGAUUU | 46 | 39 | 48 | 22 | 13.3966 | 8.9483 | 13.9470 |
| 850 | hsa-miR-223-5p | CGUGUAUUUGACAAGCUGAGUU | 1 | 0 | 2 | 22 | 0.2912 | 0.0000 | 0.5811 |
| 851 | hsa-miR-24-1-5p | UGCCUACUGAGCUGAUAUCAGU | 15 | 25 | 25 | 22 | 4.3685 | 5.7361 | 7.2641 |
| 852 | hsa-miR-24-2-5p | UGCCUACUGAGCUGAAACACAG | 91 | 85 | 74 | 22 | 26.5019 | 19.5026 | 21.5017 |
| 853 | hsa-miR-25-5p | AGGCGGAGACUUGGGCAAUUG | 2 | 2 | 2 | 21 | 0.6102 | 0.4807 | 0.6088 |
| 854 | hsa-miR-29a-5p | ACUGAUUUCUUUUGGUGUUCAG | 1 | 1 | 1 | 22 | 0.2912 | 0.2294 | 0.2906 |
| 855 | hsa-miR-29c-5p | UGACCGAUUUCUCCUGGUGUUC | 9 | 12 | 9 | 22 | 2.6211 | 2.7533 | 2.6151 |
| 856 | hsa-miR-3064-5p | UCUGGCUGUUGUGGUGUGCAA | 1 | 1 | 1 | 21 | 0.3051 | 0.2404 | 0.3044 |
| 857 | hsa-miR-30c-1-3p | CUGGGAGAGGGUUGUUUACUCC | 3 | 6 | 4 | 22 | 0.8737 | 1.3767 | 1.1623 |
| 858 | hsa-miR-324-3p | ACUGCCCCAGGUGCUGCUGG | 0 | 1 | 0 | 20 | 0.0000 | 0.2524 | 0.0000 |
| 859 | hsa-miR-331-5p | CUAGGUAUGGUCCCAGGGAUCC | 27 | 35 | 29 | 22 | 7.8632 | 8.0305 | 8.4263 |
| 860 | hsa-miR-34a-3p | CAAUCAGCAAGUAUACUGCCCU | 0 | 1 | 0 | 22 | 0.0000 | 0.2294 | 0.0000 |
| 861 | hsa-miR-361-3p | UCCCCCAGGUGUGAUUCUGAUUU | 319 | 430 | 289 | 23 | 88.8632 | 94.3707 | 80.3218 |
| 862 | hsa-miR-365b-5p | AGGGACUUUCAGGGGCAGCUGU | 16 | 5 | 11 | 22 | 4.6597 | 1.1472 | 3.1962 |
| 863 | hsa-miR-370-5p | CAGGUCACGUCUCUGCAGUUAC | 0 | 1 | 1 | 22 | 0.0000 | 0.2294 | 0.2906 |
| 864 | hsa-miR-376a-5p | GUAGAUUCUCCUUCUAUGAGUA | 1 | 5 | 2 | 22 | 0.2912 | 1.1472 | 0.5811 |
| 865 | hsa-miR-376c-5p | GGUGGAUAUUCCUUCUAUGUU | 16 | 16 | 16 | 21 | 4.8816 | 3.8459 | 4.8704 |
| 866 | hsa-miR-378d | ACUGGACUUGGAGUCAGAAA | 34 | 42 | 50 | 20 | 10.8920 | 10.6002 | 15.9810 |
| 867 | hsa-miR-378f | ACUGGACUUGGAGCCAGAAG | 3 | 1 | 0 | 20 | 0.9611 | 0.2524 | 0.0000 |
| 868 | hsa-miR-379-3p | UAUGUAACAUGGUCCACUAACU | 39 | 101 | 57 | 22 | 11.3580 | 23.1737 | 16.5621 |
| 869 | hsa-miR-411-3p | UAUGUAACACGGUCCACUAACC | 57 | 139 | 69 | 22 | 16.6001 | 31.8925 | 20.0489 |
| 870 | hsa-miR-431-3p | CAGGUCGUCUUGCAGGGCUUCU | 14 | 17 | 14 | 22 | 4.0772 | 3.9005 | 4.0679 |
| 871 | hsa-miR-431-5p | UGUCUUGCAGGCCGUCAUGCA | 70 | 231 | 123 | 21 | 21.3569 | 55.5251 | 37.4411 |
| 872 | hsa-miR-487b-5p | GUGGUUAUCCCUGUCCUGUUCG | 1 | 1 | 0 | 22 | 0.2912 | 0.2294 | 0.0000 |
| 873 | hsa-miR-500a-3p | AUGCACCUGGGCAAGGAUUCUG | 101 | 169 | 132 | 22 | 29.4142 | 38.7758 | 38.3543 |
| 874 | hsa-miR-539-3p | AUCAUACAAGGACAAUUUCUUU | 13 | 16 | 9 | 22 | 3.7860 | 3.6711 | 2.6151 |
| 875 | hsa-miR-541-5p | AAAGGAUUCUGCUGUCGGUCCCACU | 47 | 103 | 41 | 25 | 12.0453 | 20.7967 | 10.4835 |
| 876 | hsa-miR-6516-3p | AUCAUGUAUGAUACUGCAAACA | 6 | 23 | 14 | 22 | 1.7474 | 5.2772 | 4.0679 |
| 877 | hsa-miR-6516-5p | UUUGCAGUAACAGGUGUGAGCA | 15 | 28 | 11 | 22 | 4.3685 | 6.4244 | 3.1962 |
| 878 | hsa-miR-92a-1-5p | AGGUUGGGAUCGGUUGCAAUGCU | 29 | 4 | 5 | 23 | 8.0785 | 0.8779 | 1.3896 |
| 879 | hsa-miR-92b-5p | AGGGACGGGACGCGGUGCAGUG | 5 | 4 | 2 | 22 | 1.4562 | 0.9178 | 0.5811 |
| 880 | hsa-miR-99b-3p | CAAGCUCGUGUCUGUGGGUCCG | 39 | 55 | 54 | 22 | 11.3580 | 12.6193 | 15.6904 |
| 881 | ipu-miR-107b | AGCAGCAUUGUACAGGGCU | 2 | 6 | 1 | 19 | 0.6744 | 1.5940 | 0.3364 |
| 882 | ipu-miR-155 | UUAAUGCUAAUCGUGAUAGGGGUU | 801 | 955 | 926 | 24 | 213.8357 | 200.8578 | 246.6397 |
| 883 | ipu-miR-18a | UAAGGUGCAUCUAGUGCAGA | 47 | 30 | 21 | 20 | 15.0566 | 7.5716 | 6.7120 |
| 884 | ipu-miR-218a | UUGUGCUUGAUCUAACCAUG | 356 | 415 | 485 | 20 | 114.0457 | 104.7405 | 155.0154 |
| 885 | lla-miR-139 | UCUACAGUGCACGUGUCU | 129 | 240 | 180 | 18 | 45.9173 | 67.3031 | 63.9239 |
| 886 | mdo-miR-125b-2-3p | ACGGGUUAGGCUCUUGGGAGC | 23 | 67 | 53 | 21 | 7.0173 | 16.1047 | 16.1332 |
| 887 | mdo-miR-135b-5p | UAUGGCUUUUCAUUCCUAUGUG | 5 | 5 | 2 | 22 | 1.4562 | 1.1472 | 0.5811 |
| 888 | mdo-miR-145-3p | AUUCCUGGAAAUACUGUUCU | 128 | 197 | 159 | 20 | 41.0052 | 49.7202 | 50.8195 |
| 889 | mdo-miR-17-3p | ACUGCAGUGAAGGCACUUGUA | 3 | 6 | 5 | 21 | 0.9153 | 1.4422 | 1.5220 |
| 890 | mdo-miR-181a-1-3p | CCAUCGACCGUUGAUUGUACC | 140 | 86 | 142 | 21 | 42.7138 | 20.6717 | 43.2247 |
| 891 | mdo-miR-18a-3p | ACUGCCCUAAGUGCUCCUUCUGGC | 3 | 4 | 3 | 24 | 0.8009 | 0.8413 | 0.7990 |
| 892 | mdo-miR-199b-2-5p | CCAGUGUUCAGACUACCUGUUC | 20 | 44 | 35 | 22 | 5.8246 | 10.0955 | 10.1697 |
| 893 | mdo-miR-218-2-3p | AUGGUUCUGUCAAGCACCGCG | 0 | 0 | 1 | 21 | 0.0000 | 0.0000 | 0.3044 |
| 894 | mdo-miR-23a-3p | AUCACAUUGCCAGGGAUUUC | 1823 | 2814 | 3053 | 20 | 584.0038 | 710.2162 | 975.7981 |
| 895 | mdo-miR-24-5p | GUGCCUACUGAGCUGAAACACAGU | 82 | 81 | 69 | 24 | 21.8908 | 17.0361 | 18.3781 |
| 896 | mdo-miR-30a-3p | UUUCAGUCGGAUGUUUGCAGC | 9 | 33 | 26 | 21 | 2.7459 | 7.9322 | 7.9144 |
| 897 | mdo-miR-34a-3p | AAUCAGCAAGUAUACUGCCCUA | 0 | 1 | 0 | 22 | 0.0000 | 0.2294 | 0.0000 |
| 898 | mml-miR-106b-3p | ACCGCACUGUGGGUACUUGCUG | 224 | 364 | 261 | 22 | 65.2356 | 83.5171 | 75.8370 |
| 899 | mml-miR-127-5p | UGAAGCUCAGAGGGCUCUGAUU | 81 | 208 | 126 | 22 | 23.5896 | 47.7240 | 36.6110 |
| 900 | mml-miR-1296-5p | UUAGGGCCCUGGCUCCAUCUCCU | 10 | 11 | 9 | 23 | 2.7857 | 2.4141 | 2.5014 |
| 901 | mml-miR-145-3p | GGAUUCCUGGAAAUACUGUUCUU | 128 | 197 | 159 | 23 | 35.6567 | 43.2349 | 44.1909 |
| 902 | mml-miR-1911-5p | UGAGUACCGCCAUGUCUGUUGG | 1 | 6 | 7 | 22 | 0.2912 | 1.3767 | 2.0339 |
| 903 | mml-miR-195-3p | CCAAUAUUGGCUGUGCUGCUCCA | 177 | 156 | 142 | 23 | 49.3065 | 34.2368 | 39.4661 |
| 904 | mml-miR-26b-3p | CCUGUUCUCCAUUACUUGGCU | 0 | 1 | 1 | 21 | 0.0000 | 0.2404 | 0.3044 |
| 905 | mml-miR-299-3p | UAUGUGGGACGGUAAACCGCUU | 104 | 172 | 137 | 22 | 30.2879 | 39.4641 | 39.8071 |
| 906 | mml-miR-29c-5p | CCGAUUUCUCCUGGUGUUCAG | 4 | 5 | 5 | 21 | 1.2204 | 1.2018 | 1.5220 |
| 907 | mml-miR-3059-5p | UUUCCUCUCUGCCCCAUAGGGU | 0 | 1 | 0 | 22 | 0.0000 | 0.2294 | 0.0000 |
| 908 | mml-miR-30c-1-3p | UGGGAGAGGGUUGUUUACUCC | 3 | 6 | 4 | 21 | 0.9153 | 1.4422 | 1.2176 |
| 909 | mml-miR-376a-1-5p | GUAGAUUCUCCUUCUAUGAGUAC | 1 | 5 | 2 | 23 | 0.2786 | 1.0973 | 0.5559 |
| 910 | mml-miR-380-5p | AUGGUUGACCAUAGAACAUGCG | 0 | 1 | 2 | 22 | 0.0000 | 0.2294 | 0.5811 |
| 911 | mml-miR-382-3p | AAUCAUUCACGGACAACACUUUU | 254 | 570 | 382 | 23 | 70.7563 | 125.0960 | 106.1692 |
| 912 | mml-miR-412-5p | UGGUCGACCAGUUGGAAAGU | 106 | 258 | 129 | 20 | 33.9574 | 65.1158 | 41.2309 |
| 913 | mml-miR-424-3p | AAAACGUGAGGCGCUGCUAUA | 202 | 285 | 153 | 21 | 61.6298 | 68.5050 | 46.5731 |
| 914 | mml-miR-543-5p | GAAGUUGCCCGUGUCUUUUUCG | 0 | 2 | 1 | 22 | 0.0000 | 0.4589 | 0.2906 |
| 915 | mml-miR-7178-5p | UAGGAGCUAUCAGAACUUAGUG | 0 | 0 | 1 | 22 | 0.0000 | 0.0000 | 0.2906 |
| 916 | mml-miR-7180-3p | UGGCCUCUGGGUGUGUACCCU | 7 | 12 | 22 | 21 | 2.1357 | 2.8844 | 6.6968 |
| 917 | mml-miR-99b-3p | AAGCUCGUGUCUGUGGGUCCG | 39 | 55 | 54 | 21 | 11.8988 | 13.2203 | 16.4376 |
| 918 | mmu-miR-100-3p | ACAAGCUUGUGUCUAUAGGUAU | 1 | 3 | 6 | 22 | 0.2912 | 0.6883 | 1.7434 |
| 919 | mmu-miR-10b-3p | CAGAUUCGAUUCUAGGGGAAUA | 2 | 9 | 5 | 22 | 0.5825 | 2.0650 | 1.4528 |
| 920 | mmu-miR-1193-5p | UGGUAGACCGGUGACGUACA | 3 | 0 | 3 | 20 | 0.9611 | 0.0000 | 0.9589 |
| 921 | mmu-miR-1264-5p | AGGUCCUCAAUAAGUAUUUGUU | 0 | 6 | 4 | 22 | 0.0000 | 1.3767 | 1.1623 |
| 922 | mmu-miR-128-1-5p | CGGGGCCGUAGCACUGUCUGA | 0 | 0 | 1 | 21 | 0.0000 | 0.0000 | 0.3044 |
| 923 | mmu-miR-1298-3p | CAUCUGGGCAACUGAUUGAACU | 0 | 4 | 17 | 22 | 0.0000 | 0.9178 | 4.9396 |
| 924 | mmu-miR-132-5p | AACCGUGGCUUUCGAUUGUUAC | 4 | 7 | 5 | 22 | 1.1649 | 1.6061 | 1.4528 |
| 925 | mmu-miR-136-3p | AUCAUCGUCUCAAAUGAGUCUU | 263 | 791 | 406 | 22 | 76.5935 | 181.4890 | 117.9686 |
| 926 | mmu-miR-139-3p | UGGAGACGCGGCCCUGUUGGAG | 3 | 5 | 7 | 22 | 0.8737 | 1.1472 | 2.0339 |
| 927 | mmu-miR-144-5p | GGAUAUCAUCAUAUACUGUAAGU | 16 | 121 | 37 | 23 | 4.4571 | 26.5555 | 10.2834 |
| 928 | mmu-miR-145a-3p | AUUCCUGGAAAUACUGUUCUUG | 128 | 197 | 159 | 22 | 37.2775 | 45.2002 | 46.1995 |
| 929 | mmu-miR-1839-3p | AGACCUACUUAUCUACCAACAGC | 10 | 11 | 10 | 23 | 2.7857 | 2.4141 | 2.7793 |
| 930 | mmu-miR-1983 | CUCACCUGGAGCAUGUUUUCU | 361 | 267 | 386 | 21 | 110.1405 | 64.1783 | 117.4982 |
| 931 | mmu-miR-212-3p | UAACAGUCUCCAGUCACGGCCA | 0 | 1 | 5 | 22 | 0.0000 | 0.2294 | 1.4528 |
| 932 | mmu-miR-223-5p | CGUGUAUUUGACAAGCUGAGUUG | 1 | 0 | 2 | 23 | 0.2786 | 0.0000 | 0.5559 |
| 933 | mmu-miR-25-5p | AGGCGGAGACUUGGGCAAUUGC | 2 | 2 | 2 | 22 | 0.5825 | 0.4589 | 0.5811 |
| 934 | mmu-miR-29b-2-5p | CUGGUUUCACAUGGUGGCUUAGAUU | 0 | 1 | 1 | 25 | 0.0000 | 0.2019 | 0.2557 |
| 935 | mmu-miR-324-3p | CCACUGCCCCAGGUGCUGCU | 0 | 1 | 0 | 20 | 0.0000 | 0.2524 | 0.0000 |
| 936 | mmu-miR-370-5p | CAGGUCACGUCUCUGCAGUU | 0 | 1 | 1 | 20 | 0.0000 | 0.2524 | 0.3196 |
| 937 | mmu-miR-376a-5p | GGUAGAUUCUCCUUCUAUGAGU | 1 | 5 | 2 | 22 | 0.2912 | 1.1472 | 0.5811 |
| 938 | mmu-miR-378b | CUGGACUUGGAGUCAGAAGA | 19 | 34 | 29 | 20 | 6.0867 | 8.5811 | 9.2690 |
| 939 | mmu-miR-378c | ACUGGACUUGGAGUCAGAAGC | 60 | 84 | 97 | 21 | 18.3059 | 20.1909 | 29.5267 |
| 940 | mmu-miR-425-3p | AUCGGGAAUGUCGUGUCCGCC | 5 | 8 | 4 | 21 | 1.5255 | 1.9229 | 1.2176 |
| 941 | mmu-miR-485-3p | AGUCAUACACGGCUCUCCUCUC | 7 | 15 | 16 | 22 | 2.0386 | 3.4416 | 4.6490 |
| 942 | mmu-miR-493-5p | UUGUACAUGGUAGGCUUUC | 1242 | 2440 | 1674 | 19 | 418.8196 | 648.2354 | 563.2031 |
| 943 | mmu-miR-758-3p | UUUGUGACCUGGUCCACUA | 38 | 85 | 59 | 19 | 12.8141 | 22.5820 | 19.8500 |
| 944 | mse-let-7a | UGAGGUAGUAGGUUGUAUAG | 859 | 3075 | 2074 | 20 | 275.1834 | 776.0892 | 662.8907 |
| 945 | oan-miR-106-5p | AAAAGUGCUUACAGUGCAGGU | 2 | 1 | 2 | 21 | 0.6102 | 0.2404 | 0.6088 |
| 946 | oan-miR-143-5p | AGGUGCAGUGCUGCAUCUCUGGU | 89 | 178 | 132 | 23 | 24.7926 | 39.0651 | 36.6868 |
| 947 | oan-miR-145-5p | GUCCAGUUUUCCCAGGAAU | 0 | 1 | 0 | 19 | 0.0000 | 0.2657 | 0.0000 |
| 948 | oan-miR-205-5p | UCCUUCAUUCCACCGGAGUCU | 16 | 12 | 44 | 21 | 4.8816 | 2.8844 | 13.3936 |
| 949 | oan-miR-20a-5p | UAAAGUGCUUAUAGUGCAGG | 2313 | 1762 | 1341 | 20 | 740.9769 | 444.7054 | 428.6097 |
| 950 | oan-miR-214-5p | UGCCUGUCUACACUUGCUGU | 383 | 719 | 449 | 20 | 122.6953 | 181.4660 | 143.5091 |
| 951 | oan-miR-24-1-5p | UGCCUACUGAGCUGAUAUCA | 11 | 20 | 23 | 20 | 3.5239 | 5.0477 | 7.3512 |
| 952 | oan-miR-32-5p | UAUUGCACAUUACUAAGUUG | 85 | 155 | 81 | 20 | 27.2300 | 39.1199 | 25.8892 |
| 953 | oan-miR-454-5p | ACCCUAUCGAUAUUGUCUCUGCU | 5 | 2 | 0 | 23 | 1.3928 | 0.4389 | 0.0000 |
| 954 | oar-let-7d | AGAGGUAGUAGGUUGCAUAG | 336 | 548 | 355 | 20 | 107.6387 | 138.3079 | 113.4649 |
| 955 | oar-miR-10a | UACCCUGUAGAUCCGAAUUUG | 23 | 83 | 17 | 21 | 7.0173 | 19.9506 | 5.1748 |
| 956 | oar-miR-10b | ACCCUGUAGAACCGAAUUUGUG | 1773 | 2395 | 2131 | 22 | 516.3511 | 549.5147 | 619.1900 |
| 957 | oar-miR-1185-5p | AGAGGAUACCCUUUGUAUGUUC | 0 | 2 | 1 | 22 | 0.0000 | 0.4589 | 0.2906 |
| 958 | oar-miR-127 | AUCGGAUCCGUCUGAGCUUGGCU | 2457 | 5402 | 3868 | 23 | 684.4415 | 1185.5592 | 1075.0330 |
| 959 | oar-miR-154a-3p | AAUCAUACACGGUUCACCUAUU | 77 | 121 | 81 | 22 | 22.4247 | 27.7625 | 23.5356 |
| 960 | oar-miR-23b | AUCACAUUGCCAGGGAUU | 887 | 1685 | 1431 | 18 | 315.7259 | 472.5240 | 508.1949 |
| 961 | oar-miR-25 | AUUGCACUUGUCUCGGUCUGA | 577 | 923 | 598 | 21 | 176.0417 | 221.8599 | 182.0309 |
| 962 | oar-miR-30d | UGUAAACAUCCCCGACUGG | 2407 | 2842 | 2452 | 19 | 811.6738 | 755.0348 | 824.9546 |
| 963 | oar-miR-323b | CCCAAUACACGGUCGAUCUCU | 51 | 71 | 62 | 21 | 15.5600 | 17.0661 | 18.8728 |
| 964 | oar-miR-370-3p | GCCUGCUGGGGUGGAACCUGGUCU | 144 | 217 | 123 | 24 | 38.4424 | 45.6399 | 32.7610 |
| 965 | oar-miR-374b | AUAUAAUACAACCUGCUAAGU | 236 | 539 | 359 | 21 | 72.0032 | 129.5585 | 109.2794 |
| 966 | oar-miR-376a-5p | UAGAUUCUCCUUCUAUGAGUAC | 1 | 4 | 1 | 22 | 0.2912 | 0.9178 | 0.2906 |
| 967 | oar-miR-379-5p | UGGUAGACUAUGGAACGUAGGC | 1499 | 3613 | 2231 | 22 | 436.5540 | 828.9756 | 648.2463 |
| 968 | oar-miR-381-3p | AUAUACAAGGGCAAGCUCUCU | 422 | 494 | 386 | 21 | 128.7515 | 118.7419 | 117.4982 |
| 969 | oar-miR-3956-5p | GUACGUGGAUGCUGAAGGUCAGA | 85 | 152 | 56 | 23 | 23.6783 | 33.3589 | 15.5641 |
| 970 | oar-miR-3958-5p | AGGUUGUCCGUGAUGUAUUUGC | 2 | 3 | 5 | 22 | 0.5825 | 0.6883 | 1.4528 |
| 971 | oar-miR-409-3p | CGAAUGUUGCUCGGUGAACCCCU | 577 | 844 | 500 | 23 | 160.7337 | 185.2299 | 138.9650 |
| 972 | oar-miR-487b-5p | AGUGGUUAUCCCUGUCCUGUUC | 1 | 1 | 0 | 22 | 0.2912 | 0.2294 | 0.0000 |
| 973 | oar-miR-494-5p | AGGUUAUCCGUGUUGUCUUCUCU | 1 | 1 | 1 | 23 | 0.2786 | 0.2195 | 0.2779 |
| 974 | oar-miR-539-3p | AAUCAUACAAGGACAAUUUCUUU | 13 | 16 | 9 | 23 | 3.6214 | 3.5115 | 2.5014 |
| 975 | oar-miR-543-3p | AAACAUUCGCGGUGCACUUCUUU | 209 | 388 | 207 | 23 | 58.2207 | 85.1531 | 57.5315 |
| 976 | oar-miR-544-3p | AUUCUGCAUUUUUAGCAAGUU | 0 | 1 | 0 | 21 | 0.0000 | 0.2404 | 0.0000 |
| 977 | oar-miR-654-3p | UAUGUCUGCUGACCAUCACCUUUA | 174 | 332 | 260 | 24 | 46.4512 | 69.8270 | 69.2509 |
| 978 | oar-miR-665-3p | ACCAGUAGGCCGAGGCCCCUCA | 35 | 79 | 72 | 22 | 10.1931 | 18.1260 | 20.9205 |
| 979 | oar-miR-758-3p | UUUGUGACCUGGUCCACUAA | 38 | 85 | 59 | 20 | 12.1734 | 21.4529 | 18.8575 |
| 980 | oar-miR-99a | AACCCGUAGAUCCGAUCUUG | 13211 | 49497 | 22479 | 20 | 4232.1858 | 12492.3853 | 7184.7251 |
| 981 | ocu-miR-191-3p | GCUGCGCUUGGAUUUCGUUCCC | 10 | 7 | 3 | 22 | 2.9123 | 1.6061 | 0.8717 |
| 982 | oha-miR-10c-5p | UACCCUGUAGAACCGAAUUUGUGUG | 1773 | 2395 | 2131 | 25 | 454.3889 | 483.5729 | 544.8872 |
| 983 | oha-miR-212-5p | ACCUUGGCUCUAGACUGCUUACUG | 1 | 1 | 0 | 24 | 0.2670 | 0.2103 | 0.0000 |
| 984 | oha-miR-27b-5p | AGAGCUUAGCUGAUUGGUGAACAG | 88 | 37 | 57 | 24 | 23.4926 | 7.7819 | 15.1819 |
| 985 | oha-miR-30e-5p | UGUAAACAUCCUUGACUGGAAGCUG | 1019 | 1048 | 787 | 25 | 261.1519 | 211.6010 | 201.2324 |
| 986 | ola-miR-100 | AACCCGUAGAUCCGAACUU | 4072 | 10780 | 8648 | 19 | 1373.1349 | 2863.9250 | 2909.5461 |
| 987 | ola-miR-101a-3p | GUACAGUACUGUGAUAACUGA | 384 | 809 | 527 | 21 | 117.1577 | 194.4580 | 160.4185 |
| 988 | ola-miR-106a | UAAAGUGCUUACAGUGCAGGU | 1 | 0 | 5 | 21 | 0.3051 | 0.0000 | 1.5220 |
| 989 | ola-miR-126-3p | UCGUACCGUGAGUAAUAAUG | 3151 | 5557 | 4699 | 20 | 1009.4329 | 1402.5130 | 1501.8917 |
| 990 | ola-miR-140-3p | ACCACAGGGUAGAACCACGG | 3360 | 3341 | 3748 | 20 | 1076.3867 | 843.2240 | 1197.9336 |
| 991 | ola-miR-181a-5p | AACAUUCAACGCUGUCGGU | 405 | 1011 | 815 | 19 | 136.5716 | 268.5926 | 274.1998 |
| 992 | ola-miR-192-5p | UGACCUAUGAAUUGACAGCCA | 50 | 62 | 48 | 21 | 15.2549 | 14.9028 | 14.6112 |
| 993 | ola-miR-193a | AACUGGCCUACAAAGUCCC | 3 | 2 | 2 | 19 | 1.0116 | 0.5313 | 0.6729 |
| 994 | ola-miR-194-5p | UGUAACAGCAACUCCAUGU | 37 | 71 | 57 | 19 | 12.4769 | 18.8626 | 19.1772 |
| 995 | ola-miR-204 | UUCCCUUUGUCAUCCUAUGC | 104 | 472 | 229 | 20 | 33.3167 | 119.1265 | 73.1928 |
| 996 | ola-miR-20a | UAAAGUGCUUAUAGUGCAGGU | 2313 | 1762 | 1340 | 21 | 705.6923 | 423.5289 | 407.8953 |
| 997 | ola-miR-222 | AGCUACAUCUGGCUACUGG | 71 | 76 | 65 | 19 | 23.9422 | 20.1909 | 21.8687 |
| 998 | ola-miR-223 | UGUCAGUUUGUCAAAUACCC | 3 | 8 | 6 | 20 | 0.9611 | 2.0191 | 1.9177 |
| 999 | ola-miR-24b-3p | UGGCUCAGUUCAGCAGGA | 190 | 525 | 381 | 18 | 67.6301 | 147.2256 | 135.3056 |
| 1000 | ola-miR-27d-3p | UUCACAGUGGCUAAGUUC | 3 | 24 | 14 | 18 | 1.0678 | 6.7303 | 4.9719 |
| 1001 | ola-miR-29c | UAGCACCAUUUGAAAUCG | 1 | 2 | 1 | 18 | 0.3559 | 0.5609 | 0.3551 |
| 1002 | ola-miR-30a-3p | CUUUCAGUCGGAUGUUUGC | 1 | 8 | 5 | 19 | 0.3372 | 2.1254 | 1.6822 |
| 1003 | pma-miR-199a-3p | CAGUAGUCUGCACAUUGGUUA | 9197 | 29226 | 17182 | 21 | 2805.9887 | 7025.0040 | 5230.1915 |
| 1004 | pma-miR-20a-5p | CAAAGUGCUUAUAGUGCAGGUAG | 42 | 23 | 12 | 23 | 11.6999 | 5.0477 | 3.3352 |
| 1005 | ppy-miR-378d | ACUGGACUUGGAGUCAGA | 24 | 37 | 42 | 18 | 8.5428 | 10.3759 | 14.9156 |
| 1006 | ppy-miR-378e | ACUGGACUUGGAGUCAGG | 3 | 3 | 0 | 18 | 1.0678 | 0.8413 | 0.0000 |
| 1007 | prd-let-7-5p | UGAGGUAGUAGGUUGUAUAGUUU | 16495 | 30254 | 22382 | 23 | 4594.9788 | 6639.7458 | 6220.6279 |
| 1008 | ptr-miR-320d | AAAAGCUGGGUUGAGAGG | 0 | 4 | 1 | 18 | 0.0000 | 1.1217 | 0.3551 |
| 1009 | rno-miR-10b-3p | ACAGAUUCGAUUCUAGGGGAA | 2 | 9 | 5 | 21 | 0.6102 | 2.1633 | 1.5220 |
| 1010 | rno-miR-127-5p | CUGAAGCUCAGAGGGCUCUGAUU | 82 | 209 | 128 | 23 | 22.8426 | 45.8685 | 35.5750 |
| 1011 | rno-miR-1306-3p | GACGUUGGCUCUGGUGGUGAUG | 6 | 10 | 5 | 22 | 1.7474 | 2.2944 | 1.4528 |
| 1012 | rno-miR-145-3p | GGAUUCCUGGAAAUACUGUUC | 128 | 197 | 159 | 21 | 39.0526 | 47.3526 | 48.3995 |
| 1013 | rno-miR-15a-5p | UAGCAGCACAUAAUGGUUU | 20 | 34 | 28 | 19 | 6.7443 | 9.0328 | 9.4204 |
| 1014 | rno-miR-16-3p | ACCAAUAUUAUUGUGCUGCUU | 5 | 12 | 6 | 21 | 1.5255 | 2.8844 | 1.8264 |
| 1015 | rno-miR-18a-3p | ACUGCCCUAAGUGCUCCUUCU | 3 | 4 | 3 | 21 | 0.9153 | 0.9615 | 0.9132 |
| 1016 | rno-miR-204-3p | GCUGGGAAGGCAAAGGGACGUU | 4 | 29 | 14 | 22 | 1.1649 | 6.6538 | 4.0679 |
| 1017 | rno-miR-221-5p | ACCUGGCAUACAAUGUAGAUUUC | 46 | 39 | 48 | 23 | 12.8141 | 8.5592 | 13.3406 |
| 1018 | rno-miR-24-1-5p | GUGCCUACUGAGCUGAUAUCAG | 15 | 25 | 25 | 22 | 4.3685 | 5.7361 | 7.2641 |
| 1019 | rno-miR-299a-3p | UAUGUGGGACGGUAAACCGCU | 104 | 172 | 137 | 21 | 31.7302 | 41.3433 | 41.7027 |
| 1020 | rno-miR-370-5p | CAGGUCACGUCUCUGCAGUUACAC | 0 | 1 | 1 | 24 | 0.0000 | 0.2103 | 0.2663 |
| 1021 | rno-miR-376c-5p | GUGGAUAUUCCUUCUAUGUUU | 16 | 16 | 16 | 21 | 4.8816 | 3.8459 | 4.8704 |
| 1022 | rno-miR-379-3p | CUAUGUAACAUGGUCCACUAAC | 39 | 101 | 57 | 22 | 11.3580 | 23.1737 | 16.5621 |
| 1023 | rno-miR-409a-3p | AAUGUUGCUCGGUGAACCCC | 577 | 844 | 499 | 20 | 184.8438 | 213.0144 | 159.4901 |
| 1024 | rno-miR-411-3p | UAUGUAACACGGUCCACUAA | 57 | 139 | 69 | 20 | 18.2601 | 35.0818 | 22.0537 |
| 1025 | rno-miR-412-3p | CUUCACCUGGUCCACUAGCCGU | 5 | 3 | 1 | 22 | 1.4562 | 0.6883 | 0.2906 |
| 1026 | rno-miR-452-3p | UCAGUCUCAUCUGCAAAGAAG | 60 | 76 | 48 | 21 | 18.3059 | 18.2680 | 14.6112 |
| 1027 | rno-miR-452-5p | AACUGUUUGCAGAGGAAACUGAG | 1162 | 964 | 718 | 23 | 323.6960 | 211.5659 | 199.5537 |
| 1028 | rno-miR-98-3p | CUAUACAACUUACUACUUUCC | 2 | 1 | 1 | 21 | 0.6102 | 0.2404 | 0.3044 |
| 1029 | sha-miR-10b | UACCCUGUAGAACCGAAU | 1618 | 2186 | 1942 | 18 | 575.9238 | 613.0193 | 689.6677 |
| 1030 | sha-miR-126 | UCGUACCGUGAGUAAUAAU | 7951 | 12854 | 10535 | 19 | 2681.1875 | 3414.9251 | 3544.4112 |
| 1031 | sha-miR-181a-3p | ACCAUCGACCGUUGAUUGU | 141 | 88 | 145 | 19 | 47.5472 | 23.3790 | 48.7840 |
| 1032 | sha-miR-193 | AACUGGCCCACAAAGUCCCGC | 115 | 187 | 123 | 21 | 35.0863 | 44.9489 | 37.4411 |
| 1033 | sha-miR-21 | UAGCUUAUCAGACUGAUGUUGACUG | 215110 | 198623 | 164771 | 25 | 55128.9373 | 40103.8453 | 42131.2104 |
| 1034 | sha-miR-221 | AGCUACAUUGUCUGCUGGGUU | 146 | 206 | 175 | 21 | 44.5443 | 49.5159 | 53.2699 |
| 1035 | sha-miR-24 | UGGCUCAGUUCAGCAGGAA | 753 | 1212 | 980 | 19 | 253.9220 | 321.9923 | 329.7127 |
| 1036 | sha-miR-25 | CAUUGCACUUGUCUCGGUC | 577 | 923 | 598 | 19 | 194.5724 | 245.2136 | 201.1920 |
| 1037 | sha-miR-9 | UCUUUGGUUAUCUAGCUGUAU | 5 | 13 | 7 | 21 | 1.5255 | 3.1248 | 2.1308 |
| 1038 | sha-miR-92a | UAUUGCACUUGUCCCGGC | 1834 | 2576 | 1674 | 18 | 652.8086 | 722.3869 | 594.4921 |
| 1039 | sha-miR-93 | CAAAGUGCUGUUCGUGCAGGU | 563 | 567 | 424 | 21 | 171.7703 | 136.2888 | 129.0654 |
| 1040 | ssa-miR-144-3p | CUACAGUAUAGAUGAUGUAC | 0 | 2 | 0 | 20 | 0.0000 | 0.5048 | 0.0000 |
| 1041 | ssa-miR-144-5p | GGAUAUCAUCAUAUACUGUAAGUU | 6 | 54 | 17 | 24 | 1.6018 | 11.3574 | 4.5279 |
| 1042 | ssc-let-7i | UGAGGUAGUAGUUUGUGCU | 65670 | 114404 | 74010 | 19 | 22144.8348 | 30393.7367 | 24900.0357 |
| 1043 | ssc-miR-151-3p | CUAGACUGAAGCUCCUUGAGGA | 5134 | 7743 | 5496 | 22 | 1495.1756 | 1776.5730 | 1596.9349 |
| 1044 | ssc-miR-199b-5p | CCCAGUGUUUAGACUAUCUGUU | 15745 | 24978 | 17201 | 22 | 4585.4189 | 5731.0140 | 4997.9762 |
| 1045 | ssc-miR-296-3p | AGGGUUGGGCGGAGGCUUUCC | 91 | 97 | 94 | 21 | 27.7639 | 23.3157 | 28.6135 |
| 1046 | ssc-miR-299 | AUGGUUUACCGUCCCACAUAC | 7 | 18 | 11 | 21 | 2.1357 | 4.3266 | 3.3484 |
| 1047 | ssc-miR-30c-1-3p | CUGGGAGAGGGUUGUUUACU | 3 | 6 | 4 | 20 | 0.9611 | 1.5143 | 1.2785 |
| 1048 | ssc-miR-320 | AAAAGCUGGGUUGAGAGGGCGAA | 524 | 573 | 343 | 23 | 145.9696 | 125.7544 | 95.3300 |
| 1049 | ssc-miR-339 | UCCCUGUCCUCCAGGAGCUCA | 49 | 200 | 142 | 21 | 14.9498 | 48.0737 | 43.2247 |
| 1050 | ssc-miR-345-3p | CCCUGAACUAGGGGUCUGGAG | 6 | 13 | 6 | 21 | 1.8306 | 3.1248 | 1.8264 |
| 1051 | ssc-miR-361-3p | CCCCCAGGUGUGAUUCUGAUUUGC | 319 | 430 | 289 | 24 | 85.1606 | 90.4386 | 76.9750 |
| 1052 | ssc-miR-362 | AAUCCUUGGAACCUAGGUGUGAGUG | 110 | 132 | 113 | 25 | 28.1911 | 26.6520 | 28.8936 |
| 1053 | ssc-miR-382 | AAGUUGUUCGUGGUGGAUUCG | 242 | 622 | 284 | 21 | 73.8338 | 149.5091 | 86.4494 |
| 1054 | ssc-miR-411 | AUGUAACACGGUCCACUAAC | 57 | 139 | 69 | 20 | 18.2601 | 35.0818 | 22.0537 |
| 1055 | ssc-miR-432-5p | UCUUGGAGUAGGUCAUUGGGU | 54 | 119 | 70 | 21 | 16.4753 | 28.6038 | 21.3080 |
| 1056 | ssc-miR-542-5p | UCGGGGAUCAUCAUGUCACGA | 5 | 13 | 9 | 21 | 1.5255 | 3.1248 | 2.7396 |
| 1057 | ssc-miR-652 | ACAACCCUAGGAGAGGGUGCCAUUCA | 11 | 11 | 18 | 26 | 2.7107 | 2.1356 | 4.4255 |
| 1058 | ssc-miR-758 | UUUGUGACCUGGUCCACUAAC | 38 | 85 | 59 | 21 | 11.5937 | 20.4313 | 17.9596 |
| 1059 | ssc-miR-7857-3p | AUUGUUCUCCAACCUGGCUCUU | 135 | 114 | 173 | 22 | 39.3161 | 26.1564 | 50.2674 |
| 1060 | tch-miR-140-5p | CAGUGGUUUUACCCUAUGGUA | 1176 | 849 | 788 | 21 | 358.7956 | 204.0727 | 239.8668 |
| 1061 | tch-miR-194-5p | UGUAACAGCAACUCCAUGUGGAA | 37 | 71 | 57 | 23 | 10.3070 | 15.5821 | 15.8420 |
| 1062 | tch-miR-23b-3p | AUCACAUUGCCAGGGAUUACCACG | 1036 | 1881 | 1599 | 24 | 276.5716 | 395.6162 | 425.8929 |
| 1063 | tch-miR-30a-5p | UGUAAACAUCCUCGACUGGA | 687 | 1992 | 1370 | 20 | 220.0826 | 502.7543 | 437.8786 |
| 1064 | tch-miR-361-5p | UUAUCAGAAUCUCCAGGGGUA | 47 | 99 | 90 | 21 | 14.3396 | 23.7965 | 27.3960 |
| 1065 | tch-miR-374b-5p | AUAUAAUACAACCUGCUAAGUGU | 235 | 539 | 359 | 23 | 65.4635 | 118.2926 | 99.7768 |
| 1066 | tgu-miR-126-5p | CAUUAUUACUUUUGGUACGC | 64 | 131 | 101 | 20 | 20.5026 | 33.0627 | 32.2816 |
| 1067 | tgu-miR-138-1-3p | GCUACUUCACAACACCAGGGU | 1 | 0 | 2 | 21 | 0.3051 | 0.0000 | 0.6088 |
| 1068 | tgu-miR-155-5p | UUAAUGCUAAUCGUGAUAGGG | 811 | 961 | 935 | 21 | 247.4347 | 230.9939 | 284.6135 |
| 1069 | tgu-miR-181b-1-3p | GCUCACUGAACAAUGAAUGCA | 3 | 7 | 7 | 21 | 0.9153 | 1.6826 | 2.1308 |
| 1070 | tgu-miR-29a-1-5p | ACUGAUUUCUUUUGGUGUUCAGA | 1 | 1 | 1 | 23 | 0.2786 | 0.2195 | 0.2779 |
| 1071 | tgu-miR-29b-2-5p | UGGUUUCACAUGGUGGCUUAGA | 0 | 1 | 1 | 22 | 0.0000 | 0.2294 | 0.2906 |
| 1072 | tgu-miR-365-1-5p | GGGACUUUUGGGGGCAGAUGUGU | 86 | 40 | 54 | 23 | 23.9568 | 8.7787 | 15.0082 |
| 1073 | CM008008.1_10340 | AAAAACCAGAACGAACUUUGUG | 41 | 47 | 18 | 22 | 11.9404 | 10.7838 | 5.2301 |
| 1074 | CM008008.1_5534 | AAAAAGUUCGUUUGGGUUUUCC | 2 | 3 | 4 | 22 | 0.5825 | 0.6883 | 1.1623 |
| 1075 | CM008008.1_6671 | AAAAAGUUCGUUUGGGUUUUCC | 3 | 2 | 4 | 22 | 0.8737 | 0.4589 | 1.1623 |
| 1076 | CM008008.1_7149 | AAAAGUUUGUUCAGGUUUUUCU | 3 | 1 | 1 | 22 | 0.8737 | 0.2294 | 0.2906 |
| 1077 | CM008008.1_9244 | UUGACUGUGUUUCACUGAAGU | 137 | 120 | 125 | 21 | 41.7985 | 28.8442 | 38.0499 |
| 1078 | CM008008.1_9743 | GGACUGUAGCCUGCCAGGG | 2 | 7 | 4 | 19 | 0.6744 | 1.8597 | 1.3458 |
| 1079 | CM008009.1_14936 | CUCCCACCCCGCCUCCCGC | 7 | 13 | 9 | 19 | 2.3605 | 3.4537 | 3.0280 |
| 1080 | CM008009.1_15028 | UCAGUCCUUCUCCAACUCCAGA | 4 | 12 | 4 | 22 | 1.1649 | 2.7533 | 1.1623 |
| 1081 | CM008009.1_16740 | AAAACCUGAACGAACUUUUG | 2 | 0 | 1 | 20 | 0.6407 | 0.0000 | 0.3196 |
| 1082 | CM008009.1_16903 | UCUGAGACGUGAUCUUGUUUUG | 1 | 2 | 6 | 22 | 0.2912 | 0.4589 | 1.7434 |
| 1083 | CM008010.1_17978 | ACUGUGGCUCAGAUCAUGAAUU | 8 | 8 | 7 | 22 | 2.3298 | 1.8355 | 2.0339 |
| 1084 | CM008010.1_21055 | CGAGGCACUUAACUCUGGGCU | 6 | 0 | 0 | 21 | 1.8306 | 0.0000 | 0.0000 |
| 1085 | CM008010.1_21891 | AAGACCCGAAUGAACUUUUU | 1 | 2 | 2 | 20 | 0.3204 | 0.5048 | 0.6392 |
| 1086 | CM008010.1_26918 | AAAACCUGAACGAACUUUUG | 2 | 0 | 1 | 20 | 0.6407 | 0.0000 | 0.3196 |
| 1087 | CM008011.1_31347 | UUCGGGCCGUCUCUGCCUUGCAG | 9 | 6 | 1 | 23 | 2.5071 | 1.3168 | 0.2779 |
| 1088 | CM008011.1_33842 | CCCCGCCUCCCGCUCUCC | 4 | 8 | 43 | 18 | 1.4238 | 2.2434 | 15.2707 |
| 1089 | CM008011.1_37433 | UGAGUGUGUGUGUGUGAGC | 5 | 23 | 9 | 19 | 1.6861 | 6.1104 | 3.0280 |
| 1090 | CM008011.1_37670 | UAUGAUGGUUAAAGGGAGCUGG | 10 | 6 | 10 | 22 | 2.9123 | 1.3767 | 2.9056 |
| 1091 | CM008012.1_42107 | UAAUCUAGAAGAACCCUAUGCU | 3 | 3 | 1 | 22 | 0.8737 | 0.6883 | 0.2906 |
| 1092 | CM008012.1_42960 | AAAAGAUUUGUUCGGGUUUUUC | 1 | 2 | 2 | 22 | 0.2912 | 0.4589 | 0.5811 |
| 1093 | CM008012.1_43443 | UGGAAAAACCCAAACGAACUUC | 2 | 8 | 1 | 22 | 0.5825 | 1.8355 | 0.2906 |
| 1094 | CM008012.1_46261 | UGAGUGUGUGUGUGUGAGC | 12 | 33 | 18 | 19 | 4.0466 | 8.7671 | 6.0559 |
| 1095 | CM008012.1_46537 | AUAACCGUAACUUUGAAAUGCU | 0 | 5 | 13 | 22 | 0.0000 | 1.1472 | 3.7773 |
| 1096 | CM008012.1_47852 | AAAAGUUUGUUCAGGUUUUUCU | 3 | 2 | 1 | 22 | 0.8737 | 0.4589 | 0.2906 |
| 1097 | CM008012.1_48395 | AGAUGUGAUGACCUUCUGAGG | 30 | 10 | 14 | 21 | 9.1529 | 2.4037 | 4.2616 |
| 1098 | CM008012.1_48925 | UUAGGUGAGCAAAGGCUGCAUC | 0 | 4 | 4 | 22 | 0.0000 | 0.9178 | 1.1623 |
| 1099 | CM008012.1_49180 | AAAAAAGUUCGUUUGGGUUUU | 0 | 2 | 0 | 21 | 0.0000 | 0.4807 | 0.0000 |
| 1100 | CM008012.1_50670 | ACUACGGGAUAAGAGGGCCAGCU | 1 | 6 | 2 | 23 | 0.2786 | 1.3168 | 0.5559 |
| 1101 | CM008012.1_52630 | CCGUCCAGUGGUUAAGACUCUG | 21 | 22 | 15 | 22 | 6.1158 | 5.0477 | 4.3584 |
| 1102 | CM008012.1_53957 | AAAAGUUCAUUAGGGUUUUUCA | 0 | 2 | 0 | 22 | 0.0000 | 0.4589 | 0.0000 |
| 1103 | CM008012.1_55199 | ACUUCUGUACGUAUCUGAUGCAG | 2 | 4 | 0 | 23 | 0.5571 | 0.8779 | 0.0000 |
| 1104 | CM008012.1_55392 | AGAACCUGAACAAACUUUUUGG | 2 | 7 | 2 | 22 | 0.5825 | 1.6061 | 0.5811 |
| 1105 | CM008012.1_55433 | GUGGGCUUCCCUGGUGGCUCAGA | 6 | 85 | 632 | 23 | 1.6714 | 18.6547 | 175.6517 |
| 1106 | CM008012.1_56259 | GUUAUCAGGAUUUAGUGAGAU | 4 | 3 | 0 | 21 | 1.2204 | 0.7211 | 0.0000 |
| 1107 | CM008012.1_61980 | UCUUCCGGAUCGUCCUUACAGC | 7 | 8 | 4 | 22 | 2.0386 | 1.8355 | 1.1623 |
| 1108 | CM008012.1_62189 | UGGACCACCAGGGAAUUCCAUU | 2 | 5 | 1 | 22 | 0.5825 | 1.1472 | 0.2906 |
| 1109 | CM008012.1_63603 | UCACUUCUACUCUGGGUCUGCA | 60 | 66 | 60 | 22 | 17.4738 | 15.1432 | 17.4338 |
| 1110 | CM008013.1_67139 | AAAAAGUUUGUUGGAGUUUUUCU | 1 | 3 | 3 | 23 | 0.2786 | 0.6584 | 0.8338 |
| 1111 | CM008013.1_68686 | UGGAAAGACCCAAAUGAACUUU | 5 | 8 | 3 | 22 | 1.4562 | 1.8355 | 0.8717 |
| 1112 | CM008014.1_69773 | AAGCUCGAAUGAACUUUUUGGC | 3 | 3 | 3 | 22 | 0.8737 | 0.6883 | 0.8717 |
| 1113 | CM008014.1_74801 | UAGGAAUUAGAAGGUGCACGCUG | 4 | 6 | 6 | 23 | 1.1143 | 1.3168 | 1.6676 |
| 1114 | CM008014.1_76350 | AAAACGAGAAUGAACUUUUUGG | 2 | 2 | 2 | 22 | 0.5825 | 0.4589 | 0.5811 |
| 1115 | CM008015.1_78022 | GUGCUGUUUUCUCUUCAGUC | 0 | 0 | 10 | 20 | 0.0000 | 0.0000 | 3.1962 |
| 1116 | CM008015.1_81455 | AAAAGGUCGUUCCAGUUUUCUG | 9 | 2 | 3 | 22 | 2.6211 | 0.4589 | 0.8717 |
| 1117 | CM008015.1_83294 | UGGGAAAGAAUCUGCCUGC | 0 | 3 | 36 | 19 | 0.0000 | 0.7970 | 12.1119 |
| 1118 | CM008016.1_100323 | AAAAACCCAAAUGAACUUUUUG | 7 | 10 | 5 | 22 | 2.0386 | 2.2944 | 1.4528 |
| 1119 | CM008016.1_100327 | AAAAAGUUCGUUUGGGUUUUUU | 0 | 2 | 2 | 22 | 0.0000 | 0.4589 | 0.5811 |
| 1120 | CM008016.1_84545 | GUGGGCUUCCCUGGUGGCGCAGAC | 1 | 5 | 27 | 24 | 0.2670 | 1.0516 | 7.1914 |
| 1121 | CM008016.1_87034 | AAGUAACUUAACACCAGCUCUGG | 18 | 22 | 21 | 23 | 5.0142 | 4.8283 | 5.8365 |
| 1122 | CM008016.1_88050 | UGCAUGCUGUUGUGUCCAUCU | 2 | 5 | 0 | 21 | 0.6102 | 1.2018 | 0.0000 |
| 1123 | CM008016.1_89428 | UUUGAUGGCUGUUCCUCUCACU | 6 | 7 | 11 | 22 | 1.7474 | 1.6061 | 3.1962 |
| 1124 | CM008016.1_90056 | CUCUUACUGUCAUCCCUGCUUUA | 6 | 8 | 8 | 23 | 1.6714 | 1.7557 | 2.2234 |
| 1125 | CM008016.1_90073 | UCAGCUUCAGCAUCAGUCCUUU | 2 | 3 | 10 | 22 | 0.5825 | 0.6883 | 2.9056 |
| 1126 | CM008016.1_90401 | UGGCUGUACAACUGGUUCAUUGAC | 17 | 12 | 13 | 24 | 4.5383 | 2.5239 | 3.4625 |
| 1127 | CM008016.1_93249 | UUGCAGGUGUGGGCAGCAGCACU | 2 | 4 | 2 | 23 | 0.5571 | 0.8779 | 0.5559 |
| 1128 | CM008016.1_95226 | UAUUUUCAUGACAUUUUUCUGA | 13 | 3 | 10 | 22 | 3.7860 | 0.6883 | 2.9056 |
| 1129 | CM008016.1_96188 | AGAACCUGAACAAACUUUUUGG | 2 | 6 | 2 | 22 | 0.5825 | 1.3767 | 0.5811 |
| 1130 | CM008016.1_97064 | UGGAAAGACCCAAAUGAACUUU | 4 | 7 | 2 | 22 | 1.1649 | 1.6061 | 0.5811 |
| 1131 | CM008016.1_98387 | UCCUAGGGGAUUGACAUAGUCUG | 19 | 20 | 17 | 23 | 5.2928 | 4.3893 | 4.7248 |
| 1132 | CM008016.1_98716 | UAUCUGUGGUUUCUGCAU | 50 | 65 | 37 | 18 | 17.7974 | 18.2279 | 13.1399 |
| 1133 | CM008017.1_101998 | AUAUCAAAGAGCUGUAAGUGUU | 13 | 11 | 11 | 22 | 3.7860 | 2.5239 | 3.1962 |
| 1134 | CM008017.1_104971 | AGGGGAGGGCCUGGGAAGCUGU | 5 | 1 | 0 | 22 | 1.4562 | 0.2294 | 0.0000 |
| 1135 | CM008018.1_110103 | AAGGCCUGAACGAACUUUUUGG | 5 | 6 | 8 | 22 | 1.4562 | 1.3767 | 2.3245 |
| 1136 | CM008018.1_113821 | GCUAUGCGACUGAGAGCCAGU | 8 | 5 | 2 | 21 | 2.4408 | 1.2018 | 0.6088 |
| 1137 | CM008018.1_117444 | AUCCCACUUCUGACACCAAG | 24 | 58 | 63 | 20 | 7.6885 | 14.6384 | 20.1360 |
| 1138 | CM008018.1_119020 | AAGGCCUGAAUGAACUUUUUG | 5 | 8 | 4 | 21 | 1.5255 | 1.9229 | 1.2176 |
| 1139 | CM008018.1_120835 | AAAAAGUUCGUUUGGGUUUUCC | 2 | 3 | 4 | 22 | 0.5825 | 0.6883 | 1.1623 |
| 1140 | CM008019.1_128870 | UUUGAUGGCUGUUCCUCUCACU | 6 | 7 | 11 | 22 | 1.7474 | 1.6061 | 3.1962 |
| 1141 | CM008019.1_130001 | UAAUUGGGGCUUAUCUGCAGA | 2 | 7 | 0 | 21 | 0.6102 | 1.6826 | 0.0000 |
| 1142 | CM008019.1_130171 | UGGAAAGACCCAAAUGAACUUU | 4 | 7 | 2 | 22 | 1.1649 | 1.6061 | 0.5811 |
| 1143 | CM008019.1_131018 | AAAAAGUUCGUUUGGGUUUUCA | 1 | 0 | 3 | 22 | 0.2912 | 0.0000 | 0.8717 |
| 1144 | CM008019.1_131447 | AAAACACGAACAAACUUUUCGG | 38 | 77 | 22 | 22 | 11.0667 | 17.6671 | 6.3924 |
| 1145 | CM008019.1_131998 | AAAACCUGAACUAACUUUUUG | 11 | 5 | 3 | 21 | 3.3561 | 1.2018 | 0.9132 |
| 1146 | CM008019.1_134217 | AAAAGUUUGGACGGUUUUUUCC | 3 | 6 | 2 | 22 | 0.8737 | 1.3767 | 0.5811 |
| 1147 | CM008019.1_137729 | CCCAGGGAUGUAGCUCCUAGUGC | 5 | 20 | 4 | 23 | 1.3928 | 4.3893 | 1.1117 |
| 1148 | CM008019.1_138018 | AAAACCCAACGGACUUUUUGGC | 4 | 0 | 5 | 22 | 1.1649 | 0.0000 | 1.4528 |
| 1149 | CM008019.1_138754 | AAAACCCGGACGAACCUUUUGG | 4 | 0 | 4 | 22 | 1.1649 | 0.0000 | 1.1623 |
| 1150 | CM008020.1_141593 | AAGUACACAAACAAUUCCAAGU | 2 | 7 | 6 | 22 | 0.5825 | 1.6061 | 1.7434 |
| 1151 | CM008020.1_143049 | UCUGGGCCACCUAGUCGCCAAC | 5 | 2 | 1 | 22 | 1.4562 | 0.4589 | 0.2906 |
| 1152 | CM008020.1_143067 | UGGUGGGCACAGAAUCCGGC | 1 | 6 | 5 | 20 | 0.3204 | 1.5143 | 1.5981 |
| 1153 | CM008020.1_143069 | ACGCACAGCGCCUCACUGAGC | 10 | 38 | 24 | 21 | 3.0510 | 9.1340 | 7.3056 |
| 1154 | CM008020.1_143075 | CACAAUACACGGGUCGGCCUCU | 93 | 51 | 65 | 22 | 27.0844 | 11.7016 | 18.8866 |
| 1155 | CM008020.1_143606 | UGAGUGUGUGUGUGAGUGUGU | 37 | 58 | 50 | 21 | 11.2886 | 13.9414 | 15.2200 |
| 1156 | CM008020.1_145771 | AGUCAGAAUCUCUUGGACAAGGCU | 12 | 8 | 12 | 24 | 3.2035 | 1.6826 | 3.1962 |
| 1157 | CM008020.1_145882 | AGCUUUCUGUAGCAGAGAUCACC | 0 | 0 | 4 | 23 | 0.0000 | 0.0000 | 1.1117 |
| 1158 | CM008020.1_147241 | AAAAAGUUCAUUUGGGUUGUUU | 1 | 5 | 7 | 22 | 0.2912 | 1.1472 | 2.0339 |
| 1159 | CM008020.1_147456 | UCACUGGGUAUCCUCUGCUUUA | 51 | 58 | 31 | 22 | 14.8527 | 13.3077 | 9.0075 |
| 1160 | CM008021.1_147678 | AAAACUCAAACGAACUUUUUGG | 40 | 51 | 66 | 22 | 11.6492 | 11.7016 | 19.1772 |
| 1161 | CM008021.1_148608 | UUGGCCUACAGAAAUGACAGACA | 13 | 27 | 6 | 23 | 3.6214 | 5.9256 | 1.6676 |
| 1162 | CM008021.1_148609 | CACAUGGAGUUGCUGUUACAAU | 14 | 13 | 13 | 22 | 4.0772 | 2.9828 | 3.7773 |
| 1163 | CM008021.1_149700 | UUCUUGACUGCUCACCUGGGC | 113 | 182 | 119 | 21 | 34.4761 | 43.7470 | 36.2235 |
| 1164 | CM008021.1_150571 | GCACGUGAUGUGUGGGCUGAUG | 43 | 12 | 12 | 22 | 12.5229 | 2.7533 | 3.4868 |
| 1165 | CM008021.1_151843 | UAACUGUAUAGUUACCCUCUUU | 43 | 51 | 44 | 22 | 12.5229 | 11.7016 | 12.7848 |
| 1166 | CM008021.1_154122 | AAAAAACCGAGUGAACUUUUUG | 121 | 265 | 84 | 22 | 35.2388 | 60.8023 | 24.4073 |
| 1167 | CM008021.1_154130 | AAGCUCGAAUGAACUUUUUGGC | 1 | 2 | 1 | 22 | 0.2912 | 0.4589 | 0.2906 |
| 1168 | CM008022.1_158829 | AAAACCUGAACGAACUUUUG | 2 | 0 | 1 | 20 | 0.6407 | 0.0000 | 0.3196 |
| 1169 | CM008022.1_159134 | CAAAUUCGUGAAGCGUUCCAUAUUU | 71 | 31 | 57 | 25 | 18.1961 | 6.2592 | 14.5746 |
| 1170 | CM008022.1_159625 | AAACCUCAGUGAACUUUCUGGC | 4 | 15 | 4 | 22 | 1.1649 | 3.4416 | 1.1623 |
| 1171 | CM008022.1_160011 | UGCGGUAGAGCAUCAGAGAACU | 220 | 417 | 316 | 22 | 64.0706 | 95.6775 | 91.8179 |
| 1172 | CM008022.1_160361 | GCUGGCAGGACUGUAGAGGUUU | 3 | 7 | 5 | 22 | 0.8737 | 1.6061 | 1.4528 |
| 1173 | CM008022.1_164169 | UAACUCUGAGUAACCUAACUGG | 11 | 10 | 11 | 22 | 3.2035 | 2.2944 | 3.1962 |
| 1174 | CM008022.1_165248 | AAAAGUUGGUUCGAGUUUUUCU | 3 | 2 | 3 | 22 | 0.8737 | 0.4589 | 0.8717 |
| 1175 | CM008022.1_166506 | AUGCGGUAGAGCAUCAGAGA | 3 | 23 | 9 | 20 | 0.9611 | 5.8049 | 2.8766 |
| 1176 | CM008022.1_167031 | AAAAAAGUUCGUUUGGGUUUU | 0 | 2 | 0 | 21 | 0.0000 | 0.4807 | 0.0000 |
| 1177 | CM008022.1_167390 | UCUGGUGGGAAGGAAGGGACACA | 3 | 5 | 7 | 23 | 0.8357 | 1.0973 | 1.9455 |
| 1178 | CM008022.1_171045 | CUGGAGCUCUGGGUUCCUGCUUC | 25 | 3 | 6 | 23 | 6.9642 | 0.6584 | 1.6676 |
| 1179 | CM008022.1_171200 | UGGAAAGACCCAAAUGAACUU | 3 | 5 | 0 | 21 | 0.9153 | 1.2018 | 0.0000 |
| 1180 | CM008023.1_171704 | AAAAAGUUUGUUUGGGUUUUCU | 0 | 1 | 1 | 22 | 0.0000 | 0.2294 | 0.2906 |
| 1181 | CM008023.1_172014 | UGGUCCUGGAGUGCUUUGGCAGC | 5 | 1 | 2 | 23 | 1.3928 | 0.2195 | 0.5559 |
| 1182 | CM008023.1_173900 | AAAACCUGAACAAACUUUUUGG | 1 | 1 | 2 | 22 | 0.2912 | 0.2294 | 0.5811 |
| 1183 | CM008023.1_177547 | AAAAGUUCAUUCGGGUUCCUCU | 0 | 4 | 9 | 22 | 0.0000 | 0.9178 | 2.6151 |
| 1184 | CM008024.1_180123 | UAUAUAUAUAUAUGUACGUAU | 20 | 43 | 30 | 21 | 6.1020 | 10.3358 | 9.1320 |
| 1185 | CM008024.1_183606 | AAAACCUGAACUAACUUUUUGA | 37 | 23 | 17 | 22 | 10.7755 | 5.2772 | 4.9396 |
| 1186 | CM008025.1_184228 | CAAAUUCGUGAAGCGUUCCAUAUUU | 71 | 31 | 58 | 25 | 18.1961 | 6.2592 | 14.8303 |
| 1187 | CM008025.1_184735 | ACUGCCUGGAUAAGAAUCAGC | 2 | 5 | 12 | 21 | 0.6102 | 1.2018 | 3.6528 |
| 1188 | CM008025.1_185853 | AAAACUUGAAUGAACUUUUUGG | 5 | 4 | 3 | 22 | 1.4562 | 0.9178 | 0.8717 |
| 1189 | CM008025.1_186278 | AAAAAAGUUCGUUUGGGUUUU | 0 | 2 | 0 | 21 | 0.0000 | 0.4807 | 0.0000 |
| 1190 | CM008025.1_186565 | AAAAGUUCAUUUGGGUUUUUCC | 2 | 0 | 0 | 22 | 0.5825 | 0.0000 | 0.0000 |
| 1191 | CM008025.1_188371 | AAACCCUGAACGAACUUUUUGG | 2 | 5 | 3 | 22 | 0.5825 | 1.1472 | 0.8717 |
| 1192 | CM008025.1_188962 | AACUUUUGCCCCUAGUAACGGACU | 504 | 597 | 541 | 24 | 134.5483 | 125.5624 | 144.0951 |
| 1193 | CM008025.1_192294 | AGAACCUGAACAAACUUUUUGG | 2 | 6 | 1 | 22 | 0.5825 | 1.3767 | 0.2906 |
| 1194 | CM008025.1_193290 | CAGGCGGGUGCUGAUGCGAUC | 28 | 39 | 12 | 21 | 8.5428 | 9.3744 | 3.6528 |
| 1195 | CM008025.1_195252 | AACUGCUGCCUAAACUCAGGGC | 6 | 2 | 2 | 22 | 1.7474 | 0.4589 | 0.5811 |
| 1196 | CM008026.1_198184 | CAAGCAGGAUUUAGACUACAAUA | 53 | 97 | 50 | 23 | 14.7641 | 21.2883 | 13.8965 |
| 1197 | CM008026.1_201442 | CACCGGCAUCGUGAUGGACU | 0 | 3 | 9 | 20 | 0.0000 | 0.7572 | 2.8766 |
| 1198 | CM008026.1_202979 | CCGGAGAUGAAUUCCUUACAC | 2 | 6 | 0 | 21 | 0.6102 | 1.4422 | 0.0000 |
| 1199 | CM008026.1_208845 | AAAUCCUGAACGAACUUUUUGG | 2 | 3 | 2 | 22 | 0.5825 | 0.6883 | 0.5811 |
| 1200 | CM008027.1_209108 | UUGAGCCUGCGCUUUAGAAGCUG | 6 | 3 | 8 | 23 | 1.6714 | 0.6584 | 2.2234 |
| 1201 | CM008027.1_210382 | UGAUAUGUUUGAUAUUGAGUUG | 3 | 9 | 5 | 22 | 0.8737 | 2.0650 | 1.4528 |
| 1202 | CM008027.1_210664 | UGGGGGUUGAGAAUGUCGCU | 1 | 1 | 1 | 20 | 0.3204 | 0.2524 | 0.3196 |
| 1203 | CM008027.1_214124 | AGCGACCCCACGGGCUGGAGCC | 4 | 4 | 1 | 22 | 1.1649 | 0.9178 | 0.2906 |
| 1204 | CM008027.1_214244 | UACGACUACAGGAUUUCACUGA | 86 | 71 | 51 | 22 | 25.0458 | 16.2904 | 14.8187 |
| 1205 | CM008027.1_215433 | AAAACCUGAACGAACUUUUUAG | 0 | 0 | 5 | 22 | 0.0000 | 0.0000 | 1.4528 |
| 1206 | CM008027.1_215935 | AAAAACCUGAAUGACCCUUUU | 104 | 283 | 154 | 21 | 31.7302 | 68.0242 | 46.8775 |
| 1207 | CM008027.1_215941 | AAAAACCUAAACGAACUUUUUG | 5 | 9 | 5 | 22 | 1.4562 | 2.0650 | 1.4528 |
| 1208 | CM008027.1_216002 | AAUGUUCUAUGUCUUGAUUGU | 4 | 2 | 5 | 21 | 1.2204 | 0.4807 | 1.5220 |
| 1209 | CM008027.1_219971 | ACUGGGGGUUGAGAAUGUCGCU | 1 | 9 | 1 | 22 | 0.2912 | 2.0650 | 0.2906 |
| 1210 | CM008028.1_228985 | AAAACACGAACAAACUUU | 0 | 1 | 1 | 18 | 0.0000 | 0.2804 | 0.3551 |
| 1211 | CM008028.1_229104 | AAAAAGUUCGUUUGGGUUUUCC | 2 | 3 | 4 | 22 | 0.5825 | 0.6883 | 1.1623 |
| 1212 | CM008028.1_232670 | AGGAGUUGGACACGACUGGGCG | 7 | 3 | 7 | 22 | 2.0386 | 0.6883 | 2.0339 |
| 1213 | CM008028.1_233163 | AAACCCCGAAUGAACUUUUU | 1 | 1 | 0 | 20 | 0.3204 | 0.2524 | 0.0000 |
| 1214 | CM008028.1_233164 | AAACCCCGAAUGAACUUUUU | 1 | 1 | 0 | 20 | 0.3204 | 0.2524 | 0.0000 |
| 1215 | CM008028.1_234298 | AGAACCCGAAUGAACUUUUUGA | 13 | 6 | 13 | 22 | 3.7860 | 1.3767 | 3.7773 |
| 1216 | CM008029.1_236542 | UCUGAACAACGGACUAUUCUGC | 15 | 15 | 7 | 22 | 4.3685 | 3.4416 | 2.0339 |
| 1217 | CM008029.1_236567 | ACGCCCUUCCCCCCCUUCUUCA | 21 | 13 | 25 | 22 | 6.1158 | 2.9828 | 7.2641 |
| 1218 | CM008029.1_238965 | AAAACCUGAACUAACUUUUUGA | 35 | 22 | 15 | 22 | 10.1931 | 5.0477 | 4.3584 |
| 1219 | CM008029.1_241215 | AUGUCCGCAGGUUCCCUAUCCC | 118 | 164 | 139 | 22 | 34.3652 | 37.6286 | 40.3883 |
| 1220 | CM008029.1_242977 | AAAAUCUGAAUGAACUUUUUGA | 3 | 2 | 0 | 22 | 0.8737 | 0.4589 | 0.0000 |
| 1221 | CM008030.1_244854 | AAGAAGUUUGUUUGGGUGUUUCU | 10 | 10 | 3 | 23 | 2.7857 | 2.1947 | 0.8338 |
| 1222 | CM008030.1_244939 | UCAAAUGAACUUUUUAGCGACC | 1 | 5 | 5 | 22 | 0.2912 | 1.1472 | 1.4528 |
| 1223 | CM008030.1_245504 | AAAGUUCGUUCAGGUUUUUCUA | 2 | 1 | 8 | 22 | 0.5825 | 0.2294 | 2.3245 |
| 1224 | CM008030.1_246513 | CUUGCUGGGUGACCUCCCUGC | 1 | 4 | 3 | 21 | 0.3051 | 0.9615 | 0.9132 |
| 1225 | CM008030.1_248613 | AAACCCCGAAUGAACUUUUU | 3 | 5 | 0 | 20 | 0.9611 | 1.2619 | 0.0000 |
| 1226 | CM008030.1_248867 | AAAGCCUGAAUGAACUUUUUGG | 4 | 3 | 3 | 22 | 1.1649 | 0.6883 | 0.8717 |
| 1227 | CM008030.1_250130 | CAUUGCGUUUGGAACCUCGGC | 4 | 7 | 3 | 21 | 1.2204 | 1.6826 | 0.9132 |
| 1228 | CM008030.1_251217 | AAAAAGUUCGUUUGGGUUUUCC | 3 | 2 | 4 | 22 | 0.8737 | 0.4589 | 1.1623 |
| 1229 | CM008030.1_251835 | AAAAACCUGAACGAACUUUUGG | 1 | 3 | 1 | 22 | 0.2912 | 0.6883 | 0.2906 |
| 1230 | CM008030.1_255444 | UCCUUUAGGAUGGACUGGUUGGA | 9 | 20 | 6 | 23 | 2.5071 | 4.3893 | 1.6676 |
| 1231 | CM008031.1_259110 | AAAAGAUUUGUUCGGGUUUUUC | 1 | 2 | 2 | 22 | 0.2912 | 0.4589 | 0.5811 |
| 1232 | CM008031.1_262970 | AAAGCCUGAACGAACUUUUUGU | 8 | 6 | 2 | 22 | 2.3298 | 1.3767 | 0.5811 |
| 1233 | CM008031.1_263955 | AAAAGUUUGUUCAGGUUUUUCU | 3 | 1 | 1 | 22 | 0.8737 | 0.2294 | 0.2906 |
| 1234 | CM008032.1_269285 | AAAAGUUAGCUCGGAUUUUUCU | 10 | 2 | 3 | 22 | 2.9123 | 0.4589 | 0.8717 |
| 1235 | CM008032.1_270582 | AAGGCCUGAAUGAACUUUUUGG | 9 | 5 | 6 | 22 | 2.6211 | 1.1472 | 1.7434 |
| 1236 | CM008033.1_275385 | AGGACUGAUACUGAAGCUAAGG | 13 | 24 | 9 | 22 | 3.7860 | 5.5066 | 2.6151 |
| 1237 | CM008033.1_276646 | GGGGCUUCCCUGGUGGCUCAGA | 1 | 4 | 29 | 22 | 0.2912 | 0.9178 | 8.4263 |
| 1238 | CM008034.1_282887 | UCAGUCGUGUCCAACUCUUUGU | 17 | 28 | 14 | 22 | 4.9509 | 6.4244 | 4.0679 |
| 1239 | CM008034.1_284828 | GUGGGCUUCCCUGGUAGCUCAGC | 11 | 28 | 287 | 23 | 3.0642 | 6.1451 | 79.7659 |
| 1240 | CM008034.1_285336 | AUGAACUUUUUGGCCAACCCAGA | 2 | 6 | 1 | 23 | 0.5571 | 1.3168 | 0.2779 |
| 1241 | CM008035.1_291664 | UUUCUUUUUCCUUUCACUUCAA | 1 | 3 | 3 | 22 | 0.2912 | 0.6883 | 0.8717 |
| 1242 | CM008036.1_292007 | AAAAAGUUCGUUUGGGUUUUCC | 2 | 2 | 4 | 22 | 0.5825 | 0.4589 | 1.1623 |
| 1243 | CM008036.1_292916 | UGAAGAAGUUCGUUUGGGUUUU | 37 | 60 | 34 | 22 | 10.7755 | 13.7665 | 9.8791 |
| 1244 | CM008036.1_294342 | AAAAACCUGAACGAACUUUUGGG | 2 | 3 | 2 | 23 | 0.5571 | 0.6584 | 0.5559 |
| 1245 | CM008036.1_295794 | AACAACUCGGAUGAACUUUUUG | 4 | 1 | 7 | 22 | 1.1649 | 0.2294 | 2.0339 |
| 1246 | CM008037.1_299589 | UGAGUGUGUGUGUGUGAGUGUGA | 1225 | 1537 | 1135 | 23 | 341.2458 | 337.3203 | 315.4505 |
| 1247 | CM008037.1_300926 | AAAAAGUUUGUUGGAGUUUUUCU | 1 | 3 | 3 | 23 | 0.2786 | 0.6584 | 0.8338 |
| 1248 | CM008037.1_301443 | AAAACACGAACAAACUUU | 0 | 1 | 1 | 18 | 0.0000 | 0.2804 | 0.3551 |
| 1249 | CM008037.1_302471 | AAGGCCUGAAUGAACUUUUUGG | 9 | 5 | 6 | 22 | 2.6211 | 1.1472 | 1.7434 |
| 1250 | CM008037.1_302513 | GUGGGCUUCCCUGGUGGCUCAGA | 6 | 85 | 631 | 23 | 1.6714 | 18.6547 | 175.3738 |
| 1251 | CM008038.1_306877 | UAGAUGAAAAGAUCUCAGGACU | 24 | 14 | 21 | 22 | 6.9895 | 3.2122 | 6.1018 |
| 1252 | CM008038.1_307359 | AAAAUCUGAAUGAACUUUUUGA | 12 | 9 | 4 | 22 | 3.4948 | 2.0650 | 1.1623 |
| 1253 | CM008038.1_307839 | CCGGUUUCUGUUGCCAAGGCGCC | 8 | 0 | 0 | 23 | 2.2285 | 0.0000 | 0.0000 |
| 1254 | CM008038.1_309862 | AGGAACUGACCAAUGAGUUGU | 4 | 0 | 2 | 21 | 1.2204 | 0.0000 | 0.6088 |
| 1255 | CM008038.1_311195 | UUUGGACUUGACAUUGCAUGUUU | 4 | 5 | 0 | 23 | 1.1143 | 1.0973 | 0.0000 |
| 1256 | CM008039.1_313741 | AUCCCGGACGAGCCCCCC | 14 | 26 | 40 | 18 | 4.9833 | 7.2912 | 14.2053 |
| 1257 | CM008039.1_315920 | CGGAUCAGCUCAGUGCCGGGC | 0 | 13 | 0 | 21 | 0.0000 | 3.1248 | 0.0000 |
| 1258 | CM008040.1_317341 | GACAUGACUGAGUGACUUUCACU | 27 | 53 | 10 | 23 | 7.5213 | 11.6317 | 2.7793 |
| 1259 | CM008040.1_317809 | AAAAUCUGAAUGAACUUUUUGA | 3 | 2 | 0 | 22 | 0.8737 | 0.4589 | 0.0000 |
| 1260 | CM008040.1_322358 | UAUGCUGUGUAGAUAUUUAAAC | 15 | 18 | 12 | 22 | 4.3685 | 4.1300 | 3.4868 |
| 1261 | CM008040.1_323630 | AAGACCCGAAUGAACUUUUUGG | 5 | 11 | 6 | 22 | 1.4562 | 2.5239 | 1.7434 |
| 1262 | CM008041.1_325871 | AAAAACCCAAACGAGCUCUUUG | 3 | 5 | 4 | 22 | 0.8737 | 1.1472 | 1.1623 |
| 1263 | CM008041.1_326111 | AGAACCUGAACAAACUUUUUGG | 2 | 6 | 1 | 22 | 0.5825 | 1.3767 | 0.2906 |
| 1264 | CM008041.1_326329 | AUUGGGGUGCACGUGUCUCUUUCA | 3 | 8 | 2 | 24 | 0.8009 | 1.6826 | 0.5327 |
| 1265 | CM008041.1_326625 | UGAUUGGUACUUCUUAGAGUGA | 5 | 15 | 4 | 22 | 1.4562 | 3.4416 | 1.1623 |
| 1266 | CM008041.1_329257 | AAAACCUGAAUGAGCUUUUUGGA | 5 | 7 | 7 | 23 | 1.3928 | 1.5363 | 1.9455 |
| 1267 | CM008041.1_329491 | GUGGGCUUCCCUGGUGGCUCAGA | 6 | 85 | 631 | 23 | 1.6714 | 18.6547 | 175.3738 |
| 1268 | CM008041.1_329711 | AAAAACCUGAACGAACUUUUGG | 0 | 2 | 2 | 22 | 0.0000 | 0.4589 | 0.5811 |
| 1269 | CM008041.1_332283 | AAGAUGUAUGAUGUGAUGAUUU | 82 | 82 | 38 | 22 | 23.8809 | 18.8143 | 11.0414 |
| 1270 | CM008041.1_334231 | AAAAAGUUCGUUUGGGUUUUCA | 1 | 0 | 3 | 22 | 0.2912 | 0.0000 | 0.8717 |
| 1271 | CM008041.1_334512 | UAAAUCCGAACGAACUUUUU | 326 | 275 | 264 | 20 | 104.4351 | 69.4063 | 84.3795 |
| 1272 | CM008041.1_334666 | AAAACACGAACAAACUUU | 0 | 1 | 1 | 18 | 0.0000 | 0.2804 | 0.3551 |
| 1273 | MKHE01000127.1_335921 | UGACUGCUGUCUCUCCUUCCCAGU | 0 | 5 | 1 | 24 | 0.0000 | 1.0516 | 0.2663 |
| 1274 | MKHE01004964.1_338157 | CUGCACUGCAUGGUAUCUGC | 10 | 25 | 6 | 20 | 3.2035 | 6.3097 | 1.9177 |
| 1275 | MKHE01008022.1_338752 | AAGGGCGCUUUUCUGUGGAGA | 10 | 19 | 18 | 21 | 3.0510 | 4.5670 | 5.4792 |
| 1276 | MKHE01008352.1_338794 | AUCUCACUGGAGCCUCCA | 13 | 11 | 15 | 18 | 4.6273 | 3.0847 | 5.3270 |
| 1277 | xtr-miR-92b | UAUUGCACUCGUCCCGGCCUC | 31 | 29 | 17 | 21 | 9.4580 | 6.9707 | 5.1748 |
