# Supplementary material for: RNA sequencing-based identification of microRNAs in the antler cartilage of Gansu red deer (Cervus elaphus kansuensis)
Source: PeerJ. 2022 Sep 21;10:e13947. doi: 10.7717/peerj.13947 (PMC9508884; doi:10.7717/peerj.13947)
Supplement: Table S4 [file peerj-10-13947-s005.docx]

**Supplementary Table S4 log2fold changes of all miRNAs**

| #miRNA | TPM-30 d | TPM-60 d | TPM-90 d | 30d_vs_60d  P value | 30d_vs_60d  log2FC | 60d_vs_d90  P value | 60d_vs_90d  log2FC | 30d_vs_90d  P value | 30d_vs_90d  log2FC |
| --- | --- | --- | --- | --- | --- | --- | --- | --- | --- |
| aae-miR-100 | 1173.948774 | 2441.038427 | 2484.314653 | 0 | 1.056125549 | 0.005553446 | 0.025352905 | 1.089813771 | 1.081478454 |
| aae-miR-125-5p | 284.240636 | 851.0021172 | 541.0285245 | 0 | 1.582049899 | 0 | -0.653458062 | 0.936927002 | 0.928591837 |
| aae-miR-133 | 4.077222237 | 4.12996444 | 5.52069923 | 0.084677256 | 0.018542764 | 0.077461386 | 0.418721645 | 0.445591505 | 0.437264409 |
| aae-miR-33 | 0.305098263 | 1.201841504 | 1.217597825 | 0.085038376 | 1.977900779 | 0.167574911 | 0.018791038 | 2.005057116 | 1.996691817 |
| aae-miR-7 | 1199.792285 | 530.2315699 | 688.9883172 | 0 | -1.178090184 | 0 | 0.377856949 | -0.791898056 | -0.800233235 |
| aae-miR-92a-3p | 3.050982627 | 6.009207519 | 5.479190213 | 0.020433414 | 0.977900779 | 0.094334775 | -0.133212056 | 0.85299622 | 0.844688724 |
| aae-miR-9a | 1.392839895 | 2.853067222 | 1.945509568 | 0.051613846 | 1.034484308 | 0.101711574 | -0.552365663 | 0.490402688 | 0.482118644 |
| aca-let-7a-3p | 1.525491313 | 2.644051308 | 2.43519565 | 0.074343502 | 0.793476208 | 0.126273985 | -0.118712486 | 0.683091739 | 0.674763722 |
| aca-let-7a-5p | 4806.462558 | 6942.470224 | 6505.417635 | 0 | 0.530473589 | 0 | -0.093807406 | 0.445001465 | 0.436666183 |
| aca-let-7c-1-3p | 1.456150799 | 6.653831598 | 6.101825465 | 0.000282022 | 2.192025585 | 0.090288381 | -0.12494444 | 2.075388641 | 2.067081145 |
| aca-let-7c-2-3p | 0 | 2.753309627 | 1.162252469 | 0.001056766 | 1.461166859 | 0.060119616 | -1.244243368 | 23.4820999 | 0.216923491 |
| aca-let-7c-5p | 1357.715005 | 4152.679245 | 2536.906578 | 0 | 1.612861763 | 0 | -0.710972049 | 0.910225 | 0.901889714 |
| aca-let-7d-5p | 102.5130163 | 131.7218288 | 108.061807 | 3.45E-05 | 0.361687345 | 0.000846 | -0.285637736 | 0.084384632 | 0.076049609 |
| aca-let-7f-1-3p | 42.92732556 | 36.09130036 | 42.50938407 | 0.016382 | -0.250245169 | 0.024643527 | 0.236130231 | -0.005779894 | -0.014114938 |
| aca-let-7f-2-3p | 3.966277415 | 3.12478791 | 3.652793475 | 0.07992777 | -0.344027316 | 0.11106765 | 0.225241915 | -0.110455095 | -0.1187854 |
| aca-let-7f-5p | 7665.177806 | 9048.063761 | 8357.466945 | 0 | 0.239289838 | 0 | -0.114543352 | 0.133081788 | 0.124746486 |
| aca-let-7g | 11016.36326 | 12755.39517 | 11463.00554 | 0 | 0.211459559 | 0 | -0.154122235 | 0.065672609 | 0.057337324 |
| aca-let-7i-3p | 1.392839895 | 4.608800897 | 3.891019137 | 0.005402066 | 1.726362012 | 0.096088965 | -0.244243368 | 1.490437866 | 1.482118644 |
| aca-let-7i-5p | 19125.08459 | 26249.13621 | 21504.86688 | 0 | 0.456803819 | 0 | -0.287606748 | 0.177532365 | 0.169197071 |
| aca-miR-101-3p | 122.05456 | 202.6665328 | 167.1609614 | 0 | 0.731581674 | 0.000151976 | -0.277869909 | 0.46204762 | 0.453711766 |
| aca-miR-103-3p | 325.3040885 | 391.428852 | 330.9513907 | 0 | 0.266961145 | 0 | -0.242130767 | 0.033165989 | 0.024830378 |
| aca-miR-107-3p | 53.29511925 | 70.66828042 | 63.92388582 | 0.000363431 | 0.407059387 | 0.023103839 | -0.144707694 | 0.270686145 | 0.262351692 |
| aca-miR-10a-5p | 6.128495537 | 17.99627017 | 4.724808952 | 0 | 1.55409307 | 0 | -1.929370031 | -0.366928358 | -0.37527696 |
| aca-miR-10b-3p | 0.640706352 | 2.271480442 | 1.598097145 | 0.042078378 | 1.825897686 | 0.126492745 | -0.507277774 | 1.327059086 | 1.318619912 |
| aca-miR-10b-5p | 516.3510733 | 549.514713 | 619.1900031 | 0.001217459 | 0.089805804 | 1.86E-05 | 0.17222407 | 0.270365178 | 0.262029874 |
| aca-miR-126-3p | 2315.571001 | 2949.253495 | 3061.082441 | 0 | 0.348981837 | 0.000346091 | 0.053692069 | 0.411009162 | 0.402673906 |
| aca-miR-126-5p | 19.52628881 | 31.4882474 | 30.74434508 | 0.00039479 | 0.689395686 | 0.051154407 | -0.034492386 | 0.663234043 | 0.6549033 |
| aca-miR-128-3p | 50.34121334 | 43.98739904 | 32.26634237 | 0.018489718 | -0.194649692 | 0.003321037 | -0.447060251 | -0.633375387 | -0.641709942 |
| aca-miR-129a-5p | 2.135687839 | 2.163314707 | 11.56717934 | 0.108581841 | 0.018542764 | 0 | 2.418721645 | 2.445604465 | 2.437264409 |
| aca-miR-129b-3p | 0.305098263 | 0 | 1.826396738 | 0.207164791 | 1.71265413 | 0.011973116 | 0.869000188 | 2.590060659 | 2.581654318 |
| aca-miR-1306 | 1.747380959 | 2.294424689 | 1.452815587 | 0.098271658 | 0.392938279 | 0.112092073 | -0.659280867 | -0.257985786 | -0.266342589 |
| aca-miR-133a | 4.077222237 | 4.12996444 | 5.52069923 | 0.084677256 | 0.018542764 | 0.077461386 | 0.418721645 | 0.445591505 | 0.437264409 |
| aca-miR-135-5p | 0.278567979 | 0.438933419 | 0.277929938 | 0.195628342 | 0.655972684 | 0.247312928 | -0.659280867 | 0.004933982 | -0.003308183 |
| aca-miR-138-5p | 3.203531758 | 2.753309627 | 9.588582873 | 0.090672795 | -0.218496433 | 0.000149902 | 1.800150751 | 1.589970799 | 1.581654318 |
| aca-miR-140-3p | 1025.435261 | 803.3108611 | 1141.193562 | 0 | -0.352206125 | 0 | 0.506513225 | 0.162642363 | 0.1543071 |
| aca-miR-140-5p | 376.7353347 | 214.2763217 | 251.8601101 | 0 | -0.814078917 | 0.00028678 | 0.233150209 | -0.572593421 | -0.580928708 |
| aca-miR-143-3p | 3995.566848 | 7090.384136 | 6620.383775 | 0 | 0.827463599 | 0 | -0.09894894 | 0.736849922 | 0.728514659 |
| aca-miR-143-5p | 27.15374538 | 42.78555754 | 40.18072823 | 9.96E-05 | 0.655972684 | 0.042305166 | -0.090620179 | 0.573690387 | 0.565352506 |
| aca-miR-144-3p | 1.281412703 | 12.87172251 | 4.794291436 | 0 | 3.328398026 | 0.000398829 | -1.424815614 | 1.911865198 | 1.903582413 |
| aca-miR-144-5p | 1.830589576 | 12.97988824 | 5.174790757 | 0 | 2.825897686 | 0.000516584 | -1.326705528 | 1.507525784 | 1.499192158 |
| aca-miR-145-3p | 39.05257762 | 47.35255525 | 48.09511409 | 0.009177948 | 0.278024504 | 0.043792577 | 0.022448061 | 0.3088097 | 0.300472565 |
| aca-miR-145-5p | 391.1221046 | 327.6438456 | 340.2494104 | 0 | -0.255490641 | 0.01548019 | 0.054464229 | -0.192691183 | -0.201026412 |
| aca-miR-146a-5p | 1.525491313 | 7.932153925 | 1.521997281 | 5.32E-05 | 2.378438709 | 0.000113885 | -2.381746892 | 0.005032146 | -0.003308183 |
| aca-miR-148a-3p | 218579.8841 | 178561.0776 | 203518.2526 | 0 | -0.291742998 | 0 | 0.18874055 | -0.09466716 | -0.103002447 |
| aca-miR-150-5p | 3.494761918 | 10.55435357 | 6.682951699 | 0.000148384 | 1.59457214 | 0.023967469 | -0.659280867 | 0.94362238 | 0.935291273 |
| aca-miR-155-5p | 236.7701199 | 220.7236551 | 272.8387672 | 0.007984843 | -0.101245774 | 0 | 0.305807397 | 0.212896946 | 0.204561623 |
| aca-miR-16a-5p | 4.484944461 | 9.843081916 | 8.629724586 | 0.003637006 | 1.134019981 | 0.075844005 | -0.189795584 | 0.952560134 | 0.944224397 |
| aca-miR-17-3p | 0.873690479 | 1.376654813 | 1.452815587 | 0.115981432 | 0.655972684 | 0.153188595 | 0.077684727 | 0.742096585 | 0.733657411 |
| aca-miR-17-5p | 179.9549144 | 131.6800256 | 117.8422939 | 0 | -0.45059898 | 0.008576485 | -0.160179103 | -0.602442855 | -0.610778083 |
| aca-miR-181a | 149.3124367 | 243.6080474 | 254.5838235 | 0 | 0.706227455 | 0.016252962 | 0.06357896 | 0.778141652 | 0.769806415 |
| aca-miR-181b | 106.8814687 | 80.76374906 | 115.6441207 | 5.63E-05 | -0.40423195 | 0 | 0.517912135 | 0.122015346 | 0.113680185 |
| aca-miR-182-5p | 4.077222237 | 1.376654813 | 5.230136112 | 0.01211819 | -1.566419737 | 0.003237778 | 1.925681633 | 0.367597633 | 0.359261897 |
| aca-miR-184-3p | 25.3370239 | 23.40313183 | 52.59192424 | 0.039621626 | -0.114545469 | 0 | 1.168139678 | 1.061931234 | 1.053594208 |
| aca-miR-18a-3p | 0.873690479 | 0.917769876 | 0.871689352 | 0.147179596 | 0.071010184 | 0.185895435 | -0.074318367 | 0.005098158 | -0.003308183 |
| aca-miR-18a-5p | 13.09269501 | 6.584001282 | 5.836528705 | 0.001346258 | -0.991725572 | 0.086620553 | -0.17385404 | -1.157246879 | -1.165579612 |
| aca-miR-190a-5p | 2.228543832 | 2.194667094 | 0.833789815 | 0.104090671 | -0.022099221 | 0.065634915 | -1.396246461 | -1.41000112 | -1.418345682 |
| aca-miR-191-5p | 254.8897007 | 300.6693919 | 273.4830593 | 7.39E-06 | 0.238304929 | 0.002371997 | -0.13672654 | 0.109913694 | 0.101578389 |
| aca-miR-193-5p | 15.25491313 | 23.07535687 | 20.39476357 | 0.003631807 | 0.597078995 | 0.046770942 | -0.178154178 | 0.427256983 | 0.418924818 |
| aca-miR-194-5p | 10.77551591 | 16.29041529 | 16.56209769 | 0.008521797 | 0.596266438 | 0.064889463 | 0.023862027 | 0.628454661 | 0.620128466 |
| aca-miR-199a-5p | 2999.42102 | 4298.025586 | 3612.003948 | 0 | 0.518990028 | 0 | -0.250874601 | 0.276450703 | 0.268115426 |
| aca-miR-19a-3p | 32.03531758 | 10.78379604 | 13.94702963 | 0 | -1.570798177 | 0.041188114 | 0.371092782 | -1.191375436 | -1.199705396 |
| aca-miR-19b | 72.91848478 | 39.18003302 | 40.78952714 | 0 | -0.896165969 | 0.044923331 | 0.058080169 | -0.829749004 | -0.8380858 |
| aca-miR-1a-3p | 498.0035733 | 293.9158027 | 551.4887967 | 0 | -0.760753165 | 0 | 0.907928649 | 0.155510717 | 0.147175484 |
| aca-miR-200a-3p | 2.621071438 | 0.917769876 | 6.973514817 | 0.035314737 | -1.513952317 | 1.86E-05 | 2.925681633 | 1.420080743 | 1.411729316 |
| aca-miR-200b-3p | 2.329841278 | 0.688327407 | 5.811262347 | 0.03341018 | -1.759064815 | 7.84E-05 | 3.077684727 | 1.326972682 | 1.318619912 |
| aca-miR-204a-3p | 1.220393051 | 6.970680722 | 4.261592388 | 9.70E-05 | 2.51395368 | 0.041841021 | -0.70990694 | 1.812341251 | 1.804046739 |
| aca-miR-204a-5p | 34.94761918 | 116.0978893 | 72.0596531 | 0 | 1.732075664 | 0 | -0.688078132 | 1.052332386 | 1.043997532 |
| aca-miR-205a | 4.659682557 | 2.753309627 | 12.78477716 | 0.04398532 | -0.759064815 | 0 | 2.215188251 | 1.464471729 | 1.456123436 |
| aca-miR-20a-5p | 673.6153596 | 404.2776302 | 389.6451404 | 0 | -0.736578657 | 0.013048105 | -0.053185554 | -0.781428924 | -0.789764212 |
| aca-miR-212-5p | 0.29123016 | 0.229442469 | 0 | 0.227853908 | -0.344027316 | 0.315638314 | 2.123795642 | -21.47704278 | 1.779768326 |
| aca-miR-214-3p | 282.493255 | 437.0879033 | 403.59217 | 0 | 0.62970703 | 0.002115646 | -0.115025266 | 0.523017262 | 0.514681764 |
| aca-miR-214-5p | 111.5411512 | 165.1985776 | 130.4628397 | 0 | 0.566625199 | 2.94E-05 | -0.340562329 | 0.234398023 | 0.22606287 |
| aca-miR-21-5p | 62645.35476 | 45572.5515 | 47874.92259 | 0 | -0.45904239 | 0 | 0.071105008 | -0.379602091 | -0.387937381 |
| aca-miR-218-5p | 103.0954766 | 94.75973966 | 140.0514226 | 0.014793708 | -0.121634894 | 0 | 0.563610498 | 0.450311355 | 0.441975603 |
| aca-miR-221-3p | 42.51960333 | 47.2651486 | 50.84854554 | 0.021091694 | 0.152648653 | 0.036892302 | 0.105429717 | 0.266414929 | 0.25807837 |
| aca-miR-222a-3p | 64.94432564 | 41.07020193 | 51.72023489 | 3.82E-06 | -0.661111438 | 0.006958934 | 0.332636786 | -0.320139053 | -0.328474652 |
| aca-miR-23a-3p | 558.0247224 | 676.6367666 | 929.6359395 | 0 | 0.278052532 | 0 | 0.458284273 | 0.744672222 | 0.736336804 |
| aca-miR-23b-3p | 270.622159 | 405.0205868 | 435.5956219 | 0 | 0.581715266 | 0.003846072 | 0.104994213 | 0.695044598 | 0.68670948 |
| aca-miR-23b-5p | 1.830589576 | 0.721104902 | 2.43519565 | 0.07063155 | -1.344027316 | 0.043987577 | 1.755756632 | 0.420073807 | 0.411729316 |
| aca-miR-24-3p | 219.2963103 | 278.0842723 | 284.751855 | 0 | 0.342640613 | 0.020420914 | 0.034183088 | 0.385159058 | 0.376823702 |
| aca-miR-26-5p | 3676.373045 | 3980.138508 | 4663.56709 | 0 | 0.114535471 | 0 | 0.228615235 | 0.351485972 | 0.343150706 |
| aca-miR-27a-3p | 267.2660781 | 566.5480849 | 575.0105729 | 0 | 1.083921668 | 0.016353684 | 0.021390076 | 1.113647088 | 1.105311744 |
| aca-miR-27b-3p | 7525.38733 | 12280.90815 | 12104.85947 | 0 | 0.706579505 | 0.000331856 | -0.020830919 | 0.694083872 | 0.685748587 |
| aca-miR-27b-5p | 25.62825406 | 8.48937135 | 16.56209769 | 0 | -1.594005569 | 0.001148341 | 0.964155781 | -0.621516458 | -0.629849787 |
| aca-miR-29a-3p | 0.305098263 | 0.480736602 | 0.304399456 | 0.195628342 | 0.655972684 | 0.247312928 | -0.659280867 | 0.004933982 | -0.003308183 |
| aca-miR-29b | 0 | 0.240368301 | 0.304399456 | 0.25085307 | -2.056681446 | 0.286090674 | 0.340719133 | 21.48197676 | -1.715962313 |
| aca-miR-301a-3p | 0.278567979 | 0.658400128 | 0 | 0.152183516 | 1.240935185 | 0.12613315 | 0.602963478 | -21.47704278 | 1.843898663 |
| aca-miR-30a-3p | 2.621071438 | 7.571601474 | 7.554641051 | 0.001397232 | 1.530441802 | 0.086925753 | -0.003235268 | 1.535564274 | 1.527206534 |
| aca-miR-30a-5p | 200.0751198 | 457.508283 | 398.9431601 | 0 | 1.19325609 | 1.86E-05 | -0.197614652 | 1.003977029 | 0.995641439 |
| aca-miR-30b-5p | 93.4848813 | 186.0778423 | 192.0622206 | 0 | 0.993101302 | 0.022992933 | 0.04566749 | 1.047104706 | 1.038768792 |
| aca-miR-30c-3p | 0.58246032 | 2.294424689 | 2.324504939 | 0.030415169 | 1.977900779 | 0.129388308 | 0.018791038 | 2.005118679 | 1.996691817 |
| aca-miR-30c-5p | 125.395386 | 125.9529896 | 106.2354102 | 0.021768352 | 0.006401102 | 0.002440523 | -0.245620643 | -0.230883992 | -0.23921954 |
| aca-miR-30d-3p | 0 | 0.240368301 | 0.304399456 | 0.25085307 | -2.056681446 | 0.286090674 | 0.340719133 | 21.48197676 | -1.715962313 |
| aca-miR-30d-5p | 702.1559153 | 653.4521515 | 714.2041425 | 0.000888661 | -0.10370982 | 0.000202054 | 0.128254902 | 0.032880351 | 0.024545082 |
| aca-miR-30e-3p | 29.41424614 | 31.20417577 | 33.4147585 | 0.035820767 | 0.085224043 | 0.044992652 | 0.098746342 | 0.192306181 | 0.183970385 |
| aca-miR-30e-5p | 283.8607706 | 230.0011114 | 218.7308615 | 0 | -0.30354265 | 0.015634189 | -0.072484043 | -0.367691014 | -0.376026693 |
| aca-miR-32-5p | 24.75456358 | 35.56358268 | 23.53561251 | 0.001294654 | 0.522706154 | 0.00112193 | -0.59555527 | -0.064512283 | -0.072849116 |
| aca-miR-338-3p | 0.305098263 | 1.201841504 | 0.608798913 | 0.085038376 | 1.977900779 | 0.147080773 | -0.981208962 | 1.005180239 | 0.996691817 |
| aca-miR-34b-5p | 20.74668186 | 40.62224283 | 24.96075542 | 0 | 0.969389279 | 0.000151976 | -0.702608299 | 0.275118645 | 0.266780981 |
| aca-miR-365-3p | 67.56539708 | 40.611317 | 46.78066189 | 0 | -0.734402761 | 0.024218927 | 0.204030461 | -0.522036441 | -0.5303723 |
| aca-miR-365-5p | 4.457087663 | 1.097333547 | 3.057229322 | 0.002624756 | -2.022099221 | 0.034434148 | 1.478222656 | -0.535517078 | -0.543876564 |
| aca-miR-429-3p | 0.873690479 | 0.458884938 | 2.615068056 | 0.13744665 | -0.928989816 | 0.016057377 | 2.510644134 | 1.590060659 | 1.581654318 |
| aca-miR-451-5p | 977.6596465 | 5100.964969 | 2479.956207 | 0 | 2.383365982 | 0 | -1.040455549 | 1.351245684 | 1.342910433 |
| aca-miR-454-3p | 4.178519684 | 5.925601154 | 6.114458644 | 0.049239528 | 0.503969591 | 0.092237797 | 0.045263249 | 0.557565822 | 0.54923284 |
| aca-miR-499-5p | 2.038611119 | 2.06498222 | 1.162252469 | 0.108581841 | 0.018542764 | 0.109511215 | -0.829205869 | -0.80236841 | -0.810663105 |
| aca-miR-92a | 534.1161131 | 591.0437999 | 486.4026585 | 3.16E-05 | 0.146111639 | 0 | -0.281113933 | -0.126666799 | -0.135002294 |
| aca-miR-9-5p | 1.601765879 | 3.281027305 | 2.237336004 | 0.051613846 | 1.034484308 | 0.101711574 | -0.552365663 | 0.490402688 | 0.482118644 |
| aca-miR-98-5p | 296.5555113 | 431.4610999 | 306.225853 | 0 | 0.54092831 | 0 | -0.494634406 | 0.054629272 | 0.046293903 |
| aca-miR-99a-3p | 2.242472231 | 8.328761621 | 6.072769153 | 0.00035091 | 1.893011882 | 0.05833991 | -0.455747473 | 1.445591505 | 1.437264409 |
| aca-miR-99a-5p | 3847.441641 | 11356.71388 | 6531.568315 | 0 | 1.561574096 | 0 | -0.798044096 | 0.77186529 | 0.76353 |
| aga-miR-10 | 0 | 0.721104902 | 0 | 0.09860269 | -0.471718945 | 0.12613315 | 0.471718945 | -- | 0 |
| aga-miR-184 | 0.305098263 | 5.047734316 | 0.608798913 | 6.59E-05 | 4.048290107 | 0.000590438 | -3.05159829 | 1.005180239 | 0.996691817 |
| aga-miR-92a | 2.912301598 | 5.736061723 | 5.230136112 | 0.020433414 | 0.977900779 | 0.094334775 | -0.133212056 | 0.85299622 | 0.844688724 |
| age-miR-101 | 111.8323814 | 185.6189573 | 153.1267628 | 0 | 0.731006076 | 0.000149902 | -0.277617608 | 0.461723275 | 0.453388468 |
| age-miR-103 | 311.1604325 | 374.4102062 | 316.5621998 | 0 | 0.266961145 | 0 | -0.242130767 | 0.033165989 | 0.024830378 |
| age-miR-106a | 0.53392196 | 0.210322263 | 0.532699048 | 0.158964182 | -1.344027316 | 0.200950336 | 1.340719133 | 0.005180239 | -0.003308183 |
| age-miR-106b | 35.69649673 | 33.65156211 | 32.87514128 | 0.03580795 | -0.085109018 | 0.049805356 | -0.033676382 | -0.110450534 | -0.1187854 |
| age-miR-127 | 715.5525027 | 1239.448217 | 1123.898138 | 0 | 0.792568467 | 0 | -0.141186712 | 0.659717064 | 0.651381754 |
| age-miR-128 | 48.05297637 | 41.98797181 | 30.79969044 | 0.018489718 | -0.194649692 | 0.003321037 | -0.447060251 | -0.633375387 | -0.641709942 |
| age-miR-15a | 6.115833356 | 7.801043943 | 8.426330403 | 0.051742827 | 0.351118103 | 0.082310071 | 0.111237287 | 0.470696787 | 0.46235539 |
| age-miR-15b | 32.90900806 | 38.77577725 | 32.83363226 | 0.015748197 | 0.236673158 | 0.023590355 | -0.239981341 | 0.005025258 | -0.003308183 |
| age-miR-16 | 128.4325005 | 159.003631 | 168.5266081 | 4.67E-05 | 0.308049381 | 0.018984842 | 0.083916681 | 0.40030155 | 0.391966062 |
| age-miR-17-3p | 0.961059527 | 1.514320295 | 1.598097145 | 0.115981432 | 0.655972684 | 0.153188595 | 0.077684727 | 0.742096585 | 0.733657411 |
| age-miR-17-5p | 172.456793 | 126.1933579 | 112.9321983 | 0 | -0.45059898 | 0.008576485 | -0.160179103 | -0.602442855 | -0.610778083 |
| age-miR-18 | 13.68781751 | 6.883274067 | 6.101825465 | 0.001346258 | -0.991725572 | 0.086620553 | -0.17385404 | -1.157246879 | -1.165579612 |
| age-miR-197 | 53.00388909 | 53.91898019 | 56.36924477 | 0.030901743 | 0.024694991 | 0.039214902 | 0.064115028 | 0.097146425 | 0.088810019 |
| age-miR-19a | 30.64247768 | 10.31493534 | 13.34063704 | 0 | -1.570798177 | 0.041188114 | 0.371092782 | -1.191375436 | -1.199705396 |
| age-miR-19b | 73.54194644 | 39.06507427 | 42.8012105 | 0 | -0.912688004 | 0.036220062 | 0.131772242 | -0.772582172 | -0.780915761 |
| age-miR-214 | 295.9453148 | 457.901613 | 422.8108448 | 0 | 0.62970703 | 0.002115646 | -0.115025266 | 0.523017262 | 0.514681764 |
| age-miR-218 | 108.9200798 | 99.75284482 | 148.5469347 | 0.014159755 | -0.126840053 | 0 | 0.574488944 | 0.455983838 | 0.447648891 |
| age-miR-22 | 128.4325005 | 286.1147587 | 234.4844357 | 0 | 1.155583589 | 0 | -0.287101754 | 0.876817871 | 0.868481835 |
| age-miR-222 | 59.5322985 | 37.64768511 | 47.41021532 | 3.82E-06 | -0.661111438 | 0.006958934 | 0.332636786 | -0.320139053 | -0.328474652 |
| age-miR-27a | 274.6300407 | 561.216279 | 576.1866617 | 0 | 1.031067412 | 0.013419919 | 0.037979406 | 1.077382214 | 1.069046819 |
| age-miR-28 | 28.83178582 | 47.49459106 | 48.23347748 | 1.09E-05 | 0.720103022 | 0.044068038 | 0.022271607 | 0.750708064 | 0.742374628 |
| age-miR-29a | 50.96527797 | 58.04894463 | 49.39572995 | 0.013536059 | 0.187755147 | 0.015483172 | -0.232883506 | -0.036791583 | -0.045128359 |
| age-miR-29b | 4.484944461 | 2.019093726 | 2.876574862 | 0.027160268 | -1.151382238 | 0.111356788 | 0.510644134 | -0.632402367 | -0.640738103 |
| age-miR-30b | 97.93654231 | 194.9386919 | 201.2080406 | 0 | 0.993101302 | 0.022992933 | 0.04566749 | 1.047104706 | 1.038768792 |
| age-miR-34a | 19.80365087 | 38.77577725 | 23.82617562 | 0 | 0.969389279 | 0.000151976 | -0.702608299 | 0.275118645 | 0.266780981 |
| age-miR-93 | 163.96258 | 130.0938799 | 123.1987618 | 3.16E-05 | -0.333813503 | 0.022781293 | -0.078565338 | -0.404044019 | -0.41237884 |
| age-miR-98 | 283.0757153 | 411.8492317 | 292.3064961 | 0 | 0.54092831 | 0 | -0.494634406 | 0.054629272 | 0.046293903 |
| aja-let-7i | 1.456150799 | 4.818291847 | 4.067883643 | 0.005402066 | 1.726362012 | 0.096088965 | -0.244243368 | 1.490437866 | 1.482118644 |
| aja-miR-143 | 3813.950173 | 6768.093948 | 6319.457239 | 0 | 0.827463599 | 0 | -0.09894894 | 0.736849922 | 0.728514659 |
| aja-miR-145 | 37.27746046 | 45.20016638 | 46.19953566 | 0.009177948 | 0.278024504 | 0.0436536 | 0.031550269 | 0.317910849 | 0.309574772 |
| aja-miR-21 | 2.621071438 | 1.606097282 | 2.033941822 | 0.076961348 | -0.706597395 | 0.131350463 | 0.340719133 | -0.357550289 | -0.365878262 |
| aja-miR-25 | 168.0398022 | 211.7753988 | 173.7567442 | 0 | 0.333732013 | 4.59E-05 | -0.285466031 | 0.056600824 | 0.048265983 |
| aja-miR-29b | 3.899951705 | 1.755733675 | 2.501369445 | 0.027160268 | -1.151382238 | 0.111356788 | 0.510644134 | -0.632402367 | -0.640738103 |
| aja-miR-29c | 0.29123016 | 0.458884938 | 0.290563117 | 0.195628342 | 0.655972684 | 0.247312928 | -0.659280867 | 0.004933982 | -0.003308183 |
| aja-miR-3120 | 0.610196525 | 0 | 0 | 0.10800582 | 0.71265413 | -- | 0 | -22.47704278 | 0.71265413 |
| aja-miR-331 | 3.050982627 | 4.086261113 | 4.261592388 | 0.074081236 | 0.421507431 | 0.108830484 | 0.060611214 | 0.490437866 | 0.482118644 |
| aja-miR-671 | 5.186670465 | 5.528470918 | 4.8703913 | 0.076548935 | 0.092071799 | 0.095622882 | -0.182842823 | -0.08246216 | -0.090771024 |
| ame-let-7 | 262.0794076 | 739.1325248 | 631.3244723 | 0 | 1.495829058 | 0 | -0.227451384 | 1.276713278 | 1.268377675 |
| api-miR-7 | 1149.80094 | 508.1385878 | 660.2804706 | 0 | -1.178090184 | 0 | 0.377856949 | -0.791898056 | -0.800233235 |
| asu-miR-1-3p | 11.59373398 | 12.73951994 | 20.39476357 | 0.052885029 | 0.135965626 | 0.00497526 | 0.678887869 | 0.823186699 | 0.814853494 |
| bfl-miR-133 | 4.077222237 | 4.12996444 | 5.52069923 | 0.084677256 | 0.018542764 | 0.077461386 | 0.418721645 | 0.445591505 | 0.437264409 |
| bfl-miR-183 | 0.873690479 | 0.458884938 | 2.905631174 | 0.13744665 | -0.928989816 | 0.009545471 | 2.662647228 | 1.742047336 | 1.733657411 |
| bfl-miR-210-3p | 2.621071438 | 4.12996444 | 4.939572995 | 0.057073156 | 0.655972684 | 0.094360351 | 0.258256973 | 0.92259074 | 0.914229657 |
| bfl-miR-9-5p | 1.456150799 | 2.982752096 | 2.033941822 | 0.051613846 | 1.034484308 | 0.101711574 | -0.552365663 | 0.490402688 | 0.482118644 |
| bfl-miR-96-5p | 0.835703937 | 0.877866838 | 1.111719753 | 0.147179596 | 0.071010184 | 0.167109089 | 0.340719133 | 0.420094615 | 0.411729316 |
| bma-miR-92 | 0 | 0.252386716 | 0 | 0.25085307 | -1.986292118 | 0.315638314 | 1.986292118 | -- | 0 |
| bta-let-7a-3p | 39.96787241 | 34.13229871 | 39.87632877 | 0.019362468 | -0.227703198 | 0.027004051 | 0.224395015 | 0.005027716 | -0.003308183 |
| bta-let-7d | 97.8533337 | 125.734473 | 103.1499067 | 3.45E-05 | 0.361687345 | 0.000846 | -0.285637736 | 0.084384632 | 0.076049609 |
| bta-let-7e | 204.7209342 | 346.1303531 | 324.4898204 | 0 | 0.757656825 | 0.007076825 | -0.093142241 | 0.672850074 | 0.664514584 |
| bta-miR-101 | 116.2424381 | 193.0157455 | 159.2009156 | 0 | 0.731581674 | 0.000151976 | -0.277869909 | 0.46204762 | 0.453711766 |
| bta-miR-105a | 0 | 0 | 0.277929938 | -- | 0 | 0.26087974 | -1.847206846 | 21.48197676 | -1.847206846 |
| bta-miR-106a | 0.58246032 | 0.229442469 | 0.581126235 | 0.158964182 | -1.344027316 | 0.200950336 | 1.340719133 | 0.005180239 | -0.003308183 |
| bta-miR-10b | 493.9010267 | 525.622769 | 592.2686986 | 0.001217459 | 0.089805804 | 1.86E-05 | 0.17222407 | 0.270365178 | 0.262029874 |
| bta-miR-1185 | 0 | 0.480736602 | 0.304399456 | 0.152468748 | -1.056681446 | 0.247312928 | -0.659280867 | 21.48197676 | -1.715962313 |
| bta-miR-1197 | 12.81412703 | 23.31572517 | 18.26396738 | 0.000314971 | 0.863568104 | 0.026262685 | -0.352303114 | 0.519600727 | 0.51126499 |
| bta-miR-1246 | 10.11641608 | 11.68948999 | 25.23311282 | 0.053486524 | 0.208513707 | 4.59E-05 | 1.110106205 | 1.326950448 | 1.318619912 |
| bta-miR-125a | 207.8117123 | 307.4728599 | 237.3521673 | 0 | 0.565182105 | 0 | -0.373429848 | 0.200088049 | 0.191752257 |
| bta-miR-1260b | 11.39033514 | 21.87351537 | 34.4478718 | 0.000510414 | 0.941374903 | 0.001541776 | 0.655229756 | 1.604937162 | 1.596604659 |
| bta-miR-126-3p | 2425.836286 | 3089.694138 | 3206.848272 | 0 | 0.348981837 | 0.000346091 | 0.053692069 | 0.411009162 | 0.402673906 |
| bta-miR-1271 | 91.15504002 | 88.79423547 | 115.0629945 | 0.024560966 | -0.037856406 | 0.000127698 | 0.373885997 | 0.344365449 | 0.33602959 |
| bta-miR-129 | 2.038611119 | 2.06498222 | 11.04139846 | 0.108581841 | 0.018542764 | 0 | 2.418721645 | 2.445604465 | 2.437264409 |
| bta-miR-129-3p | 0.29123016 | 0 | 2.033941822 | 0.207164791 | 1.779768326 | 0.005688728 | 1.024278413 | 2.812429628 | 2.804046739 |
| bta-miR-1296 | 2.912301598 | 2.523867158 | 2.615068056 | 0.095584316 | -0.206523792 | 0.126621282 | 0.051212516 | -0.14700378 | -0.155311276 |
| bta-miR-1298 | 21.55103183 | 100.4958014 | 294.9215641 | 0 | 2.221306378 | 0 | 1.553196085 | 3.782838573 | 3.774502464 |
| bta-miR-1306 | 2.745884364 | 3.365156211 | 3.348394019 | 0.087409043 | 0.293402605 | 0.118658179 | -0.007204171 | 0.294564785 | 0.286198434 |
| bta-miR-1307 | 71.35138915 | 53.68953772 | 60.72769153 | 0.000473487 | -0.410300535 | 0.022194503 | 0.177713545 | -0.224252146 | -0.23258699 |
| bta-miR-130b | 1.747380959 | 3.212194565 | 3.486757408 | 0.056636429 | 0.878365106 | 0.113959284 | 0.118326711 | 1.005015787 | 0.996691817 |
| bta-miR-132 | 1.747380959 | 2.294424689 | 2.324504939 | 0.098271658 | 0.392938279 | 0.129388308 | 0.018791038 | 0.420073807 | 0.411729316 |
| bta-miR-1343-3p | 2.242472231 | 2.776253874 | 1.917716575 | 0.098117594 | 0.308049381 | 0.11343125 | -0.533749985 | -0.217364868 | -0.225700604 |
| bta-miR-135b | 1.392839895 | 1.097333547 | 0.555859877 | 0.120876304 | -0.344027316 | 0.147080773 | -0.981208962 | -1.316846701 | -1.325236278 |
| bta-miR-138 | 0.278567979 | 0.658400128 | 1.945509568 | 0.152183516 | 1.240935185 | 0.063436721 | 1.563111554 | 2.812429628 | 2.804046739 |
| bta-miR-1388-5p | 0.305098263 | 1.442209805 | 0.913198369 | 0.062662052 | 2.240935185 | 0.145893296 | -0.659280867 | 1.590060659 | 1.581654318 |
| bta-miR-139 | 35.93526928 | 52.89147696 | 50.0273889 | 7.53E-05 | 0.557634765 | 0.03761889 | -0.080317107 | 0.485653942 | 0.477317658 |
| bta-miR-140 | 2936.473701 | 2033.089717 | 2442.473565 | 0 | -0.530410837 | 0 | 0.264669067 | -0.257406486 | -0.26574177 |
| bta-miR-1434-3p | 5.796866991 | 2.644051308 | 5.174790757 | 0.01484101 | -1.13252321 | 0.03726487 | 0.968750355 | -0.155434893 | -0.163772855 |
| bta-miR-145 | 374.1167957 | 313.6179277 | 325.4559578 | 0 | -0.254480704 | 0.015660796 | 0.053454292 | -0.192691183 | -0.201026412 |
| bta-miR-1468 | 57.66357164 | 41.52908687 | 60.43712841 | 0.000410196 | -0.473538049 | 0.000136621 | 0.541312964 | 0.076110319 | 0.067774915 |
| bta-miR-146b | 17.88638565 | 41.85413037 | 12.78477716 | 0 | 1.226508115 | 0 | -1.710942987 | -0.476102297 | -0.484434873 |
| bta-miR-150 | 3.899951705 | 11.41226889 | 9.171687965 | 7.83E-05 | 1.549057481 | 0.052874414 | -0.315326466 | 1.242066751 | 1.233731014 |
| bta-miR-151-3p | 1566.37448 | 1861.171753 | 1672.979412 | 0 | 0.248782031 | 0 | -0.153791505 | 0.103325858 | 0.094990525 |
| bta-miR-151-5p | 31.73021932 | 59.37097029 | 49.31271192 | 0 | 0.903900198 | 0.011560706 | -0.267798096 | 0.644435448 | 0.636102102 |
| bta-miR-152 | 646.2397246 | 1301.627126 | 1389.472827 | 0 | 1.010174881 | 0.000123152 | 0.094221399 | 1.112731629 | 1.10439628 |
| bta-miR-154a | 0 | 0.229442469 | 0 | 0.25085307 | -2.123795642 | 0.315638314 | 2.123795642 | -- | 0 |
| bta-miR-154b | 11.69985512 | 18.21573688 | 9.727547842 | 0.004217595 | 0.638694693 | 0.001012658 | -0.905037282 | -0.258002162 | -0.266342589 |
| bta-miR-154c | 165.7099609 | 224.6241771 | 184.2170164 | 0 | 0.438852892 | 2.94E-05 | -0.286106887 | 0.161081272 | 0.152746005 |
| bta-miR-155 | 226.4757669 | 211.1269744 | 260.9762121 | 0.007984843 | -0.101245774 | 0 | 0.305807397 | 0.212896946 | 0.204561623 |
| bta-miR-15a | 6.407063516 | 8.172522226 | 8.827584232 | 0.051742827 | 0.351118103 | 0.082310071 | 0.111237287 | 0.470696787 | 0.46235539 |
| bta-miR-16b | 132.412646 | 164.652286 | 172.8988912 | 4.03E-05 | 0.31438163 | 0.021360305 | 0.070506074 | 0.393223204 | 0.384887704 |
| bta-miR-181a | 143.8919681 | 233.8783566 | 244.7752128 | 0 | 0.700772294 | 0.016094185 | 0.065699111 | 0.774807047 | 0.766471406 |
| bta-miR-181b | 99.04252352 | 74.45408116 | 106.5398097 | 4.03E-05 | -0.411697142 | 0 | 0.516969772 | 0.113607654 | 0.10527263 |
| bta-miR-181c | 0 | 0.420644526 | 0.266349524 | 0.152468748 | -1.249326524 | 0.247312928 | -0.659280867 | 21.48197676 | -1.908607391 |
| bta-miR-181d | 15.59980682 | 25.01920487 | 21.67853519 | 0.000858726 | 0.681507777 | 0.039724959 | -0.206768663 | 0.483081165 | 0.474739114 |
| bta-miR-182 | 3.737453718 | 1.261933579 | 4.794291436 | 0.01211819 | -1.566419737 | 0.003237778 | 1.925681633 | 0.367597633 | 0.359261897 |
| bta-miR-183 | 0.835703937 | 1.097333547 | 3.613089198 | 0.134856116 | 0.392938279 | 0.016344202 | 1.719230756 | 2.120562747 | 2.112169035 |
| bta-miR-1839 | 21.62383937 | 37.64768511 | 33.82638958 | 7.39E-06 | 0.799938459 | 0.034540496 | -0.154411958 | 0.653862208 | 0.645526501 |
| bta-miR-185 | 212.0155563 | 369.4023749 | 220.246843 | 0 | 0.801023017 | 0 | -0.746071802 | 0.063286344 | 0.054951215 |
| bta-miR-186 | 495.673732 | 680.9852477 | 552.9416123 | 0 | 0.458232739 | 0 | -0.300496398 | 0.166071623 | 0.157736342 |
| bta-miR-187 | 1.456150799 | 5.277176785 | 1.452815587 | 0.002673633 | 1.857606546 | 0.004639677 | -1.860914728 | 0.005032146 | -0.003308183 |
| bta-miR-188 | 0.29123016 | 1.835539751 | 1.452815587 | 0.030308445 | 2.655972684 | 0.138855988 | -0.337352772 | 2.327059086 | 2.318619912 |
| bta-miR-190a | 2.329841278 | 2.294424689 | 0.871689352 | 0.104090671 | -0.022099221 | 0.065634915 | -1.396246461 | -1.41000112 | -1.418345682 |
| bta-miR-192 | 56.27073175 | 61.01174521 | 59.75493674 | 0.01999447 | 0.116702274 | 0.039166272 | -0.03002909 | 0.095009179 | 0.086673184 |
| bta-miR-193a-3p | 0.873690479 | 0.458884938 | 0.581126235 | 0.13744665 | -0.928989816 | 0.221716937 | 0.340719133 | -0.579782262 | -0.588270684 |
| bta-miR-193a-5p | 16.89134927 | 24.77978664 | 23.53561251 | 0.003587 | 0.552879191 | 0.0533869 | -0.074318367 | 0.486896555 | 0.478560825 |
| bta-miR-193b | 30.70051268 | 39.33026321 | 32.76099148 | 0.004713525 | 0.357377093 | 0.018173893 | -0.263660822 | 0.102053557 | 0.093716272 |
| bta-miR-195 | 222.4998421 | 301.0285192 | 287.07636 | 0 | 0.436095861 | 0.013335017 | -0.06846564 | 0.375965492 | 0.367630221 |
| bta-miR-199a-3p | 2671.745486 | 6699.031765 | 4986.64422 | 0 | 1.326170011 | 0 | -0.425883317 | 0.908621978 | 0.900286694 |
| bta-miR-199a-5p | 2862.792471 | 4101.513574 | 3447.531387 | 0 | 0.518733313 | 0 | -0.250592717 | 0.276475879 | 0.268140596 |
| bta-miR-199b | 4386.331397 | 5481.839467 | 4780.672869 | 0 | 0.321645265 | 0 | -0.197446391 | 0.132534152 | 0.124198874 |
| bta-miR-199c | 7.688476219 | 32.81027305 | 21.09488232 | 0 | 2.093377997 | 0.001511661 | -0.637254561 | 1.464447405 | 1.456123436 |
| bta-miR-200a | 2.507111811 | 0.877866838 | 6.67031852 | 0.035314737 | -1.513952317 | 1.86E-05 | 2.925681633 | 1.420080743 | 1.411729316 |
| bta-miR-200b | 2.440786101 | 0.721104902 | 6.087989126 | 0.03341018 | -1.759064815 | 7.84E-05 | 3.077684727 | 1.326972682 | 1.318619912 |
| bta-miR-202 | 0 | 0.240368301 | 0 | 0.25085307 | -2.056681446 | 0.315638314 | 2.056681446 | -- | 0 |
| bta-miR-206 | 0 | 0.229442469 | 0.290563117 | 0.25085307 | -2.123795642 | 0.286090674 | 0.340719133 | 21.48197676 | -1.783076509 |
| bta-miR-20a | 644.3277353 | 386.7003419 | 372.7040473 | 0 | -0.736578657 | 0.013048105 | -0.053185554 | -0.781428924 | -0.789764212 |
| bta-miR-20b | 2.912301598 | 2.06498222 | 2.324504939 | 0.083536891 | -0.496030409 | 0.129815067 | 0.170794131 | -0.316908261 | -0.325236278 |
| bta-miR-210 | 2.507111811 | 4.389334188 | 5.836528705 | 0.041464278 | 0.807975778 | 0.072943647 | 0.411108461 | 1.227435665 | 1.219084239 |
| bta-miR-212 | 0.278567979 | 0.219466709 | 0 | 0.227853908 | -0.344027316 | 0.315638314 | 2.187925979 | -21.47704278 | 1.843898663 |
| bta-miR-215 | 1.164920639 | 4.12996444 | 6.392388582 | 0.007623226 | 1.825897686 | 0.049164994 | 0.63022575 | 2.464398759 | 2.456123436 |
| bta-miR-21-5p | 57457.47777 | 41801.97045 | 43912.51336 | 0 | -0.458923714 | 0 | 0.071061161 | -0.379527266 | -0.387862553 |
| bta-miR-222 | 68.03691257 | 43.02592584 | 54.18310322 | 3.82E-06 | -0.661111438 | 0.006958934 | 0.332636786 | -0.320139053 | -0.328474652 |
| bta-miR-223 | 0.873690479 | 1.835539751 | 1.743378704 | 0.081065193 | 1.071010184 | 0.141457698 | -0.074318367 | 1.005098158 | 0.996691817 |
| bta-miR-22-3p | 134.5483338 | 299.739271 | 245.6503612 | 0 | 1.155583589 | 0 | -0.287101754 | 0.876817871 | 0.868481835 |
| bta-miR-224 | 398.9093459 | 292.988057 | 262.3658618 | 0 | -0.445219066 | 0.001167965 | -0.159261844 | -0.596145814 | -0.604480911 |
| bta-miR-22-5p | 15.72642863 | 29.82752096 | 32.54306914 | 1.80E-05 | 0.923452995 | 0.043986271 | 0.125706242 | 1.05749529 | 1.049159237 |
| bta-miR-2284ab | 8.542751354 | 8.412890527 | 9.436383145 | 0.066049918 | -0.022099221 | 0.077387012 | 0.165632426 | 0.151879533 | 0.143533206 |
| bta-miR-2284x | 632.1636002 | 957.8676785 | 968.9034693 | 0 | 0.599528406 | 0.013492497 | 0.016526565 | 0.624390295 | 0.616054971 |
| bta-miR-2284y | 2.440786101 | 4.086261113 | 3.652793475 | 0.053787679 | 0.743435526 | 0.108849262 | -0.161781208 | 0.58999888 | 0.581654318 |
| bta-miR-2285ab | 0.873690479 | 0.688327407 | 0.871689352 | 0.149911551 | -0.344027316 | 0.188804199 | 0.340719133 | 0.005098158 | -0.003308183 |
| bta-miR-2285b | 2.038611119 | 0.688327407 | 2.615068056 | 0.04988742 | -1.566419737 | 0.02941626 | 1.925681633 | 0.367597633 | 0.359261897 |
| bta-miR-2285c | 0.29123016 | 1.147212345 | 1.743378704 | 0.085038376 | 1.977900779 | 0.12955274 | 0.603753539 | 2.590060659 | 2.581654318 |
| bta-miR-2285f | 34.36515886 | 26.15644146 | 27.60349615 | 0.006906708 | -0.393780351 | 0.051045348 | 0.077684727 | -0.307762946 | -0.316095624 |
| bta-miR-2285k | 0.305098263 | 0.240368301 | 0 | 0.227853908 | -0.344027316 | 0.315638314 | 2.056681446 | -21.47704278 | 1.71265413 |
| bta-miR-2285l | 238.732758 | 133.655226 | 212.060543 | 0 | -0.836880292 | 0 | 0.665959962 | -0.162584725 | -0.17092033 |
| bta-miR-2314 | 0.915294788 | 0.480736602 | 0 | 0.13744665 | -0.928989816 | 0.193791884 | 1.056681446 | -23.06200528 | 0.127691629 |
| bta-miR-2331-3p | 0.610196525 | 0.961473203 | 0.304399456 | 0.143516961 | 0.655972684 | 0.143485591 | -1.659280867 | -0.995066018 | -1.003308183 |
| bta-miR-2332 | 3.737453718 | 2.523867158 | 10.12128192 | 0.063945412 | -0.566419737 | 1.31E-05 | 2.003684145 | 1.445604465 | 1.437264409 |
| bta-miR-2355-3p | 3.494761918 | 5.506619254 | 7.264077934 | 0.042769222 | 0.655972684 | 0.065307593 | 0.399612822 | 1.063922609 | 1.055585506 |
| bta-miR-2366 | 0 | 0.229442469 | 0 | 0.25085307 | -2.123795642 | 0.315638314 | 2.123795642 | -- | 0 |
| bta-miR-23a | 532.6599623 | 645.88055 | 887.3797604 | 0 | 0.278052532 | 0 | 0.458284273 | 0.744672222 | 0.736336804 |
| bta-miR-23b-3p | 247.0897973 | 369.8014053 | 397.7177418 | 0 | 0.581715266 | 0.003846072 | 0.104994213 | 0.695044598 | 0.68670948 |
| bta-miR-24 | 4.178519684 | 5.486667735 | 6.948248459 | 0.060690946 | 0.392938279 | 0.071140764 | 0.340719133 | 0.742010988 | 0.733657411 |
| bta-miR-2419-5p | 33.20023822 | 92.2358725 | 65.95782764 | 0 | 1.474134361 | 2.38E-05 | -0.483784071 | 0.998685823 | 0.99035029 |
| bta-miR-2447 | 0.58246032 | 0.917769876 | 0.871689352 | 0.143516961 | 0.655972684 | 0.185895435 | -0.074318367 | 0.590060659 | 0.581654318 |
| bta-miR-2448-3p | 0 | 0.229442469 | 0.290563117 | 0.25085307 | -2.123795642 | 0.286090674 | 0.340719133 | 21.48197676 | -1.783076509 |
| bta-miR-26a | 3342.157314 | 3618.307735 | 4239.606445 | 0 | 0.114535471 | 0 | 0.228615235 | 0.351485972 | 0.343150706 |
| bta-miR-26b | 440.0487715 | 605.9575604 | 861.2290799 | 0 | 0.461553325 | 0 | 0.507180279 | 0.977068692 | 0.968733604 |
| bta-miR-27a-3p | 302.0930448 | 617.3379068 | 633.8053279 | 0 | 1.031067412 | 0.013419919 | 0.037979406 | 1.077382214 | 1.069046819 |
| bta-miR-27a-5p | 54.75127004 | 26.15644146 | 68.5728957 | 0 | -1.065726153 | 0 | 1.390472168 | 0.33308153 | 0.324746015 |
| bta-miR-27b | 7883.739107 | 12865.7133 | 12681.28135 | 0 | 0.706579505 | 0.000331856 | -0.020830919 | 0.694083872 | 0.685748587 |
| bta-miR-296-3p | 26.50194454 | 22.25591948 | 27.31293303 | 0.025074302 | -0.251909114 | 0.026887634 | 0.295395142 | 0.051819827 | 0.043486029 |
| bta-miR-299 | 2.038611119 | 5.047734316 | 3.196194291 | 0.012723618 | 1.308049381 | 0.061096399 | -0.659280867 | 0.657119175 | 0.648768514 |
| bta-miR-29a | 48.74939632 | 55.52507748 | 47.24808952 | 0.013536059 | 0.187755147 | 0.015483172 | -0.232883506 | -0.036791583 | -0.045128359 |
| bta-miR-29d-5p | 2.745884364 | 2.884419609 | 2.739595107 | 0.098568343 | 0.071010184 | 0.125959855 | -0.074318367 | 0.005043243 | -0.003308183 |
| bta-miR-301a | 0.512565081 | 0.605728118 | 0 | 0.163565675 | 0.240935185 | 0.12613315 | 0.723257712 | -22.47704278 | 0.964192897 |
| bta-miR-30a-5p | 183.4021931 | 419.3825928 | 365.6978968 | 0 | 1.19325609 | 1.86E-05 | -0.197614652 | 1.003977029 | 0.995641439 |
| bta-miR-30c | 114.4914393 | 115.0005557 | 96.99754848 | 0.021768352 | 0.006401102 | 0.002440523 | -0.245620643 | -0.230883992 | -0.23921954 |
| bta-miR-30d | 643.6429224 | 598.9978055 | 654.9534801 | 0.000888661 | -0.10370982 | 0.000193324 | 0.128841721 | 0.033467303 | 0.025131901 |
| bta-miR-30e-5p | 272.5671604 | 220.8383763 | 209.8834251 | 0 | -0.303620854 | 0.015559043 | -0.07340266 | -0.368688386 | -0.377023514 |
| bta-miR-30f | 18.68726859 | 32.59995079 | 29.29844767 | 2.49E-05 | 0.802814073 | 0.038430546 | -0.154045559 | 0.657098683 | 0.648768514 |
| bta-miR-3154 | 0.305098263 | 0 | 0 | 0.207164791 | 1.71265413 | -- | 0 | -21.47704278 | 1.71265413 |
| bta-miR-320a | 152.6046037 | 131.4705347 | 99.66314925 | 0.001748044 | -0.215058988 | 1.86E-05 | -0.39960743 | -0.60633076 | -0.614666418 |
| bta-miR-323 | 16.30888895 | 28.45086614 | 23.53561251 | 0.000133263 | 0.802814073 | 0.028126212 | -0.273627175 | 0.537528715 | 0.529186898 |
| bta-miR-324 | 1.114271916 | 2.194667094 | 2.501369445 | 0.070826985 | 0.977900779 | 0.126579328 | 0.188716039 | 1.174899604 | 1.166616819 |
| bta-miR-326 | 0 | 2.271480442 | 1.598097145 | 0.005399256 | 1.183632884 | 0.126492745 | -0.507277774 | 23.80410187 | 0.67635511 |
| bta-miR-328 | 26.7931747 | 49.78901575 | 34.57701097 | 0 | 0.893961961 | 0.000557557 | -0.526014336 | 0.376280136 | 0.367947624 |
| bta-miR-335 | 34.26386141 | 41.25974137 | 40.577771 | 0.01081698 | 0.268047031 | 0.044606547 | -0.02404516 | 0.252336365 | 0.244001871 |
| bta-miR-338 | 0.278567979 | 1.097333547 | 0.555859877 | 0.085038376 | 1.977900779 | 0.147080773 | -0.981208962 | 1.005180239 | 0.996691817 |
| bta-miR-339a | 94.35857178 | 167.9518872 | 145.2815587 | 0 | 0.83182252 | 0.002015585 | -0.209196421 | 0.630960979 | 0.622626099 |
| bta-miR-339b | 4.484944461 | 18.17184354 | 14.70249374 | 0 | 2.018542764 | 0.042369534 | -0.305643913 | 1.721241725 | 1.712898851 |
| bta-miR-342 | 271.9157756 | 169.4019636 | 142.1667221 | 0 | -0.682709256 | 0.000293028 | -0.252866795 | -0.927240993 | -0.935576051 |
| bta-miR-3431 | 313.363652 | 275.3309627 | 289.9819911 | 0.00033476 | -0.186670988 | 0.01342233 | 0.074796448 | -0.10353899 | -0.11187454 |
| bta-miR-345-3p | 1.922119055 | 3.281027305 | 1.917716575 | 0.066648537 | 0.771449902 | 0.086520135 | -0.774758085 | 0.005015787 | -0.003308183 |
| bta-miR-345-5p | 1.525491313 | 1.922946406 | 1.217597825 | 0.111252526 | 0.33404459 | 0.125444122 | -0.659280867 | -0.316969824 | -0.325236278 |
| bta-miR-361 | 13.68781751 | 22.71480442 | 26.15068056 | 0.000991945 | 0.730740453 | 0.040570473 | 0.203215609 | 0.942294266 | 0.933956062 |
| bta-miR-362-3p | 0.610196525 | 2.884419609 | 1.826396738 | 0.014657971 | 2.240935185 | 0.099764444 | -0.659280867 | 1.590060659 | 1.581654318 |
| bta-miR-362-5p | 29.36570778 | 27.76253874 | 30.09749624 | 0.037121992 | -0.08099291 | 0.043679536 | 0.116503976 | 0.043842479 | 0.035511066 |
| bta-miR-363 | 0 | 0.721104902 | 1.826396738 | 0.09860269 | -0.471718945 | 0.08996163 | 1.340719133 | 24.06710344 | 0.869000188 |
| bta-miR-365-5p | 24.79255013 | 8.998135085 | 15.28614661 | 0 | -1.462208742 | 0.005264355 | 0.764526842 | -0.689341781 | -0.6976819 |
| bta-miR-369-3p | 126.0055825 | 227.3884125 | 169.5504971 | 0 | 0.851671086 | 0 | -0.423443723 | 0.436562457 | 0.428227363 |
| bta-miR-370 | 41.93714301 | 49.78901575 | 35.73926344 | 0.010204528 | 0.247598915 | 0.001171071 | -0.478317594 | -0.222383841 | -0.230718679 |
| bta-miR-374a | 112.4148417 | 157.3975337 | 155.4512678 | 0 | 0.485580413 | 0.027499043 | -0.017950552 | 0.475965315 | 0.467629861 |
| bta-miR-374b | 68.73031772 | 123.6694907 | 104.3121591 | 0 | 0.847471098 | 0.002290207 | -0.245582296 | 0.610222953 | 0.601888802 |
| bta-miR-376a | 0.915294788 | 2.163314707 | 2.43519565 | 0.065479877 | 1.240935185 | 0.129815067 | 0.170794131 | 1.420156178 | 1.411729316 |
| bta-miR-376b | 6.698293676 | 6.424389129 | 4.649009878 | 0.070865668 | -0.06023435 | 0.065293441 | -0.466635789 | -0.518538523 | -0.526870139 |
| bta-miR-376c | 4.659682557 | 3.671079503 | 4.649009878 | 0.071662188 | -0.344027316 | 0.091616251 | 0.340719133 | 0.005026119 | -0.003308183 |
| bta-miR-376d | 31.12002279 | 24.27719838 | 17.04636955 | 0.012086477 | -0.358241175 | 0.010411634 | -0.510137428 | -0.860042011 | -0.868378603 |
| bta-miR-376e | 139.4992466 | 113.8034646 | 104.3121591 | 0.000320313 | -0.293712851 | 0.017239581 | -0.125637144 | -0.411014742 | -0.419349995 |
| bta-miR-377 | 0.873690479 | 0.229442469 | 0.290563117 | 0.107210574 | -1.928989816 | 0.286090674 | 0.340719133 | -1.580028518 | -1.588270684 |
| bta-miR-378 | 736.229844 | 506.6089714 | 543.0624663 | 0 | -0.539283607 | 0.002448381 | 0.10024553 | -0.430702797 | -0.439038077 |
| bta-miR-379 | 458.2575905 | 868.9314073 | 679.4195864 | 0 | 0.923083519 | 0 | -0.354939487 | 0.576479317 | 0.568144031 |
| bta-miR-380-3p | 63.15534037 | 146.6246635 | 94.36383145 | 0 | 1.215151159 | 0 | -0.635821894 | 0.587664239 | 0.579329265 |
| bta-miR-381 | 122.8991274 | 113.3445796 | 112.1573633 | 0.013343071 | -0.116759273 | 0.031675006 | -0.015191062 | -0.12361597 | -0.131950334 |
| bta-miR-382 | 70.47769867 | 142.7132157 | 82.51992533 | 0 | 1.017880217 | 0 | -0.790304518 | 0.235912565 | 0.227575699 |
| bta-miR-409a | 59.33497952 | 76.81334829 | 56.69770742 | 0.000394132 | 0.372474176 | 0.000204992 | -0.438066637 | -0.057257956 | -0.065592461 |
| bta-miR-410 | 13.42432356 | 16.58541275 | 12.48037771 | 0.032065958 | 0.305065523 | 0.03168584 | -0.410253319 | -0.096856079 | -0.105187797 |
| bta-miR-411a | 415.2942079 | 821.6334812 | 578.8017298 | 0 | 0.984361221 | 0 | -0.505425739 | 0.487270769 | 0.478935483 |
| bta-miR-411b | 39.89853189 | 49.55957328 | 35.1581372 | 0.005361604 | 0.312828104 | 0.000936846 | -0.495305132 | -0.174143732 | -0.182477029 |
| bta-miR-411c-3p | 39.02484141 | 91.08866016 | 58.98431282 | 0 | 1.222878691 | 0 | -0.626940147 | 0.604274354 | 0.595938544 |
| bta-miR-411c-5p | 369.5710728 | 442.5945225 | 416.6675103 | 0 | 0.260133759 | 0.005108045 | -0.087088987 | 0.18138016 | 0.173044772 |
| bta-miR-421 | 4.457087663 | 5.267201025 | 4.446879013 | 0.071149491 | 0.240935185 | 0.090839006 | -0.244243368 | 0.005026119 | -0.003308183 |
| bta-miR-423-3p | 374.0123327 | 323.2653185 | 282.0641462 | 2.14E-05 | -0.210367106 | 0.000118548 | -0.196695443 | -0.398727387 | -0.407062549 |
| bta-miR-423-5p | 241.2398698 | 222.9781767 | 199.8316257 | 0.005773516 | -0.113565843 | 0.002807397 | -0.158117594 | -0.263347634 | -0.271683437 |
| bta-miR-424-3p | 61.62984906 | 68.50496572 | 46.57311681 | 0.015270558 | 0.152579311 | 5.65E-05 | -0.556711134 | -0.395795663 | -0.404131823 |
| bta-miR-424-5p | 129.6667616 | 277.8657557 | 145.5029401 | 0 | 1.099579336 | 0 | -0.933339742 | 0.174575288 | 0.166239594 |
| bta-miR-425-3p | 1.456150799 | 1.835539751 | 1.162252469 | 0.111252526 | 0.33404459 | 0.125444122 | -0.659280867 | -0.316969824 | -0.325236278 |
| bta-miR-425-5p | 58.24603196 | 40.611317 | 42.42221513 | 0.000160015 | -0.520277955 | 0.044153203 | 0.062938142 | -0.44900311 | -0.457339814 |
| bta-miR-431 | 19.49975853 | 50.69680987 | 34.18538242 | 0 | 1.378438709 | 0.000184491 | -0.568515403 | 0.818255986 | 0.809923306 |
| bta-miR-432 | 15.04267086 | 26.11653842 | 19.45509568 | 0.000190212 | 0.795902946 | 0.01311018 | -0.424815614 | 0.379425144 | 0.371087332 |
| bta-miR-433 | 23.00718263 | 40.38187453 | 26.7318068 | 7.39E-06 | 0.811623555 | 0.00058467 | -0.59515053 | 0.224806078 | 0.216473025 |
| bta-miR-449a | 15.72642863 | 16.9787427 | 15.98097145 | 0.046930944 | 0.110538548 | 0.062126769 | -0.087374519 | 0.03150312 | 0.023164029 |
| bta-miR-449b | 0.29123016 | 0.229442469 | 0.581126235 | 0.227853908 | -0.344027316 | 0.200950336 | 1.340719133 | 1.005180239 | 0.996691817 |
| bta-miR-451 | 935.1527053 | 4879.183883 | 2372.132024 | 0 | 2.383365982 | 0 | -1.040455549 | 1.351245684 | 1.342910433 |
| bta-miR-452 | 251.0403978 | 179.1945682 | 165.3304138 | 0 | -0.486392636 | 0.011693966 | -0.116174763 | -0.594231844 | -0.6025674 |
| bta-miR-483 | 0.915294788 | 3.605524511 | 1.521997281 | 0.010623533 | 1.977900779 | 0.043883813 | -1.244243368 | 0.742096585 | 0.733657411 |
| bta-miR-485 | 2.228543832 | 3.072533931 | 1.945509568 | 0.080548559 | 0.463327607 | 0.090000021 | -0.659280867 | -0.187632151 | -0.195953261 |
| bta-miR-486 | 27.66686518 | 186.7661697 | 74.38415804 | 0 | 2.75500206 | 0 | -1.328165852 | 1.435172922 | 1.426836209 |
| bta-miR-487b | 8.154444475 | 15.60208789 | 11.04139846 | 0.001044151 | 0.936080604 | 0.022936857 | -0.498816195 | 0.445604465 | 0.437264409 |
| bta-miR-490 | 0 | 0.688327407 | 0 | 0.09860269 | -0.538833141 | 0.12613315 | 0.538833141 | -- | 0 |
| bta-miR-491 | 1.164920639 | 3.441637034 | 1.743378704 | 0.019979853 | 1.56286328 | 0.06061857 | -0.981208962 | 0.589937103 | 0.581654318 |
| bta-miR-493 | 317.149644 | 380.4156135 | 247.8503391 | 0 | 0.262412737 | 0 | -0.618107227 | -0.347359307 | -0.35569449 |
| bta-miR-494 | 538.7757957 | 444.8889472 | 452.9879 | 0 | -0.276239783 | 0.017197182 | 0.026027257 | -0.241877455 | -0.250212526 |
| bta-miR-495 | 102.8042464 | 176.6707011 | 128.1383348 | 0 | 0.781162947 | 0 | -0.463360657 | 0.326137776 | 0.317802289 |
| bta-miR-496 | 0.29123016 | 0.458884938 | 0.290563117 | 0.195628342 | 0.655972684 | 0.247312928 | -0.659280867 | 0.004933982 | -0.003308183 |
| bta-miR-497 | 46.59682557 | 72.73326264 | 57.24093412 | 0 | 0.64238362 | 0.002116266 | -0.345568078 | 0.305151082 | 0.296815542 |
| bta-miR-499 | 2.440786101 | 2.884419609 | 1.826396738 | 0.096057344 | 0.240935185 | 0.099764444 | -0.659280867 | -0.41000112 | -0.418345682 |
| bta-miR-500 | 27.29966194 | 17.55733675 | 17.78751605 | 0.001078347 | -0.636809065 | 0.062206079 | 0.018791038 | -0.609682203 | -0.618018027 |
| bta-miR-503-3p | 6.407063516 | 9.656535213 | 4.446879013 | 0.021846081 | 0.591842347 | 0.0043005 | -1.118712486 | -0.518538523 | -0.526870139 |
| bta-miR-503-5p | 105.0758417 | 127.2029048 | 75.7498047 | 0.000662944 | 0.275700603 | 0 | -0.747817542 | -0.463780275 | -0.472116939 |
| bta-miR-504 | 0.29123016 | 3.671079503 | 0.581126235 | 0.00080425 | 3.655972684 | 0.004860417 | -2.659280867 | 1.005180239 | 0.996691817 |
| bta-miR-505 | 11.35797623 | 8.259928881 | 13.07534028 | 0.03045629 | -0.459504533 | 0.015942144 | 0.662647228 | 0.211473 | 0.203142695 |
| bta-miR-532 | 250.7491676 | 324.890536 | 358.8454499 | 0 | 0.373708807 | 0.001490825 | 0.143408909 | 0.525452758 | 0.517117716 |
| bta-miR-539 | 1.747380959 | 0.458884938 | 1.743378704 | 0.047739679 | -1.928989816 | 0.060821177 | 1.925681633 | 0.005015787 | -0.003308183 |
| bta-miR-542-5p | 1.456150799 | 2.982752096 | 2.615068056 | 0.051613846 | 1.034484308 | 0.121146579 | -0.189795584 | 0.85299622 | 0.844688724 |
| bta-miR-543 | 60.8671034 | 89.02367794 | 60.14656529 | 0 | 0.548526395 | 0 | -0.565706752 | -0.008844944 | -0.017180357 |
| bta-miR-544a | 0 | 0.229442469 | 0 | 0.25085307 | -2.123795642 | 0.315638314 | 2.123795642 | -- | 0 |
| bta-miR-582 | 0.557135958 | 1.536266966 | 0.277929938 | 0.073694749 | 1.463327607 | 0.057483806 | -2.466635789 | -0.995066018 | -1.003308183 |
| bta-miR-6123 | 5.186670465 | 4.326629414 | 6.087989126 | 0.073984812 | -0.261565155 | 0.066934919 | 0.492722226 | 0.239484403 | 0.231157071 |
| bta-miR-628 | 0.29123016 | 1.376654813 | 0.871689352 | 0.062662052 | 2.240935185 | 0.145893296 | -0.659280867 | 1.590060659 | 1.581654318 |
| bta-miR-652 | 5.491768728 | 7.932153925 | 13.69797553 | 0.038490582 | 0.530441802 | 0.008589925 | 0.78817811 | 1.326943882 | 1.318619912 |
| bta-miR-6529a | 17.39060097 | 27.64235459 | 24.04755705 | 0.000932557 | 0.668572721 | 0.040277387 | -0.20099017 | 0.475915012 | 0.467582551 |
| bta-miR-654 | 50.67404781 | 76.17489968 | 75.54641051 | 3.82E-06 | 0.58806862 | 0.037323889 | -0.011952486 | 0.584451151 | 0.576116134 |
| bta-miR-655 | 53.29511925 | 64.24389129 | 58.69374971 | 0.004816976 | 0.269555863 | 0.027460544 | -0.130352401 | 0.147538246 | 0.139203462 |
| bta-miR-656 | 25.62825406 | 26.68088138 | 26.4827527 | 0.04156198 | 0.058071128 | 0.055152492 | -0.010753238 | 0.055653404 | 0.04731789 |
| bta-miR-660 | 330.2550012 | 390.7405246 | 389.0640141 | 0 | 0.242630479 | 0.019883063 | -0.006203342 | 0.244762507 | 0.236427138 |
| bta-miR-665 | 11.21236115 | 19.68616383 | 23.01259889 | 0.001409514 | 0.812091886 | 0.043823804 | 0.225241915 | 1.045669538 | 1.037333802 |
| bta-miR-671 | 17.54978267 | 17.55733675 | 10.00547778 | 0.048717586 | 0.000620856 | 0.00245766 | -0.811283961 | -0.802327368 | -0.810663105 |
| bta-miR-708 | 47.07798844 | 45.64907555 | 55.30805773 | 0.032182244 | -0.044467034 | 0.010295439 | 0.276904035 | 0.240774356 | 0.232437001 |
| bta-miR-744 | 16.01765879 | 16.51985776 | 13.07534028 | 0.05014726 | 0.044537972 | 0.03885046 | -0.337352772 | -0.284480347 | -0.2928148 |
| bta-miR-758 | 11.06674607 | 19.50260986 | 17.14322392 | 0.000930288 | 0.817436107 | 0.049980178 | -0.186028754 | 0.63973844 | 0.631407353 |
| bta-miR-760-3p | 1.601765879 | 1.514320295 | 0.639238858 | 0.12274424 | -0.08099291 | 0.121908424 | -1.244243368 | -1.316846701 | -1.325236278 |
| bta-miR-767 | 0 | 0.219466709 | 0 | 0.25085307 | -2.187925979 | 0.315638314 | 2.187925979 | -- | 0 |
| bta-miR-769 | 20.67734135 | 18.81428245 | 24.98842809 | 0.042159674 | -0.13622243 | 0.016573909 | 0.409431883 | 0.281541131 | 0.273209452 |
| bta-miR-7857 | 9.901825434 | 9.177698756 | 11.91308781 | 0.060757179 | -0.109562062 | 0.045489468 | 0.376343042 | 0.275118645 | 0.266780981 |
| bta-miR-7859 | 6.698293676 | 7.342159005 | 9.007456638 | 0.066411081 | 0.132410728 | 0.065776771 | 0.294915443 | 0.435678639 | 0.427326172 |
| bta-miR-874 | 4.077222237 | 6.194946661 | 5.52069923 | 0.041573862 | 0.603505265 | 0.090237063 | -0.166240856 | 0.445591505 | 0.437264409 |
| bta-miR-885 | 0 | 1.147212345 | 0 | 0.041163135 | 0.198132453 | 0.053049451 | -0.198132453 | -- | 0 |
| bta-miR-92b | 9.028134954 | 6.883274067 | 4.939572995 | 0.044320034 | -0.39133303 | 0.060742464 | -0.478708622 | -0.861705369 | -0.870041652 |
| bta-miR-93 | 163.96258 | 130.0938799 | 123.1987618 | 3.16E-05 | -0.333813503 | 0.022781293 | -0.078565338 | -0.404044019 | -0.41237884 |
| bta-miR-95 | 0.873690479 | 6.424389129 | 7.845204169 | 4.67E-05 | 2.878365106 | 0.072754939 | 0.288251713 | 3.17502316 | 3.166616819 |
| bta-miR-99a-5p | 4030.653148 | 11897.50978 | 6842.595378 | 0 | 1.561574096 | 0 | -0.798044096 | 0.77186529 | 0.76353 |
| bta-miR-99b | 282.2020249 | 334.0682347 | 333.5664587 | 3.82E-06 | 0.243414469 | 0.02102366 | -0.002168581 | 0.249581338 | 0.241245888 |
| ccr-let-7i | 20035.80291 | 27499.09508 | 22528.90816 | 0 | 0.456803819 | 0 | -0.287606748 | 0.177532365 | 0.169197071 |
| ccr-miR-100 | 1242.360126 | 2591.170282 | 2632.446498 | 0 | 1.060520396 | 0.00611217 | 0.022800383 | 1.091656097 | 1.083320779 |
| ccr-miR-126-3p | 917.6662336 | 1275.0118 | 1365.356088 | 0 | 0.47446917 | 8.31E-05 | 0.09876666 | 0.581571092 | 0.573235831 |
| ccr-miR-133a-5p | 0 | 0.240368301 | 0.913198369 | 0.25085307 | -2.056681446 | 0.132213553 | 1.925681633 | 23.06710344 | -0.130999812 |
| ccr-miR-140-5p | 342.4866679 | 194.7966561 | 228.9637365 | 0 | -0.814078917 | 0.00028678 | 0.233150209 | -0.572593421 | -0.580928708 |
| ccr-miR-143 | 4194.704484 | 7444.650956 | 6951.083344 | 0 | 0.827635033 | 0 | -0.098966366 | 0.737003964 | 0.728668666 |
| ccr-miR-144 | 1.220393051 | 12.25878334 | 4.565991844 | 0 | 3.328398026 | 0.000398829 | -1.424815614 | 1.911865198 | 1.903582413 |
| ccr-miR-181a | 164.1428653 | 267.2895504 | 279.4387009 | 0 | 0.703451395 | 0.016372844 | 0.064128403 | 0.775914948 | 0.767579798 |
| ccr-miR-181b | 111.0557676 | 84.12890527 | 120.2377852 | 6.59E-05 | -0.400610844 | 0 | 0.515216864 | 0.122940593 | 0.11460602 |
| ccr-miR-182-5p | 3.899951705 | 1.316800256 | 5.00273889 | 0.01211819 | -1.566419737 | 0.003237778 | 1.925681633 | 0.367597633 | 0.359261897 |
| ccr-miR-199-3p | 2805.988722 | 7025.244326 | 5230.191458 | 0 | 1.324039191 | 0 | -0.425684642 | 0.906689864 | 0.898354549 |
| ccr-miR-199-5p | 2738.044665 | 3922.747964 | 3296.804929 | 0 | 0.518718664 | 0 | -0.25079612 | 0.276257871 | 0.267922544 |
| ccr-miR-200a | 2.745884364 | 0.961473203 | 7.305586951 | 0.035314737 | -1.513952317 | 1.86E-05 | 2.925681633 | 1.420080743 | 1.411729316 |
| ccr-miR-214 | 49.90765265 | 104.6740695 | 87.13834962 | 0 | 1.068571138 | 0.004026375 | -0.264524399 | 0.812382783 | 0.804046739 |
| ccr-miR-221 | 49.23322491 | 54.72806679 | 58.87726325 | 0.021091694 | 0.152648653 | 0.036892302 | 0.105429717 | 0.266414929 | 0.25807837 |
| ccr-miR-222 | 71.11840503 | 45.17722213 | 56.57263895 | 3.82E-06 | -0.654627405 | 0.007752327 | 0.324508906 | -0.321782842 | -0.330118499 |
| ccr-miR-30d | 671.6273973 | 625.0411883 | 683.1517884 | 0.000888661 | -0.10370982 | 0.000202054 | 0.128254902 | 0.032880351 | 0.024545082 |
| cfa-let-7e | 195.1242071 | 330.1677128 | 309.44972 | 0 | 0.758806276 | 0.007044616 | -0.093494029 | 0.673647322 | 0.665312247 |
| cfa-miR-107 | 12.20393051 | 19.46983236 | 14.00237499 | 0.003569383 | 0.673894592 | 0.019603432 | -0.475568914 | 0.206665022 | 0.198325678 |
| cfa-miR-10a | 6.407063516 | 18.81428245 | 4.939572995 | 0 | 1.55409307 | 0 | -1.929370031 | -0.366928358 | -0.37527696 |
| cfa-miR-125a | 217.2576992 | 321.4488989 | 248.1409022 | 0 | 0.565182105 | 0 | -0.373429848 | 0.200088049 | 0.191752257 |
| cfa-miR-1296 | 2.562825406 | 2.221003099 | 2.301259889 | 0.095584316 | -0.206523792 | 0.126621282 | 0.051212516 | -0.14700378 | -0.155311276 |
| cfa-miR-132 | 1.671407874 | 2.194667094 | 2.223439507 | 0.098271658 | 0.392938279 | 0.129388308 | 0.018791038 | 0.420073807 | 0.411729316 |
| cfa-miR-133c | 4.271375677 | 4.326629414 | 5.783589669 | 0.084677256 | 0.018542764 | 0.077461386 | 0.418721645 | 0.445591505 | 0.437264409 |
| cfa-miR-134 | 22.42472231 | 33.26915799 | 24.40730186 | 0.000991945 | 0.569095234 | 0.006669913 | -0.446872535 | 0.130557881 | 0.122222699 |
| cfa-miR-1343 | 1.949975853 | 2.414133803 | 1.66757963 | 0.098117594 | 0.308049381 | 0.11343125 | -0.533749985 | -0.217364868 | -0.225700604 |
| cfa-miR-140 | 437.5109087 | 386.5122276 | 584.4469561 | 0.000121179 | -0.178804933 | 0 | 0.596558037 | 0.426088335 | 0.417753104 |
| cfa-miR-1468 | 55.15645983 | 39.7234744 | 57.80942718 | 0.000410196 | -0.473538049 | 0.000136621 | 0.541312964 | 0.076110319 | 0.067774915 |
| cfa-miR-146a | 54.16880973 | 83.28761621 | 75.83697363 | 0 | 0.620639611 | 0.021556303 | -0.135200609 | 0.493774717 | 0.485439003 |
| cfa-miR-146b | 19.51242071 | 45.65905131 | 13.94702963 | 0 | 1.226508115 | 0 | -1.710942987 | -0.476102297 | -0.484434873 |
| cfa-miR-152 | 655.9612647 | 1295.825509 | 1388.974719 | 0 | 0.982188934 | 8.31E-05 | 0.100148877 | 1.090673019 | 1.082337811 |
| cfa-miR-15b | 36.19990886 | 42.65335497 | 36.11699549 | 0.015748197 | 0.236673158 | 0.023590355 | -0.239981341 | 0.005025258 | -0.003308183 |
| cfa-miR-181a | 156.9730561 | 255.1400254 | 266.7369417 | 0 | 0.700772294 | 0.016372844 | 0.064128403 | 0.773235986 | 0.764900698 |
| cfa-miR-181b | 116.608556 | 88.33535053 | 126.2496745 | 6.59E-05 | -0.400610844 | 0 | 0.515216864 | 0.122940593 | 0.11460602 |
| cfa-miR-181c | 0 | 0.438933419 | 0.277929938 | 0.152468748 | -1.187925979 | 0.247312928 | -0.659280867 | 21.48197676 | -1.847206846 |
| cfa-miR-1839 | 22.13349215 | 39.69354712 | 35.73926344 | 3.82E-06 | 0.842673399 | 0.035936429 | -0.15139459 | 0.699617621 | 0.691278809 |
| cfa-miR-1842 | 1.525491313 | 1.201841504 | 1.826396738 | 0.120876304 | -0.344027316 | 0.12955274 | 0.603753539 | 0.268033719 | 0.259726223 |
| cfa-miR-191 | 266.4755962 | 314.3361824 | 285.9141075 | 7.39E-06 | 0.238304929 | 0.002371997 | -0.13672654 | 0.109913694 | 0.101578389 |
| cfa-miR-192 | 60.71455427 | 66.34165101 | 64.22828528 | 0.018850642 | 0.127872521 | 0.038332273 | -0.046706135 | 0.08950211 | 0.081166385 |
| cfa-miR-193b | 1.747380959 | 1.835539751 | 1.452815587 | 0.113865386 | 0.071010184 | 0.138855988 | -0.337352772 | -0.257985786 | -0.266342589 |
| cfa-miR-19b | 22.71595247 | 13.9959906 | 14.81871899 | 0.00150897 | -0.698692197 | 0.065739081 | 0.082407137 | -0.607954754 | -0.61628506 |
| cfa-miR-200a | 0.29123016 | 0.458884938 | 2.324504939 | 0.195628342 | 0.655972684 | 0.02557176 | 2.340719133 | 3.005118679 | 2.996691817 |
| cfa-miR-223 | 0.915294788 | 1.922946406 | 1.826396738 | 0.081065193 | 1.071010184 | 0.141457698 | -0.074318367 | 1.005098158 | 0.996691817 |
| cfa-miR-224 | 417.0415889 | 306.305696 | 274.2915828 | 0 | -0.445219066 | 0.001167965 | -0.159261844 | -0.596145814 | -0.604480911 |
| cfa-miR-23a | 2.360497085 | 14.08052204 | 14.46698469 | 0 | 2.576538217 | 0.072136911 | 0.039063433 | 2.623945022 | 2.61560165 |
| cfa-miR-23b | 1.348855477 | 6.641755679 | 8.074596103 | 0.000520625 | 2.299828874 | 0.078226006 | 0.281825444 | 2.589937103 | 2.581654318 |
| cfa-miR-24 | 209.7616881 | 265.7741851 | 272.3713396 | 0 | 0.341449779 | 0.02029911 | 0.035373922 | 0.385159058 | 0.376823702 |
| cfa-miR-29a | 49.21789701 | 57.36061723 | 49.10516683 | 0.01032411 | 0.220877533 | 0.016283355 | -0.224185716 | 0.00502699 | -0.003308183 |
| cfa-miR-301b | 1.392839895 | 3.73093406 | 5.00273889 | 0.01887205 | 1.421507431 | 0.080013708 | 0.423181293 | 1.85299622 | 1.844688724 |
| cfa-miR-30a | 191.3762015 | 437.1776851 | 381.3198754 | 0 | 1.191808328 | 1.86E-05 | -0.197218033 | 1.002926007 | 0.994590295 |
| cfa-miR-30c | 109.7209627 | 110.2088659 | 92.95598396 | 0.021768352 | 0.006401102 | 0.002440523 | -0.245620643 | -0.230883992 | -0.23921954 |
| cfa-miR-323 | 16.47530618 | 29.32493269 | 24.3519565 | 0.000102303 | 0.83182252 | 0.029271168 | -0.26809011 | 0.572067584 | 0.56373241 |
| cfa-miR-329b | 2.912301598 | 7.342159005 | 3.777320526 | 0.003505846 | 1.33404459 | 0.019637806 | -0.958841149 | 0.383498308 | 0.37520344 |
| cfa-miR-339 | 109.2572936 | 194.4706063 | 168.2207522 | 0 | 0.83182252 | 0.002015585 | -0.209196421 | 0.630960979 | 0.622626099 |
| cfa-miR-33a | 0.320353176 | 1.261933579 | 1.278477716 | 0.085038376 | 1.977900779 | 0.167574911 | 0.018791038 | 2.005057116 | 1.996691817 |
| cfa-miR-345 | 1.830589576 | 2.884419609 | 1.826396738 | 0.077430733 | 0.655972684 | 0.099764444 | -0.659280867 | 0.005015787 | -0.003308183 |
| cfa-miR-34c | 0.835703937 | 0.658400128 | 0.277929938 | 0.149911551 | -0.344027316 | 0.192756666 | -1.244243368 | -1.580028518 | -1.588270684 |
| cfa-miR-374a | 117.7679294 | 164.8926543 | 162.8537091 | 0 | 0.485580413 | 0.027499043 | -0.017950552 | 0.475965315 | 0.467629861 |
| cfa-miR-375 | 0 | 0.458884938 | 0.290563117 | 0.152468748 | -1.123795642 | 0.247312928 | -0.659280867 | 21.48197676 | -1.783076509 |
| cfa-miR-377 | 0 | 0.229442469 | 0 | 0.25085307 | -2.123795642 | 0.315638314 | 2.123795642 | -- | 0 |
| cfa-miR-382 | 71.64261931 | 126.8816853 | 108.3800428 | 0 | 0.824593849 | 0.003032963 | -0.227384717 | 0.605544659 | 0.597209132 |
| cfa-miR-3958 | 158.783748 | 214.8579085 | 176.2075809 | 0 | 0.436319625 | 2.94E-05 | -0.286106887 | 0.158548136 | 0.150212738 |
| cfa-miR-421 | 0.58246032 | 1.376654813 | 2.033941822 | 0.095170799 | 1.240935185 | 0.124855427 | 0.563111554 | 1.812429628 | 1.804046739 |
| cfa-miR-425 | 55.71359579 | 38.84560756 | 40.577771 | 0.000160015 | -0.520277955 | 0.044153203 | 0.062938142 | -0.44900311 | -0.457339814 |
| cfa-miR-452 | 338.4094457 | 221.18254 | 208.6243183 | 0 | -0.613532333 | 0.0144392 | -0.08433017 | -0.689527393 | -0.697862502 |
| cfa-miR-454 | 27.95809534 | 17.66707011 | 21.21110757 | 0.000905646 | -0.662203276 | 0.039154871 | 0.263757151 | -0.390107361 | -0.398446125 |
| cfa-miR-486 | 28.37413843 | 180.0358573 | 74.27346733 | 0 | 2.665635782 | 0 | -1.277365438 | 1.396605648 | 1.388270344 |
| cfa-miR-493 | 12.81412703 | 31.7286157 | 16.13317118 | 0 | 1.308049381 | 1.86E-05 | -0.975754532 | 0.340633683 | 0.332294849 |
| cfa-miR-497 | 48.81572203 | 76.19675134 | 59.96669289 | 0 | 0.64238362 | 0.002116266 | -0.345568078 | 0.305151082 | 0.296815542 |
| cfa-miR-500 | 6.407063516 | 11.29731014 | 12.48037771 | 0.00812763 | 0.818244113 | 0.06857092 | 0.143682286 | 0.97026614 | 0.961926399 |
| cfa-miR-542 | 284.240636 | 528.1765634 | 333.5664587 | 0 | 0.893907465 | 0 | -0.663046059 | 0.239196884 | 0.230861406 |
| cfa-miR-582 | 0.58246032 | 1.606097282 | 0.290563117 | 0.073694749 | 1.463327607 | 0.057483806 | -2.466635789 | -0.995066018 | -1.003308183 |
| cfa-miR-590 | 9.458046142 | 9.133995429 | 15.21997281 | 0.063741593 | -0.050296112 | 0.008405259 | 0.736647809 | 0.694681607 | 0.686351697 |
| cfa-miR-652 | 5.242142877 | 7.571601474 | 13.07534028 | 0.038490582 | 0.530441802 | 0.008589925 | 0.78817811 | 1.326943882 | 1.318619912 |
| cfa-miR-6529 | 0.873690479 | 0.229442469 | 0 | 0.107210574 | -1.928989816 | 0.315638314 | 2.123795642 | -23.06200528 | 0.194805825 |
| cfa-miR-93 | 156.8337721 | 124.4376242 | 117.8422939 | 3.16E-05 | -0.333813503 | 0.022781293 | -0.078565338 | -0.404044019 | -0.41237884 |
| cgr-let-7d-3p | 13.10535719 | 19.27316739 | 16.27153457 | 0.007156846 | 0.556437011 | 0.043507241 | -0.244243368 | 0.320526035 | 0.312193643 |
| cgr-let-7g-3p | 0.305098263 | 0.240368301 | 0 | 0.227853908 | -0.344027316 | 0.315638314 | 2.056681446 | -21.47704278 | 1.71265413 |
| cgr-let-7i | 1.525491313 | 5.047734316 | 4.261592388 | 0.005402066 | 1.726362012 | 0.096088965 | -0.244243368 | 1.490437866 | 1.482118644 |
| cgr-miR-100-3p | 0.29123016 | 0.688327407 | 1.743378704 | 0.152183516 | 1.240935185 | 0.08996163 | 1.340719133 | 2.590060659 | 2.581654318 |
| cgr-miR-103-5p | 0 | 0.210322263 | 0 | 0.25085307 | -2.249326524 | 0.315638314 | 2.249326524 | -- | 0 |
| cgr-miR-106b-3p | 65.2355558 | 83.51705868 | 75.83697363 | 0.00045506 | 0.356412403 | 0.02096209 | -0.139169511 | 0.22557799 | 0.217242892 |
| cgr-miR-106b-5p | 34.0739287 | 32.12194565 | 31.38081667 | 0.03580795 | -0.085109018 | 0.049805356 | -0.033676382 | -0.110450534 | -0.1187854 |
| cgr-miR-10b-3p | 0.58246032 | 2.06498222 | 1.452815587 | 0.042078378 | 1.825897686 | 0.126492745 | -0.507277774 | 1.327059086 | 1.318619912 |
| cgr-miR-125a-3p | 0.915294788 | 1.922946406 | 0.913198369 | 0.081065193 | 1.071010184 | 0.104224791 | -1.074318367 | 0.005098158 | -0.003308183 |
| cgr-miR-125a-5p | 199.152891 | 294.6614907 | 227.4624937 | 0 | 0.565182105 | 0 | -0.373429848 | 0.200088049 | 0.191752257 |
| cgr-miR-125b-3p | 17.18257943 | 48.64180341 | 35.73926344 | 0 | 1.50125009 | 0.002121429 | -0.444686816 | 1.064898227 | 1.056563273 |
| cgr-miR-128-5p | 0 | 0 | 0.277929938 | -- | 0 | 0.26087974 | -1.847206846 | 21.48197676 | -1.847206846 |
| cgr-miR-1306-3p | 0.305098263 | 0.961473203 | 0.913198369 | 0.114580875 | 1.655972684 | 0.185895435 | -0.074318367 | 1.590060659 | 1.581654318 |
| cgr-miR-130b-5p | 26.21071438 | 18.12595504 | 19.46772886 | 0.00416936 | -0.532099664 | 0.057911611 | 0.103027575 | -0.420737727 | -0.429072089 |
| cgr-miR-132-5p | 1.164920639 | 1.606097282 | 1.452815587 | 0.11418857 | 0.463327607 | 0.148131301 | -0.144707694 | 0.326935531 | 0.318619912 |
| cgr-miR-139-3p | 0.835703937 | 1.097333547 | 1.945509568 | 0.134856116 | 0.392938279 | 0.108711 | 0.82614596 | 1.227467127 | 1.219084239 |
| cgr-miR-139-5p | 37.56869062 | 55.29563501 | 52.30136112 | 7.53E-05 | 0.557634765 | 0.03761889 | -0.080317107 | 0.485653942 | 0.477317658 |
| cgr-miR-140-3p | 2937.347392 | 2033.319159 | 2443.635817 | 0 | -0.530677214 | 0 | 0.265192606 | -0.257149323 | -0.265484608 |
| cgr-miR-144 | 4.659682557 | 27.76253874 | 10.75083534 | 0 | 2.574835922 | 0 | -1.368690739 | 1.214498619 | 1.206145183 |
| cgr-miR-146b-3p | 0.29123016 | 0.458884938 | 0 | 0.195628342 | 0.655972684 | 0.193791884 | 1.123795642 | -21.47704278 | 1.779768326 |
| cgr-miR-146b-5p | 18.66405459 | 43.67387517 | 13.34063704 | 0 | 1.226508115 | 0 | -1.710942987 | -0.476102297 | -0.484434873 |
| cgr-miR-152-5p | 16.43551076 | 10.75386876 | 18.89923581 | 0.00749107 | -0.611960521 | 0.001682804 | 0.81347213 | 0.209844973 | 0.201511609 |
| cgr-miR-15a-5p | 5.606180576 | 7.150956948 | 7.724136203 | 0.051742827 | 0.351118103 | 0.082310071 | 0.111237287 | 0.470696787 | 0.46235539 |
| cgr-miR-15b-3p | 5.796866991 | 5.047734316 | 5.479190213 | 0.072748996 | -0.199637406 | 0.097969644 | 0.118326711 | -0.07298239 | -0.081310695 |
| cgr-miR-16-3p | 1.114271916 | 0.658400128 | 1.111719753 | 0.121667271 | -0.759064815 | 0.151642571 | 0.755756632 | 0.004933561 | -0.003308183 |
| cgr-miR-181a-3p | 41.06345253 | 20.19093726 | 42.13165202 | 0 | -1.024147049 | 0 | 1.061196604 | 0.045384551 | 0.037049555 |
| cgr-miR-181b-3p | 1.011641608 | 1.85969159 | 2.35509053 | 0.098534335 | 0.878365106 | 0.131350463 | 0.340719133 | 1.227467127 | 1.219084239 |
| cgr-miR-181c-3p | 0.29123016 | 0.917769876 | 1.452815587 | 0.114580875 | 1.655972684 | 0.139046575 | 0.662647228 | 2.327059086 | 2.318619912 |
| cgr-miR-181c-5p | 0 | 0.458884938 | 0.290563117 | 0.152468748 | -1.123795642 | 0.247312928 | -0.659280867 | 21.48197676 | -1.783076509 |
| cgr-miR-183 | 0.873690479 | 1.147212345 | 3.777320526 | 0.134856116 | 0.392938279 | 0.016344202 | 1.719230756 | 2.120562747 | 2.112169035 |
| cgr-miR-1839-3p | 2.912301598 | 2.523867158 | 2.905631174 | 0.095584316 | -0.206523792 | 0.121297295 | 0.203215609 | 0.004982896 | -0.003308183 |
| cgr-miR-1839-5p | 22.5640063 | 39.28454098 | 35.29710217 | 7.39E-06 | 0.799938459 | 0.034540496 | -0.154411958 | 0.653862208 | 0.645526501 |
| cgr-miR-186-5p | 474.1227002 | 651.3771935 | 528.9006727 | 0 | 0.458232739 | 0 | -0.300496398 | 0.166071623 | 0.157736342 |
| cgr-miR-187 | 1.525491313 | 5.528470918 | 1.521997281 | 0.002673633 | 1.857606546 | 0.004639677 | -1.860914728 | 0.005032146 | -0.003308183 |
| cgr-miR-188 | 0.320353176 | 2.019093726 | 1.598097145 | 0.030308445 | 2.655972684 | 0.138855988 | -0.337352772 | 2.327059086 | 2.318619912 |
| cgr-miR-18a-3p | 0.835703937 | 0.877866838 | 0.833789815 | 0.147179596 | 0.071010184 | 0.185895435 | -0.074318367 | 0.005098158 | -0.003308183 |
| cgr-miR-190a | 2.135687839 | 2.103222632 | 0.799048573 | 0.104090671 | -0.022099221 | 0.065634915 | -1.396246461 | -1.41000112 | -1.418345682 |
| cgr-miR-191-3p | 3.050982627 | 1.682578105 | 0.913198369 | 0.062736997 | -0.858600488 | 0.126084122 | -0.881673289 | -1.731966281 | -1.740273777 |
| cgr-miR-192 | 58.82849228 | 63.78500636 | 62.47107023 | 0.01999447 | 0.116702274 | 0.039166272 | -0.03002909 | 0.095009179 | 0.086673184 |
| cgr-miR-193b-3p | 33.49146838 | 42.90574169 | 35.73926344 | 0.004713525 | 0.357377093 | 0.018173893 | -0.263660822 | 0.102053557 | 0.093716272 |
| cgr-miR-19b-5p | 0.278567979 | 0.219466709 | 0.555859877 | 0.227853908 | -0.344027316 | 0.200950336 | 1.340719133 | 1.005180239 | 0.996691817 |
| cgr-miR-200b | 10.02844724 | 2.853067222 | 25.56955433 | 3.45E-05 | -1.813512599 | 0 | 3.163841371 | 1.358668254 | 1.350328772 |
| cgr-miR-205 | 5.395421908 | 3.188042726 | 14.80342619 | 0.04398532 | -0.759064815 | 0 | 2.215188251 | 1.464471729 | 1.456123436 |
| cgr-miR-210-5p | 0.29123016 | 0.229442469 | 0 | 0.227853908 | -0.344027316 | 0.315638314 | 2.123795642 | -21.47704278 | 1.779768326 |
| cgr-miR-221-3p | 40.67092493 | 45.21014213 | 48.63773921 | 0.021091694 | 0.152648653 | 0.036892302 | 0.105429717 | 0.266414929 | 0.25807837 |
| cgr-miR-221-5p | 11.33557391 | 7.571601474 | 11.80133277 | 0.014834373 | -0.582187053 | 0.015552345 | 0.640279415 | 0.066423494 | 0.058092362 |
| cgr-miR-222-3p | 57.15100656 | 36.1417777 | 45.5138067 | 3.82E-06 | -0.661111438 | 0.006958934 | 0.332636786 | -0.320139053 | -0.328474652 |
| cgr-miR-22-5p | 16.47530618 | 31.2478791 | 34.0927391 | 1.80E-05 | 0.923452995 | 0.043986271 | 0.125706242 | 1.05749529 | 1.049159237 |
| cgr-miR-23a-5p | 0.29123016 | 0 | 0 | 0.207164791 | 1.779768326 | -- | 0 | -21.47704278 | 1.779768326 |
| cgr-miR-23b-5p | 1.922119055 | 0.757160147 | 2.556955433 | 0.07063155 | -1.344027316 | 0.043987577 | 1.755756632 | 0.420073807 | 0.411729316 |
| cgr-miR-25-5p | 0.557135958 | 0.438933419 | 0.555859877 | 0.174900687 | -0.344027316 | 0.221716937 | 0.340719133 | 0.005180239 | -0.003308183 |
| cgr-miR-26b-3p | 0 | 0.229442469 | 0.290563117 | 0.25085307 | -2.123795642 | 0.286090674 | 0.340719133 | 21.48197676 | -1.783076509 |
| cgr-miR-28-5p | 30.204728 | 49.75623826 | 50.53030974 | 1.09E-05 | 0.720103022 | 0.044068038 | 0.022271607 | 0.750708064 | 0.742374628 |
| cgr-miR-29a-3p | 53.39219597 | 60.81318009 | 51.74790757 | 0.013536059 | 0.187755147 | 0.015483172 | -0.232883506 | -0.036791583 | -0.045128359 |
| cgr-miR-29a-5p | 0.26696098 | 0.210322263 | 0.266349524 | 0.227853908 | -0.344027316 | 0.286090674 | 0.340719133 | 0.004933982 | -0.003308183 |
| cgr-miR-29b-5p | 0.53392196 | 0 | 0 | 0.10800582 | 0.905299208 | -- | 0 | -22.47704278 | 0.905299208 |
| cgr-miR-29c-5p | 2.507111811 | 2.633600513 | 2.501369445 | 0.098568343 | 0.071010184 | 0.125959855 | -0.074318367 | 0.005043243 | -0.003308183 |
| cgr-miR-301a-5p | 3.785992078 | 2.06498222 | 2.324504939 | 0.047340275 | -0.874542032 | 0.129815067 | 0.170794131 | -0.695397075 | -0.703747901 |
| cgr-miR-30d | 0 | 0.229442469 | 0.290563117 | 0.25085307 | -2.123795642 | 0.286090674 | 0.340719133 | 21.48197676 | -1.783076509 |
| cgr-miR-32-3p | 4.271375677 | 2.884419609 | 3.348394019 | 0.063945412 | -0.566419737 | 0.115686484 | 0.215188251 | -0.342880825 | -0.351231486 |
| cgr-miR-324-5p | 1.164920639 | 2.294424689 | 2.615068056 | 0.070826985 | 0.977900779 | 0.126579328 | 0.188716039 | 1.174899604 | 1.166616819 |
| cgr-miR-32-5p | 25.93335233 | 37.25708662 | 24.65635596 | 0.001294654 | 0.522706154 | 0.00112193 | -0.59555527 | -0.064512283 | -0.072849116 |
| cgr-miR-331-3p | 3.203531758 | 4.290574169 | 4.474672007 | 0.074081236 | 0.421507431 | 0.108830484 | 0.060611214 | 0.490437866 | 0.482118644 |
| cgr-miR-331-5p | 7.932554829 | 8.412890527 | 8.827584232 | 0.065562575 | 0.084815983 | 0.083535891 | 0.069417111 | 0.162569061 | 0.154233094 |
| cgr-miR-339 | 107.2486719 | 186.7661697 | 157.3083451 | 0 | 0.800273371 | 0.000329 | -0.247637946 | 0.560971081 | 0.552635424 |
| cgr-miR-362 | 27.10680718 | 25.62695884 | 27.78230422 | 0.037121992 | -0.08099291 | 0.043679536 | 0.116503976 | 0.043842479 | 0.035511066 |
| cgr-miR-369-5p | 14.85273815 | 33.03971552 | 21.50167068 | 0 | 1.153472344 | 0.001163766 | -0.619752503 | 0.542050874 | 0.533719841 |
| cgr-miR-374-3p | 6.407063516 | 6.424389129 | 8.135767286 | 0.071775884 | 0.003895988 | 0.065745277 | 0.340719133 | 0.352965793 | 0.344615121 |
| cgr-miR-377-3p | 0.835703937 | 0.219466709 | 0.277929938 | 0.107210574 | -1.928989816 | 0.286090674 | 0.340719133 | -1.580028518 | -1.588270684 |
| cgr-miR-378-5p | 6.698293676 | 4.12996444 | 6.101825465 | 0.031593612 | -0.69766427 | 0.05830046 | 0.563111554 | -0.12621084 | -0.134552716 |
| cgr-miR-409-3p | 168.0398022 | 193.6494438 | 145.2815587 | 0.000571107 | 0.204644365 | 0 | -0.414595771 | -0.201616302 | -0.209951407 |
| cgr-miR-409-5p | 56.8626887 | 73.61279211 | 54.33530295 | 0.000394132 | 0.372474176 | 0.000204992 | -0.438066637 | -0.057257956 | -0.065592461 |
| cgr-miR-412-3p | 1.456150799 | 0.917769876 | 0.581126235 | 0.112137039 | -0.66595541 | 0.181619692 | -0.659280867 | -1.316846701 | -1.325236278 |
| cgr-miR-423-3p | 390.2737385 | 337.3203323 | 294.0498748 | 2.14E-05 | -0.210367106 | 0.000108927 | -0.198058405 | -0.400090191 | -0.408425511 |
| cgr-miR-425-3p | 1.525491313 | 1.922946406 | 1.217597825 | 0.111252526 | 0.33404459 | 0.125444122 | -0.659280867 | -0.316969824 | -0.325236278 |
| cgr-miR-505-3p | 10.86415118 | 7.900801538 | 12.50684723 | 0.03045629 | -0.459504533 | 0.015942144 | 0.662647228 | 0.211473 | 0.203142695 |
| cgr-miR-505-5p | 0 | 0.438933419 | 0 | 0.152468748 | -1.187925979 | 0.193791884 | 1.187925979 | -- | 0 |
| cgr-miR-542-3p | 276.6180031 | 510.2600993 | 322.3987285 | 0 | 0.883337778 | 0 | -0.662386778 | 0.229286222 | 0.220951 |
| cgr-miR-652-5p | 2.819107947 | 2.221003099 | 4.602519779 | 0.085702667 | -0.344027316 | 0.028315176 | 1.051212516 | 0.715501681 | 0.7071852 |
| cgr-miR-671-5p | 16.81854173 | 16.82578105 | 9.588582873 | 0.048717586 | 0.000620856 | 0.00245766 | -0.811283961 | -0.802327368 | -0.810663105 |
| cgr-miR-708 | 49.21789701 | 47.72403353 | 57.82206035 | 0.032182244 | -0.044467034 | 0.010295439 | 0.276904035 | 0.240774356 | 0.232437001 |
| cgr-miR-744-3p | 0.610196525 | 0.480736602 | 0 | 0.174900687 | -0.344027316 | 0.193791884 | 1.056681446 | -22.47704278 | 0.71265413 |
| cgr-miR-744-5p | 16.78040445 | 17.30651765 | 13.69797553 | 0.05014726 | 0.044537972 | 0.03885046 | -0.337352772 | -0.284480347 | -0.2928148 |
| cgr-miR-874 | 3.899951705 | 5.925601154 | 5.280668829 | 0.041573862 | 0.603505265 | 0.090237063 | -0.166240856 | 0.445591505 | 0.437264409 |
| cgr-miR-92b-5p | 1.334804899 | 0.841289053 | 0.532699048 | 0.112137039 | -0.66595541 | 0.181619692 | -0.659280867 | -1.316846701 | -1.325236278 |
| cgr-miR-99a-3p | 2.038611119 | 7.571601474 | 5.52069923 | 0.00035091 | 1.893011882 | 0.05833991 | -0.455747473 | 1.445591505 | 1.437264409 |
| chi-let-7a-3p | 38.15115094 | 32.58083059 | 38.06376837 | 0.019362468 | -0.227703198 | 0.027004051 | 0.224395015 | 0.005027716 | -0.003308183 |
| chi-let-7d-3p | 13.72942182 | 20.19093726 | 17.04636955 | 0.007156846 | 0.556437011 | 0.043507241 | -0.244243368 | 0.320526035 | 0.312193643 |
| chi-let-7e-3p | 1.747380959 | 1.835539751 | 1.452815587 | 0.113865386 | 0.071010184 | 0.138855988 | -0.337352772 | -0.257985786 | -0.266342589 |
| chi-let-7f-3p | 3.785992078 | 2.982752096 | 3.486757408 | 0.07992777 | -0.344027316 | 0.11106765 | 0.225241915 | -0.110455095 | -0.1187854 |
| chi-miR-100-3p | 0.305098263 | 0.721104902 | 1.826396738 | 0.152183516 | 1.240935185 | 0.08996163 | 1.340719133 | 2.590060659 | 2.581654318 |
| chi-miR-106b-3p | 71.75911138 | 91.86876455 | 83.42067099 | 0.00045506 | 0.356412403 | 0.02096209 | -0.139169511 | 0.22557799 | 0.217242892 |
| chi-miR-1185-3p | 9.901825434 | 9.407141225 | 6.973514817 | 0.061586905 | -0.073938152 | 0.050249912 | -0.431870371 | -0.497474863 | -0.505808523 |
| chi-miR-1185-5p | 0 | 0.504773432 | 0.319619429 | 0.152468748 | -0.986292118 | 0.247312928 | -0.659280867 | 21.48197676 | -1.645572985 |
| chi-miR-125b-3p | 17.69569923 | 50.95807976 | 37.13673367 | 0 | 1.525912144 | 0.001792683 | -0.456463984 | 1.077783192 | 1.06944816 |
| chi-miR-125b-5p | 322.2752948 | 965.8839614 | 613.3496844 | 0 | 1.58355628 | 0 | -0.655140057 | 0.936751312 | 0.928416224 |
| chi-miR-126-3p | 2425.836286 | 3089.694138 | 3206.848272 | 0 | 0.348981837 | 0.000346091 | 0.053692069 | 0.411009162 | 0.402673906 |
| chi-miR-126-5p | 18.92996039 | 30.05696343 | 29.63743797 | 0.000510414 | 0.667027873 | 0.05149775 | -0.020278527 | 0.65508448 | 0.646749346 |
| chi-miR-1271-5p | 100.270544 | 97.67365901 | 126.5692939 | 0.024560966 | -0.037856406 | 0.000127698 | 0.373885997 | 0.344365449 | 0.33602959 |
| chi-miR-127-3p | 787.1077529 | 1363.393039 | 1236.287952 | 0 | 0.792568467 | 0 | -0.141186712 | 0.659717064 | 0.651381754 |
| chi-miR-127-5p | 23.58964294 | 47.72403353 | 36.61095279 | 0 | 1.0165624 | 0.004800202 | -0.382440662 | 0.642458768 | 0.634121738 |
| chi-miR-1306-3p | 0.29123016 | 0.917769876 | 0.871689352 | 0.114580875 | 1.655972684 | 0.185895435 | -0.074318367 | 1.590060659 | 1.581654318 |
| chi-miR-1307-3p | 68.24915484 | 51.35521 | 58.08735711 | 0.000473487 | -0.410300535 | 0.022194503 | 0.177713545 | -0.224252146 | -0.23258699 |
| chi-miR-133a-3p | 3.899951705 | 3.950400769 | 5.280668829 | 0.084677256 | 0.018542764 | 0.077461386 | 0.418721645 | 0.445591505 | 0.437264409 |
| chi-miR-133a-5p | 0 | 0.229442469 | 0.871689352 | 0.25085307 | -2.123795642 | 0.132213553 | 1.925681633 | 23.06710344 | -0.198114008 |
| chi-miR-1343 | 2.038611119 | 2.523867158 | 1.743378704 | 0.098117594 | 0.308049381 | 0.11343125 | -0.533749985 | -0.217364868 | -0.225700604 |
| chi-miR-136-3p | 80.24084308 | 190.6120625 | 124.4993776 | 0 | 1.248230751 | 0 | -0.61450089 | 0.642064245 | 0.633729861 |
| chi-miR-136-5p | 26.7931747 | 62.63779401 | 31.67137979 | 0 | 1.225167869 | 0 | -0.983853683 | 0.24964833 | 0.241314186 |
| chi-miR-144-3p | 1.348855477 | 13.54918159 | 5.046622565 | 0 | 3.328398026 | 0.000398829 | -1.424815614 | 1.911865198 | 1.903582413 |
| chi-miR-144-5p | 4.881572203 | 29.08456439 | 10.95838043 | 0 | 2.574835922 | 0 | -1.408219103 | 1.174961381 | 1.166616819 |
| chi-miR-145-3p | 39.05257762 | 47.35255525 | 48.39951355 | 0.009177948 | 0.278024504 | 0.0436536 | 0.031550269 | 0.317910849 | 0.309574772 |
| chi-miR-146b-3p | 0.278567979 | 0.438933419 | 0 | 0.195628342 | 0.655972684 | 0.193791884 | 1.187925979 | -21.47704278 | 1.843898663 |
| chi-miR-148a-5p | 530.6213512 | 245.0445568 | 423.6410251 | 0 | -1.114638628 | 0 | 0.789798205 | -0.316505028 | -0.324840422 |
| chi-miR-154b-3p | 1.114271916 | 1.755733675 | 2.779299383 | 0.09921642 | 0.655972684 | 0.094435052 | 0.662647228 | 1.326886281 | 1.318619912 |
| chi-miR-154b-5p | 12.23166671 | 19.04372492 | 10.16970911 | 0.004217595 | 0.638694693 | 0.001012658 | -0.905037282 | -0.258002162 | -0.266342589 |
| chi-miR-15a-5p | 5.849927558 | 7.461868119 | 8.059968212 | 0.051742827 | 0.351118103 | 0.082310071 | 0.111237287 | 0.470696787 | 0.46235539 |
| chi-miR-16b-3p | 1.456150799 | 2.753309627 | 1.743378704 | 0.063235077 | 0.91900709 | 0.099764444 | -0.659280867 | 0.268033719 | 0.259726223 |
| chi-miR-17-3p | 0.800882939 | 1.261933579 | 1.331747621 | 0.115981432 | 0.655972684 | 0.153188595 | 0.077684727 | 0.742096585 | 0.733657411 |
| chi-miR-1814 | 0.355947973 | 0.560859368 | 0.710265398 | 0.195628342 | 0.655972684 | 0.221716937 | 0.340719133 | 1.005180239 | 0.996691817 |
| chi-miR-181b-5p | 103.3487202 | 77.69121512 | 111.1719753 | 4.03E-05 | -0.411697142 | 0 | 0.516969772 | 0.113607654 | 0.10527263 |
| chi-miR-181c-5p | 0 | 0.480736602 | 0.304399456 | 0.152468748 | -1.056681446 | 0.247312928 | -0.659280867 | 21.48197676 | -1.715962313 |
| chi-miR-187 | 1.601765879 | 5.804894463 | 1.598097145 | 0.002673633 | 1.857606546 | 0.004639677 | -1.860914728 | 0.005032146 | -0.003308183 |
| chi-miR-188-5p | 0.305098263 | 1.922946406 | 1.521997281 | 0.030308445 | 2.655972684 | 0.138855988 | -0.337352772 | 2.327059086 | 2.318619912 |
| chi-miR-193a | 38.77959496 | 49.41466225 | 41.04586353 | 0.00519415 | 0.349641445 | 0.017768528 | -0.267702341 | 0.090277767 | 0.081939104 |
| chi-miR-195-3p | 54.00239249 | 37.49745492 | 43.22472279 | 0.000295349 | -0.526230647 | 0.027499429 | 0.205064033 | -0.312833351 | -0.321166613 |
| chi-miR-20b | 2.78567979 | 1.975200385 | 2.223439507 | 0.083536891 | -0.496030409 | 0.129815067 | 0.170794131 | -0.316908261 | -0.325236278 |
| chi-miR-211 | 0 | 0.229442469 | 1.743378704 | 0.25085307 | -2.123795642 | 0.033904916 | 2.925681633 | 24.06710344 | 0.801885992 |
| chi-miR-21-3p | 2.745884364 | 1.682578105 | 2.130796194 | 0.076961348 | -0.706597395 | 0.131350463 | 0.340719133 | -0.357550289 | -0.365878262 |
| chi-miR-214-3p | 310.7425805 | 481.0490803 | 444.2710064 | 0 | 0.630464151 | 0.00211948 | -0.114744104 | 0.524055425 | 0.515720048 |
| chi-miR-215-5p | 1.525491313 | 4.326629414 | 6.696788038 | 0.014170498 | 1.503969591 | 0.049164994 | 0.63022575 | 2.142495375 | 2.134195341 |
| chi-miR-21-5p | 59955.35041 | 43619.44742 | 45821.19721 | 0 | -0.458917011 | 0 | 0.07104366 | -0.379538063 | -0.387873351 |
| chi-miR-221-5p | 14.03452008 | 9.37436373 | 14.6111739 | 0.014834373 | -0.582187053 | 0.015552345 | 0.640279415 | 0.066423494 | 0.058092362 |
| chi-miR-22-3p | 148.7113163 | 331.2907733 | 271.508294 | 0 | 1.155583589 | 0 | -0.287101754 | 0.876817871 | 0.868481835 |
| chi-miR-224-3p | 0.29123016 | 0.458884938 | 0.290563117 | 0.195628342 | 0.655972684 | 0.247312928 | -0.659280867 | 0.004933982 | -0.003308183 |
| chi-miR-2331 | 0.640706352 | 1.009546863 | 0.319619429 | 0.143516961 | 0.655972684 | 0.143485591 | -1.659280867 | -0.995066018 | -1.003308183 |
| chi-miR-23b-5p | 1.747380959 | 0.688327407 | 2.324504939 | 0.07063155 | -1.344027316 | 0.043987577 | 1.755756632 | 0.420073807 | 0.411729316 |
| chi-miR-24-3p | 241.2259414 | 305.8926995 | 313.2270405 | 0 | 0.342640613 | 0.020420914 | 0.034183088 | 0.385159058 | 0.376823702 |
| chi-miR-24-5p | 4.805297637 | 6.309667895 | 7.990485727 | 0.060690946 | 0.392938279 | 0.071140764 | 0.340719133 | 0.742010988 | 0.733657411 |
| chi-miR-26a-3p | 0.873690479 | 0.917769876 | 1.452815587 | 0.147179596 | 0.071010184 | 0.139046575 | 0.662647228 | 0.742096585 | 0.733657411 |
| chi-miR-28-3p | 598.4779784 | 533.6831827 | 641.5633631 | 3.45E-05 | -0.165314613 | 0 | 0.265608208 | 0.108628727 | 0.100293595 |
| chi-miR-296-3p | 26.50194454 | 22.25591948 | 27.31293303 | 0.025074302 | -0.251909114 | 0.026887634 | 0.295395142 | 0.051819827 | 0.043486029 |
| chi-miR-29a-5p | 0.305098263 | 0.240368301 | 0.304399456 | 0.227853908 | -0.344027316 | 0.286090674 | 0.340719133 | 0.004933982 | -0.003308183 |
| chi-miR-29b-5p | 0 | 0.219466709 | 0.277929938 | 0.25085307 | -2.187925979 | 0.286090674 | 0.340719133 | 21.48197676 | -1.847206846 |
| chi-miR-29c-5p | 2.507111811 | 2.633600513 | 2.501369445 | 0.098568343 | 0.071010184 | 0.125959855 | -0.074318367 | 0.005043243 | -0.003308183 |
| chi-miR-301a-5p | 3.966277415 | 2.163314707 | 2.43519565 | 0.047340275 | -0.874542032 | 0.129815067 | 0.170794131 | -0.695397075 | -0.703747901 |
| chi-miR-30a-3p | 2.745884364 | 7.932153925 | 7.914385863 | 0.001397232 | 1.530441802 | 0.086925753 | -0.003235268 | 1.535564274 | 1.527206534 |
| chi-miR-30c-3p | 0.915294788 | 1.442209805 | 1.217597825 | 0.115981432 | 0.655972684 | 0.156876716 | -0.244243368 | 0.420094615 | 0.411729316 |
| chi-miR-30e-3p | 30.81492453 | 32.6900889 | 35.00593747 | 0.035820767 | 0.085224043 | 0.044992652 | 0.098746342 | 0.192306181 | 0.183970385 |
| chi-miR-30f-5p | 19.49975853 | 34.01733996 | 30.57229322 | 2.49E-05 | 0.802814073 | 0.038430546 | -0.154045559 | 0.657098683 | 0.648768514 |
| chi-miR-324-3p | 0 | 0.240368301 | 0 | 0.25085307 | -2.056681446 | 0.315638314 | 2.056681446 | -- | 0 |
| chi-miR-326-3p | 0 | 2.163314707 | 1.521997281 | 0.005399256 | 1.113243556 | 0.126492745 | -0.507277774 | 23.80410187 | 0.605965782 |
| chi-miR-326-5p | 0 | 0.252386716 | 0.319619429 | 0.25085307 | -1.986292118 | 0.286090674 | 0.340719133 | 21.48197676 | -1.645572985 |
| chi-miR-329b-3p | 3.203531758 | 8.076374906 | 4.155052578 | 0.003505846 | 1.33404459 | 0.019637806 | -0.958841149 | 0.383498308 | 0.37520344 |
| chi-miR-331-3p | 3.050982627 | 4.086261113 | 4.261592388 | 0.074081236 | 0.421507431 | 0.108830484 | 0.060611214 | 0.490437866 | 0.482118644 |
| chi-miR-331-5p | 7.932554829 | 8.412890527 | 8.827584232 | 0.065562575 | 0.084815983 | 0.083535891 | 0.069417111 | 0.162569061 | 0.154233094 |
| chi-miR-335-3p | 59.11972244 | 55.75451994 | 59.56543906 | 0.027139524 | -0.084550729 | 0.034750279 | 0.095386729 | 0.019170871 | 0.010836 |
| chi-miR-335-5p | 37.52708631 | 45.18924054 | 44.44232062 | 0.01081698 | 0.268047031 | 0.044606547 | -0.02404516 | 0.252336365 | 0.244001871 |
| chi-miR-338-5p | 0.610196525 | 1.922946406 | 2.130796194 | 0.056978612 | 1.655972684 | 0.1359945 | 0.148074055 | 1.812429628 | 1.804046739 |
| chi-miR-33a-3p | 1.525491313 | 2.403683008 | 1.826396738 | 0.086866464 | 0.655972684 | 0.125909278 | -0.396246461 | 0.268033719 | 0.259726223 |
| chi-miR-33b-3p | 0 | 0.240368301 | 0 | 0.25085307 | -2.056681446 | 0.315638314 | 2.056681446 | -- | 0 |
| chi-miR-342-3p | 308.9951996 | 192.5022314 | 161.5530933 | 0 | -0.682709256 | 0.000293028 | -0.252866795 | -0.927240993 | -0.935576051 |
| chi-miR-342-5p | 3.064247768 | 0.877866838 | 2.501369445 | 0.015024144 | -1.803458934 | 0.045246099 | 1.510644134 | -0.284498319 | -0.2928148 |
| chi-miR-3431-3p | 71.93384947 | 44.05295403 | 57.53149724 | 0 | -0.707432046 | 0.002620217 | 0.385113252 | -0.313984 | -0.322318794 |
| chi-miR-3432-5p | 62.3232542 | 73.65103252 | 88.33118768 | 0.005135124 | 0.240935185 | 0.004721475 | 0.262217159 | 0.511487482 | 0.503152344 |
| chi-miR-345-3p | 1.747380959 | 2.982752096 | 1.743378704 | 0.066648537 | 0.771449902 | 0.086520135 | -0.774758085 | 0.005015787 | -0.003308183 |
| chi-miR-34b-3p | 0.29123016 | 0 | 0.290563117 | 0.207164791 | 1.779768326 | 0.26087974 | -1.783076509 | 0.004933982 | -0.003308183 |
| chi-miR-361-3p | 92.90242098 | 98.66026163 | 83.97274092 | 0.017077066 | 0.08675292 | 0.00529697 | -0.232548034 | -0.1374603 | -0.145795114 |
| chi-miR-363-3p | 0 | 0.688327407 | 1.743378704 | 0.09860269 | -0.538833141 | 0.08996163 | 1.340719133 | 24.06710344 | 0.801885992 |
| chi-miR-369-5p | 15.5600114 | 34.61303531 | 22.52555976 | 0 | 1.153472344 | 0.001163766 | -0.619752503 | 0.542050874 | 0.533719841 |
| chi-miR-374a-3p | 194.8329769 | 152.3497994 | 202.2319297 | 3.82E-06 | -0.354850285 | 0 | 0.408623197 | 0.062108177 | 0.053772912 |
| chi-miR-376a | 0.305098263 | 1.201841504 | 0.608798913 | 0.085038376 | 1.977900779 | 0.147080773 | -0.981208962 | 1.005180239 | 0.996691817 |
| chi-miR-376b-3p | 7.755918993 | 7.43876636 | 5.383064069 | 0.070865668 | -0.06023435 | 0.065293441 | -0.466635789 | -0.518538523 | -0.526870139 |
| chi-miR-376b-5p | 3.494761918 | 3.212194565 | 3.196194291 | 0.089261599 | -0.121634894 | 0.118658179 | -0.007204171 | -0.120500171 | -0.128839065 |
| chi-miR-376c-3p | 11.59373398 | 16.82578105 | 13.39357608 | 0.012145453 | 0.537328188 | 0.040810804 | -0.329132266 | 0.216527958 | 0.208195922 |
| chi-miR-379-3p | 11.89883224 | 24.27719838 | 17.35076901 | 3.73E-05 | 1.028781948 | 0.012543577 | -0.484602336 | 0.552509918 | 0.544179612 |
| chi-miR-382-3p | 77.49495872 | 137.0099314 | 116.2805923 | 0 | 0.822106107 | 0.002306934 | -0.236670148 | 0.593771798 | 0.585435958 |
| chi-miR-409-3p | 176.0416976 | 202.8708458 | 152.1997281 | 0.000571107 | 0.204644365 | 0 | -0.414595771 | -0.201616302 | -0.209951407 |
| chi-miR-411a-3p | 17.39060097 | 33.41119381 | 21.00356248 | 7.39E-06 | 0.942023743 | 0.000761835 | -0.669697483 | 0.280654565 | 0.27232626 |
| chi-miR-411a-5p | 435.0701226 | 860.758885 | 606.3637169 | 0 | 0.984361221 | 0 | -0.505425739 | 0.487270769 | 0.478935483 |
| chi-miR-411b-3p | 0 | 2.163314707 | 0 | 0.005399256 | 1.113243556 | 0.008694126 | -1.113243556 | -- | 0 |
| chi-miR-412-5p | 29.52820577 | 56.62241102 | 35.85296205 | 0 | 0.939279485 | 1.31E-05 | -0.659280867 | 0.288334176 | 0.279998618 |
| chi-miR-425-3p | 1.456150799 | 1.835539751 | 1.162252469 | 0.111252526 | 0.33404459 | 0.125444122 | -0.659280867 | -0.316969824 | -0.325236278 |
| chi-miR-429 | 0.915294788 | 0.480736602 | 2.739595107 | 0.13744665 | -0.928989816 | 0.016057377 | 2.510644134 | 1.590060659 | 1.581654318 |
| chi-miR-449a-3p | 0.278567979 | 0.219466709 | 0.277929938 | 0.227853908 | -0.344027316 | 0.286090674 | 0.340719133 | 0.004933982 | -0.003308183 |
| chi-miR-449a-5p | 16.47530618 | 17.78725426 | 16.7419701 | 0.046930944 | 0.110538548 | 0.062126769 | -0.087374519 | 0.03150312 | 0.023164029 |
| chi-miR-451-5p | 1121.236115 | 5864.935935 | 2852.351074 | 0 | 2.387025229 | 0 | -1.039963786 | 1.355396684 | 1.347061443 |
| chi-miR-454-5p | 1.525491313 | 0.480736602 | 0 | 0.071126842 | -1.66595541 | 0.193791884 | 1.056681446 | -23.79906972 | -0.609273965 |
| chi-miR-483 | 0.873690479 | 3.441637034 | 1.452815587 | 0.010623533 | 1.977900779 | 0.043883813 | -1.244243368 | 0.742096585 | 0.733657411 |
| chi-miR-485-3p | 1.949975853 | 3.292000641 | 4.446879013 | 0.060685873 | 0.755508358 | 0.085072973 | 0.433828537 | 1.197662371 | 1.189336895 |
| chi-miR-485-5p | 2.329841278 | 3.212194565 | 2.033941822 | 0.080548559 | 0.463327607 | 0.090000021 | -0.659280867 | -0.187632151 | -0.195953261 |
| chi-miR-491-3p | 0 | 0.229442469 | 0 | 0.25085307 | -2.123795642 | 0.315638314 | 2.123795642 | -- | 0 |
| chi-miR-493-5p | 362.2903188 | 560.757394 | 487.5649109 | 0 | 0.630230484 | 0 | -0.201782437 | 0.436783519 | 0.428448047 |
| chi-miR-494 | 515.3507611 | 425.5459495 | 433.2927739 | 0 | -0.276239783 | 0.017197182 | 0.026027257 | -0.241877455 | -0.250212526 |
| chi-miR-496-3p | 0.305098263 | 0.480736602 | 0.304399456 | 0.195628342 | 0.655972684 | 0.247312928 | -0.659280867 | 0.004933982 | -0.003308183 |
| chi-miR-497-5p | 46.59682557 | 72.73326264 | 57.24093412 | 0 | 0.64238362 | 0.002116266 | -0.345568078 | 0.305151082 | 0.296815542 |
| chi-miR-502b-3p | 5.072258617 | 2.944511684 | 1.331747621 | 0.03402084 | -0.784599907 | 0.054375519 | -1.144707694 | -1.920946464 | -1.929307601 |
| chi-miR-502b-5p | 4.368452397 | 3.671079503 | 10.75083534 | 0.078663119 | -0.250917911 | 0.000260555 | 1.550172498 | 1.307603905 | 1.299254587 |
| chi-miR-504 | 0.305098263 | 3.845892812 | 0.608798913 | 0.00080425 | 3.655972684 | 0.004860417 | -2.659280867 | 1.005180239 | 0.996691817 |
| chi-miR-505-3p | 11.35797623 | 8.259928881 | 13.07534028 | 0.03045629 | -0.459504533 | 0.015942144 | 0.662647228 | 0.211473 | 0.203142695 |
| chi-miR-532-3p | 4.271375677 | 11.05694184 | 10.04518206 | 0.000513822 | 1.372179718 | 0.073243731 | -0.138448704 | 1.242066751 | 1.233731014 |
| chi-miR-542-3p | 318.1107036 | 586.7991142 | 370.7585377 | 0 | 0.883337778 | 0 | -0.662386778 | 0.229286222 | 0.220951 |
| chi-miR-542-5p | 1.392839895 | 2.853067222 | 2.501369445 | 0.051613846 | 1.034484308 | 0.121146579 | -0.189795584 | 0.85299622 | 0.844688724 |
| chi-miR-543-3p | 63.7655369 | 93.2629007 | 63.01068745 | 0 | 0.548526395 | 0 | -0.565706752 | -0.008844944 | -0.017180357 |
| chi-miR-543-5p | 0.337213869 | 3.188042726 | 1.682207522 | 0.005410559 | 3.240935185 | 0.082109228 | -0.922315273 | 2.327059086 | 2.318619912 |
| chi-miR-544-5p | 1.525491313 | 0.480736602 | 1.521997281 | 0.071126842 | -1.66595541 | 0.090463571 | 1.662647228 | 0.005032146 | -0.003308183 |
| chi-miR-545-3p | 2.621071438 | 0.458884938 | 0.871689352 | 0.010645221 | -2.513952317 | 0.172123693 | 0.925681633 | -1.579919257 | -1.588270684 |
| chi-miR-592 | 0.29123016 | 1.835539751 | 2.033941822 | 0.030308445 | 2.655972684 | 0.1359945 | 0.148074055 | 2.812429628 | 2.804046739 |
| chi-miR-660 | 315.8960881 | 373.7518061 | 372.1481874 | 0 | 0.242630479 | 0.019883063 | -0.006203342 | 0.244762507 | 0.236427138 |
| chi-miR-708-3p | 72.70624251 | 102.4909533 | 102.0002874 | 0 | 0.495345428 | 0.032500267 | -0.006923354 | 0.496758143 | 0.488422073 |
| chi-miR-93-3p | 1.392839895 | 0.438933419 | 2.223439507 | 0.071126842 | -1.66595541 | 0.02557176 | 2.340719133 | 0.683091739 | 0.674763722 |
| chi-miR-96 | 0.915294788 | 0.961473203 | 1.521997281 | 0.147179596 | 0.071010184 | 0.139046575 | 0.662647228 | 0.742096585 | 0.733657411 |
| chi-miR-98-3p | 0.58246032 | 0.229442469 | 0.290563117 | 0.158964182 | -1.344027316 | 0.286090674 | 0.340719133 | -0.995066018 | -1.003308183 |
| chi-miR-99a-3p | 1.949975853 | 7.24240141 | 5.280668829 | 0.00035091 | 1.893011882 | 0.05833991 | -0.455747473 | 1.445591505 | 1.437264409 |
| chi-miR-99b-3p | 12.49377386 | 13.88126937 | 17.25944917 | 0.051503402 | 0.151930179 | 0.043436772 | 0.314246921 | 0.474507406 | 0.4661771 |
| cin-miR-7-5p | 1254.328298 | 554.3330049 | 720.3059679 | 0 | -1.178090184 | 0 | 0.377856949 | -0.791898056 | -0.800233235 |
| cqu-miR-92 | 3.372138693 | 6.641755679 | 6.055947078 | 0.020433414 | 0.977900779 | 0.094334775 | -0.133212056 | 0.85299622 | 0.844688724 |
| crm-miR-1-3p | 12.17342068 | 13.37649594 | 21.41450175 | 0.052885029 | 0.135965626 | 0.00497526 | 0.678887869 | 0.823186699 | 0.814853494 |
| dre-miR-107a-3p | 50.42080419 | 67.37627978 | 60.8666565 | 0.000295349 | 0.418221643 | 0.022878849 | -0.146588653 | 0.279970103 | 0.27163299 |
| dre-miR-1306 | 2.621071438 | 3.212194565 | 3.196194291 | 0.087409043 | 0.293402605 | 0.118658179 | -0.007204171 | 0.294564785 | 0.286198434 |
| dre-miR-1388-5p | 0.29123016 | 1.376654813 | 0.581126235 | 0.062662052 | 2.240935185 | 0.121908424 | -1.244243368 | 1.005180239 | 0.996691817 |
| dre-miR-140-3p | 936.2669773 | 733.4577428 | 1041.959339 | 0 | -0.352206125 | 0 | 0.506513225 | 0.162642363 | 0.1543071 |
| dre-miR-17a-5p | 188.1346832 | 137.6654813 | 122.9081986 | 0 | -0.45059898 | 0.008145 | -0.163585705 | -0.605849062 | -0.614184684 |
| dre-miR-181b-3p | 0 | 0 | 0.290563117 | -- | 0 | 0.26087974 | -1.783076509 | 21.48197676 | -1.783076509 |
| dre-miR-194a | 11.28863572 | 17.06614935 | 17.35076901 | 0.008521797 | 0.596266438 | 0.064889463 | 0.023862027 | 0.628454661 | 0.620128466 |
| dre-miR-199-3p | 2671.745486 | 6699.031765 | 4986.64422 | 0 | 1.326170011 | 0 | -0.425883317 | 0.908621978 | 0.900286694 |
| dre-miR-23b | 258.3211518 | 386.6105601 | 415.7958209 | 0 | 0.581715266 | 0.003846072 | 0.104994213 | 0.695044598 | 0.68670948 |
| dre-miR-27b-5p | 24.51398215 | 8.120268247 | 15.84200649 | 0 | -1.594005569 | 0.001148341 | 0.964155781 | -0.621516458 | -0.629849787 |
| dre-miR-29b | 4.077222237 | 1.835539751 | 2.615068056 | 0.027160268 | -1.151382238 | 0.111356788 | 0.510644134 | -0.632402367 | -0.640738103 |
| dre-miR-301c-5p | 10.37334093 | 1.922946406 | 4.8703913 | 0 | -2.431490157 | 0.020057113 | 1.340719133 | -1.082447624 | -1.090771024 |
| dre-miR-30c-5p | 117.6569846 | 118.8511989 | 99.95371237 | 0.021606018 | 0.014569489 | 0.002363605 | -0.2498244 | -0.226919853 | -0.235254911 |
| dre-miR-30e-5p | 294.1424614 | 238.1612827 | 226.9297947 | 0 | -0.304576165 | 0.015992044 | -0.069692857 | -0.365933487 | -0.374269022 |
| dvi-miR-125-5p | 297.7759044 | 891.5260275 | 566.7917876 | 0 | 1.582049899 | 0 | -0.653458062 | 0.936927002 | 0.928591837 |
| eca-miR-125a-3p | 0.873690479 | 1.835539751 | 0.871689352 | 0.081065193 | 1.071010184 | 0.104224791 | -1.074318367 | 0.005098158 | -0.003308183 |
| eca-miR-1307 | 62.78922246 | 47.2467932 | 53.44036854 | 0.000473487 | -0.410300535 | 0.022194503 | 0.177713545 | -0.224252146 | -0.23258699 |
| eca-miR-139-3p | 0.873690479 | 1.147212345 | 2.033941822 | 0.134856116 | 0.392938279 | 0.108711 | 0.82614596 | 1.227467127 | 1.219084239 |
| eca-miR-1468 | 60.40945601 | 43.50666244 | 63.31508691 | 0.000410196 | -0.473538049 | 0.000136621 | 0.541312964 | 0.076110319 | 0.067774915 |
| eca-miR-146b-3p | 0.29123016 | 0.458884938 | 0 | 0.195628342 | 0.655972684 | 0.193791884 | 1.123795642 | -21.47704278 | 1.779768326 |
| eca-miR-154b | 1.114271916 | 1.755733675 | 2.779299383 | 0.09921642 | 0.655972684 | 0.094435052 | 0.662647228 | 1.326886281 | 1.318619912 |
| eca-miR-182 | 3.587955569 | 1.211456236 | 4.602519779 | 0.01211819 | -1.566419737 | 0.003237778 | 1.925681633 | 0.367597633 | 0.359261897 |
| eca-miR-1912 | 0.29123016 | 1.147212345 | 3.777320526 | 0.085038376 | 1.977900779 | 0.016344202 | 1.719230756 | 3.705525248 | 3.697131535 |
| eca-miR-195 | 233.0950727 | 315.3632106 | 300.7466628 | 0 | 0.436095861 | 0.013335017 | -0.06846564 | 0.375965492 | 0.367630221 |
| eca-miR-224 | 436.9007121 | 320.8916815 | 287.3530867 | 0 | -0.445219066 | 0.001167965 | -0.159261844 | -0.596145814 | -0.604480911 |
| eca-miR-302d | 0 | 0 | 0.277929938 | -- | 0 | 0.26087974 | -1.847206846 | 21.48197676 | -1.847206846 |
| eca-miR-345-3p | 1.747380959 | 2.982752096 | 1.743378704 | 0.066648537 | 0.771449902 | 0.086520135 | -0.774758085 | 0.005015787 | -0.003308183 |
| eca-miR-362-3p | 0.58246032 | 2.753309627 | 1.743378704 | 0.014657971 | 2.240935185 | 0.099764444 | -0.659280867 | 1.590060659 | 1.581654318 |
| eca-miR-376b | 7.017260041 | 6.730312421 | 4.8703913 | 0.070865668 | -0.06023435 | 0.065293441 | -0.466635789 | -0.518538523 | -0.526870139 |
| eca-miR-378 | 456.1219027 | 354.7836119 | 354.6253666 | 0 | -0.362480079 | 0.020970375 | -0.000643634 | -0.354788513 | -0.363123712 |
| eca-miR-3959 | 37.32810918 | 87.12828363 | 56.41977748 | 0 | 1.222878691 | 0 | -0.626940147 | 0.604274354 | 0.595938544 |
| eca-miR-411 | 435.0701226 | 860.758885 | 606.3637169 | 0 | 0.984361221 | 0 | -0.505425739 | 0.487270769 | 0.478935483 |
| eca-miR-421 | 0.305098263 | 0 | 0 | 0.207164791 | 1.71265413 | -- | 0 | -21.47704278 | 1.71265413 |
| eca-miR-424 | 123.7728179 | 265.2354941 | 138.8891701 | 0 | 1.099579336 | 0 | -0.933339742 | 0.174575288 | 0.166239594 |
| eca-miR-485-3p | 2.038611119 | 3.441637034 | 4.649009878 | 0.060685873 | 0.755508358 | 0.085072973 | 0.433828537 | 1.197662371 | 1.189336895 |
| eca-miR-503 | 91.3702971 | 110.6112215 | 65.86939539 | 0.000662944 | 0.275700603 | 0 | -0.747817542 | -0.463780275 | -0.472116939 |
| eca-miR-532-3p | 4.077222237 | 10.55435357 | 9.588582873 | 0.000513822 | 1.372179718 | 0.073243731 | -0.138448704 | 1.242066751 | 1.233731014 |
| efu-let-7d | 89.69888922 | 115.2566002 | 94.55408111 | 3.45E-05 | 0.361687345 | 0.000846 | -0.285637736 | 0.084384632 | 0.076049609 |
| efu-let-7e | 179.1308175 | 302.864059 | 283.9285928 | 0 | 0.757656825 | 0.007076825 | -0.093142241 | 0.672850074 | 0.664514584 |
| efu-miR-103b | 291.1369662 | 346.6783928 | 293.5384837 | 0 | 0.251899889 | 0 | -0.240048265 | 0.020187145 | 0.011851624 |
| efu-miR-106 | 57.4072891 | 73.49501164 | 66.73653679 | 0.00045506 | 0.356412403 | 0.02096209 | -0.139169511 | 0.22557799 | 0.217242892 |
| efu-miR-107 | 46.89970494 | 62.18808677 | 56.25301952 | 0.000363431 | 0.407059387 | 0.023103839 | -0.144707694 | 0.270686145 | 0.262351692 |
| efu-miR-125a | 75.42861139 | 127.3405702 | 126.3949561 | 0 | 0.755508358 | 0.030163318 | -0.010753238 | 0.753090437 | 0.74475512 |
| efu-miR-125b | 268.5627457 | 804.9033011 | 511.124737 | 0 | 1.58355628 | 0 | -0.655140057 | 0.936751312 | 0.928416224 |
| efu-miR-126 | 2214.894001 | 2821.244549 | 2927.9919 | 0 | 0.349094069 | 0.000346921 | 0.053579837 | 0.411009162 | 0.402673906 |
| efu-miR-128a | 42.22837317 | 74.5688024 | 34.57701097 | 0 | 0.820359502 | 0 | -1.108759012 | -0.280066776 | -0.28839951 |
| efu-miR-128b | 45.96371653 | 40.16240782 | 29.46057346 | 0.018489718 | -0.194649692 | 0.003321037 | -0.447060251 | -0.633375387 | -0.641709942 |
| efu-miR-133-3p | 3.737453718 | 3.785800737 | 5.060640961 | 0.084677256 | 0.018542764 | 0.077461386 | 0.418721645 | 0.445591505 | 0.437264409 |
| efu-miR-133-5p | 0 | 0.219466709 | 0.833789815 | 0.25085307 | -2.187925979 | 0.132213553 | 1.925681633 | 23.06710344 | -0.262244346 |
| efu-miR-143 | 3146.669069 | 5396.448628 | 5118.705157 | 0 | 0.77818483 | 0 | -0.076231382 | 0.710288745 | 0.701953447 |
| efu-miR-145 | 344.4437346 | 288.5284935 | 299.4194812 | 0 | -0.255554537 | 0.015660796 | 0.053454292 | -0.193764961 | -0.202100245 |
| efu-miR-155 | 208.3577055 | 194.2368165 | 240.0981151 | 0.007984843 | -0.101245774 | 0 | 0.305807397 | 0.212896946 | 0.204561623 |
| efu-miR-16 | 120.8985029 | 150.5541626 | 158.9759247 | 3.73E-05 | 0.316486218 | 0.020082663 | 0.078525703 | 0.403347973 | 0.395011922 |
| efu-miR-181a | 39.27808503 | 19.31307043 | 40.29984106 | 0 | -1.024147049 | 0 | 1.061196604 | 0.045384551 | 0.037049555 |
| efu-miR-181d | 14.94981487 | 23.976738 | 20.77526289 | 0.000858726 | 0.681507777 | 0.039724959 | -0.206768663 | 0.483081165 | 0.474739114 |
| efu-miR-181e | 95.08082258 | 71.27400854 | 102.2782173 | 3.73E-05 | -0.415778319 | 0 | 0.521050949 | 0.113607654 | 0.10527263 |
| efu-miR-181f | 19.80365087 | 16.9787427 | 21.50167068 | 0.03586941 | -0.222036791 | 0.028624922 | 0.340719133 | 0.12701822 | 0.118682342 |
| efu-miR-185 | 202.7974887 | 353.3414021 | 210.6708933 | 0 | 0.801023017 | 0 | -0.746071802 | 0.063286344 | 0.054951215 |
| efu-miR-199 | 2555.582639 | 6407.769514 | 4769.833602 | 0 | 1.326170011 | 0 | -0.425883317 | 0.908621978 | 0.900286694 |
| efu-miR-20 | 592.7815165 | 355.7643146 | 342.8877235 | 0 | -0.736578657 | 0.013048105 | -0.053185554 | -0.781428924 | -0.789764212 |
| efu-miR-200a | 2.402648818 | 0.841289053 | 6.392388582 | 0.035314737 | -1.513952317 | 1.86E-05 | 2.925681633 | 1.420080743 | 1.411729316 |
| efu-miR-200b | 9.610595274 | 2.734189421 | 24.50415623 | 3.45E-05 | -1.813512599 | 0 | 3.163841371 | 1.358668254 | 1.350328772 |
| efu-miR-205 | 4.457087663 | 2.633600513 | 12.22891729 | 0.04398532 | -0.759064815 | 0 | 2.215188251 | 1.464471729 | 1.456123436 |
| efu-miR-214 | 270.2109396 | 418.3035481 | 386.3226143 | 0 | 0.630464151 | 0.00211948 | -0.114744104 | 0.524055425 | 0.515720048 |
| efu-miR-218a | 91.74914955 | 83.99429902 | 124.7794251 | 0.01407958 | -0.127403375 | 0 | 0.571016752 | 0.451948534 | 0.443613377 |
| efu-miR-221 | 11.78899687 | 7.874465533 | 12.27338608 | 0.014834373 | -0.582187053 | 0.015552345 | 0.640279415 | 0.066423494 | 0.058092362 |
| efu-miR-223 | 0.835703937 | 1.755733675 | 1.66757963 | 0.081065193 | 1.071010184 | 0.141457698 | -0.074318367 | 1.005098158 | 0.996691817 |
| efu-miR-23b | 265.7649946 | 379.7915299 | 409.3685648 | 0 | 0.515056639 | 0.002641374 | 0.108192592 | 0.631584709 | 0.623249231 |
| efu-miR-25 | 154.0364854 | 194.1274489 | 159.2770155 | 0 | 0.333732013 | 4.59E-05 | -0.285466031 | 0.056600824 | 0.048265983 |
| efu-miR-26b | 403.3780405 | 555.461097 | 789.4599899 | 0 | 0.461553325 | 0 | 0.507180279 | 0.977068692 | 0.968733604 |
| efu-miR-28 | 27.57822992 | 45.42960884 | 46.13636976 | 1.09E-05 | 0.720103022 | 0.044068038 | 0.022271607 | 0.750708064 | 0.742374628 |
| efu-miR-299 | 2.135687839 | 4.326629414 | 3.348394019 | 0.034181107 | 1.018542764 | 0.096049901 | -0.36977425 | 0.657119175 | 0.648768514 |
| efu-miR-29a | 46.71817147 | 53.21153258 | 45.27941912 | 0.013536059 | 0.187755147 | 0.015483172 | -0.232883506 | -0.036791583 | -0.045128359 |
| efu-miR-30b | 105.3321242 | 105.8005113 | 89.2377446 | 0.021768352 | 0.006401102 | 0.002440523 | -0.245620643 | -0.230883992 | -0.23921954 |
| efu-miR-30c | 0.835703937 | 1.316800256 | 1.111719753 | 0.115981432 | 0.655972684 | 0.156876716 | -0.244243368 | 0.420094615 | 0.411729316 |
| efu-miR-30d | 0.557135958 | 0.877866838 | 0.833789815 | 0.143516961 | 0.655972684 | 0.185895435 | -0.074318367 | 0.590060659 | 0.581654318 |
| efu-miR-30e | 28.13536587 | 29.84747248 | 31.96194291 | 0.035820767 | 0.085224043 | 0.044992652 | 0.098746342 | 0.192306181 | 0.183970385 |
| efu-miR-320 | 134.2920513 | 115.6940705 | 87.70357134 | 0.001748044 | -0.215058988 | 1.86E-05 | -0.39960743 | -0.60633076 | -0.614666418 |
| efu-miR-323 | 15.59980682 | 27.21387196 | 22.51232501 | 0.000133263 | 0.802814073 | 0.028126212 | -0.273627175 | 0.537528715 | 0.529186898 |
| efu-miR-331 | 2.912301598 | 3.900521971 | 4.067883643 | 0.074081236 | 0.421507431 | 0.108830484 | 0.060611214 | 0.490437866 | 0.482118644 |
| efu-miR-34a | 18.15334663 | 35.54446248 | 21.84066099 | 0 | 0.969389279 | 0.000151976 | -0.702608299 | 0.275118645 | 0.266780981 |
| efu-miR-379 | 384.936376 | 730.1042915 | 570.7124526 | 0 | 0.923482549 | 0 | -0.355338518 | 0.576479317 | 0.568144031 |
| efu-miR-381 | 117.5556871 | 108.4165544 | 107.2809562 | 0.013343071 | -0.116759273 | 0.031675006 | -0.015191062 | -0.12361597 | -0.131950334 |
| efu-miR-409 | 154.0364854 | 177.5119901 | 133.1747621 | 0.000571107 | 0.204644365 | 0 | -0.414595771 | -0.201616302 | -0.209951407 |
| efu-miR-423 | 231.1882085 | 213.6874194 | 191.5053079 | 0.005773516 | -0.113565843 | 0.002807397 | -0.158117594 | -0.263347634 | -0.271683437 |
| efu-miR-452 | 17.47380959 | 17.43762764 | 13.94702963 | 0.049779087 | -0.002990398 | 0.038742238 | -0.32224588 | -0.316904071 | -0.325236278 |
| efu-miR-487 | 1.164920639 | 1.376654813 | 0 | 0.125796769 | 0.240935185 | 0.035004543 | -0.461166859 | -23.47716634 | -0.220231674 |
| efu-miR-493 | 318.8154805 | 493.4665067 | 429.0571216 | 0 | 0.630230484 | 0 | -0.201782437 | 0.436783519 | 0.428448047 |
| efu-miR-495 | 96.10595274 | 163.2832318 | 117.008504 | 0 | 0.764678944 | 0 | -0.480763255 | 0.292250656 | 0.283915689 |
| efu-miR-497 | 44.57087663 | 69.57094688 | 54.75219785 | 0 | 0.64238362 | 0.002116266 | -0.345568078 | 0.305151082 | 0.296815542 |
| efu-miR-500 | 26.96305896 | 35.54446248 | 35.1581372 | 0.004006575 | 0.398640638 | 0.046464923 | -0.015766184 | 0.391211125 | 0.382874454 |
| efu-miR-503 | 6.140102536 | 9.464501842 | 4.527941912 | 0.019031155 | 0.624263825 | 0.005014959 | -1.063671122 | -0.431055765 | -0.439407298 |
| efu-miR-532 | 239.8470299 | 310.7648605 | 343.2434739 | 0 | 0.373708807 | 0.001490825 | 0.143408909 | 0.525452758 | 0.517117716 |
| efu-miR-628 | 0.26696098 | 1.261933579 | 0.799048573 | 0.062662052 | 2.240935185 | 0.145893296 | -0.659280867 | 1.590060659 | 1.581654318 |
| efu-miR-7a | 1110.215966 | 492.4569599 | 639.2388582 | 0 | -1.1727708 | 0 | 0.376357469 | -0.788077895 | -0.796413331 |
| efu-miR-7c | 1118.417007 | 491.2455036 | 636.6819028 | 0 | -1.186942097 | 0 | 0.374128555 | -0.804478245 | -0.812813542 |
| efu-miR-874 | 3.737453718 | 5.678701105 | 5.060640961 | 0.041573862 | 0.603505265 | 0.090237063 | -0.166240856 | 0.445591505 | 0.437264409 |
| fru-miR-7 | 1314.058217 | 580.7298146 | 754.6062521 | 0 | -1.178090184 | 0 | 0.377856949 | -0.791898056 | -0.800233235 |
| gga-let-7g-5p | 11608.98889 | 13433.94396 | 12064.26365 | 0 | 0.210640592 | 0 | -0.155143053 | 0.063832833 | 0.055497539 |
| gga-miR-101-3p | 111.8323814 | 185.6189573 | 153.1267628 | 0 | 0.731006076 | 0.000149902 | -0.277617608 | 0.461723275 | 0.453388468 |
| gga-miR-103-2-5p | 0 | 0.219466709 | 0 | 0.25085307 | -2.187925979 | 0.315638314 | 2.187925979 | -- | 0 |
| gga-miR-10b-3p | 0.610196525 | 2.163314707 | 1.521997281 | 0.042078378 | 1.825897686 | 0.126492745 | -0.507277774 | 1.327059086 | 1.318619912 |
| gga-miR-125b-3p | 16.89134927 | 48.64180341 | 35.44870032 | 0 | 1.525912144 | 0.001792683 | -0.456463984 | 1.077783192 | 1.06944816 |
| gga-miR-126-3p | 2214.894001 | 2821.025083 | 2927.9919 | 0 | 0.348981837 | 0.000346091 | 0.053692069 | 0.411009162 | 0.402673906 |
| gga-miR-1306-3p | 1.671407874 | 2.194667094 | 1.389649692 | 0.098271658 | 0.392938279 | 0.112092073 | -0.659280867 | -0.257985786 | -0.266342589 |
| gga-miR-140-3p | 1024.825064 | 802.8301245 | 1140.584763 | 0 | -0.352211008 | 0 | 0.506607008 | 0.162731307 | 0.154395999 |
| gga-miR-140-5p | 358.7955569 | 204.0726873 | 239.8667715 | 0 | -0.814078917 | 0.00028678 | 0.233150209 | -0.572593421 | -0.580928708 |
| gga-miR-221-5p | 10.91573784 | 7.29117179 | 11.36424637 | 0.014834373 | -0.582187053 | 0.015552345 | 0.640279415 | 0.066423494 | 0.058092362 |
| gga-miR-26a-5p | 3501.307662 | 3790.608103 | 4441.492467 | 0 | 0.114535471 | 0 | 0.228615235 | 0.351485972 | 0.343150706 |
| gga-miR-29c-3p | 0.320353176 | 0.504773432 | 0.319619429 | 0.195628342 | 0.655972684 | 0.247312928 | -0.659280867 | 0.004933982 | -0.003308183 |
| gga-miR-30c-2-3p | 0.610196525 | 2.403683008 | 2.43519565 | 0.030415169 | 1.977900779 | 0.129388308 | 0.018791038 | 2.005118679 | 1.996691817 |
| gga-miR-30e-3p | 30.50982627 | 32.4497206 | 35.00593747 | 0.03585998 | 0.088932092 | 0.043977223 | 0.109393587 | 0.20666074 | 0.198325678 |
| gga-miR-30e-5p | 335.8650138 | 273.3746637 | 260.4057243 | 0 | -0.297001981 | 0.016003647 | -0.070118378 | -0.358785268 | -0.367120359 |
| gga-miR-34a-5p | 18.94262257 | 37.08987389 | 22.79025494 | 0 | 0.969389279 | 0.000151976 | -0.702608299 | 0.275118645 | 0.266780981 |
| gga-miR-365-1-5p | 24.79255013 | 8.998135085 | 15.28614661 | 0 | -1.462208742 | 0.005264355 | 0.764526842 | -0.689341781 | -0.6976819 |
| gga-miR-365-2-5p | 4.457087663 | 1.097333547 | 3.057229322 | 0.002624756 | -2.022099221 | 0.034434148 | 1.478222656 | -0.535517078 | -0.543876564 |
| gga-miR-92-3p | 727.0491599 | 801.1475464 | 666.0260103 | 7.39E-06 | 0.14001505 | 0 | -0.266489447 | -0.11813921 | -0.126474396 |
| gga-miR-99a-3p | 2.135687839 | 7.932153925 | 5.783589669 | 0.00035091 | 1.893011882 | 0.05833991 | -0.455747473 | 1.445591505 | 1.437264409 |
| ggo-let-7c | 1422.368101 | 4350.425875 | 2657.711653 | 0 | 1.612861763 | 0 | -0.710972049 | 0.910225 | 0.901889714 |
| ggo-let-7f | 8030.186273 | 9478.923941 | 8755.137162 | 0 | 0.239289838 | 0 | -0.114593511 | 0.133031618 | 0.124696327 |
| ggo-miR-125a | 227.6033039 | 336.7559894 | 259.9571357 | 0 | 0.565182105 | 0 | -0.373429848 | 0.200088049 | 0.191752257 |
| ggo-miR-127 | 749.6264314 | 1298.469561 | 1177.417097 | 0 | 0.792568467 | 0 | -0.141186712 | 0.659717064 | 0.651381754 |
| ggo-miR-128 | 52.85827401 | 46.18676899 | 33.87965948 | 0.018489718 | -0.194649692 | 0.003321037 | -0.447060251 | -0.633375387 | -0.641709942 |
| ggo-miR-1298 | 23.70613501 | 110.5453815 | 324.4137205 | 0 | 2.221306378 | 0 | 1.553196085 | 3.782838573 | 3.774502464 |
| ggo-miR-130b | 1.830589576 | 3.365156211 | 3.652793475 | 0.056636429 | 0.878365106 | 0.113959284 | 0.118326711 | 1.005015787 | 0.996691817 |
| ggo-miR-134 | 23.49256622 | 34.85340361 | 25.26515487 | 0.000991945 | 0.569095234 | 0.005637229 | -0.464150526 | 0.113276004 | 0.104944708 |
| ggo-miR-145 | 358.7955569 | 300.5505141 | 311.8952929 | 0 | -0.255554537 | 0.015660796 | 0.053454292 | -0.193764961 | -0.202100245 |
| ggo-miR-146a | 71.69809173 | 101.4354229 | 92.84183417 | 3.82E-06 | 0.500554927 | 0.020028023 | -0.127714624 | 0.381176803 | 0.372840303 |
| ggo-miR-148a | 230473.0582 | 187820.1847 | 214366.3159 | 0 | -0.295245998 | 0 | 0.190726113 | -0.096184597 | -0.104519885 |
| ggo-miR-151a | 32.99637711 | 61.83474537 | 50.81948923 | 0 | 0.906110096 | 0.009864125 | -0.283035851 | 0.63140691 | 0.623074245 |
| ggo-miR-191 | 279.1649103 | 329.304572 | 299.529065 | 7.39E-06 | 0.238304929 | 0.002371997 | -0.13672654 | 0.109913694 | 0.101578389 |
| ggo-miR-193a | 0.915294788 | 0.480736602 | 0.608798913 | 0.13744665 | -0.928989816 | 0.221716937 | 0.340719133 | -0.579782262 | -0.588270684 |
| ggo-miR-26b | 461.0034749 | 634.8126823 | 902.2399884 | 0 | 0.461553325 | 0 | 0.507180279 | 0.977068692 | 0.968733604 |
| ggo-miR-323a | 17.93977784 | 31.29595276 | 25.88917376 | 0.000133263 | 0.802814073 | 0.028126212 | -0.273627175 | 0.537528715 | 0.529186898 |
| ggo-miR-328 | 28.06904016 | 52.15992127 | 36.2235353 | 0 | 0.893961961 | 0.000557557 | -0.526014336 | 0.376280136 | 0.367947624 |
| ggo-miR-335 | 35.82130966 | 43.13518415 | 42.42221513 | 0.01081698 | 0.268047031 | 0.044606547 | -0.02404516 | 0.252336365 | 0.244001871 |
| ggo-miR-365a | 70.78279694 | 42.54518923 | 49.00831246 | 0 | -0.734402761 | 0.024218927 | 0.204030461 | -0.522036441 | -0.5303723 |
| ggo-miR-370 | 40.11378897 | 47.62427594 | 34.18538242 | 0.010204528 | 0.247598915 | 0.001171071 | -0.478317594 | -0.222383841 | -0.230718679 |
| ggo-miR-376b | 7.368123043 | 7.066828042 | 5.113910866 | 0.070865668 | -0.06023435 | 0.065293441 | -0.466635789 | -0.518538523 | -0.526870139 |
| ggo-miR-376c | 12.17342068 | 17.66707011 | 14.06325488 | 0.012145453 | 0.537328188 | 0.040810804 | -0.329132266 | 0.216527958 | 0.208195922 |
| ggo-miR-378a | 416.4591285 | 323.9328631 | 323.7883782 | 0 | -0.362480079 | 0.020970375 | -0.000643634 | -0.354788513 | -0.363123712 |
| ggo-miR-381 | 128.7514668 | 118.7419406 | 117.4981901 | 0.013343071 | -0.116759273 | 0.031675006 | -0.015191062 | -0.12361597 | -0.131950334 |
| ggo-miR-409 | 62.03202404 | 80.30486412 | 59.27487594 | 0.000394132 | 0.372474176 | 0.000204992 | -0.438066637 | -0.057257956 | -0.065592461 |
| ggo-miR-410 | 14.09553973 | 17.41468339 | 13.10439659 | 0.032065958 | 0.305065523 | 0.03168584 | -0.410253319 | -0.096856079 | -0.105187797 |
| ggo-miR-423 | 252.2053184 | 233.1135484 | 208.9148814 | 0.005773516 | -0.113565843 | 0.002807397 | -0.158117594 | -0.263347634 | -0.271683437 |
| ggo-miR-431 | 6.712161779 | 6.489944121 | 5.479190213 | 0.071770483 | -0.048571432 | 0.086808664 | -0.244243368 | -0.284475854 | -0.2928148 |
| ggo-miR-432 | 15.72642863 | 27.3036538 | 20.33941822 | 0.000190212 | 0.795902946 | 0.01311018 | -0.424815614 | 0.379425144 | 0.371087332 |
| ggo-miR-433 | 24.10276275 | 42.30482093 | 28.00474998 | 7.39E-06 | 0.811623555 | 0.00058467 | -0.59515053 | 0.224806078 | 0.216473025 |
| ggo-miR-454 | 25.62825406 | 16.19481426 | 19.44351527 | 0.000905646 | -0.662203276 | 0.039154871 | 0.263757151 | -0.390107361 | -0.398446125 |
| ggo-miR-485 | 2.562825406 | 3.533414021 | 2.237336004 | 0.080548559 | 0.463327607 | 0.090000021 | -0.659280867 | -0.187632151 | -0.195953261 |
| ggo-miR-487b | 8.542751354 | 16.34504445 | 11.56717934 | 0.001044151 | 0.936080604 | 0.022936857 | -0.498816195 | 0.445604465 | 0.437264409 |
| ggo-miR-491 | 1.114271916 | 3.292000641 | 1.66757963 | 0.019979853 | 1.56286328 | 0.06061857 | -0.981208962 | 0.589937103 | 0.581654318 |
| ggo-miR-493 | 378.9320422 | 586.7390222 | 510.7822876 | 0 | 0.630779806 | 0 | -0.200010447 | 0.439104712 | 0.430769359 |
| ggo-miR-495 | 107.6996867 | 185.0835916 | 136.6753559 | 0 | 0.781162947 | 0 | -0.437423868 | 0.352074871 | 0.343739079 |
| ggo-miR-497 | 51.25650813 | 80.00658891 | 62.96502753 | 0 | 0.64238362 | 0.002116266 | -0.345568078 | 0.305151082 | 0.296815542 |
| ggo-miR-499a | 2.562825406 | 3.02864059 | 1.917716575 | 0.096057344 | 0.240935185 | 0.099764444 | -0.659280867 | -0.41000112 | -0.418345682 |
| ggo-miR-502b | 28.13536587 | 37.08987389 | 36.68675186 | 0.004006575 | 0.398640638 | 0.046464923 | -0.015766184 | 0.391211125 | 0.382874454 |
| ggo-miR-532 | 261.7743094 | 339.640409 | 375.0201301 | 0 | 0.375684597 | 0.001518834 | 0.142959923 | 0.526979885 | 0.51864452 |
| ggo-miR-582 | 0.58246032 | 1.606097282 | 0.290563117 | 0.073694749 | 1.463327607 | 0.057483806 | -2.466635789 | -0.995066018 | -1.003308183 |
| ggo-miR-656 | 26.90966677 | 28.01492545 | 27.80689033 | 0.04156198 | 0.058071128 | 0.055152492 | -0.010753238 | 0.055653404 | 0.04731789 |
| ggo-miR-760 | 1.525491313 | 1.442209805 | 0.608798913 | 0.12274424 | -0.08099291 | 0.121908424 | -1.244243368 | -1.316846701 | -1.325236278 |
| ggo-miR-96 | 0.873690479 | 0.917769876 | 1.452815587 | 0.147179596 | 0.071010184 | 0.139046575 | 0.662647228 | 0.742096585 | 0.733657411 |
| hhi-miR-183 | 0.800882939 | 1.051611316 | 3.462543815 | 0.134856116 | 0.392938279 | 0.016344202 | 1.719230756 | 2.120562747 | 2.112169035 |
| hhi-miR-301 | 0.53392196 | 0.630966789 | 0 | 0.163565675 | 0.240935185 | 0.12613315 | 0.664364023 | -22.47704278 | 0.905299208 |
| hsa-let-7f-2-3p | 1.456150799 | 2.523867158 | 2.324504939 | 0.074343502 | 0.793476208 | 0.126273985 | -0.118712486 | 0.683091739 | 0.674763722 |
| hsa-miR-106a-5p | 0.557135958 | 0.219466709 | 0.555859877 | 0.158964182 | -1.344027316 | 0.200950336 | 1.340719133 | 0.005180239 | -0.003308183 |
| hsa-miR-1260a | 0.711895946 | 3.365156211 | 2.130796194 | 0.014657971 | 2.240935185 | 0.099764444 | -0.659280867 | 1.590060659 | 1.581654318 |
| hsa-miR-127-5p | 23.8808731 | 47.953476 | 37.19207902 | 0 | 1.005779812 | 0.005564197 | -0.366639999 | 0.647478957 | 0.639139813 |
| hsa-miR-1307-5p | 0.915294788 | 0.961473203 | 0.913198369 | 0.147179596 | 0.071010184 | 0.185895435 | -0.074318367 | 0.005098158 | -0.003308183 |
| hsa-miR-130b-5p | 27.15374538 | 18.50835916 | 19.78596466 | 0.003599652 | -0.552974206 | 0.058971176 | 0.096300405 | -0.448337125 | -0.456673801 |
| hsa-miR-136-3p | 76.59353203 | 181.9478778 | 118.840315 | 0 | 1.248230751 | 0 | -0.61450089 | 0.642064245 | 0.633729861 |
| hsa-miR-143-5p | 25.91948422 | 40.84075947 | 38.35433149 | 9.96E-05 | 0.655972684 | 0.042305166 | -0.090620179 | 0.573690387 | 0.565352506 |
| hsa-miR-151b | 2.135687839 | 5.047734316 | 4.971857786 | 0.022905327 | 1.240935185 | 0.10838445 | -0.021850947 | 1.227419934 | 1.219084239 |
| hsa-miR-15b-3p | 5.533373036 | 4.818291847 | 5.230136112 | 0.072748996 | -0.199637406 | 0.097969644 | 0.118326711 | -0.07298239 | -0.081310695 |
| hsa-miR-16-1-3p | 1.164920639 | 0.688327407 | 1.162252469 | 0.121667271 | -0.759064815 | 0.151642571 | 0.755756632 | 0.004933561 | -0.003308183 |
| hsa-miR-181b-2-3p | 0 | 0 | 0.319619429 | -- | 0 | 0.26087974 | -1.645572985 | 21.48197676 | -1.645572985 |
| hsa-miR-181b-3p | 0.915294788 | 1.682578105 | 2.130796194 | 0.098534335 | 0.878365106 | 0.131350463 | 0.340719133 | 1.227467127 | 1.219084239 |
| hsa-miR-181c-3p | 0.29123016 | 0.917769876 | 1.452815587 | 0.114580875 | 1.655972684 | 0.139046575 | 0.662647228 | 2.327059086 | 2.318619912 |
| hsa-miR-1911-5p | 0.278567979 | 1.316800256 | 1.945509568 | 0.062662052 | 2.240935185 | 0.124855427 | 0.563111554 | 2.812429628 | 2.804046739 |
| hsa-miR-194-3p | 0.873690479 | 0.458884938 | 0.581126235 | 0.13744665 | -0.928989816 | 0.221716937 | 0.340719133 | -0.579782262 | -0.588270684 |
| hsa-miR-195-3p | 51.54773829 | 35.79302515 | 41.25996266 | 0.000295349 | -0.526230647 | 0.027499429 | 0.205064033 | -0.312833351 | -0.321166613 |
| hsa-miR-221-5p | 13.39658735 | 8.948256287 | 13.94702963 | 0.014834373 | -0.582187053 | 0.015552345 | 0.640279415 | 0.066423494 | 0.058092362 |
| hsa-miR-223-5p | 0.29123016 | 0 | 0.581126235 | 0.207164791 | 1.779768326 | 0.13358663 | -0.783076509 | 1.005180239 | 0.996691817 |
| hsa-miR-24-1-5p | 4.368452397 | 5.736061723 | 7.264077934 | 0.060690946 | 0.392938279 | 0.071140764 | 0.340719133 | 0.742010988 | 0.733657411 |
| hsa-miR-24-2-5p | 26.50194454 | 19.50260986 | 21.50167068 | 0.008341234 | -0.44243102 | 0.051893476 | 0.140781562 | -0.293316933 | -0.301649457 |
| hsa-miR-25-5p | 0.610196525 | 0.480736602 | 0.608798913 | 0.174900687 | -0.344027316 | 0.221716937 | 0.340719133 | 0.005180239 | -0.003308183 |
| hsa-miR-29a-5p | 0.29123016 | 0.229442469 | 0.290563117 | 0.227853908 | -0.344027316 | 0.286090674 | 0.340719133 | 0.004933982 | -0.003308183 |
| hsa-miR-29c-5p | 2.621071438 | 2.753309627 | 2.615068056 | 0.098568343 | 0.071010184 | 0.125959855 | -0.074318367 | 0.005043243 | -0.003308183 |
| hsa-miR-3064-5p | 0.305098263 | 0.240368301 | 0.304399456 | 0.227853908 | -0.344027316 | 0.286090674 | 0.340719133 | 0.004933982 | -0.003308183 |
| hsa-miR-30c-1-3p | 0.873690479 | 1.376654813 | 1.162252469 | 0.115981432 | 0.655972684 | 0.156876716 | -0.244243368 | 0.420094615 | 0.411729316 |
| hsa-miR-324-3p | 0 | 0.252386716 | 0 | 0.25085307 | -1.986292118 | 0.315638314 | 1.986292118 | -- | 0 |
| hsa-miR-331-5p | 7.863214315 | 8.030486412 | 8.426330403 | 0.066188432 | 0.030368199 | 0.083535891 | 0.069417111 | 0.108124093 | 0.09978531 |
| hsa-miR-34a-3p | 0 | 0.229442469 | 0 | 0.25085307 | -2.123795642 | 0.315638314 | 2.123795642 | -- | 0 |
| hsa-miR-361-3p | 88.86318529 | 94.37068504 | 80.32175218 | 0.017077066 | 0.08675292 | 0.00529697 | -0.232548034 | -0.1374603 | -0.145795114 |
| hsa-miR-365b-5p | 4.659682557 | 1.147212345 | 3.196194291 | 0.002624756 | -2.022099221 | 0.034434148 | 1.478222656 | -0.535517078 | -0.543876564 |
| hsa-miR-370-5p | 0 | 0.229442469 | 0.290563117 | 0.25085307 | -2.123795642 | 0.286090674 | 0.340719133 | 21.48197676 | -1.783076509 |
| hsa-miR-376a-5p | 0.29123016 | 1.147212345 | 0.581126235 | 0.085038376 | 1.977900779 | 0.147080773 | -0.981208962 | 1.005180239 | 0.996691817 |
| hsa-miR-376c-5p | 4.881572203 | 3.845892812 | 4.8703913 | 0.071662188 | -0.344027316 | 0.091616251 | 0.340719133 | 0.005026119 | -0.003308183 |
| hsa-miR-378d | 10.89200798 | 10.60024206 | 15.98097145 | 0.061906941 | -0.039172734 | 0.017337078 | 0.5922579 | 0.561422109 | 0.553085166 |
| hsa-miR-378f | 0.961059527 | 0.252386716 | 0 | 0.107210574 | -1.928989816 | 0.315638314 | 1.986292118 | -23.06200528 | 0.057302302 |
| hsa-miR-379-3p | 11.35797623 | 23.17368936 | 16.56209769 | 3.73E-05 | 1.028781948 | 0.012543577 | -0.484602336 | 0.552509918 | 0.544179612 |
| hsa-miR-411-3p | 16.60011911 | 31.89250318 | 20.0488551 | 7.39E-06 | 0.942023743 | 0.000761835 | -0.669697483 | 0.280654565 | 0.27232626 |
| hsa-miR-431-3p | 4.077222237 | 3.900521971 | 4.067883643 | 0.085114466 | -0.063919396 | 0.108830484 | 0.060611214 | 0.00503928 | -0.003308183 |
| hsa-miR-431-5p | 21.35687839 | 55.52507748 | 37.44113312 | 0 | 1.378438709 | 0.000184491 | -0.568515403 | 0.818255986 | 0.809923306 |
| hsa-miR-487b-5p | 0.29123016 | 0.229442469 | 0 | 0.227853908 | -0.344027316 | 0.315638314 | 2.123795642 | -21.47704278 | 1.779768326 |
| hsa-miR-500a-3p | 29.41424614 | 38.77577725 | 38.35433149 | 0.004006575 | 0.398640638 | 0.046464923 | -0.015766184 | 0.391211125 | 0.382874454 |
| hsa-miR-539-3p | 3.785992078 | 3.671079503 | 2.615068056 | 0.08676524 | -0.044467034 | 0.094555391 | -0.489355866 | -0.525492594 | -0.5338229 |
| hsa-miR-541-5p | 12.04527941 | 20.79666538 | 10.48351727 | 0.000473487 | 0.78788436 | 0.000118548 | -0.98822939 | -0.192008294 | -0.20034503 |
| hsa-miR-6516-3p | 1.747380959 | 5.277176785 | 4.067883643 | 0.005414621 | 1.59457214 | 0.084160263 | -0.375487901 | 1.227419934 | 1.219084239 |
| hsa-miR-6516-5p | 4.368452397 | 6.424389129 | 3.196194291 | 0.043279691 | 0.556437011 | 0.0224801 | -1.007204171 | -0.442411792 | -0.45076716 |
| hsa-miR-92a-1-5p | 8.07847139 | 0.877866838 | 1.389649692 | 0 | -3.202008311 | 0.139046575 | 0.662647228 | -2.530990079 | -2.539361083 |
| hsa-miR-92b-5p | 1.456150799 | 0.917769876 | 0.581126235 | 0.112137039 | -0.66595541 | 0.181619692 | -0.659280867 | -1.316846701 | -1.325236278 |
| hsa-miR-99b-3p | 11.35797623 | 12.61933579 | 15.69040834 | 0.051503402 | 0.151930179 | 0.043436772 | 0.314246921 | 0.474507406 | 0.4661771 |
| ipu-miR-107b | 0.674427739 | 1.594021363 | 0.336441504 | 0.095170799 | 1.240935185 | 0.080863586 | -2.244243368 | -0.995066018 | -1.003308183 |
| ipu-miR-155 | 213.8357448 | 200.8577613 | 246.6396595 | 0.009557532 | -0.090328825 | 6.66E-06 | 0.296230593 | 0.214236818 | 0.205901768 |
| ipu-miR-18a | 15.05659926 | 7.571601474 | 6.712008011 | 0.001346258 | -0.991725572 | 0.086620553 | -0.17385404 | -1.157246879 | -1.165579612 |
| ipu-miR-218a | 114.0457306 | 104.7404871 | 155.0154231 | 0.014702864 | -0.12279322 | 0 | 0.565592544 | 0.451134082 | 0.442799323 |
| lla-miR-139 | 45.91728853 | 67.30312421 | 63.92388582 | 8.45E-05 | 0.551636025 | 0.038352898 | -0.074318367 | 0.485653942 | 0.477317658 |
| mdo-miR-125b-2-3p | 7.017260041 | 16.10467615 | 16.13317118 | 0.000157463 | 1.198499919 | 0.065973469 | 0.002550397 | 1.209395289 | 1.201050316 |
| mdo-miR-135b-5p | 1.456150799 | 1.147212345 | 0.581126235 | 0.120876304 | -0.344027316 | 0.147080773 | -0.981208962 | -1.316846701 | -1.325236278 |
| mdo-miR-145-3p | 41.0052065 | 49.72018301 | 50.81948923 | 0.009177948 | 0.278024504 | 0.0436536 | 0.031550269 | 0.317910849 | 0.309574772 |
| mdo-miR-17-3p | 0.915294788 | 1.442209805 | 1.521997281 | 0.115981432 | 0.655972684 | 0.153188595 | 0.077684727 | 0.742096585 | 0.733657411 |
| mdo-miR-181a-1-3p | 42.71375677 | 20.67167387 | 43.22472279 | 0 | -1.047045578 | 0 | 1.064201498 | 0.025490438 | 0.01715592 |
| mdo-miR-18a-3p | 0.800882939 | 0.841289053 | 0.799048573 | 0.147179596 | 0.071010184 | 0.185895435 | -0.074318367 | 0.005098158 | -0.003308183 |
| mdo-miR-199b-2-5p | 5.824603196 | 10.09546863 | 10.16970911 | 0.010645221 | 0.793476208 | 0.078185018 | 0.010570531 | 0.812383636 | 0.804046739 |
| mdo-miR-218-2-3p | 0 | 0 | 0.304399456 | -- | 0 | 0.26087974 | -1.715962313 | 21.48197676 | -1.715962313 |
| mdo-miR-23a-3p | 584.0038395 | 710.2162183 | 975.798117 | 0 | 0.282280452 | 0 | 0.458324394 | 0.748940073 | 0.740604845 |
| mdo-miR-24-5p | 21.89080035 | 17.03610332 | 18.37811717 | 0.016535877 | -0.361729317 | 0.056776959 | 0.109393587 | -0.244001716 | -0.252335731 |
| mdo-miR-30a-3p | 2.745884364 | 7.932153925 | 7.914385863 | 0.001397232 | 1.530441802 | 0.086925753 | -0.003235268 | 1.535564274 | 1.527206534 |
| mdo-miR-34a-3p | 0 | 0.229442469 | 0 | 0.25085307 | -2.123795642 | 0.315638314 | 2.123795642 | -- | 0 |
| mml-miR-106b-3p | 65.2355558 | 83.51705868 | 75.83697363 | 0.00045506 | 0.356412403 | 0.02096209 | -0.139169511 | 0.22557799 | 0.217242892 |
| mml-miR-127-5p | 23.58964294 | 47.72403353 | 36.61095279 | 0 | 1.0165624 | 0.004800202 | -0.382440662 | 0.642458768 | 0.634121738 |
| mml-miR-1296-5p | 2.78567979 | 2.414133803 | 2.501369445 | 0.095584316 | -0.206523792 | 0.126621282 | 0.051212516 | -0.14700378 | -0.155311276 |
| mml-miR-145-3p | 35.65670131 | 43.23494175 | 44.1908602 | 0.009177948 | 0.278024504 | 0.0436536 | 0.031550269 | 0.317910849 | 0.309574772 |
| mml-miR-1911-5p | 0.29123016 | 1.376654813 | 2.033941822 | 0.062662052 | 2.240935185 | 0.124855427 | 0.563111554 | 2.812429628 | 2.804046739 |
| mml-miR-195-3p | 49.30653227 | 34.23680667 | 39.46605124 | 0.000295349 | -0.526230647 | 0.027499429 | 0.205064033 | -0.312833351 | -0.321166613 |
| mml-miR-26b-3p | 0 | 0.240368301 | 0.304399456 | 0.25085307 | -2.056681446 | 0.286090674 | 0.340719133 | 21.48197676 | -1.715962313 |
| mml-miR-299-3p | 30.28793662 | 39.46410465 | 39.80714708 | 0.004551372 | 0.381797721 | 0.046152051 | 0.012486461 | 0.402619281 | 0.394284182 |
| mml-miR-29c-5p | 1.220393051 | 1.201841504 | 1.521997281 | 0.135007553 | -0.022099221 | 0.15082115 | 0.340719133 | 0.326935531 | 0.318619912 |
| mml-miR-3059-5p | 0 | 0.229442469 | 0 | 0.25085307 | -2.123795642 | 0.315638314 | 2.123795642 | -- | 0 |
| mml-miR-30c-1-3p | 0.915294788 | 1.442209805 | 1.217597825 | 0.115981432 | 0.655972684 | 0.156876716 | -0.244243368 | 0.420094615 | 0.411729316 |
| mml-miR-376a-1-5p | 0.278567979 | 1.097333547 | 0.555859877 | 0.085038376 | 1.977900779 | 0.147080773 | -0.981208962 | 1.005180239 | 0.996691817 |
| mml-miR-380-5p | 0 | 0.229442469 | 0.581126235 | 0.25085307 | -2.123795642 | 0.200950336 | 1.340719133 | 22.48222302 | -0.783076509 |
| mml-miR-382-3p | 70.75626665 | 125.0960244 | 106.1692364 | 0 | 0.822106107 | 0.002306934 | -0.236670148 | 0.593771798 | 0.585435958 |
| mml-miR-412-5p | 33.95743663 | 65.11577268 | 41.23090635 | 0 | 0.939279485 | 1.31E-05 | -0.659280867 | 0.288334176 | 0.279998618 |
| mml-miR-424-3p | 61.62984906 | 68.50496572 | 46.57311681 | 0.015270558 | 0.152579311 | 5.65E-05 | -0.556711134 | -0.395795663 | -0.404131823 |
| mml-miR-543-5p | 0 | 0.458884938 | 0.290563117 | 0.152468748 | -1.123795642 | 0.247312928 | -0.659280867 | 21.48197676 | -1.783076509 |
| mml-miR-7178-5p | 0 | 0 | 0.290563117 | -- | 0 | 0.26087974 | -1.783076509 | 21.48197676 | -1.783076509 |
| mml-miR-7180-3p | 2.135687839 | 2.884419609 | 6.696788038 | 0.088424008 | 0.433580263 | 0.012597709 | 1.215188251 | 1.657096788 | 1.648768514 |
| mml-miR-99b-3p | 11.89883224 | 13.22025654 | 16.43757064 | 0.051503402 | 0.151930179 | 0.043436772 | 0.314246921 | 0.474507406 | 0.4661771 |
| mmu-miR-100-3p | 0.29123016 | 0.688327407 | 1.743378704 | 0.152183516 | 1.240935185 | 0.08996163 | 1.340719133 | 2.590060659 | 2.581654318 |
| mmu-miR-10b-3p | 0.58246032 | 2.06498222 | 1.452815587 | 0.042078378 | 1.825897686 | 0.126492745 | -0.507277774 | 1.327059086 | 1.318619912 |
| mmu-miR-1193-5p | 0.961059527 | 0 | 0.958858287 | 0.059621757 | 0.057302302 | 0.07590956 | -0.060610484 | 0.005098158 | -0.003308183 |
| mmu-miR-1264-5p | 0 | 1.376654813 | 1.162252469 | 0.024994565 | 0.461166859 | 0.156876716 | -0.244243368 | 23.4820999 | 0.216923491 |
| mmu-miR-128-1-5p | 0 | 0 | 0.304399456 | -- | 0 | 0.26087974 | -1.715962313 | 21.48197676 | -1.715962313 |
| mmu-miR-1298-3p | 0 | 0.917769876 | 4.939572995 | 0.064085535 | -0.123795642 | 0.001204747 | 2.428181974 | 25.56961344 | 2.304386332 |
| mmu-miR-132-5p | 1.164920639 | 1.606097282 | 1.452815587 | 0.11418857 | 0.463327607 | 0.148131301 | -0.144707694 | 0.326935531 | 0.318619912 |
| mmu-miR-136-3p | 76.59353203 | 181.4889929 | 117.9686256 | 0 | 1.24458758 | 0 | -0.621478835 | 0.631443153 | 0.623108745 |
| mmu-miR-139-3p | 0.873690479 | 1.147212345 | 2.033941822 | 0.134856116 | 0.392938279 | 0.108711 | 0.82614596 | 1.227467127 | 1.219084239 |
| mmu-miR-144-5p | 4.457087663 | 26.55547184 | 10.28340772 | 0 | 2.574835922 | 0 | -1.368690739 | 1.214498619 | 1.206145183 |
| mmu-miR-145a-3p | 37.27746046 | 45.20016638 | 46.19953566 | 0.009177948 | 0.278024504 | 0.0436536 | 0.031550269 | 0.317910849 | 0.309574772 |
| mmu-miR-1839-3p | 2.78567979 | 2.414133803 | 2.779299383 | 0.095584316 | -0.206523792 | 0.121297295 | 0.203215609 | 0.004982896 | -0.003308183 |
| mmu-miR-1983 | 110.1404728 | 64.1783363 | 117.4981901 | 0 | -0.779186411 | 0 | 0.872480238 | 0.101629217 | 0.093293828 |
| mmu-miR-212-3p | 0 | 0.229442469 | 1.452815587 | 0.25085307 | -2.123795642 | 0.054627842 | 2.662647228 | 23.80410187 | 0.538851586 |
| mmu-miR-223-5p | 0.278567979 | 0 | 0.555859877 | 0.207164791 | 1.843898663 | 0.13358663 | -0.847206846 | 1.005180239 | 0.996691817 |
| mmu-miR-25-5p | 0.58246032 | 0.458884938 | 0.581126235 | 0.174900687 | -0.344027316 | 0.221716937 | 0.340719133 | 0.005180239 | -0.003308183 |
| mmu-miR-29b-2-5p | 0 | 0.201909373 | 0.255695543 | 0.25085307 | -2.308220213 | 0.286090674 | 0.340719133 | 21.48197676 | -1.96750108 |
| mmu-miR-324-3p | 0 | 0.252386716 | 0 | 0.25085307 | -1.986292118 | 0.315638314 | 1.986292118 | -- | 0 |
| mmu-miR-370-5p | 0 | 0.252386716 | 0.319619429 | 0.25085307 | -1.986292118 | 0.286090674 | 0.340719133 | 21.48197676 | -1.645572985 |
| mmu-miR-376a-5p | 0.29123016 | 1.147212345 | 0.581126235 | 0.085038376 | 1.977900779 | 0.147080773 | -0.981208962 | 1.005180239 | 0.996691817 |
| mmu-miR-378b | 6.08671034 | 8.581148337 | 9.268963444 | 0.040371333 | 0.495508012 | 0.082310071 | 0.111237287 | 0.615079265 | 0.606745299 |
| mmu-miR-378c | 18.30589576 | 20.19093726 | 29.52674726 | 0.041798474 | 0.141399512 | 0.00437286 | 0.548314552 | 0.698049655 | 0.689714064 |
| mmu-miR-425-3p | 1.525491313 | 1.922946406 | 1.217597825 | 0.111252526 | 0.33404459 | 0.125444122 | -0.659280867 | -0.316969824 | -0.325236278 |
| mmu-miR-485-3p | 2.038611119 | 3.441637034 | 4.649009878 | 0.060685873 | 0.755508358 | 0.085072973 | 0.433828537 | 1.197662371 | 1.189336895 |
| mmu-miR-493-5p | 418.8196256 | 648.2353543 | 563.2030782 | 0 | 0.630188659 | 0 | -0.202862487 | 0.43566163 | 0.427326172 |
| mmu-miR-758-3p | 12.81412703 | 22.58196931 | 19.85004875 | 0.000930288 | 0.817436107 | 0.049980178 | -0.186028754 | 0.63973844 | 0.631407353 |
| mse-let-7a | 275.183378 | 776.0891511 | 662.8906959 | 0 | 1.495829058 | 0 | -0.227451384 | 1.276713278 | 1.268377675 |
| oan-miR-106-5p | 0.610196525 | 0.240368301 | 0.608798913 | 0.158964182 | -1.344027316 | 0.200950336 | 1.340719133 | 0.005180239 | -0.003308183 |
| oan-miR-143-5p | 24.79255013 | 39.06507427 | 36.68675186 | 9.96E-05 | 0.655972684 | 0.042305166 | -0.090620179 | 0.573690387 | 0.565352506 |
| oan-miR-145-5p | 0 | 0.265670227 | 0 | 0.25085307 | -1.912291536 | 0.315638314 | 1.912291536 | -- | 0 |
| oan-miR-205-5p | 4.881572203 | 2.884419609 | 13.39357608 | 0.04398532 | -0.759064815 | 0 | 2.215188251 | 1.464471729 | 1.456123436 |
| oan-miR-20a-5p | 740.9768956 | 444.7053932 | 428.6096544 | 0 | -0.736578657 | 0.013048105 | -0.053185554 | -0.781428924 | -0.789764212 |
| oan-miR-214-5p | 122.6952663 | 181.4660487 | 143.5091237 | 0 | 0.564620063 | 2.94E-05 | -0.338557193 | 0.234398023 | 0.22606287 |
| oan-miR-24-1-5p | 3.523884934 | 5.047734316 | 7.351246869 | 0.061532811 | 0.518469161 | 0.055280271 | 0.542352994 | 1.069145774 | 1.060822155 |
| oan-miR-32-5p | 27.23001994 | 39.11994095 | 25.88917376 | 0.001294654 | 0.522706154 | 0.00112193 | -0.59555527 | -0.064512283 | -0.072849116 |
| oan-miR-454-5p | 1.392839895 | 0.438933419 | 0 | 0.071126842 | -1.66595541 | 0.193791884 | 1.187925979 | -23.79906972 | -0.478029431 |
| oar-let-7d | 107.6386671 | 138.3079203 | 113.4648973 | 3.45E-05 | 0.361687345 | 0.000846 | -0.285637736 | 0.084384632 | 0.076049609 |
| oar-miR-10a | 7.017260041 | 19.95056896 | 5.174790757 | 0 | 1.50745016 | 0 | -1.946857457 | -0.431055765 | -0.439407298 |
| oar-miR-10b | 516.3510733 | 549.514713 | 619.1900031 | 0.001217459 | 0.089805804 | 1.86E-05 | 0.17222407 | 0.270365178 | 0.262029874 |
| oar-miR-1185-5p | 0 | 0.458884938 | 0.290563117 | 0.152468748 | -1.123795642 | 0.247312928 | -0.659280867 | 21.48197676 | -1.783076509 |
| oar-miR-127 | 684.4415243 | 1185.559164 | 1075.033002 | 0 | 0.792568467 | 0 | -0.141186712 | 0.659717064 | 0.651381754 |
| oar-miR-154a-3p | 22.42472231 | 27.76253874 | 23.53561251 | 0.016061373 | 0.308049381 | 0.033889641 | -0.238294102 | 0.078090678 | 0.069755279 |
| oar-miR-23b | 315.7258521 | 472.5240179 | 508.1948923 | 0 | 0.581715266 | 0.003846072 | 0.104994213 | 0.695044598 | 0.68670948 |
| oar-miR-25 | 176.0416976 | 221.8599416 | 182.0308749 | 0 | 0.333732013 | 4.59E-05 | -0.285466031 | 0.056600824 | 0.048265983 |
| oar-miR-30d | 811.6737833 | 755.0347856 | 824.9545686 | 0.000864183 | -0.104356903 | 0.000208271 | 0.127771557 | 0.031749808 | 0.023414654 |
| oar-miR-323b | 15.5600114 | 17.06614935 | 18.87276629 | 0.046428884 | 0.133294462 | 0.056831837 | 0.145168324 | 0.286794248 | 0.278462786 |
| oar-miR-370-3p | 38.4423811 | 45.63993111 | 32.76099148 | 0.010204528 | 0.247598915 | 0.001171071 | -0.478317594 | -0.222383841 | -0.230718679 |
| oar-miR-374b | 72.00318999 | 129.5585141 | 109.2794048 | 0 | 0.847471098 | 0.002290207 | -0.245582296 | 0.610222953 | 0.601888802 |
| oar-miR-376a-5p | 0.29123016 | 0.917769876 | 0.290563117 | 0.114580875 | 1.655972684 | 0.143485591 | -1.659280867 | 0.004933982 | -0.003308183 |
| oar-miR-379-5p | 436.5540096 | 828.9756402 | 648.2463148 | 0 | 0.925169556 | 0 | -0.354787608 | 0.57871716 | 0.570381947 |
| oar-miR-381-3p | 128.7514668 | 118.7419406 | 117.4981901 | 0.013343071 | -0.116759273 | 0.031675006 | -0.015191062 | -0.12361597 | -0.131950334 |
| oar-miR-3956-5p | 23.67827821 | 33.35893983 | 15.56407655 | 0.001938168 | 0.494509262 | 0 | -1.099853459 | -0.597010512 | -0.605344197 |
| oar-miR-3958-5p | 0.58246032 | 0.688327407 | 1.452815587 | 0.163565675 | 0.240935185 | 0.121131216 | 1.077684727 | 1.327059086 | 1.318619912 |
| oar-miR-409-3p | 160.7337239 | 185.2299027 | 138.9649692 | 0.000571107 | 0.204644365 | 0 | -0.414595771 | -0.201616302 | -0.209951407 |
| oar-miR-487b-5p | 0.29123016 | 0.229442469 | 0 | 0.227853908 | -0.344027316 | 0.315638314 | 2.123795642 | -21.47704278 | 1.779768326 |
| oar-miR-494-5p | 0.278567979 | 0.219466709 | 0.277929938 | 0.227853908 | -0.344027316 | 0.286090674 | 0.340719133 | 0.004933982 | -0.003308183 |
| oar-miR-539-3p | 3.621383726 | 3.51146735 | 2.501369445 | 0.08676524 | -0.044467034 | 0.094555391 | -0.489355866 | -0.525492594 | -0.5338229 |
| oar-miR-543-3p | 58.2207076 | 85.15308324 | 57.53149724 | 0 | 0.548526395 | 0 | -0.565706752 | -0.008844944 | -0.017180357 |
| oar-miR-544-3p | 0 | 0.240368301 | 0 | 0.25085307 | -2.056681446 | 0.315638314 | 2.056681446 | -- | 0 |
| oar-miR-654-3p | 46.45121049 | 69.82699137 | 69.2508763 | 3.82E-06 | 0.58806862 | 0.037323889 | -0.011952486 | 0.584451151 | 0.576116134 |
| oar-miR-665-3p | 10.19305559 | 18.12595504 | 20.92054445 | 0.001168413 | 0.830470416 | 0.045227014 | 0.206863386 | 1.045669538 | 1.037333802 |
| oar-miR-758-3p | 12.17342068 | 21.45287084 | 18.85754632 | 0.000930288 | 0.817436107 | 0.049980178 | -0.186028754 | 0.63973844 | 0.631407353 |
| oar-miR-99a | 4232.185805 | 12492.38527 | 7184.725147 | 0 | 1.561574096 | 0 | -0.798044096 | 0.77186529 | 0.76353 |
| ocu-miR-191-3p | 2.912301598 | 1.606097282 | 0.871689352 | 0.062736997 | -0.858600488 | 0.126084122 | -0.881673289 | -1.731966281 | -1.740273777 |
| oha-miR-10c-5p | 454.3889445 | 483.5729475 | 544.8872027 | 0.001217459 | 0.089805804 | 1.86E-05 | 0.17222407 | 0.270365178 | 0.262029874 |
| oha-miR-212-5p | 0.26696098 | 0.210322263 | 0 | 0.227853908 | -0.344027316 | 0.315638314 | 2.249326524 | -21.47704278 | 1.905299208 |
| oha-miR-27b-5p | 23.49256622 | 7.781923737 | 15.18192288 | 0 | -1.594005569 | 0.001148341 | 0.964155781 | -0.621516458 | -0.629849787 |
| oha-miR-30e-5p | 261.1519089 | 211.6010225 | 201.2323926 | 0 | -0.30354265 | 0.015634189 | -0.072484043 | -0.367691014 | -0.376026693 |
| ola-miR-100 | 1373.134876 | 2863.925049 | 2909.546129 | 0 | 1.060520396 | 0.00611217 | 0.022800383 | 1.091656097 | 1.083320779 |
| ola-miR-101a-3p | 117.1577329 | 194.4579553 | 160.4185135 | 0 | 0.731006076 | 0.000149902 | -0.277617608 | 0.461723275 | 0.453388468 |
| ola-miR-106a | 0.305098263 | 0 | 1.521997281 | 0.207164791 | 1.71265413 | 0.022290674 | 0.605965782 | 2.327059086 | 2.318619912 |
| ola-miR-126-3p | 1009.432857 | 1402.51298 | 1501.891697 | 0 | 0.47446917 | 8.31E-05 | 0.09876666 | 0.581571092 | 0.573235831 |
| ola-miR-140-3p | 1076.386671 | 843.2240175 | 1197.93362 | 0 | -0.352208566 | 0 | 0.506560103 | 0.162686822 | 0.154351537 |
| ola-miR-181a-5p | 136.571617 | 268.5925997 | 274.199826 | 0 | 0.975761869 | 0.022659039 | 0.0298081 | 1.013905093 | 1.005569969 |
| ola-miR-192-5p | 15.25491313 | 14.90283465 | 14.6111739 | 0.052790618 | -0.033687195 | 0.068211159 | -0.028514677 | -0.053874608 | -0.062201872 |
| ola-miR-193a | 1.011641608 | 0.531340454 | 0.672883009 | 0.13744665 | -0.928989816 | 0.221716937 | 0.340719133 | -0.579782262 | -0.588270684 |
| ola-miR-194-5p | 12.47691316 | 18.86258613 | 19.17716575 | 0.008521797 | 0.596266438 | 0.064889463 | 0.023862027 | 0.628454661 | 0.620128466 |
| ola-miR-204 | 33.31673028 | 119.1265299 | 73.19284926 | 0 | 1.838176016 | 0 | -0.702720129 | 1.143789777 | 1.135455887 |
| ola-miR-20a | 705.6922815 | 423.5289459 | 407.8952714 | 0 | -0.736578657 | 0.01284148 | -0.054261791 | -0.782505036 | -0.790840448 |
| ola-miR-222 | 23.94218472 | 20.19093726 | 21.86869778 | 0.032250538 | -0.245846922 | 0.057888693 | 0.115159432 | -0.122355193 | -0.130687489 |
| ola-miR-223 | 0.961059527 | 2.019093726 | 1.917716575 | 0.081065193 | 1.071010184 | 0.141457698 | -0.074318367 | 1.005098158 | 0.996691817 |
| ola-miR-24b-3p | 67.63011489 | 147.2255842 | 135.3055583 | 0 | 1.122290689 | 0.01688077 | -0.121807292 | 1.00881818 | 1.000483396 |
| ola-miR-27d-3p | 1.067843919 | 6.730312421 | 4.971857786 | 0.000282022 | 2.655972684 | 0.076894796 | -0.436888446 | 2.227502306 | 2.219084239 |
| ola-miR-29c | 0.355947973 | 0.560859368 | 0.355132699 | 0.195628342 | 0.655972684 | 0.247312928 | -0.659280867 | 0.004933982 | -0.003308183 |
| ola-miR-30a-3p | 0.337213869 | 2.125361817 | 1.682207522 | 0.030308445 | 2.655972684 | 0.138855988 | -0.337352772 | 2.327059086 | 2.318619912 |
| pma-miR-199a-3p | 2805.988722 | 7025.003958 | 5230.191458 | 0 | 1.323989828 | 0 | -0.425635279 | 0.906689864 | 0.898354549 |
| pma-miR-20a-5p | 11.69985512 | 5.047734316 | 3.33515926 | 0.000557946 | -1.212782782 | 0.064111799 | -0.597880323 | -1.802327368 | -1.810663105 |
| ppy-miR-378d | 8.542751354 | 10.37589832 | 14.91557336 | 0.053794752 | 0.280463549 | 0.029635008 | 0.52358319 | 0.812370709 | 0.804046739 |
| ppy-miR-378e | 1.067843919 | 0.841289053 | 0 | 0.149911551 | -0.344027316 | 0.12613315 | 0.249326524 | -23.06200528 | -0.094700792 |
| prd-let-7-5p | 4594.978813 | 6639.745826 | 6220.62788 | 0 | 0.531069806 | 0 | -0.094067809 | 0.445337307 | 0.437001997 |
| ptr-miR-320d | 0 | 1.121718737 | 0.355132699 | 0.064085535 | 0.165710976 | 0.143485591 | -1.659280867 | 21.48197676 | -1.493569892 |
| rno-miR-10b-3p | 0.610196525 | 2.163314707 | 1.521997281 | 0.042078378 | 1.825897686 | 0.126492745 | -0.507277774 | 1.327059086 | 1.318619912 |
| rno-miR-127-5p | 22.84257427 | 45.86854226 | 35.57503211 | 0 | 1.005779812 | 0.005564197 | -0.366639999 | 0.647478957 | 0.639139813 |
| rno-miR-1306-3p | 1.747380959 | 2.294424689 | 1.452815587 | 0.098271658 | 0.392938279 | 0.112092073 | -0.659280867 | -0.257985786 | -0.266342589 |
| rno-miR-145-3p | 39.05257762 | 47.35255525 | 48.39951355 | 0.009177948 | 0.278024504 | 0.0436536 | 0.031550269 | 0.317910849 | 0.309574772 |
| rno-miR-15a-5p | 6.744277385 | 9.032787723 | 9.420362121 | 0.04638604 | 0.421507431 | 0.084871206 | 0.060611214 | 0.490462577 | 0.482118644 |
| rno-miR-16-3p | 1.525491313 | 2.884419609 | 1.826396738 | 0.063235077 | 0.91900709 | 0.099764444 | -0.659280867 | 0.268033719 | 0.259726223 |
| rno-miR-18a-3p | 0.915294788 | 0.961473203 | 0.913198369 | 0.147179596 | 0.071010184 | 0.185895435 | -0.074318367 | 0.005098158 | -0.003308183 |
| rno-miR-204-3p | 1.164920639 | 6.653831598 | 4.067883643 | 9.70E-05 | 2.51395368 | 0.041841021 | -0.70990694 | 1.812341251 | 1.804046739 |
| rno-miR-221-5p | 12.81412703 | 8.559201666 | 13.34063704 | 0.014834373 | -0.582187053 | 0.015552345 | 0.640279415 | 0.066423494 | 0.058092362 |
| rno-miR-24-1-5p | 4.368452397 | 5.736061723 | 7.264077934 | 0.060690946 | 0.392938279 | 0.071140764 | 0.340719133 | 0.742010988 | 0.733657411 |
| rno-miR-299a-3p | 31.73021932 | 41.34334773 | 41.70272551 | 0.004551372 | 0.381797721 | 0.046152051 | 0.012486461 | 0.402619281 | 0.394284182 |
| rno-miR-370-5p | 0 | 0.210322263 | 0.266349524 | 0.25085307 | -2.249326524 | 0.286090674 | 0.340719133 | 21.48197676 | -1.908607391 |
| rno-miR-376c-5p | 4.881572203 | 3.845892812 | 4.8703913 | 0.071662188 | -0.344027316 | 0.091616251 | 0.340719133 | 0.005026119 | -0.003308183 |
| rno-miR-379-3p | 11.35797623 | 23.17368936 | 16.56209769 | 3.73E-05 | 1.028781948 | 0.012543577 | -0.484602336 | 0.552509918 | 0.544179612 |
| rno-miR-409a-3p | 184.8437824 | 213.0143881 | 159.4900951 | 0.000571107 | 0.204644365 | 0 | -0.417484051 | -0.204504246 | -0.212839686 |
| rno-miR-411-3p | 18.26013102 | 35.0817535 | 22.05374061 | 7.39E-06 | 0.942023743 | 0.000761835 | -0.669697483 | 0.280654565 | 0.27232626 |
| rno-miR-412-3p | 1.456150799 | 0.688327407 | 0.290563117 | 0.095554794 | -1.08099291 | 0.192756666 | -1.244243368 | -2.317092958 | -2.325236278 |
| rno-miR-452-3p | 18.30589576 | 18.26799086 | 14.6111739 | 0.049779087 | -0.002990398 | 0.038742238 | -0.32224588 | -0.316904071 | -0.325236278 |
| rno-miR-452-5p | 323.6959915 | 211.5659079 | 199.5536957 | 0 | -0.613532333 | 0.0144392 | -0.08433017 | -0.689527393 | -0.697862502 |
| rno-miR-98-3p | 0.610196525 | 0.240368301 | 0.304399456 | 0.158964182 | -1.344027316 | 0.286090674 | 0.340719133 | -0.995066018 | -1.003308183 |
| sha-miR-10b | 575.9238205 | 613.0192897 | 689.6677014 | 0.001523794 | 0.090054478 | 4.07E-05 | 0.169968932 | 0.268358685 | 0.26002341 |
| sha-miR-126 | 2681.187474 | 3414.9251 | 3544.411248 | 0 | 0.348981837 | 0.000346091 | 0.053692069 | 0.411009162 | 0.402673906 |
| sha-miR-181a-3p | 47.54715557 | 23.37897999 | 48.78401812 | 0 | -1.024147049 | 0 | 1.061196604 | 0.045384551 | 0.037049555 |
| sha-miR-193 | 35.08630021 | 44.94887224 | 37.44113312 | 0.004713525 | 0.357377093 | 0.018173893 | -0.263660822 | 0.102053557 | 0.093716272 |
| sha-miR-21 | 55128.93732 | 40103.84532 | 42131.21036 | 0 | -0.459069217 | 0 | 0.071148788 | -0.379585142 | -0.387920429 |
| sha-miR-221 | 44.54434635 | 49.51586996 | 53.26990485 | 0.021091694 | 0.152648653 | 0.036892302 | 0.105429717 | 0.266414929 | 0.25807837 |
| sha-miR-24 | 253.9220435 | 321.9923153 | 329.7126742 | 0 | 0.342640613 | 0.020420914 | 0.034183088 | 0.385159058 | 0.376823702 |
| sha-miR-25 | 194.5724026 | 245.2136197 | 201.1920196 | 0 | 0.333732013 | 4.59E-05 | -0.285466031 | 0.056600824 | 0.048265983 |
| sha-miR-9 | 1.525491313 | 3.12478791 | 2.130796194 | 0.051613846 | 1.034484308 | 0.101711574 | -0.552365663 | 0.490402688 | 0.482118644 |
| sha-miR-92a | 652.8085827 | 722.3868666 | 594.4921381 | 3.16E-05 | 0.146111639 | 0 | -0.281113933 | -0.126666799 | -0.135002294 |
| sha-miR-93 | 171.7703219 | 136.2888265 | 129.0653695 | 3.16E-05 | -0.333813503 | 0.022781293 | -0.078565338 | -0.404044019 | -0.41237884 |
| ssa-miR-144-3p | 0 | 0.504773432 | 0 | 0.152468748 | -0.986292118 | 0.193791884 | 0.986292118 | -- | 0 |
| ssa-miR-144-5p | 1.601765879 | 11.35740221 | 4.527941912 | 0 | 2.825897686 | 0.000516584 | -1.326705528 | 1.507525784 | 1.499192158 |
| ssc-let-7i | 22144.83479 | 30393.73667 | 24900.03573 | 0 | 0.456803819 | 0 | -0.287626241 | 0.177512867 | 0.169177578 |
| ssc-miR-151-3p | 1495.17564 | 1776.573037 | 1596.934893 | 0 | 0.248782031 | 0 | -0.153791505 | 0.103325858 | 0.094990525 |
| ssc-miR-199b-5p | 4585.418866 | 5731.013988 | 4997.976182 | 0 | 0.321736891 | 0 | -0.197446391 | 0.132625773 | 0.1242905 |
| ssc-miR-296-3p | 27.7639419 | 23.31572517 | 28.61354889 | 0.025074302 | -0.251909114 | 0.026887634 | 0.295395142 | 0.051819827 | 0.043486029 |
| ssc-miR-299 | 2.135687839 | 4.326629414 | 3.348394019 | 0.034181107 | 1.018542764 | 0.096049901 | -0.36977425 | 0.657119175 | 0.648768514 |
| ssc-miR-30c-1-3p | 0.961059527 | 1.514320295 | 1.278477716 | 0.115981432 | 0.655972684 | 0.156876716 | -0.244243368 | 0.420094615 | 0.411729316 |
| ssc-miR-320 | 145.969621 | 125.7544245 | 95.32996885 | 0.001748044 | -0.215058988 | 1.86E-05 | -0.39960743 | -0.60633076 | -0.614666418 |
| ssc-miR-339 | 14.94981487 | 48.07366015 | 43.22472279 | 0 | 1.68511903 | 0.031714443 | -0.153389938 | 1.540067141 | 1.531729093 |
| ssc-miR-345-3p | 1.830589576 | 3.12478791 | 1.826396738 | 0.066648537 | 0.771449902 | 0.086520135 | -0.774758085 | 0.005015787 | -0.003308183 |
| ssc-miR-361-3p | 85.16055257 | 90.43857316 | 76.97501251 | 0.017077066 | 0.08675292 | 0.00529697 | -0.232548034 | -0.1374603 | -0.145795114 |
| ssc-miR-362 | 28.19107947 | 26.65203719 | 28.89359639 | 0.037121992 | -0.08099291 | 0.043679536 | 0.116503976 | 0.043842479 | 0.035511066 |
| ssc-miR-382 | 73.83377956 | 149.5090831 | 86.44944558 | 0 | 1.017880217 | 0 | -0.790304518 | 0.235912565 | 0.227575699 |
| ssc-miR-411 | 18.26013102 | 35.0817535 | 22.05374061 | 7.39E-06 | 0.942023743 | 0.000761835 | -0.669697483 | 0.280654565 | 0.27232626 |
| ssc-miR-432-5p | 16.47530618 | 28.60382779 | 21.30796194 | 0.000190212 | 0.795902946 | 0.01311018 | -0.424815614 | 0.379425144 | 0.371087332 |
| ssc-miR-542-5p | 1.525491313 | 3.12478791 | 2.739595107 | 0.051613846 | 1.034484308 | 0.121146579 | -0.189795584 | 0.85299622 | 0.844688724 |
| ssc-miR-652 | 2.710680718 | 2.135579903 | 4.425499787 | 0.085702667 | -0.344027316 | 0.028315176 | 1.051212516 | 0.715501681 | 0.7071852 |
| ssc-miR-758 | 11.59373398 | 20.43130556 | 17.95956792 | 0.000930288 | 0.817436107 | 0.049980178 | -0.186028754 | 0.63973844 | 0.631407353 |
| ssc-miR-7857-3p | 39.31607157 | 26.15644146 | 50.2674193 | 0.00045506 | -0.587952898 | 0 | 0.942457346 | 0.362841365 | 0.354504448 |
| tch-miR-140-5p | 358.7955569 | 204.0726873 | 239.8667715 | 0 | -0.814078917 | 0.00028678 | 0.233150209 | -0.572593421 | -0.580928708 |
| tch-miR-194-5p | 10.30701522 | 15.58213637 | 15.84200649 | 0.008521797 | 0.596266438 | 0.064889463 | 0.023862027 | 0.628454661 | 0.620128466 |
| tch-miR-23b-3p | 276.5715751 | 395.616177 | 425.8928893 | 0 | 0.51644853 | 0.002830575 | 0.106389223 | 0.631173061 | 0.622837753 |
| tch-miR-30a-5p | 220.0826318 | 502.7543379 | 437.8786179 | 0 | 1.191808328 | 1.86E-05 | -0.199322621 | 1.000821304 | 0.992485706 |
| tch-miR-361-5p | 14.33961835 | 23.79646178 | 27.39595107 | 0.000991945 | 0.730740453 | 0.040570473 | 0.203215609 | 0.942294266 | 0.933956062 |
| tch-miR-374b-5p | 65.46347505 | 118.2925564 | 99.77684787 | 0 | 0.853597201 | 0.002290207 | -0.245582296 | 0.616350838 | 0.608014904 |
| tgu-miR-126-5p | 20.50260325 | 33.06265977 | 32.28156234 | 0.00039479 | 0.689395686 | 0.051154407 | -0.034492386 | 0.663234043 | 0.6549033 |
| tgu-miR-138-1-3p | 0.305098263 | 0 | 0.608798913 | 0.207164791 | 1.71265413 | 0.13358663 | -0.715962313 | 1.005180239 | 0.996691817 |
| tgu-miR-155-5p | 247.434691 | 230.993937 | 284.6134916 | 0.008277346 | -0.099192799 | 0 | 0.301149067 | 0.210291597 | 0.201956268 |
| tgu-miR-181b-1-3p | 0.915294788 | 1.682578105 | 2.130796194 | 0.098534335 | 0.878365106 | 0.131350463 | 0.340719133 | 1.227467127 | 1.219084239 |
| tgu-miR-29a-1-5p | 0.278567979 | 0.219466709 | 0.277929938 | 0.227853908 | -0.344027316 | 0.286090674 | 0.340719133 | 0.004933982 | -0.003308183 |
| tgu-miR-29b-2-5p | 0 | 0.229442469 | 0.290563117 | 0.25085307 | -2.123795642 | 0.286090674 | 0.340719133 | 21.48197676 | -1.783076509 |
| tgu-miR-365-1-5p | 23.95684619 | 8.778668376 | 15.00821667 | 0 | -1.448363975 | 0.005257286 | 0.77367854 | -0.666353804 | -0.674685435 |
| CM008008.1_10340 | 11.94043655 | 10.78379604 | 5.230136112 | 0.053858671 | -0.146990468 | 0.004772758 | -1.043944718 | -1.182601171 | -1.190935186 |
| CM008008.1_5534 | 0.58246032 | 0.688327407 | 1.162252469 | 0.163565675 | 0.240935185 | 0.151642571 | 0.755756632 | 1.005057116 | 0.996691817 |
| CM008008.1_6671 | 0.873690479 | 0.458884938 | 1.162252469 | 0.13744665 | -0.928989816 | 0.126051039 | 1.340719133 | 0.420094615 | 0.411729316 |
| CM008008.1_7149 | 0.873690479 | 0.229442469 | 0.290563117 | 0.107210574 | -1.928989816 | 0.286090674 | 0.340719133 | -1.580028518 | -1.588270684 |
| CM008008.1_9244 | 41.79846198 | 28.84419609 | 38.04993204 | 0.000873293 | -0.535168803 | 0.008377196 | 0.399612822 | -0.127220145 | -0.135555981 |
| CM008008.1_9743 | 0.674427739 | 1.85969159 | 1.345766017 | 0.073694749 | 1.463327607 | 0.142404131 | -0.466635789 | 1.005057116 | 0.996691817 |
| CM008009.1_14936 | 2.360497085 | 3.453712953 | 3.027973539 | 0.079589849 | 0.549057481 | 0.121146579 | -0.189795584 | 0.367597633 | 0.359261897 |
| CM008009.1_15028 | 1.164920639 | 2.753309627 | 1.162252469 | 0.046435012 | 1.240935185 | 0.060119616 | -1.244243368 | 0.004933561 | -0.003308183 |
| CM008009.1_16740 | 0.640706352 | 0 | 0.319619429 | 0.10800582 | 0.642264802 | 0.26087974 | -1.645572985 | -0.995066018 | -1.003308183 |
| CM008009.1_16903 | 0.29123016 | 0.458884938 | 1.743378704 | 0.195628342 | 0.655972684 | 0.060821177 | 1.925681633 | 2.590060659 | 2.581654318 |
| CM008010.1_17978 | 2.329841278 | 1.835539751 | 2.033941822 | 0.099187354 | -0.344027316 | 0.1359945 | 0.148074055 | -0.187632151 | -0.195953261 |
| CM008010.1_21055 | 1.830589576 | 0 | 0 | 0.007561845 | -0.872308371 | -- | 0 | -24.06208765 | -0.872308371 |
| CM008010.1_21891 | 0.320353176 | 0.504773432 | 0.639238858 | 0.195628342 | 0.655972684 | 0.221716937 | 0.340719133 | 1.005180239 | 0.996691817 |
| CM008010.1_26918 | 0.640706352 | 0 | 0.319619429 | 0.10800582 | 0.642264802 | 0.26087974 | -1.645572985 | -0.995066018 | -1.003308183 |
| CM008011.1_31347 | 2.507111811 | 1.316800256 | 0.277929938 | 0.06463625 | -0.928989816 | 0.080863586 | -2.244243368 | -3.165045934 | -3.173233184 |
| CM008011.1_33842 | 1.423791892 | 2.243437474 | 15.27070606 | 0.09921642 | 0.655972684 | 0 | 2.766983887 | 3.431246993 | 3.422956572 |
| CM008011.1_37433 | 1.686069346 | 6.110415225 | 3.027973539 | 0.002673633 | 1.857606546 | 0.03185032 | -1.012917822 | 0.85299622 | 0.844688724 |
| CM008011.1_37670 | 2.912301598 | 1.376654813 | 2.905631174 | 0.049883985 | -1.08099291 | 0.063353914 | 1.077684727 | 0.004982896 | -0.003308183 |
| CM008012.1_42107 | 0.873690479 | 0.688327407 | 0.290563117 | 0.149911551 | -0.344027316 | 0.192756666 | -1.244243368 | -1.580028518 | -1.588270684 |
| CM008012.1_42960 | 0.29123016 | 0.458884938 | 0.581126235 | 0.195628342 | 0.655972684 | 0.221716937 | 0.340719133 | 1.005180239 | 0.996691817 |
| CM008012.1_43443 | 0.58246032 | 1.835539751 | 0.290563117 | 0.056978612 | 1.655972684 | 0.041197319 | -2.659280867 | -0.995066018 | -1.003308183 |
| CM008012.1_46261 | 4.046566431 | 8.767117496 | 6.055947078 | 0.006958413 | 1.115404303 | 0.050259499 | -0.533749985 | 0.589978287 | 0.581654318 |
| CM008012.1_46537 | 0 | 1.147212345 | 3.777320526 | 0.041163135 | 0.198132453 | 0.016344202 | 1.719230756 | 25.18256803 | 1.917363209 |
| CM008012.1_47852 | 0.873690479 | 0.458884938 | 0.290563117 | 0.13744665 | -0.928989816 | 0.247312928 | -0.659280867 | -1.580028518 | -1.588270684 |
| CM008012.1_48395 | 9.15294788 | 2.403683008 | 4.261592388 | 8.45E-05 | -1.928989816 | 0.058830976 | 0.82614596 | -1.094508161 | -1.102843856 |
| CM008012.1_48925 | 0 | 0.917769876 | 1.162252469 | 0.064085535 | -0.123795642 | 0.167109089 | 0.340719133 | 23.4820999 | 0.216923491 |
| CM008012.1_49180 | 0 | 0.480736602 | 0 | 0.152468748 | -1.056681446 | 0.193791884 | 1.056681446 | -- | 0 |
| CM008012.1_50670 | 0.278567979 | 1.316800256 | 0.555859877 | 0.062662052 | 2.240935185 | 0.121908424 | -1.244243368 | 1.005180239 | 0.996691817 |
| CM008012.1_52630 | 6.115833356 | 5.047734316 | 4.35844676 | 0.066055586 | -0.27691312 | 0.095910663 | -0.21182189 | -0.480399273 | -0.48873501 |
| CM008012.1_53957 | 0 | 0.458884938 | 0 | 0.152468748 | -1.123795642 | 0.193791884 | 1.123795642 | -- | 0 |
| CM008012.1_55199 | 0.557135958 | 0.877866838 | 0 | 0.143516961 | 0.655972684 | 0.082988013 | 0.187925979 | -22.47704278 | 0.843898663 |
| CM008012.1_55392 | 0.58246032 | 1.606097282 | 0.581126235 | 0.073694749 | 1.463327607 | 0.095458439 | -1.466635789 | 0.005180239 | -0.003308183 |
| CM008012.1_55433 | 1.671407874 | 18.6546703 | 175.651721 | 0 | 3.48040112 | 0 | 3.235108945 | 6.723837411 | 6.715510065 |
| CM008012.1_56259 | 1.220393051 | 0.721104902 | 0 | 0.121667271 | -0.759064815 | 0.12613315 | 0.471718945 | -23.47716634 | -0.28734587 |
| CM008012.1_61980 | 2.038611119 | 1.835539751 | 1.162252469 | 0.109338053 | -0.151382238 | 0.125444122 | -0.659280867 | -0.80236841 | -0.810663105 |
| CM008012.1_62189 | 0.58246032 | 1.147212345 | 0.290563117 | 0.116762733 | 0.977900779 | 0.109522678 | -1.981208962 | -0.995066018 | -1.003308183 |
| CM008012.1_63603 | 17.47380959 | 15.14320295 | 17.43378704 | 0.040771781 | -0.206523792 | 0.051579563 | 0.203215609 | 0.005024024 | -0.003308183 |
| CM008013.1_67139 | 0.278567979 | 0.658400128 | 0.833789815 | 0.152183516 | 1.240935185 | 0.188804199 | 0.340719133 | 1.590060659 | 1.581654318 |
| CM008013.1_68686 | 1.456150799 | 1.835539751 | 0.871689352 | 0.111252526 | 0.33404459 | 0.104224791 | -1.074318367 | -0.731966281 | -0.740273777 |
| CM008014.1_69773 | 0.873690479 | 0.688327407 | 0.871689352 | 0.149911551 | -0.344027316 | 0.188804199 | 0.340719133 | 0.005098158 | -0.003308183 |
| CM008014.1_74801 | 1.114271916 | 1.316800256 | 1.66757963 | 0.125796769 | 0.240935185 | 0.139933788 | 0.340719133 | 0.589937103 | 0.581654318 |
| CM008014.1_76350 | 0.58246032 | 0.458884938 | 0.581126235 | 0.174900687 | -0.344027316 | 0.221716937 | 0.340719133 | 0.005180239 | -0.003308183 |
| CM008015.1_78022 | 0 | 0 | 3.196194291 | -- | 0 | 0.000693779 | 1.67635511 | 24.80405262 | 1.67635511 |
| CM008015.1_81455 | 2.621071438 | 0.458884938 | 0.871689352 | 0.010645221 | -2.513952317 | 0.172123693 | 0.925681633 | -1.579919257 | -1.588270684 |
| CM008015.1_83294 | 0 | 0.797010681 | 12.11189416 | 0.09860269 | -0.327329036 | 0 | 3.925681633 | 26.65206594 | 3.598352598 |
| CM008016.1_100323 | 2.038611119 | 2.294424689 | 1.452815587 | 0.104343066 | 0.170545857 | 0.112092073 | -0.659280867 | -0.48036644 | -0.48873501 |
| CM008016.1_100327 | 0 | 0.458884938 | 0.581126235 | 0.152468748 | -1.123795642 | 0.221716937 | 0.340719133 | 22.48222302 | -0.783076509 |
| CM008016.1_84545 | 0.26696098 | 1.051611316 | 7.191437155 | 0.085038376 | 1.977900779 | 1.31E-05 | 2.77367854 | 4.75998566 | 4.751579319 |
| CM008016.1_87034 | 5.014223621 | 4.828267607 | 5.836528705 | 0.077375187 | -0.054520698 | 0.084963729 | 0.273604937 | 0.227408208 | 0.219084239 |
| CM008016.1_88050 | 0.610196525 | 1.201841504 | 0 | 0.116762733 | 0.977900779 | 0.053049451 | -0.265246649 | -22.47704278 | 0.71265413 |
| CM008016.1_89428 | 1.747380959 | 1.606097282 | 3.196194291 | 0.115094418 | -0.121634894 | 0.062369894 | 0.992795829 | 0.879499829 | 0.871160935 |
| CM008016.1_90056 | 1.671407874 | 1.755733675 | 2.223439507 | 0.113865386 | 0.071010184 | 0.126452118 | 0.340719133 | 0.420073807 | 0.411729316 |
| CM008016.1_90073 | 0.58246032 | 0.688327407 | 2.905631174 | 0.163565675 | 0.240935185 | 0.019616365 | 2.077684727 | 2.327009836 | 2.318619912 |
| CM008016.1_90401 | 4.538336657 | 2.523867158 | 3.462543815 | 0.035282172 | -0.846527656 | 0.094342583 | 0.45619635 | -0.38202481 | -0.390331306 |
| CM008016.1_93249 | 0.557135958 | 0.877866838 | 0.555859877 | 0.143516961 | 0.655972684 | 0.181619692 | -0.659280867 | 0.005180239 | -0.003308183 |
| CM008016.1_95226 | 3.785992078 | 0.688327407 | 2.905631174 | 0.002671834 | -2.459504533 | 0.019616365 | 2.077684727 | -0.373505917 | -0.381819806 |
| CM008016.1_96188 | 0.58246032 | 1.376654813 | 0.581126235 | 0.095170799 | 1.240935185 | 0.121908424 | -1.244243368 | 0.005180239 | -0.003308183 |
| CM008016.1_97064 | 1.164920639 | 1.606097282 | 0.581126235 | 0.11418857 | 0.463327607 | 0.095458439 | -1.466635789 | -0.994943316 | -1.003308183 |
| CM008016.1_98387 | 5.2927916 | 4.389334188 | 4.724808952 | 0.070369965 | -0.270026734 | 0.100155439 | 0.106253879 | -0.155434893 | -0.163772855 |
| CM008016.1_98716 | 17.79739866 | 18.22792947 | 13.13990986 | 0.051906 | 0.034484308 | 0.027464627 | -0.472195315 | -0.429374869 | -0.437711007 |
| CM008017.1_101998 | 3.785992078 | 2.523867158 | 3.196194291 | 0.066131979 | -0.585035415 | 0.109952314 | 0.340719133 | -0.235971053 | -0.244316282 |
| CM008017.1_104971 | 1.456150799 | 0.229442469 | 0 | 0.042408889 | -2.66595541 | 0.315638314 | 2.123795642 | -23.79906972 | -0.542159769 |
| CM008018.1_110103 | 1.456150799 | 1.376654813 | 2.324504939 | 0.12274424 | -0.08099291 | 0.102537989 | 0.755756632 | 0.683091739 | 0.674763722 |
| CM008018.1_113821 | 2.440786101 | 1.201841504 | 0.608798913 | 0.066510741 | -1.022099221 | 0.147080773 | -0.981208962 | -1.99488154 | -2.003308183 |
| CM008018.1_117444 | 7.688476219 | 14.63842952 | 20.13602403 | 0.002209837 | 0.928991179 | 0.021182723 | 0.460018061 | 1.39733321 | 1.38900924 |
| CM008018.1_119020 | 1.525491313 | 1.922946406 | 1.217597825 | 0.111252526 | 0.33404459 | 0.125444122 | -0.659280867 | -0.316969824 | -0.325236278 |
| CM008018.1_120835 | 0.58246032 | 0.688327407 | 1.162252469 | 0.163565675 | 0.240935185 | 0.151642571 | 0.755756632 | 1.005057116 | 0.996691817 |
| CM008019.1_128870 | 1.747380959 | 1.606097282 | 3.196194291 | 0.115094418 | -0.121634894 | 0.062369894 | 0.992795829 | 0.879499829 | 0.871160935 |
| CM008019.1_130001 | 0.610196525 | 1.682578105 | 0 | 0.073694749 | 1.463327607 | 0.022246347 | -0.750673476 | -22.47704278 | 0.71265413 |
| CM008019.1_130171 | 1.164920639 | 1.606097282 | 0.581126235 | 0.11418857 | 0.463327607 | 0.095458439 | -1.466635789 | -0.994943316 | -1.003308183 |
| CM008019.1_131018 | 0.29123016 | 0 | 0.871689352 | 0.207164791 | 1.779768326 | 0.07590956 | -0.198114008 | 1.590060659 | 1.581654318 |
| CM008019.1_131447 | 11.06674607 | 17.66707011 | 6.392388582 | 0.004174901 | 0.674831712 | 1.31E-05 | -1.466635789 | -0.783483235 | -0.791804078 |
| CM008019.1_131998 | 3.356080889 | 1.201841504 | 0.913198369 | 0.024969589 | -1.481530839 | 0.168263069 | -0.396246461 | -1.869460819 | -1.877777301 |
| CM008019.1_134217 | 0.873690479 | 1.376654813 | 0.581126235 | 0.115981432 | 0.655972684 | 0.121908424 | -1.244243368 | -0.579782262 | -0.588270684 |
| CM008019.1_137729 | 1.392839895 | 4.389334188 | 1.111719753 | 0.00762782 | 1.655972684 | 0.006063344 | -1.981208962 | -0.316969824 | -0.325236278 |
| CM008019.1_138018 | 1.164920639 | 0 | 1.452815587 | 0.030942207 | -0.220231674 | 0.022290674 | 0.538851586 | 0.326935531 | 0.318619912 |
| CM008019.1_138754 | 1.164920639 | 0 | 1.162252469 | 0.030942207 | -0.220231674 | 0.041496984 | 0.216923491 | 0.004933561 | -0.003308183 |
| CM008020.1_141593 | 0.58246032 | 1.606097282 | 1.743378704 | 0.073694749 | 1.463327607 | 0.143607107 | 0.118326711 | 1.590060659 | 1.581654318 |
| CM008020.1_143049 | 1.456150799 | 0.458884938 | 0.290563117 | 0.071126842 | -1.66595541 | 0.247312928 | -0.659280867 | -2.317092958 | -2.325236278 |
| CM008020.1_143067 | 0.320353176 | 1.514320295 | 1.598097145 | 0.062662052 | 2.240935185 | 0.153188595 | 0.077684727 | 2.327059086 | 2.318619912 |
| CM008020.1_143069 | 3.050982627 | 9.133995429 | 7.305586951 | 0.000539327 | 1.581972103 | 0.065065389 | -0.32224588 | 1.268033719 | 1.259726223 |
| CM008020.1_143075 | 27.08440486 | 11.70156591 | 18.88660263 | 0 | -1.210760785 | 0.005014805 | 0.690661604 | -0.511764714 | -0.520099181 |
| CM008020.1_143606 | 11.28863572 | 13.94136144 | 15.21997281 | 0.037438676 | 0.304500314 | 0.064160508 | 0.126594327 | 0.43942412 | 0.431094641 |
| CM008020.1_145771 | 3.203531758 | 1.682578105 | 3.196194291 | 0.048314055 | -0.928989816 | 0.061240132 | 0.925681633 | 0.005015787 | -0.003308183 |
| CM008020.1_145882 | 0 | 0 | 1.111719753 | -- | 0 | 0.041496984 | 0.152793154 | 23.4820999 | 0.152793154 |
| CM008020.1_147241 | 0.29123016 | 1.147212345 | 2.033941822 | 0.085038376 | 1.977900779 | 0.108711 | 0.82614596 | 2.812429628 | 2.804046739 |
| CM008020.1_147456 | 14.85273815 | 13.3076632 | 9.007456638 | 0.0482858 | -0.158471662 | 0.022904296 | -0.563065552 | -0.713197808 | -0.721537214 |
| CM008021.1_147678 | 11.64920639 | 11.70156591 | 19.17716575 | 0.057874286 | 0.006469932 | 0.004103212 | 0.71268791 | 0.727490048 | 0.719157842 |
| CM008021.1_148608 | 3.621383726 | 5.925601154 | 1.66757963 | 0.033536448 | 0.710420468 | 0.002450734 | -1.829205869 | -1.110455095 | -1.1187854 |
| CM008021.1_148609 | 4.077222237 | 2.982752096 | 3.777320526 | 0.070884478 | -0.450942519 | 0.101515793 | 0.340719133 | -0.101900278 | -0.110223387 |
| CM008021.1_149700 | 34.47610368 | 43.74703074 | 36.2235353 | 0.005759376 | 0.343588362 | 0.017778665 | -0.272257744 | 0.079663985 | 0.071330618 |
| CM008021.1_150571 | 12.52289687 | 2.753309627 | 3.486757408 | 0 | -2.18532957 | 0.105865111 | 0.340719133 | -1.836273058 | -1.844610437 |
| CM008021.1_151843 | 12.52289687 | 11.70156591 | 12.78477716 | 0.054933391 | -0.097866728 | 0.067258608 | 0.127725409 | 0.038199791 | 0.029858681 |
| CM008021.1_154122 | 35.23884934 | 60.80225426 | 24.40730186 | 0 | 0.786957997 | 0 | -1.316811994 | -0.521515315 | -0.529853997 |
| CM008021.1_154130 | 0.29123016 | 0.458884938 | 0.290563117 | 0.195628342 | 0.655972684 | 0.247312928 | -0.659280867 | 0.004933982 | -0.003308183 |
| CM008022.1_158829 | 0.640706352 | 0 | 0.319619429 | 0.10800582 | 0.642264802 | 0.26087974 | -1.645572985 | -0.995066018 | -1.003308183 |
| CM008022.1_159134 | 18.19606039 | 6.259190552 | 14.57464597 | 0 | -1.539578125 | 0.000136621 | 1.219412837 | -0.311835518 | -0.320165288 |
| CM008022.1_159625 | 1.164920639 | 3.441637034 | 1.162252469 | 0.019979853 | 1.56286328 | 0.028533535 | -1.566171463 | 0.004933561 | -0.003308183 |
| CM008022.1_160011 | 64.07063516 | 95.67750954 | 91.81794509 | 0 | 0.578516544 | 0.030141425 | -0.059403693 | 0.527449214 | 0.519112852 |
| CM008022.1_160361 | 0.873690479 | 1.606097282 | 1.452815587 | 0.098534335 | 0.878365106 | 0.148131301 | -0.144707694 | 0.742096585 | 0.733657411 |
| CM008022.1_164169 | 3.203531758 | 2.294424689 | 3.196194291 | 0.08002175 | -0.481530839 | 0.101505962 | 0.478222656 | 0.005023223 | -0.003308183 |
| CM008022.1_165248 | 0.873690479 | 0.458884938 | 0.871689352 | 0.13744665 | -0.928989816 | 0.172123693 | 0.925681633 | 0.005098158 | -0.003308183 |
| CM008022.1_166506 | 0.961059527 | 5.804894463 | 2.876574862 | 0.000425916 | 2.59457214 | 0.03185032 | -1.012917822 | 1.590060659 | 1.581654318 |
| CM008022.1_167031 | 0 | 0.480736602 | 0 | 0.152468748 | -1.056681446 | 0.193791884 | 1.056681446 | -- | 0 |
| CM008022.1_167390 | 0.835703937 | 1.097333547 | 1.945509568 | 0.134856116 | 0.392938279 | 0.108711 | 0.82614596 | 1.227467127 | 1.219084239 |
| CM008022.1_171045 | 6.964199474 | 0.658400128 | 1.66757963 | 0 | -3.402921005 | 0.08996163 | 1.340719133 | -2.053874608 | -2.062201872 |
| CM008022.1_171200 | 0.915294788 | 1.201841504 | 0 | 0.134856116 | 0.392938279 | 0.053049451 | -0.265246649 | -23.06200528 | 0.127691629 |
| CM008023.1_171704 | 0 | 0.229442469 | 0.290563117 | 0.25085307 | -2.123795642 | 0.286090674 | 0.340719133 | 21.48197676 | -1.783076509 |
| CM008023.1_172014 | 1.392839895 | 0.219466709 | 0.555859877 | 0.042408889 | -2.66595541 | 0.200950336 | 1.340719133 | -1.316846701 | -1.325236278 |
| CM008023.1_173900 | 0.29123016 | 0.229442469 | 0.581126235 | 0.227853908 | -0.344027316 | 0.200950336 | 1.340719133 | 1.005180239 | 0.996691817 |
| CM008023.1_177547 | 0 | 0.917769876 | 2.615068056 | 0.064085535 | -0.123795642 | 0.045246099 | 1.510644134 | 24.65206594 | 1.386848493 |
| CM008024.1_180123 | 6.101965253 | 10.33583693 | 9.131983688 | 0.012842067 | 0.760309344 | 0.072907941 | -0.178655026 | 0.589986524 | 0.581654318 |
| CM008024.1_183606 | 10.77551591 | 5.277176785 | 4.939572995 | 0.002952352 | -1.029918725 | 0.098252396 | -0.095379982 | -1.116962855 | -1.125298707 |
| CM008025.1_184228 | 18.19606039 | 6.259190552 | 14.83034151 | 0 | -1.539578125 | 9.85E-05 | 1.244503817 | -0.286738876 | -0.295074307 |
| CM008025.1_184735 | 0.610196525 | 1.201841504 | 3.652793475 | 0.116762733 | 0.977900779 | 0.023558665 | 1.603753539 | 2.590060659 | 2.581654318 |
| CM008025.1_185853 | 1.456150799 | 0.917769876 | 0.871689352 | 0.112137039 | -0.66595541 | 0.185895435 | -0.074318367 | -0.731966281 | -0.740273777 |
| CM008025.1_186278 | 0 | 0.480736602 | 0 | 0.152468748 | -1.056681446 | 0.193791884 | 1.056681446 | -- | 0 |
| CM008025.1_186565 | 0.58246032 | 0 | 0 | 0.10800582 | 0.779768326 | -- | 0 | -22.47704278 | 0.779768326 |
| CM008025.1_188371 | 0.58246032 | 1.147212345 | 0.871689352 | 0.116762733 | 0.977900779 | 0.168263069 | -0.396246461 | 0.590060659 | 0.581654318 |
| CM008025.1_188962 | 134.5483338 | 125.5623911 | 144.0950926 | 0.013325542 | -0.099720118 | 0.003329828 | 0.198616795 | 0.10723183 | 0.098896678 |
| CM008025.1_192294 | 0.58246032 | 1.376654813 | 0.290563117 | 0.095170799 | 1.240935185 | 0.080863586 | -2.244243368 | -0.995066018 | -1.003308183 |
| CM008025.1_193290 | 8.542751354 | 9.37436373 | 3.652793475 | 0.061555222 | 0.134019981 | 0.002413301 | -1.359720585 | -1.217364868 | -1.225700604 |
| CM008025.1_195252 | 1.747380959 | 0.458884938 | 0.581126235 | 0.047739679 | -1.928989816 | 0.221716937 | 0.340719133 | -1.579864633 | -1.588270684 |
| CM008026.1_198184 | 14.76410288 | 21.28827081 | 13.89649692 | 0.005903388 | 0.527965072 | 0.005294903 | -0.61533752 | -0.079037424 | -0.087372448 |
| CM008026.1_201442 | 0 | 0.757160147 | 2.876574862 | 0.09860269 | -0.401329617 | 0.02941626 | 1.925681633 | 24.65206594 | 1.524352016 |
| CM008026.1_202979 | 0.610196525 | 1.442209805 | 0 | 0.095170799 | 1.240935185 | 0.035004543 | -0.528281055 | -22.47704278 | 0.71265413 |
| CM008026.1_208845 | 0.58246032 | 0.688327407 | 0.581126235 | 0.163565675 | 0.240935185 | 0.207850689 | -0.244243368 | 0.005180239 | -0.003308183 |
| CM008027.1_209108 | 1.671407874 | 0.658400128 | 2.223439507 | 0.07063155 | -1.344027316 | 0.043987577 | 1.755756632 | 0.420073807 | 0.411729316 |
| CM008027.1_210382 | 0.873690479 | 2.06498222 | 1.452815587 | 0.065479877 | 1.240935185 | 0.126492745 | -0.507277774 | 0.742096585 | 0.733657411 |
| CM008027.1_210664 | 0.320353176 | 0.252386716 | 0.319619429 | 0.227853908 | -0.344027316 | 0.286090674 | 0.340719133 | 0.004933982 | -0.003308183 |
| CM008027.1_214124 | 1.164920639 | 0.917769876 | 0.290563117 | 0.134719781 | -0.344027316 | 0.143485591 | -1.659280867 | -1.995189573 | -2.003308183 |
| CM008027.1_214244 | 25.04579374 | 16.29041529 | 14.81871899 | 0.002158757 | -0.620544951 | 0.06004178 | -0.136602645 | -0.748815964 | -0.757147596 |
| CM008027.1_215433 | 0 | 0 | 1.452815587 | -- | 0 | 0.022290674 | 0.538851586 | 23.80410187 | 0.538851586 |
| CM008027.1_215935 | 31.73021932 | 68.02422912 | 46.87751627 | 0 | 1.100191209 | 9.34E-05 | -0.537152569 | 0.571371077 | 0.56303864 |
| CM008027.1_215941 | 1.456150799 | 2.06498222 | 1.452815587 | 0.099190719 | 0.503969591 | 0.126492745 | -0.507277774 | 0.005032146 | -0.003308183 |
| CM008027.1_216002 | 1.220393051 | 0.480736602 | 1.521997281 | 0.099921766 | -1.344027316 | 0.090463571 | 1.662647228 | 0.326935531 | 0.318619912 |
| CM008027.1_219971 | 0.29123016 | 2.06498222 | 0.290563117 | 0.019873664 | 2.825897686 | 0.028320684 | -2.829205869 | 0.004933982 | -0.003308183 |
| CM008028.1_228985 | 0 | 0.280429684 | 0.355132699 | 0.25085307 | -1.834289024 | 0.286090674 | 0.340719133 | 21.48197676 | -1.493569892 |
| CM008028.1_229104 | 0.58246032 | 0.688327407 | 1.162252469 | 0.163565675 | 0.240935185 | 0.151642571 | 0.755756632 | 1.005057116 | 0.996691817 |
| CM008028.1_232670 | 2.038611119 | 0.688327407 | 2.033941822 | 0.04988742 | -1.566419737 | 0.063436721 | 1.563111554 | 0.005004101 | -0.003308183 |
| CM008028.1_233163 | 0.320353176 | 0.252386716 | 0 | 0.227853908 | -0.344027316 | 0.315638314 | 1.986292118 | -21.47704278 | 1.642264802 |
| CM008028.1_233164 | 0.320353176 | 0.252386716 | 0 | 0.227853908 | -0.344027316 | 0.315638314 | 1.986292118 | -21.47704278 | 1.642264802 |
| CM008028.1_234298 | 3.785992078 | 1.376654813 | 3.777320526 | 0.017833138 | -1.459504533 | 0.025244278 | 1.45619635 | 0.005009495 | -0.003308183 |
| CM008029.1_236542 | 4.368452397 | 3.441637034 | 2.033941822 | 0.073551649 | -0.344027316 | 0.077684599 | -0.758816541 | -1.094526865 | -1.102843856 |
| CM008029.1_236567 | 6.115833356 | 2.982752096 | 7.264077934 | 0.015268727 | -1.03590502 | 0.006372654 | 1.284135604 | 0.256579454 | 0.248230584 |
| CM008029.1_238965 | 10.19305559 | 5.047734316 | 4.35844676 | 0.003973292 | -1.013878714 | 0.095910663 | -0.21182189 | -1.217364868 | -1.225700604 |
| CM008029.1_241215 | 34.36515886 | 37.6285649 | 40.38827331 | 0.028402273 | 0.13088164 | 0.041795129 | 0.102108201 | 0.241323153 | 0.232989841 |
| CM008029.1_242977 | 0.873690479 | 0.458884938 | 0 | 0.13744665 | -0.928989816 | 0.193791884 | 1.123795642 | -23.06200528 | 0.194805825 |
| CM008030.1_244854 | 2.78567979 | 2.194667094 | 0.833789815 | 0.090180695 | -0.344027316 | 0.065634915 | -1.396246461 | -1.731966281 | -1.740273777 |
| CM008030.1_244939 | 0.29123016 | 1.147212345 | 1.452815587 | 0.085038376 | 1.977900779 | 0.15082115 | 0.340719133 | 2.327059086 | 2.318619912 |
| CM008030.1_245504 | 0.58246032 | 0.229442469 | 2.324504939 | 0.158964182 | -1.344027316 | 0.011762877 | 3.340719133 | 2.005118679 | 1.996691817 |
| CM008030.1_246513 | 0.305098263 | 0.961473203 | 0.913198369 | 0.114580875 | 1.655972684 | 0.185895435 | -0.074318367 | 1.590060659 | 1.581654318 |
| CM008030.1_248613 | 0.961059527 | 1.261933579 | 0 | 0.134856116 | 0.392938279 | 0.053049451 | -0.335635977 | -23.06200528 | 0.057302302 |
| CM008030.1_248867 | 1.164920639 | 0.688327407 | 0.871689352 | 0.121667271 | -0.759064815 | 0.188804199 | 0.340719133 | -0.410062897 | -0.418345682 |
| CM008030.1_250130 | 1.220393051 | 1.682578105 | 0.913198369 | 0.11418857 | 0.463327607 | 0.126084122 | -0.881673289 | -0.410062897 | -0.418345682 |
| CM008030.1_251217 | 0.873690479 | 0.458884938 | 1.162252469 | 0.13744665 | -0.928989816 | 0.126051039 | 1.340719133 | 0.420094615 | 0.411729316 |
| CM008030.1_251835 | 0.29123016 | 0.688327407 | 0.290563117 | 0.152183516 | 1.240935185 | 0.192756666 | -1.244243368 | 0.004933982 | -0.003308183 |
| CM008030.1_255444 | 2.507111811 | 4.389334188 | 1.66757963 | 0.041464278 | 0.807975778 | 0.020279502 | -1.396246461 | -0.579919257 | -0.588270684 |
| CM008031.1_259110 | 0.29123016 | 0.458884938 | 0.581126235 | 0.195628342 | 0.655972684 | 0.221716937 | 0.340719133 | 1.005180239 | 0.996691817 |
| CM008031.1_262970 | 2.329841278 | 1.376654813 | 0.581126235 | 0.080866784 | -0.759064815 | 0.121908424 | -1.244243368 | -1.99488154 | -2.003308183 |
| CM008031.1_263955 | 0.873690479 | 0.229442469 | 0.290563117 | 0.107210574 | -1.928989816 | 0.286090674 | 0.340719133 | -1.580028518 | -1.588270684 |
| CM008032.1_269285 | 2.912301598 | 0.458884938 | 0.871689352 | 0.006012349 | -2.66595541 | 0.172123693 | 0.925681633 | -1.731966281 | -1.740273777 |
| CM008032.1_270582 | 2.621071438 | 1.147212345 | 1.743378704 | 0.050325325 | -1.192024222 | 0.12955274 | 0.603753539 | -0.579919257 | -0.588270684 |
| CM008033.1_275385 | 3.785992078 | 5.506619254 | 2.615068056 | 0.051530232 | 0.540495467 | 0.0261225 | -1.074318367 | -0.525492594 | -0.5338229 |
| CM008033.1_276646 | 0.29123016 | 0.917769876 | 8.426330403 | 0.114580875 | 1.655972684 | 0 | 3.198700128 | 4.863084814 | 4.854672812 |
| CM008034.1_282887 | 4.950912717 | 6.424389129 | 4.067883643 | 0.057197911 | 0.375864765 | 0.0467748 | -0.659280867 | -0.275085252 | -0.283416102 |
| CM008034.1_284828 | 3.064247768 | 6.145067863 | 79.7658923 | 0.015025901 | 1.003895988 | 0 | 3.698271137 | 4.710487611 | 4.702167125 |
| CM008034.1_285336 | 0.557135958 | 1.316800256 | 0.277929938 | 0.095170799 | 1.240935185 | 0.080863586 | -2.244243368 | -0.995066018 | -1.003308183 |
| CM008035.1_291664 | 0.29123016 | 0.688327407 | 0.871689352 | 0.152183516 | 1.240935185 | 0.188804199 | 0.340719133 | 1.590060659 | 1.581654318 |
| CM008036.1_292007 | 0.58246032 | 0.458884938 | 1.162252469 | 0.174900687 | -0.344027316 | 0.126051039 | 1.340719133 | 1.005057116 | 0.996691817 |
| CM008036.1_292916 | 10.77551591 | 13.76654813 | 9.87914599 | 0.031840717 | 0.353409914 | 0.02966368 | -0.478708622 | -0.116962855 | -0.125298707 |
| CM008036.1_294342 | 0.557135958 | 0.658400128 | 0.555859877 | 0.163565675 | 0.240935185 | 0.207850689 | -0.244243368 | 0.005180239 | -0.003308183 |
| CM008036.1_295794 | 1.164920639 | 0.229442469 | 2.033941822 | 0.069364477 | -2.344027316 | 0.020058545 | 3.148074055 | 0.812306072 | 0.804046739 |
| CM008037.1_299589 | 341.2457742 | 337.3203323 | 315.45048 | 0.013969655 | -0.0166919 | 0.005783413 | -0.096705735 | -0.105062606 | -0.113397635 |
| CM008037.1_300926 | 0.278567979 | 0.658400128 | 0.833789815 | 0.152183516 | 1.240935185 | 0.188804199 | 0.340719133 | 1.590060659 | 1.581654318 |
| CM008037.1_301443 | 0 | 0.280429684 | 0.355132699 | 0.25085307 | -1.834289024 | 0.286090674 | 0.340719133 | 21.48197676 | -1.493569892 |
| CM008037.1_302471 | 2.621071438 | 1.147212345 | 1.743378704 | 0.050325325 | -1.192024222 | 0.12955274 | 0.603753539 | -0.579919257 | -0.588270684 |
| CM008037.1_302513 | 1.671407874 | 18.6546703 | 175.3737911 | 0 | 3.48040112 | 0 | 3.232824392 | 6.721553123 | 6.713225511 |
| CM008038.1_306877 | 6.989523835 | 3.212194565 | 6.101825465 | 0.008528135 | -1.121634894 | 0.028350416 | 0.925681633 | -0.187629291 | -0.195953261 |
| CM008038.1_307359 | 3.494761918 | 2.06498222 | 1.162252469 | 0.059371196 | -0.759064815 | 0.109511215 | -0.829205869 | -1.579987756 | -1.588270684 |
| CM008038.1_307839 | 2.228543832 | 0 | 0 | 0.001655734 | -1.156101337 | -- | 0 | -24.47710456 | -1.156101337 |
| CM008038.1_309862 | 1.220393051 | 0 | 0.608798913 | 0.030942207 | -0.28734587 | 0.13358663 | -0.715962313 | -0.994943316 | -1.003308183 |
| CM008038.1_311195 | 1.114271916 | 1.097333547 | 0 | 0.135007553 | -0.022099221 | 0.053049451 | -0.134002116 | -23.47716634 | -0.156101337 |
| CM008039.1_313741 | 4.983271623 | 7.29117179 | 14.20530796 | 0.047374878 | 0.549057481 | 0.004988594 | 0.962207509 | 1.519608935 | 1.51126499 |
| CM008039.1_315920 | 0 | 3.12478791 | 0 | 0.000620424 | 1.643758272 | 0.001174544 | -1.643758272 | -- | 0 |
| CM008040.1_317341 | 7.521335432 | 11.6317356 | 2.779299383 | 0.013871862 | 0.629005637 | 6.66E-06 | -2.065273227 | -1.427950885 | -1.43626759 |
| CM008040.1_317809 | 0.873690479 | 0.458884938 | 0 | 0.13744665 | -0.928989816 | 0.193791884 | 1.123795642 | -23.06200528 | 0.194805825 |
| CM008040.1_322358 | 4.368452397 | 4.12996444 | 3.486757408 | 0.082920266 | -0.08099291 | 0.103077346 | -0.244243368 | -0.316895834 | -0.325236278 |
| CM008040.1_323630 | 1.456150799 | 2.523867158 | 1.743378704 | 0.074343502 | 0.793476208 | 0.11343125 | -0.533749985 | 0.268033719 | 0.259726223 |
| CM008041.1_325871 | 0.873690479 | 1.147212345 | 1.162252469 | 0.134856116 | 0.392938279 | 0.167574911 | 0.018791038 | 0.420094615 | 0.411729316 |
| CM008041.1_326111 | 0.58246032 | 1.376654813 | 0.290563117 | 0.095170799 | 1.240935185 | 0.080863586 | -2.244243368 | -0.995066018 | -1.003308183 |
| CM008041.1_326329 | 0.800882939 | 1.682578105 | 0.532699048 | 0.081065193 | 1.071010184 | 0.073272883 | -1.659280867 | -0.579782262 | -0.588270684 |
| CM008041.1_326625 | 1.456150799 | 3.441637034 | 1.162252469 | 0.03274672 | 1.240935185 | 0.028533535 | -1.566171463 | -0.316969824 | -0.325236278 |
| CM008041.1_329257 | 1.392839895 | 1.536266966 | 1.945509568 | 0.118302648 | 0.141399512 | 0.131350463 | 0.340719133 | 0.490402688 | 0.482118644 |
| CM008041.1_329491 | 1.671407874 | 18.6546703 | 175.3737911 | 0 | 3.48040112 | 0 | 3.232824392 | 6.721553123 | 6.713225511 |
| CM008041.1_329711 | 0 | 0.458884938 | 0.581126235 | 0.152468748 | -1.123795642 | 0.221716937 | 0.340719133 | 22.48222302 | -0.783076509 |
| CM008041.1_332283 | 23.8808731 | 18.81428245 | 11.04139846 | 0.018294592 | -0.344027316 | 0.003032963 | -0.768905358 | -1.104594339 | -1.112932674 |
| CM008041.1_334231 | 0.29123016 | 0 | 0.871689352 | 0.207164791 | 1.779768326 | 0.07590956 | -0.198114008 | 1.590060659 | 1.581654318 |
| CM008041.1_334512 | 104.4351353 | 69.40634684 | 84.37952928 | 0 | -0.589467661 | 0.00497913 | 0.281825444 | -0.299306432 | -0.307642218 |
| CM008041.1_334666 | 0 | 0.280429684 | 0.355132699 | 0.25085307 | -1.834289024 | 0.286090674 | 0.340719133 | 21.48197676 | -1.493569892 |
| MKHE01000127.1_335921 | 0 | 1.051611316 | 0.266349524 | 0.041163135 | 0.072601571 | 0.109522678 | -1.981208962 | 21.48197676 | -1.908607391 |
| MKHE01004964.1_338157 | 3.203531758 | 6.309667895 | 1.917716575 | 0.020433414 | 0.977900779 | 0.004748836 | -1.718174556 | -0.731966281 | -0.740273777 |
| MKHE01008022.1_338752 | 3.050982627 | 4.566997714 | 5.479190213 | 0.059801801 | 0.581972103 | 0.091670979 | 0.262716621 | 0.85299622 | 0.844688724 |
| MKHE01008352.1_338794 | 4.62732365 | 3.084726526 | 5.326990485 | 0.066131979 | -0.585035415 | 0.057769551 | 0.78817811 | 0.211473 | 0.203142695 |
| xtr-miR-92b | 9.458046142 | 6.970680722 | 5.174790757 | 0.040543216 | -0.440242631 | 0.066702316 | -0.429799021 | -0.861705369 | -0.870041652 |
