# Supplementary material for: RNA sequencing-based identification of microRNAs in the antler cartilage of Gansu red deer (Cervus elaphus kansuensis)
Source: PeerJ. 2022 Sep 21;10:e13947. doi: 10.7717/peerj.13947 (PMC9508884; doi:10.7717/peerj.13947)
Supplement: Table S5 [file peerj-10-13947-s006.docx]

Additional file Table S5 All 243 differentially expressed miRNAs in 30 d, 60 d and 90 d antler cartilage.

|  | ID | Sequence | Length | 30 d | 60 d | 90 d | 30 d_vs_60 d | 30 d_vs_90 d | 60 d_vs_90 d |
| --- | --- | --- | --- | --- | --- | --- | --- | --- | --- |
| 1 | aae-miR-100 | AACCCGUAGAUCCGAACUUGUG | 22 | 1176.71 | 2464.893 | 2504.586 | up | up | normal |
| 2 | aae-miR-125-5p | UCCCUGAGACCCUAACUUGUGA | 22 | 284.9091 | 859.3183 | 545.4431 | up | normal | normal |
| 3 | aae-miR-7 | UGGAAGACUAGUGAUUUUGUUGU | 23 | 1257.278 | 559.7501 | 726.1834 | down | normal | normal |
| 4 | aca-let-7c-1-3p | CUGUACAACCUUCUAGCUUUCC | 22 | 1.459575 | 6.7188a54 | 6.151614 | up | up | normal |
| 5 | aca-let-7c-2-3p | CUGUACAGCCUCCUAGCUUUCC | 22 | 0 | 2.780216 | 1.171736 | up | normal | normal |
| 6 | aca-let-7c-5p | UGAGGUAGUAGGUUGUAUGGUU | 22 | 1360.908 | 4193.26 | 2557.607 | up | normal | normal |
| 7 | aca-let-7i-3p | CUGCGCAAGCUACUGCCUUGCUA | 23 | 1.459575 | 4.865377 | 4.101076 | up | normal | normal |
| 8 | aca-miR-10a-5p | UACCCUGUAGAUCCGAAUUUGUG | 23 | 6.422131 | 18.99814 | 4.979878 | up | normal | down |
| 9 | aca-miR-129a-5p | CUUUUUGCGGUCUGGGCUUGC | 21 | 2.043405 | 2.085162 | 11.13149 | normal | up | up |
| 10 | aca-miR-138-5p | AGCUGGUGUUGUGAAUCAGGCC | 22 | 3.211066 | 2.780216 | 9.666823 | normal | up | up |
| 11 | aca-miR-144-3p | UACAGUAUAGAUGAUGUACU | 20 | 1.16766 | 11.81592 | 4.39401 | up | up | down |
| 12 | aca-miR-144-5p | GGAUAUCAUCAUAUACUGUAA | 21 | 1.75149 | 12.51097 | 4.979878 | up | normal | down |
| 13 | aca-miR-146a-5p | UGAGAACUGAAUUCCAUAGGC | 21 | 1.459575 | 7.645593 | 1.46467 | up | normal | down |
| 14 | aca-miR-150-5p | UCUCCCAACCCUUGUACCAGUG | 22 | 3.502981 | 10.65749 | 6.737482 | up | normal | normal |
| 15 | aca-miR-16a-5p | UAGCAGCACGUAAAUAUUGG | 20 | 4.086811 | 9.035701 | 7.909219 | up | normal | normal |
| 16 | aca-miR-182-5p | UUUGGCAAUGGUAGAACUCACA | 22 | 4.086811 | 1.390108 | 5.272812 | normal | normal | up |
| 17 | aca-miR-184-3p | UGGACGGAGAACUGAUAAGGGU | 22 | 25.39661 | 23.63183 | 53.02106 | normal | up | up |
| 18 | aca-miR-18a-5p | UAAGGUGCAUCUAGUGCAGAUAG | 23 | 13.72001 | 6.950539 | 6.151614 | normal | down | normal |
| 19 | aca-miR-19a-3p | UGUGCAAAUCUAUGCAAAACUG | 22 | 32.11066 | 10.88918 | 14.06083 | down | down | normal |
| 20 | aca-miR-200a-3p | UAACACUGUCUGGUAACGAUGU | 22 | 2.627236 | 0.926739 | 7.030417 | normal | up | up |
| 21 | aca-miR-200b-3p | UAAUACUGCCUGGUAAUGAUGA | 22 | 2.33532 | 0.695054 | 5.85868 | normal | normal | up |
| 22 | aca-miR-204a-3p | GCUGGGAAGGCAAAGGGACGU | 21 | 1.16766 | 6.718854 | 4.101076 | up | normal | normal |
| 23 | aca-miR-204a-5p | UUCCCUUUGUCAUCCUAUGCCU | 22 | 35.02981 | 117.2324 | 72.64764 | up | up | normal |
| 24 | aca-miR-205a | UCCUUCAUUCCACCGGAGUCUG | 22 | 4.670641 | 2.780216 | 12.8891 | normal | up | up |
| 25 | aca-miR-27a-3p | UUCACAGUGGCUAAGUUCCGC | 21 | 255.7176 | 546.0807 | 553.3524 | up | up | normal |
| 26 | aca-miR-27b-5p | AGAGCUUAGCUGAUUGGUGAAC | 22 | 25.68853 | 8.572331 | 16.69724 | down | normal | normal |
| 27 | aca-miR-30a-3p | CUUUCAGUCGGAUGUUUGCAGC | 22 | 2.627236 | 7.645593 | 7.616285 | up | up | normal |
| 28 | aca-miR-30a-5p | UGUAAACAUCCUCGACUGGAAG | 22 | 200.5456 | 461.9792 | 402.1984 | up | up | normal |
| 29 | aca-miR-30b-5p | UGUAAACAUCCUACACUCAGCU | 22 | 93.70473 | 187.8962 | 193.6294 | up | up | normal |
| 30 | aca-miR-365-5p | AGGGACUUUCAGGGGCAGCUGUG | 23 | 4.670641 | 1.158423 | 3.222274 | down | normal | normal |
| 31 | aca-miR-451-5p | AAACCGUUACCAUUACUGAGUU | 22 | 979.9589 | 5150.813 | 2500.192 | up | up | down |
| 32 | aca-miR-99a-3p | CAAGCUCGCUUCUAUGGGUC | 20 | 2.043405 | 7.645593 | 5.565746 | up | normal | normal |
| 33 | aca-miR-99a-5p | AACCCGUAGAUCCGAUCUUGUG | 22 | 3856.49 | 11467.69 | 6584.864 | up | normal | normal |
| 34 | aga-miR-184 | UGGACGGAGAACUGAUAAGGG | 21 | 0.291915 | 4.865377 | 0.585868 | up | normal | down |
| 35 | age-miR-18 | UAAGGUGCAUCUAGUGCAGAUA | 22 | 13.72001 | 6.950539 | 6.151614 | normal | down | normal |
| 36 | age-miR-19a | UGUGCAAAUCUAUGCAAAACUGA | 23 | 32.11066 | 10.88918 | 14.06083 | down | down | normal |
| 37 | age-miR-22 | AAGCUGCCAGUUGAAGAACUGU | 22 | 128.7345 | 288.9107 | 236.3978 | up | normal | normal |
| 38 | age-miR-27a | UUCACAGUGGCUAAGUUCCGCC | 22 | 275.2759 | 566.7006 | 580.8882 | up | up | normal |
| 39 | age-miR-30b | UGUAAACAUCCUACACUCAGC | 21 | 93.70473 | 187.8962 | 193.6294 | up | up | normal |
| 40 | aja-let-7i | CUGCGCAAGCUACUGCCUUGCU | 22 | 1.459575 | 4.865377 | 4.101076 | up | normal | normal |
| 41 | ame-let-7 | UGAGGUAGUAGGUUGUAUAGU | 21 | 250.755 | 712.4303 | 607.5452 | up | up | normal |
| 42 | api-miR-7 | UGGAAGACUAGUGAUUUUGUUGUU | 24 | 1257.278 | 559.7501 | 726.1834 | down | normal | normal |
| 43 | bfl-miR-183 | UAUGGCACUGGUAGAAUUCACU | 22 | 0.875745 | 0.463369 | 2.92934 | normal | normal | up |
| 44 | bta-miR-1246 | AAUGGAUUUUUGGAGCAGG | 19 | 8.757452 | 10.19412 | 21.97005 | normal | up | up |
| 45 | bta-miR-1260b | AUCCCACCACUGCCACCA | 18 | 9.341282 | 18.0714 | 28.4146 | normal | up | normal |
| 46 | bta-miR-129 | CUUUUUGCGGUCUGGGCUUGCU | 22 | 2.043405 | 2.085162 | 11.13149 | normal | up | up |
| 47 | bta-miR-129-3p | AAGCCCUUACCCCAAAAAGCAU | 22 | 0.291915 | 0 | 2.050538 | normal | normal | up |
| 48 | bta-miR-1298 | UUCAUUCGGCUGUCCAGAUGUA | 22 | 21.60171 | 101.4779 | 297.328 | up | up | up |
| 49 | bta-miR-146b | UGAGAACUGAAUUCCAUAGGCUGU | 24 | 19.55831 | 46.10524 | 14.06083 | up | normal | down |
| 50 | bta-miR-150 | UCUCCCAACCCUUGUACCAGUGU | 23 | 4.086811 | 12.0476 | 9.666823 | up | up | normal |
| 51 | bta-miR-152 | UCAGUGCAUGACAGAACUUGGG | 22 | 647.7595 | 1314.347 | 1400.81 | up | up | normal |
| 52 | bta-miR-182 | UUUGGCAAUGGUAGAACUCACACU | 24 | 4.086811 | 1.390108 | 5.272812 | normal | normal | up |
| 53 | bta-miR-187 | UCGUGUCUUGUGUUGCAGCCGG | 22 | 1.459575 | 5.328747 | 1.46467 | up | normal | down |
| 54 | bta-miR-199a-3p | ACAGUAGUCUGCACAUUGGUUA | 22 | 2678.029 | 6764.496 | 5027.334 | up | normal | normal |
| 55 | bta-miR-199c | UACAGUAGUCUGCACAUUGG | 20 | 7.005961 | 30.119 | 19.33365 | up | up | normal |
| 56 | bta-miR-200a | UAACACUGUCUGGUAACGAUGUU | 23 | 2.627236 | 0.926739 | 7.030417 | normal | up | up |
| 57 | bta-miR-200b | UAAUACUGCCUGGUAAUGAUG | 21 | 2.33532 | 0.695054 | 5.85868 | normal | normal | up |
| 58 | bta-miR-215 | AUGACCUAUGAAUUGACAGACA | 22 | 1.16766 | 4.170323 | 6.444548 | up | up | normal |
| 59 | bta-miR-22-3p | AAGCUGCCAGUUGAAGAACUG | 21 | 128.7345 | 288.9107 | 236.3978 | up | normal | normal |
| 60 | bta-miR-22-5p | AGUUCUUCAGUGGCAAGCUUUA | 22 | 15.76341 | 30.119 | 32.80861 | normal | up | normal |
| 61 | bta-miR-2332 | CGGUUUAAGGUCUUGGAGACAAAG | 24 | 4.086811 | 2.780216 | 11.13149 | normal | up | up |
| 62 | bta-miR-2419-5p | AUCGCAUCAACACUCGUCUGUU | 22 | 33.27832 | 93.13722 | 66.49602 | up | normal | normal |
| 63 | bta-miR-27a-3p | UUCACAGUGGCUAAGUUCCG | 20 | 275.2759 | 566.7006 | 580.8882 | up | up | normal |
| 64 | bta-miR-27a-5p | AGGGCUUAGCUGCUUGUGAGCA | 22 | 54.88003 | 26.41205 | 69.13243 | down | normal | up |
| 65 | bta-miR-30a-5p | UGUAAACAUCCUCGACUGGAAGCU | 24 | 200.5456 | 461.9792 | 402.1984 | up | up | normal |
| 66 | bta-miR-326 | CCUCUGGGCCCUUCCUCCAG | 20 | 0 | 2.085162 | 1.46467 | up | normal | normal |
| 67 | bta-miR-339b | UCCCUGUCCUCCAGGAGCUC | 20 | 4.086811 | 16.68129 | 13.47496 | up | up | normal |
| 68 | bta-miR-365-5p | AGGGACUUUUGGGGGCAGAUGUG | 23 | 25.98044 | 9.49907 | 16.11137 | down | normal | normal |
| 69 | bta-miR-380-3p | UAUGUAAUGUGGUCCACGUCU | 21 | 60.42642 | 141.3276 | 90.80955 | up | normal | normal |
| 70 | bta-miR-382 | GAAGUUGUUCGUGGUGGAUUCG | 22 | 70.64345 | 144.1078 | 83.19326 | up | normal | normal |
| 71 | bta-miR-411c-3p | UGUAUGUCAACUGAUCCACAGU | 22 | 39.11662 | 91.9788 | 59.46561 | up | normal | normal |
| 72 | bta-miR-424-5p | CAGCAGCAAUUCAUGUUUUGA | 21 | 124.0639 | 267.8274 | 140.0225 | up | normal | normal |
| 73 | bta-miR-431 | UGUCUUGCAGGCCGUCAUGCAGG | 23 | 20.43405 | 53.51915 | 36.03088 | up | normal | normal |
| 74 | bta-miR-451 | AAACCGUUACCAUUACUGAGUUU | 23 | 979.9589 | 5150.813 | 2500.192 | up | up | down |
| 75 | bta-miR-486 | UCCUGUACUGAGCUGCCCCGAG | 22 | 27.73193 | 188.5913 | 74.99111 | up | up | down |
| 76 | bta-miR-503-3p | GGAGUAUUGUUUCUGCUGCCCGG | 23 | 6.714046 | 10.19412 | 4.686944 | normal | normal | down |
| 77 | bta-miR-504 | AGACCCUGGUCUGCACUCUGUC | 22 | 0.291915 | 3.706954 | 0.585868 | up | normal | down |
| 78 | bta-miR-652 | AAUGGCGCCACUAGGGUUGUG | 21 | 5.254471 | 7.645593 | 13.18203 | normal | up | normal |
| 79 | bta-miR-665 | ACCAGUAGGCCGAGGCCCCU | 20 | 10.21703 | 18.0714 | 21.09125 | normal | up | normal |
| 80 | bta-miR-95 | UUCAACGGGUAUUUAUUGAGCA | 22 | 0.875745 | 6.48717 | 7.909219 | up | up | normal |
| 81 | bta-miR-99a-5p | AACCCGUAGAUCCGAUCUUGU | 21 | 3856.49 | 11467.69 | 6584.864 | up | normal | normal |
| 82 | ccr-miR-100 | AACCCGUAGAUCCGAACUUGU | 21 | 1188.678 | 2497.56 | 2533.293 | up | up | normal |
| 83 | ccr-miR-144 | CUACAGUAUAGAUGAUGUACU | 21 | 1.16766 | 11.81592 | 4.39401 | up | up | down |
| 84 | ccr-miR-182-5p | UUUGGCAAUGGUAGAACUCACAC | 23 | 4.086811 | 1.390108 | 5.272812 | normal | normal | up |
| 85 | ccr-miR-199-3p | ACAGUAGUCUGCACAUUGGUU | 21 | 2684.743 | 6771.447 | 5033.192 | up | normal | normal |
| 86 | ccr-miR-200a | UAACACUGUCUGGUAACGAUG | 21 | 2.627236 | 0.926739 | 7.030417 | normal | up | up |
| 87 | ccr-miR-214 | UACAGCAGGCACAGACAGG | 19 | 43.20343 | 91.28375 | 75.86991 | up | normal | normal |
| 88 | cfa-miR-10a | UACCCUGUAGAUCCGAAUUUGU | 22 | 6.422131 | 18.99814 | 4.979878 | up | normal | down |
| 89 | cfa-miR-146b | UGAGAACUGAAUUCCAUAGGCU | 22 | 19.55831 | 46.10524 | 14.06083 | up | normal | down |
| 90 | cfa-miR-152 | UCAGUGCAUGACAGAACUUGG | 21 | 627.6174 | 1249.012 | 1336.658 | normal | up | normal |
| 91 | cfa-miR-23a | AUCACAUUGCCAGGGAUUU | 19 | 2.043405 | 12.27929 | 12.59616 | up | up | normal |
| 92 | cfa-miR-23b | AUCACAUUGCCAGGGAUUA | 19 | 1.16766 | 5.792116 | 7.030417 | up | up | normal |
| 93 | cfa-miR-301b | CAGUGCAAUGAUAUUGUCAAAGC | 23 | 1.459575 | 3.938639 | 5.272812 | normal | up | normal |
| 94 | cfa-miR-30a | UGUAAACAUCCUCGACUGGAAGC | 23 | 200.5456 | 461.5158 | 401.9055 | up | up | normal |
| 95 | cfa-miR-329b | AACACACCUGGUUAACCUCUUU | 22 | 2.919151 | 7.413908 | 3.808142 | up | normal | normal |
| 96 | cfa-miR-486 | UCCUGUACUGAGCUGCCCCGA | 21 | 27.1481 | 173.5318 | 71.4759 | up | up | down |
| 97 | cfa-miR-493 | UGAAGGUCUACUGUGUGCCAG | 21 | 12.26043 | 30.58237 | 15.5255 | up | normal | normal |
| 98 | cfa-miR-652 | AAUGGCGCCACUAGGGUUGUGC | 22 | 5.254471 | 7.645593 | 13.18203 | normal | up | normal |
| 99 | cgr-let-7i | CUGCGCAAGCUACUGCCUUGC | 21 | 1.459575 | 4.865377 | 4.101076 | up | normal | normal |
| 100 | cgr-miR-125b-3p | UCACAAGUCAGGCUCUUGGGAC | 22 | 17.22299 | 49.11714 | 36.03088 | up | up | normal |
| 101 | cgr-miR-144 | GGAUAUCAUCAUAUACUGUAAG | 22 | 4.670641 | 28.03384 | 10.83856 | up | up | down |
| 102 | cgr-miR-146b-5p | UGAGAACUGAAUUCCAUAGGCUG | 23 | 19.55831 | 46.10524 | 14.06083 | up | normal | down |
| 103 | cgr-miR-181a-3p | ACCAUCGACCGUUGAUUGUACC | 22 | 41.16002 | 20.38825 | 42.47543 | down | normal | up |
| 104 | cgr-miR-187 | UCGUGUCUUGUGUUGCAGCCG | 21 | 1.459575 | 5.328747 | 1.46467 | up | normal | down |
| 105 | cgr-miR-200b | UAAUACUGCCUGGUAAUGAUGAC | 23 | 10.50894 | 3.0119 | 26.94993 | down | up | up |
| 106 | cgr-miR-205 | UCCUUCAUUCCACCGGAGU | 19 | 4.670641 | 2.780216 | 12.8891 | normal | up | up |
| 107 | cgr-miR-22-5p | AGUUCUUCAGUGGCAAGCUUU | 21 | 15.76341 | 30.119 | 32.80861 | normal | up | normal |
| 108 | cgr-miR-369-5p | AGAUCGACCGUGUUAUAUUCGC | 22 | 14.88767 | 33.36259 | 21.67712 | up | normal | normal |
| 109 | cgr-miR-99a-3p | CAAGCUCGCUUCUAUGGGUCUG | 22 | 2.043405 | 7.645593 | 5.565746 | up | normal | normal |
| 110 | chi-miR-125b-3p | ACAAGUCAGGCUCUUGGGACC | 21 | 16.93107 | 49.11714 | 35.73795 | up | up | normal |
| 111 | chi-miR-125b-5p | UCCCUGAGACCCUAACUUGU | 20 | 293.6666 | 886.6571 | 562.1404 | up | normal | normal |
| 112 | chi-miR-127-5p | GAAGCUCAGAGGGCUCUGAUUC | 22 | 23.64512 | 48.1904 | 36.90969 | up | normal | normal |
| 113 | chi-miR-136-3p | AUCAUCGUCUCAAAUGAGUCU | 21 | 76.77366 | 183.7259 | 119.81 | up | normal | normal |
| 114 | chi-miR-136-5p | ACUCCAUUUGUUUUGAUGAUGG | 22 | 26.85619 | 63.24991 | 31.92981 | up | normal | normal |
| 115 | chi-miR-144-3p | UACAGUAUAGAUGAUGUAC | 19 | 1.16766 | 11.81592 | 4.39401 | up | up | down |
| 116 | chi-miR-144-5p | UGGGAUAUCAUCAUAUACUGU | 21 | 4.670641 | 28.03384 | 10.54562 | up | up | down |
| 117 | chi-miR-148a-5p | AAAGUUCUGAGACACUCCGACU | 22 | 531.8692 | 247.4392 | 427.0978 | down | normal | normal |
| 118 | chi-miR-187 | UCGUGUCUUGUGUUGCAGCC | 20 | 1.459575 | 5.328747 | 1.46467 | up | normal | down |
| 119 | chi-miR-215-5p | AUGACCUAUGAAUUGACAGAC | 21 | 1.459575 | 4.170323 | 6.444548 | normal | up | normal |
| 120 | chi-miR-22-3p | AAGCUGCCAGUUGAAGAAC | 19 | 128.7345 | 288.9107 | 236.3978 | up | normal | normal |
| 121 | chi-miR-30a-3p | CUUUCAGUCGGAUGUUUGCAG | 21 | 2.627236 | 7.645593 | 7.616285 | up | up | normal |
| 122 | chi-miR-326-3p | CCUCUGGGCCCUUCCUCCAGC | 21 | 0 | 2.085162 | 1.46467 | up | normal | normal |
| 123 | chi-miR-329b-3p | AACACACCUGGUUAACCUCU | 20 | 2.919151 | 7.413908 | 3.808142 | up | normal | normal |
| 124 | chi-miR-369-5p | AGAUCGACCGUGUUAUAUUCG | 21 | 14.88767 | 33.36259 | 21.67712 | up | normal | normal |
| 125 | chi-miR-379-3p | UAUGUAACAUGGUCCACUAAC | 21 | 11.38469 | 23.40015 | 16.69724 | up | normal | normal |
| 126 | chi-miR-411b-3p | UAUGUCACAUGGUCCACUAAU | 21 | 0 | 2.085162 | 0 | up | -- | down |
| 127 | chi-miR-451-5p | AAACCGUUACCAUUACUGA | 19 | 970.6176 | 5114.67 | 2483.495 | up | up | down |
| 128 | chi-miR-502b-3p | AUCCACCUGGGCAAGGAUUCUGAA | 24 | 5.546386 | 3.243585 | 1.46467 | normal | down | normal |
| 129 | chi-miR-502b-5p | UAAUUCUUGCUCCCCAGGUGAG | 22 | 4.378726 | 3.706954 | 10.83856 | normal | up | up |
| 130 | chi-miR-504 | AGACCCUGGUCUGCACUCUGU | 21 | 0.291915 | 3.706954 | 0.585868 | up | normal | down |
| 131 | chi-miR-532-3p | CCUCCCACACCCAAGGCUUGC | 21 | 4.086811 | 10.65749 | 9.666823 | up | up | normal |
| 132 | chi-miR-543-5p | ACCUGUGGUGCUUAAGGAG | 19 | 0.291915 | 2.780216 | 1.46467 | up | normal | normal |
| 133 | chi-miR-99a-3p | CAAGCUCGCUUCUAUGGGUCUGU | 23 | 2.043405 | 7.645593 | 5.565746 | up | normal | normal |
| 134 | cin-miR-7-5p | UGGAAGACUAGUGAUUUUGUUG | 22 | 1257.278 | 559.7501 | 726.1834 | down | normal | normal |
| 135 | dre-miR-199-3p | UACAGUAGUCUGCACAUUGGUU | 22 | 2678.029 | 6764.496 | 5027.334 | up | normal | normal |
| 136 | dre-miR-27b-5p | AGAGCUUAGCUGAUUGGUGAACA | 23 | 25.68853 | 8.572331 | 16.69724 | down | normal | normal |
| 137 | dre-miR-301c-5p | GCUCUGACGAUGUUGCACUAC | 21 | 9.925112 | 1.853477 | 4.686944 | down | down | normal |
| 138 | dvi-miR-125-5p | UCCCUGAGACCCUAACUUGUG | 21 | 284.9091 | 859.3183 | 545.4431 | up | normal | normal |
| 139 | eca-miR-182 | UUUGGCAAUGGUAGAACUCACACUG | 25 | 4.086811 | 1.390108 | 5.272812 | normal | normal | up |
| 140 | eca-miR-1912 | UACCCAGAGCGUGCAGUGUGAA | 22 | 0.291915 | 1.158423 | 3.808142 | normal | up | normal |
| 141 | eca-miR-3959 | UGUAUGUCAACUGAUCCACAGUC | 23 | 39.11662 | 91.9788 | 59.46561 | up | normal | normal |
| 142 | eca-miR-424 | CAGCAGCAAUUCAUGUUUUGAA | 22 | 124.0639 | 267.8274 | 140.0225 | up | normal | normal |
| 143 | eca-miR-532-3p | CCUCCCACACCCAAGGCUUGCA | 22 | 4.086811 | 10.65749 | 9.666823 | up | up | normal |
| 144 | efu-miR-125b | UCCCUGAGACCCUAACUUGUGAGG | 24 | 293.6666 | 886.6571 | 562.1404 | up | normal | normal |
| 145 | efu-miR-128a | UCACAGUGAACCGGUCUCUUUC | 22 | 42.32768 | 75.29751 | 34.85915 | normal | normal | down |
| 146 | efu-miR-181a | AACCAUCGACCGUUGAUUGUACC | 23 | 41.16002 | 20.38825 | 42.47543 | down | normal | up |
| 147 | efu-miR-199 | UACAGUAGUCUGCACAUUGGUUA | 23 | 2678.029 | 6764.496 | 5027.334 | up | normal | normal |
| 148 | efu-miR-200a | UAACACUGUCUGGUAACGAUGUUC | 24 | 2.627236 | 0.926739 | 7.030417 | normal | up | up |
| 149 | efu-miR-200b | UAAUACUGCCUGGUAAUGAUGACG | 24 | 10.50894 | 3.0119 | 26.94993 | down | up | up |
| 150 | efu-miR-205 | UCCUUCAUUCCACCGGAGUCUGU | 23 | 4.670641 | 2.780216 | 12.8891 | normal | up | up |
| 151 | efu-miR-503 | UGGAGUAUUGUUUCUGCUGCCCGG | 24 | 6.714046 | 10.42581 | 4.979878 | normal | normal | down |
| 152 | efu-miR-7a | CUGGAAGACUAGUGAUUUUGUUGUU | 25 | 1264.576 | 565.0788 | 732.3351 | down | normal | normal |
| 153 | efu-miR-7c | UGGAAGACUAGUGAUUUUGUUGUUC | 25 | 1273.917 | 563.6887 | 729.4057 | down | normal | normal |
| 154 | fru-miR-7 | UGGAAGACUAGUGAUUUUGUU | 21 | 1257.278 | 559.7501 | 726.1834 | down | normal | normal |
| 155 | gga-miR-125b-3p | ACAAGUCAGGCUCUUGGGACCU | 22 | 16.93107 | 49.11714 | 35.73795 | up | up | normal |
| 156 | gga-miR-365-1-5p | GAGGGACUUUUGGGGGCAGAUGU | 23 | 25.98044 | 9.49907 | 16.11137 | down | normal | normal |
| 157 | gga-miR-365-2-5p | GAGGGACUUUCAGGGGCAGCUGU | 23 | 4.670641 | 1.158423 | 3.222274 | down | normal | normal |
| 158 | gga-miR-99a-3p | CAAGCUCGCUUCUAUGGGUCU | 21 | 2.043405 | 7.645593 | 5.565746 | up | normal | normal |
| 159 | ggo-let-7c | UGAGGUAGUAGGUUGUAUGGU | 21 | 1360.908 | 4193.26 | 2557.607 | up | normal | normal |
| 160 | ggo-miR-1298 | UUCAUUCGGCUGUCCAGAUG | 20 | 21.60171 | 101.4779 | 297.328 | up | up | up |
| 161 | hsa-miR-127-5p | CUGAAGCUCAGAGGGCUCUGAU | 22 | 23.93704 | 48.42209 | 37.49555 | up | normal | normal |
| 162 | hsa-miR-136-3p | CAUCAUCGUCUCAAAUGAGUCU | 22 | 76.77366 | 183.7259 | 119.81 | up | normal | normal |
| 163 | hsa-miR-365b-5p | AGGGACUUUCAGGGGCAGCUGU | 22 | 4.670641 | 1.158423 | 3.222274 | down | normal | normal |
| 164 | hsa-miR-379-3p | UAUGUAACAUGGUCCACUAACU | 22 | 11.38469 | 23.40015 | 16.69724 | up | normal | normal |
| 165 | hsa-miR-431-5p | UGUCUUGCAGGCCGUCAUGCA | 21 | 20.43405 | 53.51915 | 36.03088 | up | normal | normal |
| 166 | hsa-miR-6516-3p | AUCAUGUAUGAUACUGCAAACA | 22 | 1.75149 | 5.328747 | 4.101076 | up | normal | normal |
| 167 | hsa-miR-92a-1-5p | AGGUUGGGAUCGGUUGCAAUGCU | 23 | 8.465537 | 0.926739 | 1.46467 | down | down | normal |
| 168 | ipu-miR-18a | UAAGGUGCAUCUAGUGCAGA | 20 | 13.72001 | 6.950539 | 6.151614 | normal | down | normal |
| 169 | mdo-miR-125b-2-3p | ACGGGUUAGGCUCUUGGGAGC | 21 | 6.714046 | 15.52287 | 15.5255 | up | up | normal |
| 170 | mdo-miR-181a-1-3p | CCAUCGACCGUUGAUUGUACC | 21 | 40.86811 | 19.92488 | 41.59663 | down | normal | up |
| 171 | mdo-miR-30a-3p | UUUCAGUCGGAUGUUUGCAGC | 21 | 2.627236 | 7.645593 | 7.616285 | up | up | normal |
| 172 | mml-miR-127-5p | UGAAGCUCAGAGGGCUCUGAUU | 22 | 23.64512 | 48.1904 | 36.90969 | up | normal | normal |
| 173 | mml-miR-7180-3p | UGGCCUCUGGGUGUGUACCCU | 21 | 2.043405 | 2.780216 | 6.444548 | normal | up | normal |
| 174 | mmu-miR-1298-3p | CAUCUGGGCAACUGAUUGAACU | 22 | 0 | 0.926739 | 4.979878 | normal | up | up |
| 175 | mmu-miR-136-3p | AUCAUCGUCUCAAAUGAGUCUU | 22 | 76.77366 | 183.2625 | 118.9312 | up | normal | normal |
| 176 | mmu-miR-144-5p | GGAUAUCAUCAUAUACUGUAAGU | 23 | 4.670641 | 28.03384 | 10.83856 | up | up | down |
| 177 | mse-let-7a | UGAGGUAGUAGGUUGUAUAG | 20 | 250.755 | 712.4303 | 607.5452 | up | up | normal |
| 178 | oan-miR-205-5p | UCCUUCAUUCCACCGGAGUCU | 21 | 4.670641 | 2.780216 | 12.8891 | normal | up | up |
| 179 | oar-miR-10a | UACCCUGUAGAUCCGAAUUUG | 21 | 6.714046 | 19.22982 | 4.979878 | up | normal | down |
| 180 | oar-miR-3956-5p | GUACGUGGAUGCUGAAGGUCAGA | 23 | 24.81278 | 35.21606 | 16.40431 | normal | normal | down |
| 181 | oar-miR-665-3p | ACCAGUAGGCCGAGGCCCCUCA | 22 | 10.21703 | 18.30309 | 21.09125 | normal | up | normal |
| 182 | oar-miR-99a | AACCCGUAGAUCCGAUCUUG | 20 | 3856.49 | 11467.69 | 6584.864 | up | normal | normal |
| 183 | oha-miR-27b-5p | AGAGCUUAGCUGAUUGGUGAACAG | 24 | 25.68853 | 8.572331 | 16.69724 | down | normal | normal |
| 184 | ola-miR-100 | AACCCGUAGAUCCGAACUU | 19 | 1188.678 | 2497.56 | 2533.293 | up | up | normal |
| 185 | ola-miR-181a-5p | AACAUUCAACGCUGUCGGU | 19 | 118.2256 | 234.2332 | 238.7412 | normal | up | normal |
| 186 | ola-miR-204 | UUCCCUUUGUCAUCCUAUGC | 20 | 30.35917 | 109.3551 | 67.08189 | up | up | normal |
| 187 | ola-miR-24b-3p | UGGCUCAGUUCAGCAGGA | 18 | 55.46386 | 121.6344 | 111.6079 | up | up | normal |
| 188 | ola-miR-27d-3p | UUCACAGUGGCUAAGUUC | 18 | 0.875745 | 5.560431 | 4.101076 | up | up | normal |
| 189 | pma-miR-199a-3p | CAGUAGUCUGCACAUUGGUUA | 21 | 2684.743 | 6771.215 | 5033.192 | up | normal | normal |
| 190 | pma-miR-20a-5p | CAAAGUGCUUAUAGUGCAGGUAG | 23 | 12.26043 | 5.328747 | 3.515208 | down | down | normal |
| 191 | rno-miR-127-5p | CUGAAGCUCAGAGGGCUCUGAUU | 23 | 23.93704 | 48.42209 | 37.49555 | up | normal | normal |
| 192 | rno-miR-204-3p | GCUGGGAAGGCAAAGGGACGUU | 22 | 1.16766 | 6.718854 | 4.101076 | up | normal | normal |
| 193 | rno-miR-379-3p | CUAUGUAACAUGGUCCACUAAC | 22 | 11.38469 | 23.40015 | 16.69724 | up | normal | normal |
| 194 | sha-miR-181a-3p | ACCAUCGACCGUUGAUUGU | 19 | 41.16002 | 20.38825 | 42.47543 | down | normal | up |
| 195 | ssa-miR-144-5p | GGAUAUCAUCAUAUACUGUAAGUU | 24 | 1.75149 | 12.51097 | 4.979878 | up | normal | down |
| 196 | ssc-miR-339 | UCCCUGUCCUCCAGGAGCUCA | 21 | 14.30384 | 46.33693 | 41.59663 | up | up | normal |
| 197 | ssc-miR-382 | AAGUUGUUCGUGGUGGAUUCG | 21 | 70.64345 | 144.1078 | 83.19326 | up | normal | normal |
| 198 | tch-miR-30a-5p | UGUAAACAUCCUCGACUGGA | 20 | 200.5456 | 461.5158 | 401.3196 | up | up | normal |
| 199 | tgu-miR-365-1-5p | GGGACUUUUGGGGGCAGAUGUGU | 23 | 25.1047 | 9.267385 | 15.81844 | down | normal | normal |
| 200 | CM008008.1_10340 | AAAAACCAGAACGAACUUUGUG | 22 | 11.96852 | 10.88918 | 5.272812 | normal | down | down |
| 201 | CM008010.1_21055 | CGAGGCACUUAACUCUGGGCU | 21 | 1.75149 | 0 | 0 | down | normal | -- |
| 202 | CM008011.1_33842 | CCCCGCCUCCCGCUCUCC | 18 | 1.16766 | 1.853477 | 12.59616 | normal | up | up |
| 203 | CM008011.1_37433 | UGAGUGUGUGUGUGUGAGC | 19 | 1.459575 | 5.328747 | 2.636406 | up | normal | normal |
| 204 | CM008012.1_46261 | UGAGUGUGUGUGUGUGAGC | 19 | 3.502981 | 7.645593 | 5.272812 | up | normal | normal |
| 205 | CM008012.1_46537 | AUAACCGUAACUUUGAAAUGCU | 22 | 0 | 1.158423 | 3.808142 | normal | up | normal |
| 206 | CM008012.1_48395 | AGAUGUGAUGACCUUCUGAGG | 21 | 8.757452 | 2.316846 | 4.101076 | down | down | normal |
| 207 | CM008012.1_55433 | GUGGGCUUCCCUGGUGGCUCAGA | 23 | 1.75149 | 19.69319 | 185.1343 | up | up | up |
| 208 | CM008015.1_78022 | GUGCUGUUUUCUCUUCAGUC | 20 | 0 | 0 | 2.92934 | -- | up | up |
| 209 | CM008015.1_83294 | UGGGAAAGAAUCUGCCUGC | 19 | 0 | 0.695054 | 10.54562 | normal | up | up |
| 210 | CM008016.1_84545 | GUGGGCUUCCCUGGUGGCGCAGAC | 24 | 0.291915 | 1.158423 | 7.909219 | normal | up | up |
| 211 | CM008016.1_95226 | UAUUUUCAUGACAUUUUUCUGA | 22 | 3.794896 | 0.695054 | 2.92934 | down | normal | normal |
| 212 | CM008018.1_117444 | AUCCCACUUCUGACACCAAG | 20 | 7.005961 | 13.43771 | 18.45484 | normal | up | normal |
| 213 | CM008019.1_131447 | AAAACACGAACAAACUUUUCGG | 22 | 11.09277 | 17.83972 | 6.444548 | normal | normal | down |
| 214 | CM008019.1_137729 | CCCAGGGAUGUAGCUCCUAGUGC | 23 | 1.459575 | 4.633693 | 1.171736 | up | normal | down |
| 215 | CM008020.1_143069 | ACGCACAGCGCCUCACUGAGC | 21 | 2.919151 | 8.804016 | 7.030417 | up | up | normal |
| 216 | CM008020.1_143075 | CACAAUACACGGGUCGGCCUCU | 22 | 27.1481 | 11.81592 | 19.04071 | down | normal | normal |
| 217 | CM008021.1_148608 | UUGGCCUACAGAAAUGACAGACA | 23 | 3.794896 | 6.255485 | 1.757604 | normal | normal | down |
| 218 | CM008021.1_150571 | GCACGUGAUGUGUGGGCUGAUG | 22 | 12.55235 | 2.780216 | 3.515208 | down | down | normal |
| 219 | CM008021.1_154122 | AAAAAACCGAGUGAACUUUUUG | 22 | 35.32172 | 61.39643 | 24.60646 | normal | normal | down |
| 220 | CM008022.1_159134 | CAAAUUCGUGAAGCGUUCCAUAUUU | 25 | 20.72597 | 7.182224 | 16.69724 | down | normal | up |
| 221 | CM008022.1_166506 | AUGCGGUAGAGCAUCAGAGA | 20 | 0.875745 | 5.328747 | 2.636406 | up | normal | normal |
| 222 | CM008022.1_171045 | CUGGAGCUCUGGGUUCCUGCUUC | 23 | 7.297877 | 0.695054 | 1.757604 | down | down | normal |
| 223 | CM008023.1_177547 | AAAAGUUCAUUCGGGUUCCUCU | 22 | 0 | 0.926739 | 2.636406 | normal | up | normal |
| 224 | CM008024.1_183606 | AAAACCUGAACUAACUUUUUGA | 22 | 10.80086 | 5.328747 | 4.979878 | down | down | normal |
| 225 | CM008025.1_184228 | CAAAUUCGUGAAGCGUUCCAUAUUU | 25 | 20.72597 | 7.182224 | 16.99017 | down | normal | up |
| 226 | CM008025.1_184735 | ACUGCCUGGAUAAGAAUCAGC | 21 | 0.58383 | 1.158423 | 3.515208 | normal | up | normal |
| 227 | CM008025.1_193290 | CAGGCGGGUGCUGAUGCGAUC | 21 | 8.173622 | 9.035701 | 3.515208 | normal | down | down |
| 228 | CM008026.1_201442 | CACCGGCAUCGUGAUGGACU | 20 | 0 | 0.695054 | 2.636406 | normal | up | normal |
| 229 | CM008027.1_215935 | AAAAACCUGAAUGACCCUUUU | 21 | 30.35917 | 65.56675 | 45.11184 | up | normal | normal |
| 230 | CM008029.1_236567 | ACGCCCUUCCCCCCCUUCUUCA | 22 | 6.130216 | 3.0119 | 7.323351 | normal | normal | up |
| 231 | CM008029.1_238965 | AAAACCUGAACUAACUUUUUGA | 22 | 10.21703 | 5.097062 | 4.39401 | down | down | normal |
| 232 | CM008032.1_269285 | AAAAGUUAGCUCGGAUUUUUCU | 22 | 2.919151 | 0.463369 | 0.878802 | down | normal | normal |
| 233 | CM008033.1_276646 | GGGGCUUCCCUGGUGGCUCAGA | 22 | 0.291915 | 0.926739 | 8.495087 | normal | up | up |
| 234 | CM008034.1_284828 | GUGGGCUUCCCUGGUAGCUCAGC | 23 | 3.211066 | 6.48717 | 84.07206 | normal | up | up |
| 235 | CM008037.1_302513 | GUGGGCUUCCCUGGUGGCUCAGA | 23 | 1.75149 | 19.69319 | 184.8414 | up | up | up |
| 236 | CM008038.1_306877 | UAGAUGAAAAGAUCUCAGGACU | 22 | 7.005961 | 3.243585 | 6.151614 | down | normal | normal |
| 237 | CM008038.1_307839 | CCGGUUUCUGUUGCCAAGGCGCC | 23 | 2.33532 | 0 | 0 | down | down | -- |
| 238 | CM008039.1_313741 | AUCCCGGACGAGCCCCCC | 18 | 4.086811 | 6.0238 | 11.71736 | normal | up | normal |
| 239 | CM008039.1_315920 | CGGAUCAGCUCAGUGCCGGGC | 21 | 0 | 3.0119 | 0 | up | -- | down |
| 240 | CM008040.1_317341 | GACAUGACUGAGUGACUUUCACU | 23 | 7.881707 | 12.27929 | 2.92934 | normal | down | down |
| 241 | CM008041.1_329491 | GUGGGCUUCCCUGGUGGCUCAGA | 23 | 1.75149 | 19.69319 | 184.8414 | up | up | up |
| 242 | CM008041.1_332283 | AAGAUGUAUGAUGUGAUGAUUU | 22 | 23.93704 | 18.99814 | 11.13149 | normal | down | normal |
| 243 | MKHE01004964.1_338157 | CUGCACUGCAUGGUAUCUGC | 20 | 2.919151 | 5.792116 | 1.757604 | normal | normal | down |
