# Supplementary figures and images for: RNA sequencing-based identification of microRNAs in the antler cartilage of Gansu red deer (Cervus elaphus kansuensis)
Source: PeerJ. 2022 Sep 21;10:e13947. doi: 10.7717/peerj.13947 (PMC9508884; doi:10.7717/peerj.13947)

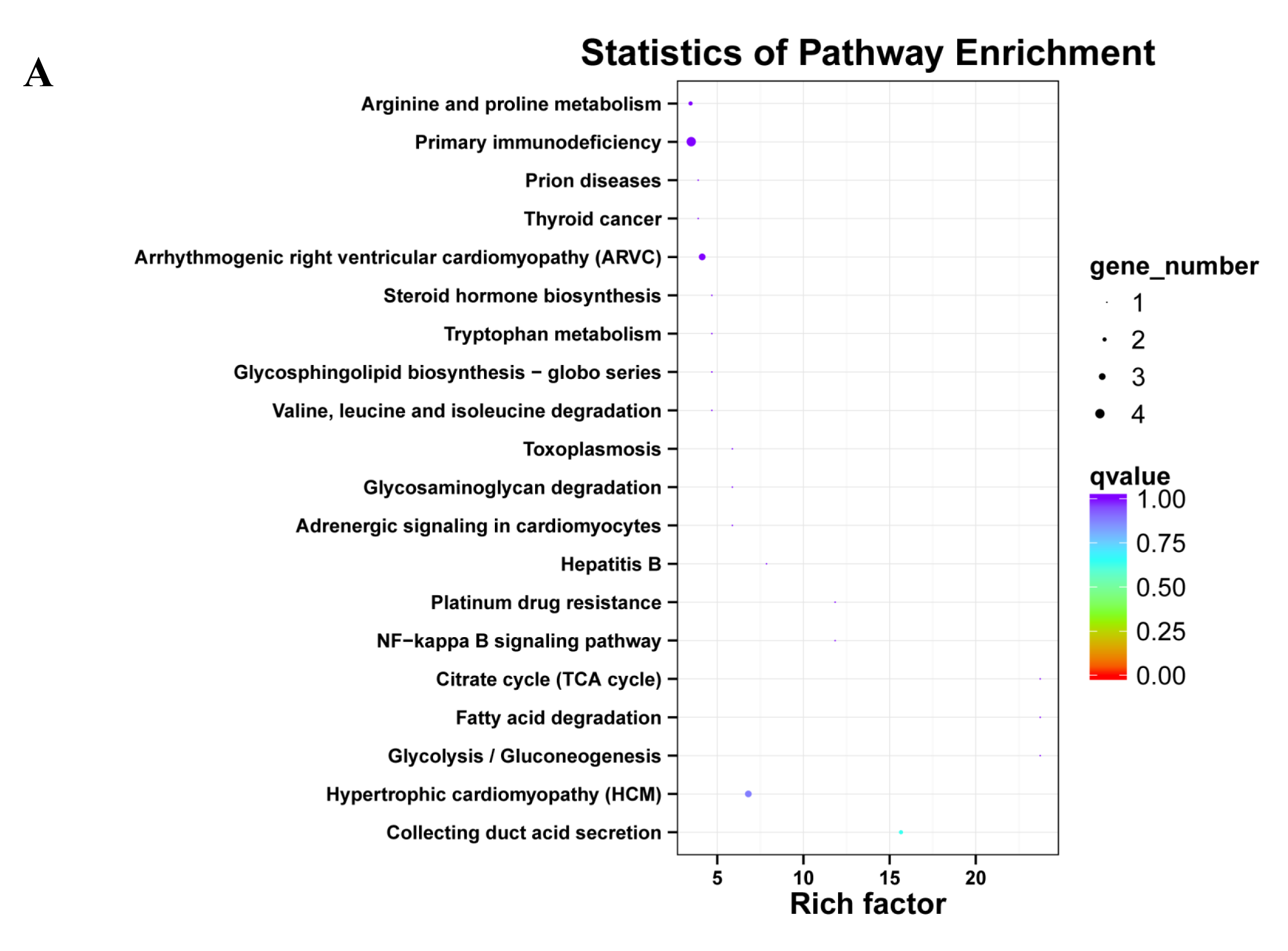

Supplement: Figure S1A [file peerj-10-13947-s008.png]

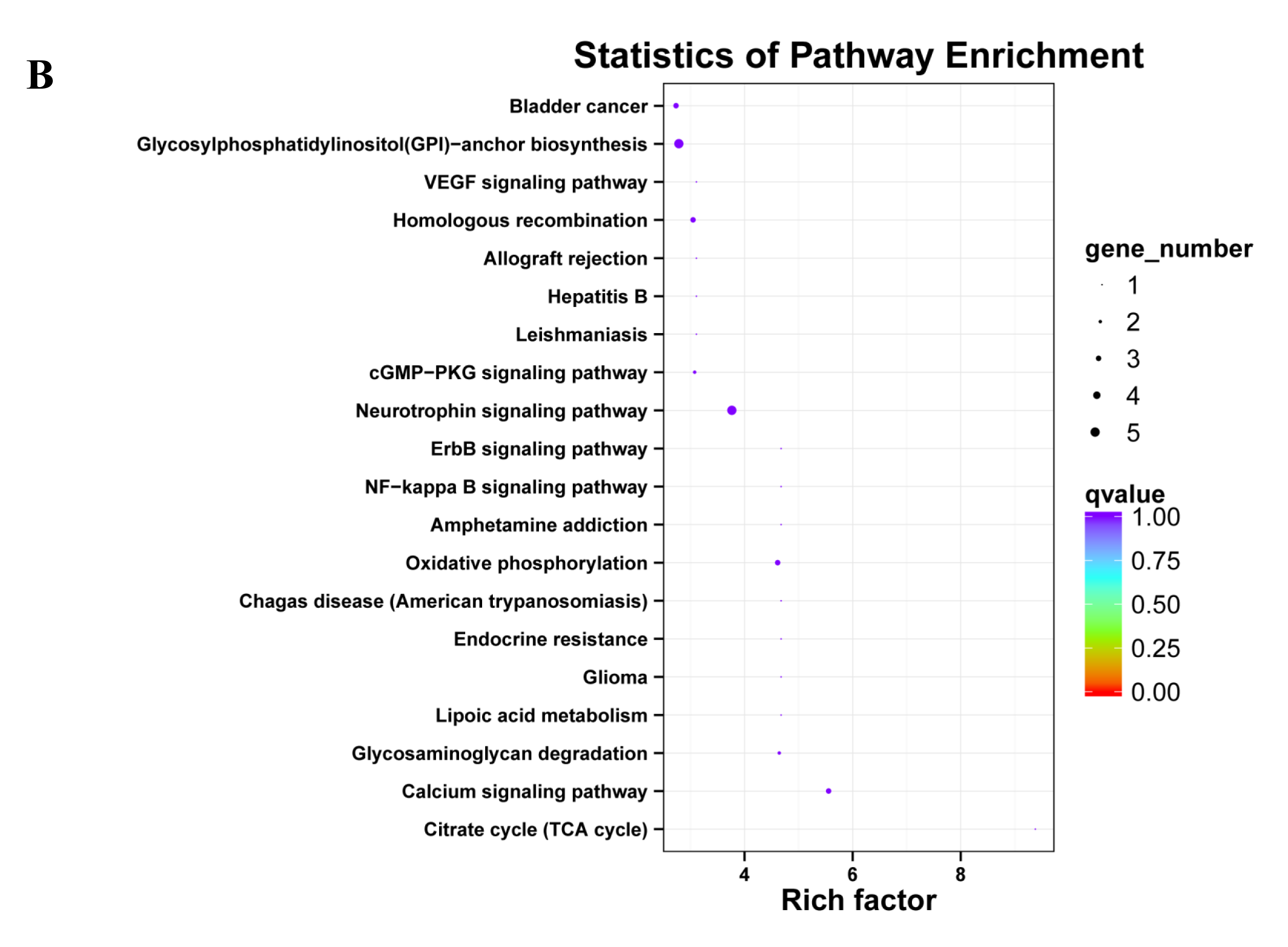

Supplement: Figure S1B [file peerj-10-13947-s009.png]

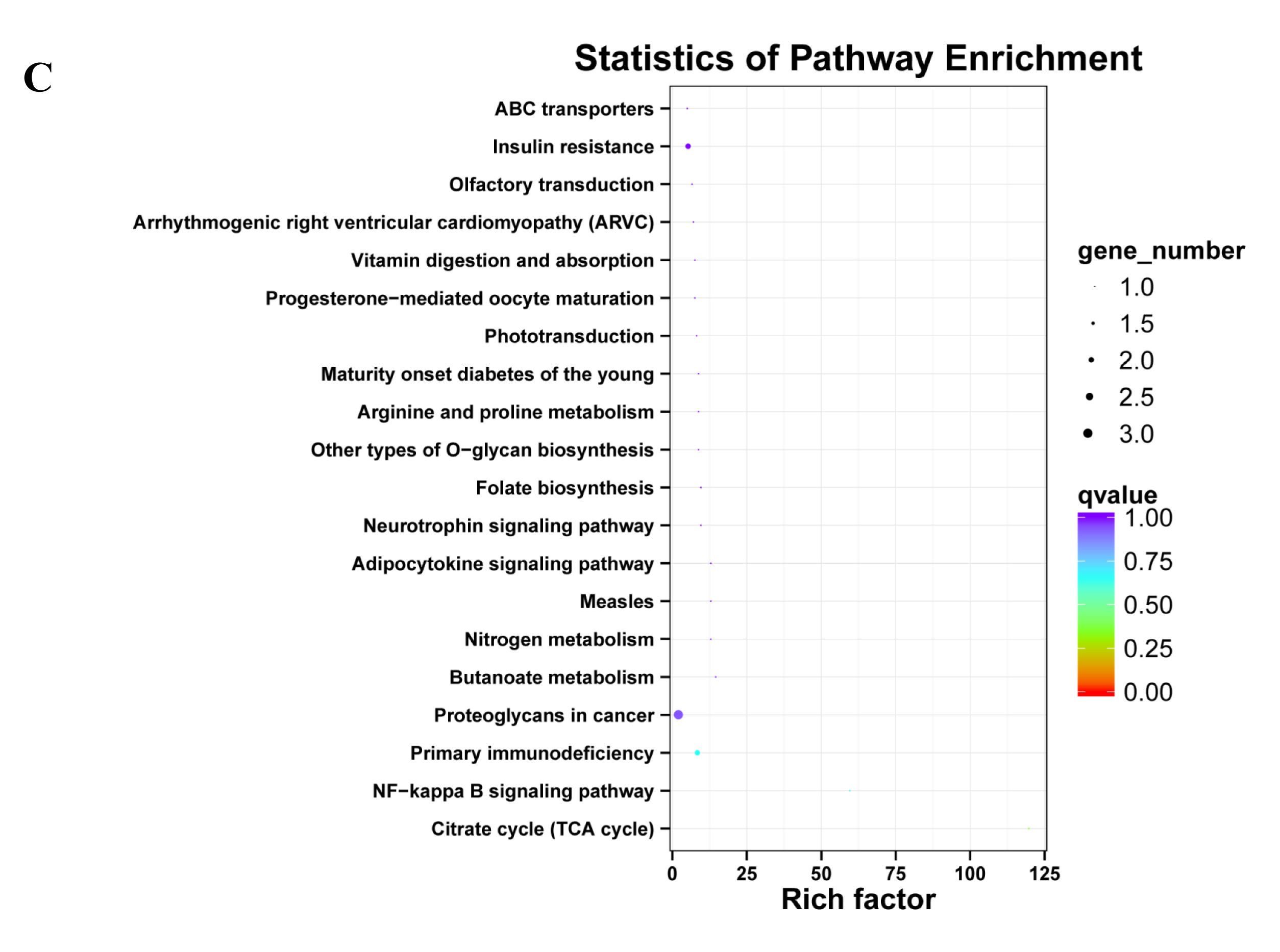

Supplement: Figure S1C [file peerj-10-13947-s010.png]
